# Violet-light-induced ring expansion of 2-aryl-1,3-indandiones with chlorodiazirines toward 1,4-naphthoquinones

**DOI:** 10.1039/d6sc04532d

**Published:** 2026-07-24

**Authors:** Bao-Jie Wang, Xinyu Zhang, Huimin Liang, Li-Ming Zhang, Weisi Guo, Lin-Bao Zhang

**Affiliations:** a State Key Laboratory Base of Eco-Chemical Engineering, College of Chemistry and Molecular Engineering, Qingdao University of Science and Technology Qingdao 266042 P. R. China zhang_linbao@126.com; b Jinan Vocational College Automotive Engineering Department P. R. China xqzhangliming@163.com

## Abstract

1,4-Naphthoquinones are widely used in medicine and materials, but noble metal catalysts and harsh conditions limit their synthesis. We report a violet-light-induced skeletal editing strategy that enables ring-expansion of 2-aryl-1,3-indandiones *via* single-carbon insertion using chlorodiazirines as carbene precursors. This protocol proceeds without metal catalysts or ligands, featuring broad substrate scope, excellent selectivity, high efficiency under mild conditions, and moderate to good yields. Mechanistic studies indicate that the reaction proceeds *via* carbene [2 + 1] cycloaddition, followed by ring expansion.

## Abstract 2

We present a metal-free photochemical skeleton editing strategy, wherein the single-carbon insertion and ring expansion of 2-aryl-1,3-indandiones with chlorodiazirines affords 1,4-naphthoquinones (1,4-NQs) with high efficiency under mild conditions.

## Graphical abstract

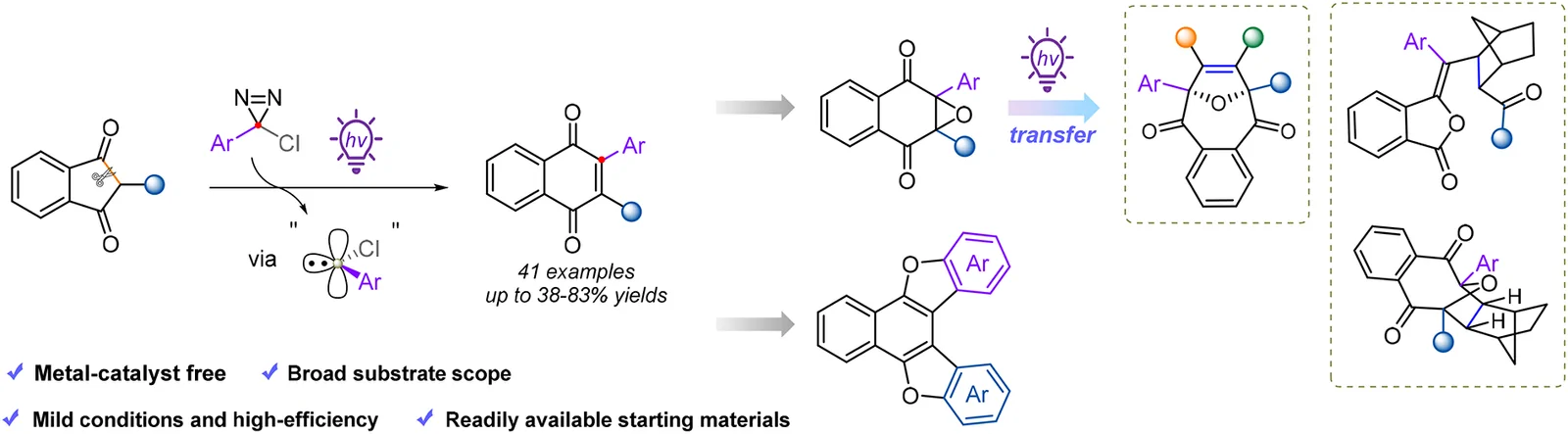

## Introduction

1,4-Naphthoquinones (1,4-NQs) have been extensively studied in the pharmaceutical and materials fields due to their unique structure and reactivity.^1–10^ In organic synthesis, 1,4-naphthoquinones are also highly important intermediates,^11–21^ serving as key building blocks for complex molecules and functional materials.

During the past few decades, various synthetic strategies have been developed for 1,4-NQs (Scheme 1a), including the Diels–Alder reaction of substituted 1,3-dienes with preformed quinones (path a),^22^ the thermal cycloisomerization of cyclobutenones (path b),^23,24^ the single-electron transfer oxidation of substituted naphthalenes accompanied by 1,2-substituent migration (NIH shift) (path c),^25^ the reaction of alkynes with organotransition metal complexes (including metal carbonyl complexes^26–28^ and Fischer carbene complexes^29–34^) (path d), and the Cu^2+^-catalyzed intramolecular oxidative 6-*exo*-trig cyclization of 1,6-enynes (path e).^35^ Although these methods have achieved moderate success, they generally suffer from transition-metal usage, strict substrate prefunctionalization, harsh conditions and tedious procedures, restricting their practical applications. Therefore, a metal-free step-economic route to 1,4-NQ skeletons is synthetically valuable.

**Scheme 1:**
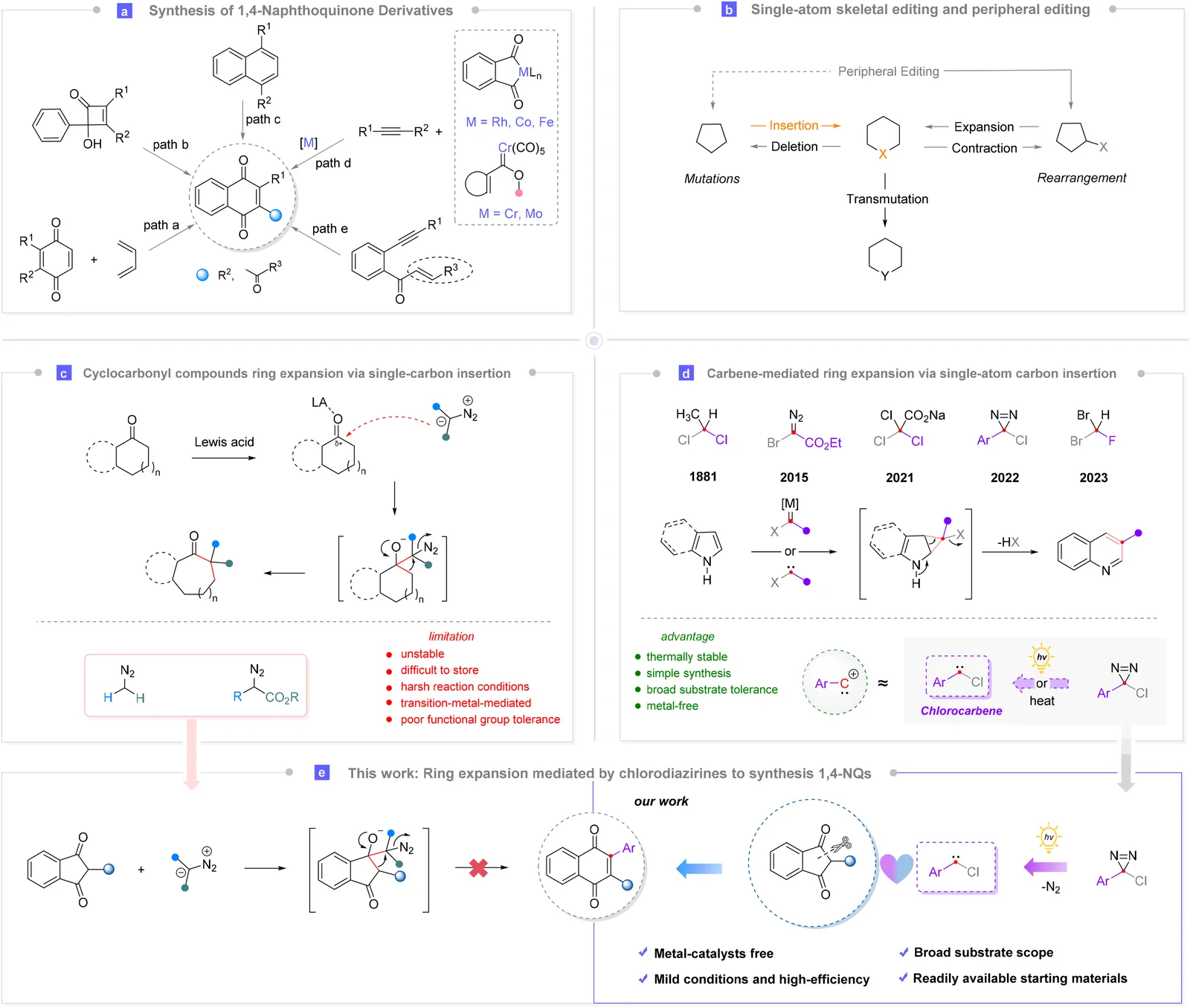
Research background and our work.

Recently, single-atom skeletal editing has garnered widespread attention for precise modification of molecular core skeletons.^36–40^ Unlike traditional peripheral editing, which only modifies functional groups at the molecular periphery, skeletal editing enables direct reconstruction of the molecular core *via* atom insertion, deletion, or transmutation (Scheme 1b), thereby altering its physicochemical properties. This strategy dramatically shortens synthetic steps, enabling rapid access to complex core skeletons that are difficult to prepare conventionally. Among these, insertion reactions can elongate saturated carbon chains and enable ring expansion, with ring expansion in particular attracting extensive interest. Motivated by this widespread interest, researchers have established that single-carbon ring expansion *via* insertion of diazo species (*e.g.*, diazomethane and α-diazoesters) into cyclocarbonyl compounds allows access to core cyclic scaffolds^41–43^ (Scheme 1c, above). This strategy is highly appealing for the one-step construction of 1,4-NQs. However, these approaches suffer from transition-metal dependence, harsh conditions, and low functional-group tolerance (Scheme 1c, bottom). Crucially, the substituents attached to the central carbon of these diazo variants are poor leaving groups. Mechanistically, this favors the formation of saturated rings rather than 1,4-NQs (Scheme 1e, left). Thus, exploring alternative carbenes to diazo carbenes for superior synthetic routes is worthwhile.

Carbenes, highly reactive transient species, are widely used in single-carbon insertion for molecular skeletal editing.^44–49^ Within this field, diverse α-halocarbene precursors have been developed, including chloroform, α-halodiazoacetates,^50^ sodium trichloroacetate,^51^ chlorodiazirines,^48,52–56^ and dibromofluoromethane.^57,58^ These species readily yield halocarbenes under thermal, photochemical, or basic conditions. The generated halocarbenes undergo cyclopropanation at the 2,3-double bond of indoles and pyrroles, forming strained three-membered ring intermediates. Subsequent charge-triggered ring opening achieves mild aromatic heterocycle ring expansion and significantly promotes this research field (Scheme 1d, above). Among them, chlorodiazirines draw our attention due to their stability and accessibility. Importantly, separate research groups led by Levin,^55,56^ Ball^52^ and Suero^48^ have all reported that chlorodiazirines serve as reactive equivalents of cationic carbynes (:^+^C–R) or carbynoids with dual carbene/carbocation behavior (Scheme 1d, bottom), whose electron-deficient carbene center with a suitable leaving group facilitates C–C bond cleavage and single-carbon insertion-enabled ring expansion.

Driven by this rationale, we explored readily accessible 2-aryl-1,3-indandiones as substrates, aiming to construct 1,4-NQs in one step through a single-carbon insertion ring-expansion approach. Interestingly, Zhang *et al.*, in 2023, achieved the single-carbon insertion ring-expansion of 2-aryl-1,3-indandiones by employing alkenes as an external carbon source to access 1,4-NQs.^59^ However, this remains limited to metal catalysis and elevated temperatures. In contrast, visible light as the sole energy source enables mild transformations to bypass the limitations of conventional chemistry, attracting widespread interest for its green and highly selective routes toward complex molecules and pharmaceutical scaffolds.^60–63^ To date, no visible-light-driven, metal-free single-carbon insertion ring expansion has been reported for their synthesis. The C2–H of 2-aryl-1,3-indandiones is acidic and readily deprotonated to form enolates. Given the reactivity of carbenes toward alkenes, we speculated that photochemically derived electrophilic carbenes from chlorodiazirines could undergo [2 + 1] cycloaddition with enolates. Oxygen anion-driven C–C bond cleavage and rearrangement then enable single-carbon insertion to form 1,4-naphthoquinones, as confirmed by further mechanistic studies. Herein, we report the first metal-free protocol for the violet-light-induced carbon-insertion ring expansion of readily available 2-aryl-1,3-indandiones with chlorodiazirines, enabling the mild and highly efficient synthesis of 1,4-NQs (Scheme 1e, right).

## Results and discussion

### Optimization of reaction conditions

To establish the optimal reaction conditions, we initiated our investigation by using 1a and 2a as model substrates (Table 1). After systematic screening of reaction conditions, including the base and solvent (Tables S1–S5), product 3a was obtained in 81% yield with KHMDS as the base and Et_2_O/DME as the mixed solvent under violet light (390 nm, 25 W) irradiation at room temperature for 25 min. The unique structure of 1a, together with the control experiments (Table 1, entry 2), demonstrates that the base is indispensable for this transformation. No reaction proceeded without the base, and only byproducts 2a′ and 2a″ from decomposition-dimerization of 2a were detected. We thus speculate that the first step of the reaction is base-induced enolate formation. When using mild or strong inorganic bases (K_2_CO_3_, K_3_PO_4_, NaOH, and KOH), only a trace amount of product was obtained, and other bases gave decreased yields (Table S1). Solvent screening indicated that when the reaction was conducted in MeCN as the sole solvent, the yield was 30%, whereas toluene, Et_2_O, and other solvents produced inferior results (Table 1, entries 5, 6, 7, and 9). When DME was used alone without Et_2_O, the yield dropped sharply to 42% (entry 8). To further probe the feasibility of the reaction, violet light was also crucial (Table 1, entries 10 and 11). In addition, thermolysis of chlorodiazirine at 50 °C gave only a trace amount of product (Table 1, entry 12). Notably, extending the heating time to 12 h delivered a 56% yield (Table S5, entry 8), owing to the much slower generation of chlorocarbene at 50 °C compared with that under light irradiation. Under an argon atmosphere, 3a was obtained in 83% yield (Table 1, entry 13).

**Table 1:** Optimization of reaction conditions

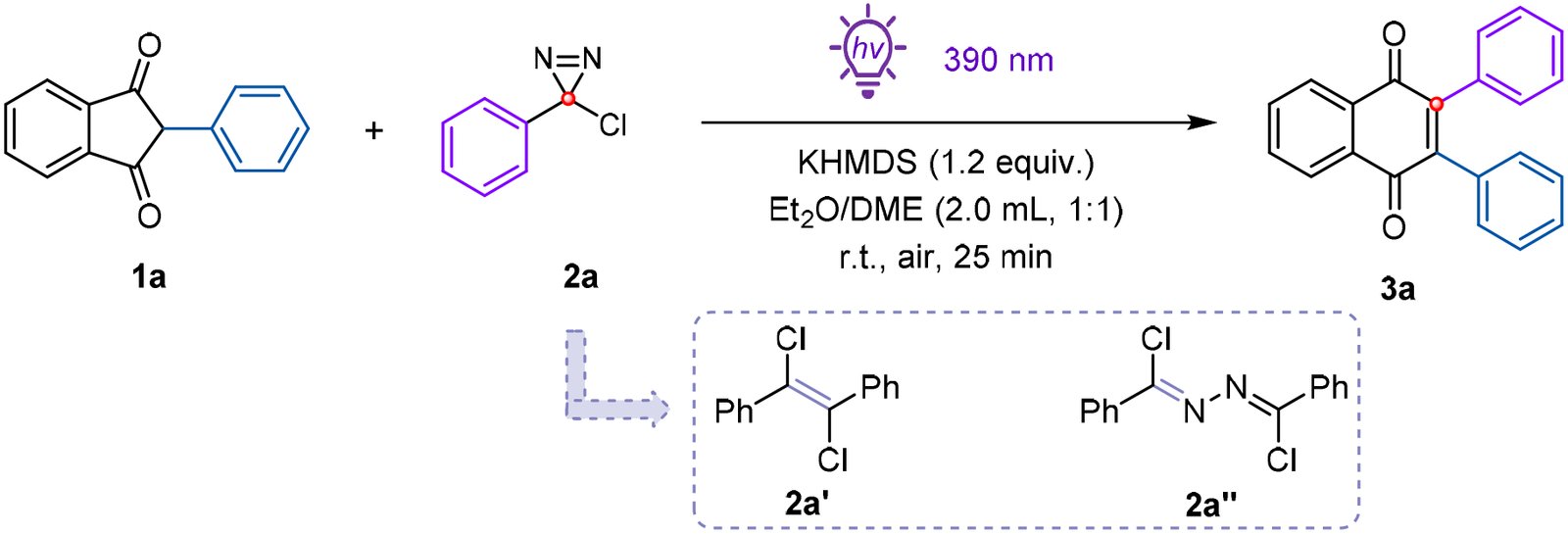		
Entry	Deviation from standard conditions^a^	Yield^b^ (%)
1	None	81
2	No base	n.r.^c^
3	K_2_CO_3_ or K_3_PO_4_ instead of KHMDS	Trace
4	NaOH or KOH instead of KHMDS	Trace
5	CH_3_CN as the solvent	30
6	Toluene as the solvent	Trace
7	Et_2_O as the solvent	Trace
8	DME as the solvent	42
9	Other solvents	<73
10	No light	n.r.^c^
11	410 nm instead of 390 nm	56
12	50 °C in darkness	Trace
13	In Ar	83

a Reaction conditions: 1a (0.1 mmol, 1.0 equiv.), 2a (0.3 mmol, 3.0 equiv.), KHMDS (0.12 mmol, 1.2 equiv.), Et_2_O/DME (2.0 mL, 1 : 1), r.t., air, and 25 min.

b Isolated yield.

c n.r. = no reaction.

With the optimal reaction conditions in hand, we explored the scope of phenindione derivatives 1 and diazirines 2 (Scheme 2). We first explored the scope of 2-aryl-1,3-indandiones 1 using 3-chloro-3-phenyl-3*H*-diazirine 2a as the reaction partner (Scheme 2, above). Product 3a was obtained in 81% yield, while ortho-substituted derivatives (3b–3f) afforded only moderate yields (40–58%), likely due to steric hindrance. Meta-substituted substrates (3g–3k) afforded good yields (76–82%), and the nitro group (NO_2_) was well tolerated. A wide range of *para*-substituted aryl groups, including halogens (F, Cl, Br, and I), alkyl substituents (Me and ^i^Pr), and both electron-donating and electron-withdrawing moieties (OMe and CF_3_), were well tolerated (3l–3t, 58–82% yields), with sensitive groups such as cyano (CN, 3r) and ester (COOMe, 3u) also proving compatible. Heteroaryl and fused-aromatic substrates, including benzodioxane, thiophene, pyridine and naphthalene derivatives, proceeded smoothly to give 3v–3z in 42–82% yields. By contrast, electron-donating aliphatic cyclohexyl (3aa) and cyclopropyl (3ab) substituents displayed lower reactivity, affording only 40–42% yields, likely due to the ability of aromatic conjugation to stabilize the intermediate. Replacing the 2-aryl substituent with an electron-withdrawing acetyl group (Scheme S1, S1b) led to no product. This reveals poor substrate tolerance of the 2-position toward electron-withdrawing aliphatic groups. Polysubstituted aryl substrates 3ac and 3ad were also evaluated, with markedly different yields, suggesting that the combined electronic and steric effects govern the reaction efficiency.

**Scheme 2:**
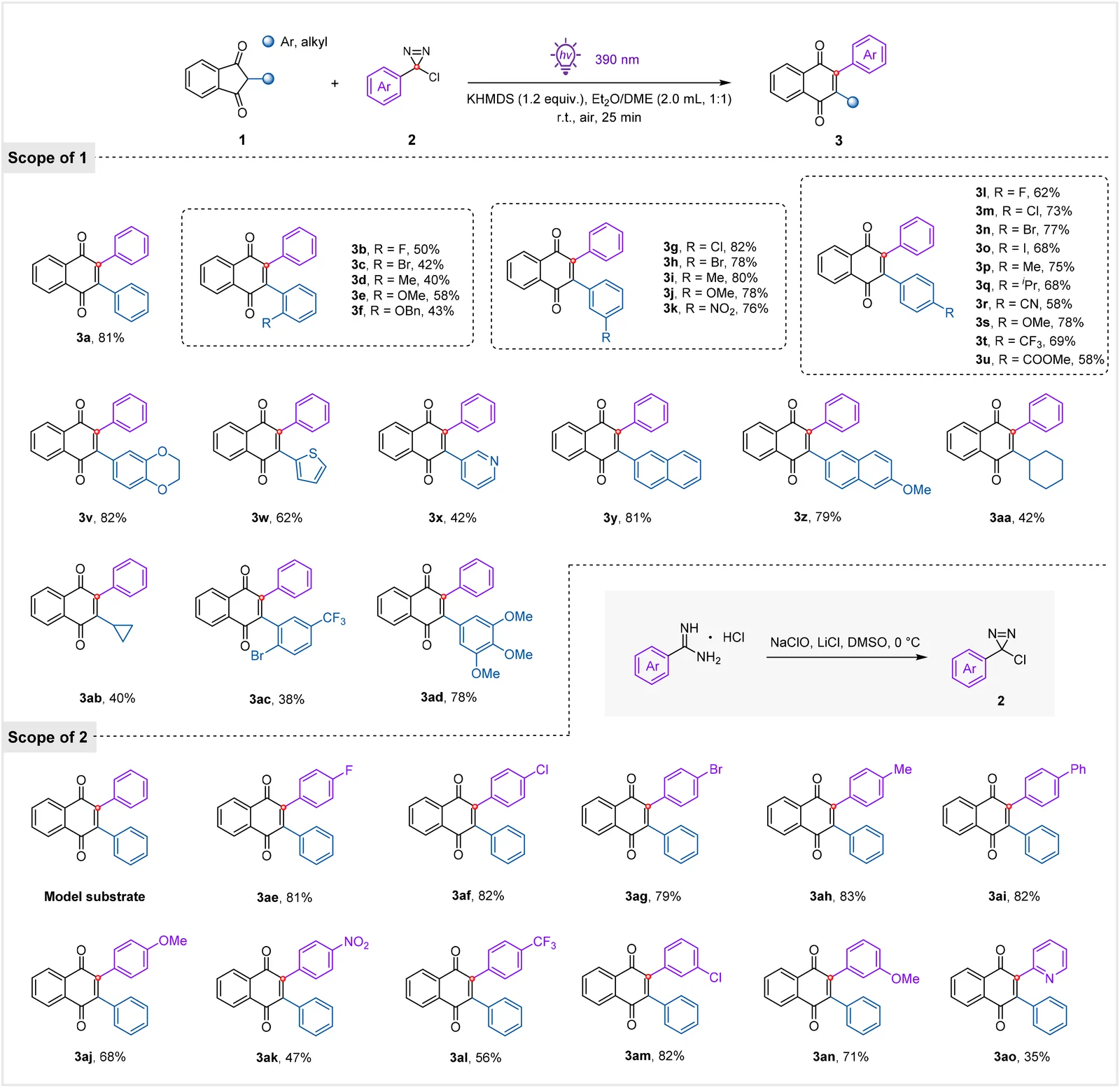
Substrate scope. ^*a*^Current reaction conditions: 1 (0.1 mmol, 1.0 equiv.), 2a (0.3 mmol, 3.0 equiv.), KHMDS (0.12 mmol, 1.2 equiv.), Et_2_O/DME (2.0 mL, 1 : 1), r.t., air, and 25 min. ^*b*^Isolated yield. ^*c*^n.r. = no reaction.

We next examined the scope of aryl chlorodiazirines 2 using 2-aryl-1,3-indandione 1a as the reaction partner (Scheme 2, bottom). Para-substituted phenyl substrates, regardless of electron-withdrawing (halogen, CF_3_, and NO_2_) or electron-donating (Me, Ph, and OMe) groups, were well tolerated, affording 3ae–3al in 47–83% yields. Meta-substituted aryl substrates also reacted efficiently, delivering 3am and 3an in good yields, and a pyridyl substrate proved compatible, giving 3ao in 35% yield. Notably, the aryl chlorodiazirine precursors were conveniently prepared from the corresponding amidine hydrochlorides.

To further demonstrate the practicality of this synthetic protocol, a gram-scale reaction was performed using 1a (5.0 mmol) and 2a (15.0 mmol), affording product 3a (1.22 g) in an isolated yield of 78% (Scheme 3). Photolysis of 4 induces homolytic cleavage of the epoxide C–C bond and ring expansion to form a seven-membered cyclic diradical. The diradical tautomerizes to 3,6-dioxooxepinium-7-ylide 4A, which undergoes [3 + 2] cycloaddition with ethyl propiolate and phenylacetylene to give 5 (52%) and 6 (49%). Upon irradiation of 4A with norbornene, the endo initial adduct 8 is formed in 37% yield, along with 7 in 31% yield; the latter arises from further photochemical rearrangement of 8. Notably, 3a can be converted to benzofuran-fused heteroarene 9 (51%) under the synergistic effect of TfOH and DDQ, and the structure of 9 was further confirmed by X-ray crystallography analysis (see the SI for details). This multifunctional rigid π-conjugated scaffold can be applied to optoelectronic functional materials, chiral catalysis and other related fields.^64–68^ To gain insight into the reaction mechanism, a series of mechanistic investigations were carried out (Scheme 4). Initially, violet-light photolysis of chlorodiazirines 2 yields singlet carbenes, which may undergo intersystem crossing (ISC) to triplet carbenes. To identify the key carbene spin state, TBACl was first employed to trap the singlet carbene. As expected, the model reaction was significantly suppressed, affording 3a in only 22% yield. In contrast, trapping the triplet carbene with TEMPO led to only marginal inhibition, indicating that the triplet carbene represents a minor reaction pathway (Scheme 4a). Therefore, the reaction is predominantly mediated by the singlet carbene. As noted earlier, the C2 proton of 1a is acidic due to the flanking carbonyl groups and the benzene ring, and the proton can be abstracted by a base to form a stable enolate. Deprotonation of 1a with KHMDS provided the corresponding enolate 10 as a red solid in 93% yield (Scheme 4b), and we confirmed the formation of the enolate. In contrast, sequestering K^+^ with 18-crown-6 afforded only a trace amount of product, as the enolate deprived of K^+^ stabilization switched from the oxyanion to the carbanion form and became unreactive toward the carbene (Scheme 4c). We speculate that the enolate C=C bond is the key reactive site toward the carbene. To confirm this inference, we used 11 instead of the enolate to react with 2a and observed a [2 + 1] cycloaddition of the carbene to the C=C bond to afford three-membered ring product 12 (Scheme 4d). In addition, the Hammett study also indirectly verified this conclusion. A series of *para*-substituted substrates 1 (R = –OMe, –^i^Pr, –H, –Br, –CN) were selected for competitive reaction experiments. The substituent effects were quantified using the Hammett linear free-energy relationship (LFER). As shown in Scheme 4e, the reaction rate constant (log(*k*_X_/*k*_H_)) exhibits a significant linear correlation with the substituent Hammett parameter (*σ*_P_), with a *ρ* value of −0.45. The negative *ρ* value points to a cationic transition state, where electron-donating substituents enhance reactivity, implying partial positive charge development during the reaction. This result reveals that the [2 + 1] cycloaddition, rather than enolate formation, is the rate-determining step, where electron density flows from the enolate double bond to the electron-deficient carbene, and the electron density at the double bond decreases to form the C–C σ-bond, rendering the transition state electropositive relative to the starting state and stabilized by electron-donating groups.

**Scheme 3:**
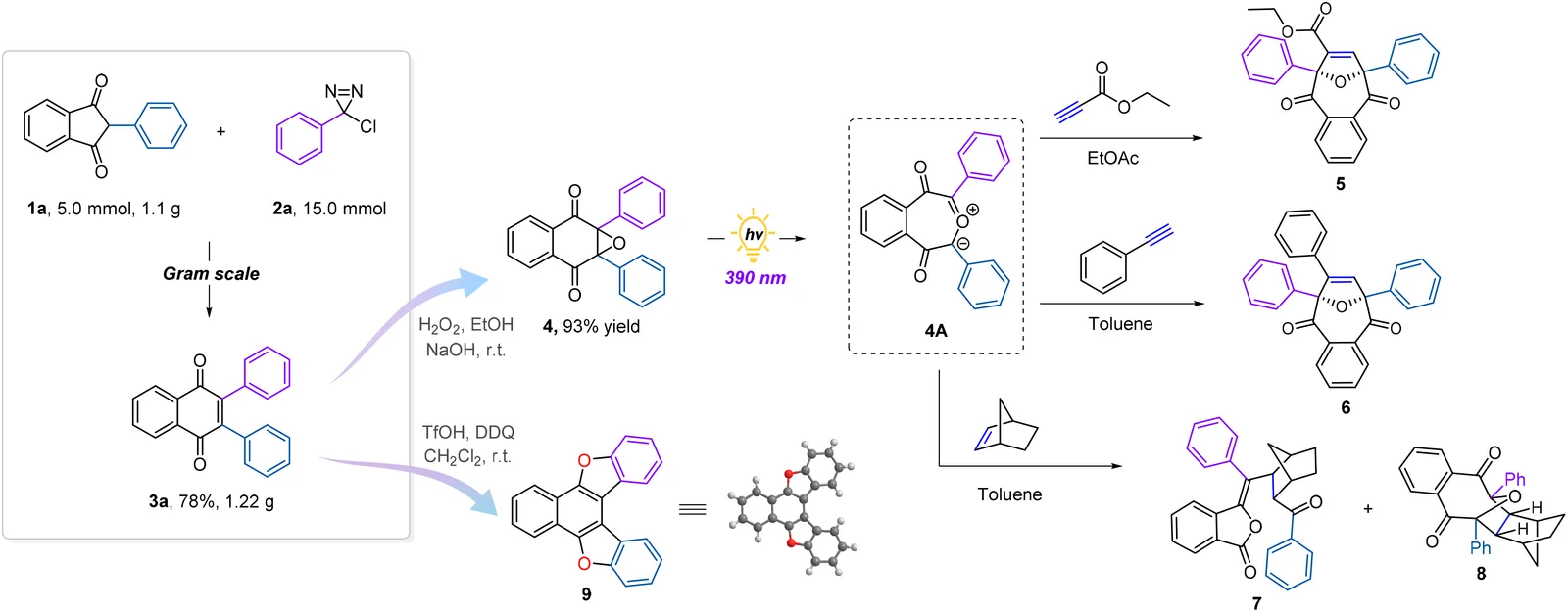
Gram-scale synthesis and follow-up transformation.

**Scheme 4:**
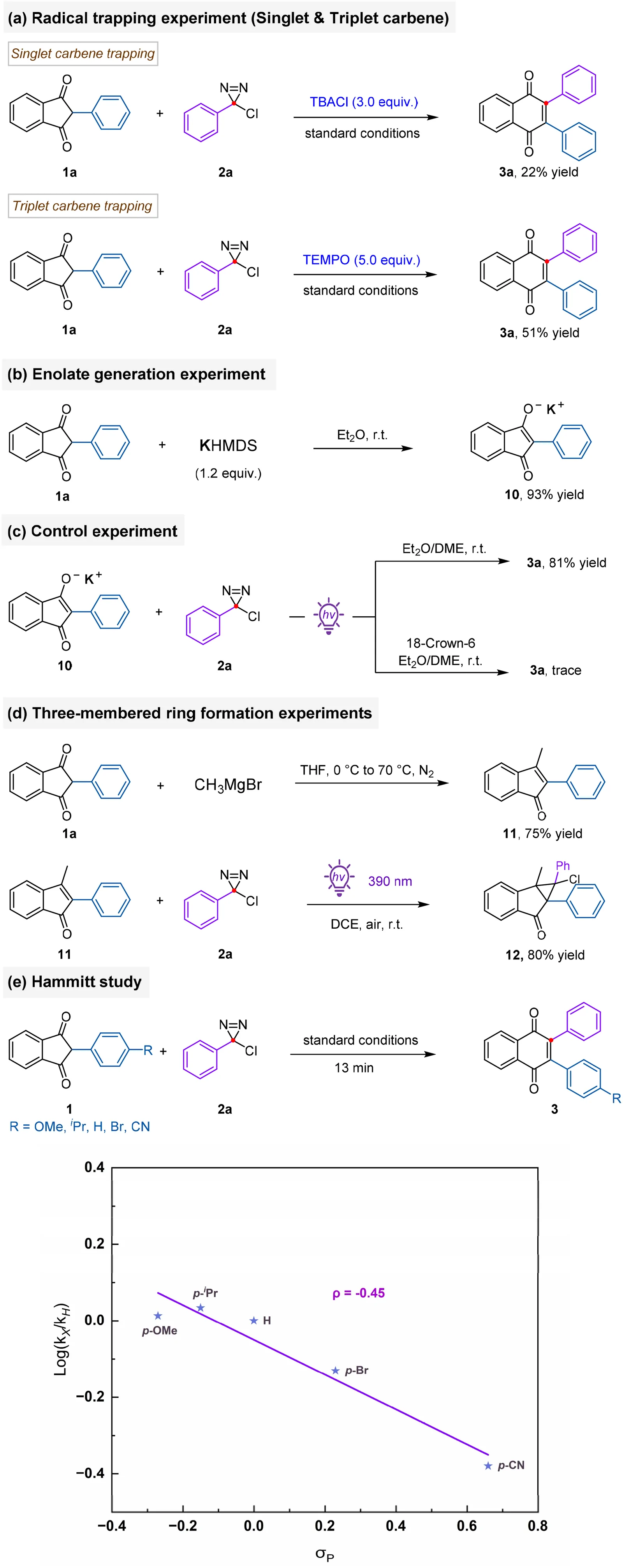
Mechanistic studies: (a) Radical trapping experiment (Singlet & Triplet carbene). (b) Enolate generation experiment. (c) Control experiment. (d) Three-membered ring formation experiments. (e) Hammitt study.

Based on the above experiments and previous studies,^49,52,55,56^ we propose a plausible mechanism in Scheme 5. Initially, chlorodiazirine 2a undergoes photolysis to generate a singlet carbene, which may undergo intersystem crossing (ISC) to the triplet carbene. 2-Aryl-1,3-indandione 1a is deprotonated by a base to form the enolate 10. The singlet carbene then attacks the C=C double bond of the enolate *via* a [2 + 1] cycloaddition, furnishing a strained three-membered ring intermediate Int B. Subsequent intramolecular rearrangement leads to ring-opening of the three-membered ring with concomitant chloride loss, ultimately delivering product 3a (major path). In contrast, for triplet carbene, the rigid cyclic structure of Int A prohibits C–C bond (β bond) flipping. Spin-parallel radicals can undergo slow spin inversion to exclusively generate Int B, which then delivers product 3a (minor path).

**Scheme 5:**
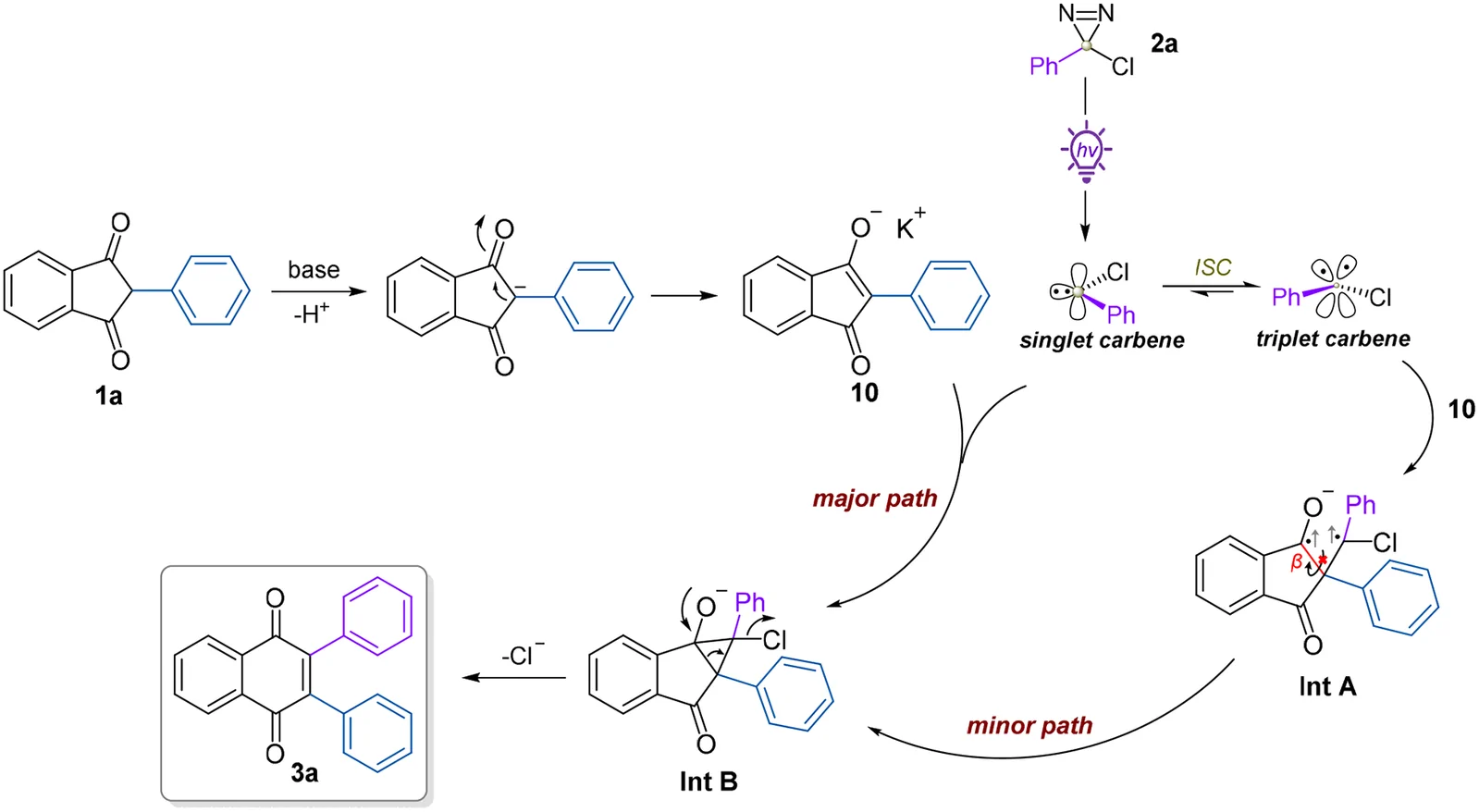
Mechanistic proposal.

## Conclusions

In summary, we have established a violet-light-induced skeletal editing strategy *via* one-carbon ring expansion to synthesize 1,4-naphthoquinones from 2-aryl-1,3-indandiones and chlorodiazirines. This metal-free method features mild reaction conditions, simple operation and high efficiency, providing a straightforward, innovative and green route to 1,4-NQs. Our group is currently exploring other chlorodiazirine-based reaction systems.

## Author contributions

B.-J. W. and L.-B. Z. conceived the project and designed the experiments. B.-J. W., X. Z., and H. L. performed the experiments and analyzed the data. L.-M. Z., W. G. and L.-B. Z. examined the initial manuscript. L.-B. Z. supervised the project.

## Conflicts of interest

There are no conflicts to declare.

## Supplementary Material

SC-017-D6SC04532D-s001

SC-017-D6SC04532D-s002

## Data Availability

CCDC 2572273 contains the supplementary crystallographic data for this paper.^69^ The data supporting this article have been included as part of the supplementary information (SI). Supplementary information is available. See DOI: https://doi.org/10.1039/d6sc04532d.
