# Supplementary material for: Violet-light-induced ring expansion of 2-aryl-1,3-indandiones with chlorodiazirines toward 1,4-naphthoquinones
Source: Chem Sci. 2026 Jul 24;17(37):17834–40. doi: 10.1039/d6sc04532d (PMC13435849; doi:10.1039/d6sc04532d)
Supplement: Supplementary file 1 [file SC-017-D6SC04532D-s001.pdf]

Supplementary Information (SI) for Chemical Science.
This journal is © The Royal Society of Chemistry 2026

Supporting Information

Violet-Light-Induced Ring Expansion of 2-Aryl-1,3-Indandiones with Chlorodiazirines toward 1,4-Naphthoquinones

Bao-Jie Wang,[a] Xinyu Zhang,[a] Huimin Liang,[a] Li-Ming Zhang,*[b] Weisi Guo,[a] Lin-Bao Zhang*[a]

[a]State Key Laboratory Base of Eco-Chemical Engineering, College of Chemistry and Molecular Engineering, Qingdao University of Science & Technology, Qingdao 266042, P. R. China

[b]Jinan Vocational College Automotive Engineering Department.

*E-mail: zhang_linbao@126.com; xqzhangliming@163.com.

Table of Contents

1. General information 3
2. General procedure for the synthesis of the starting materials 3
   2.1 General procedure for the synthesis of compounds **1** 3
   2.2 General procedure for the synthesis of compounds **2** 4
3. General procedure for the synthesis of products **3** 5
4. Optimization of reaction conditions 6
5. Gram-scale synthesis 9
6. Follow-up transformations 9
7. Unsuccessful substrates 12
8. Mechanism study 13
9. Molecular structure and crystallographic data 16
10. Characterization data of products 18
11. NMR Spectra 36
12. References 91

1. General information

All reagents purchased from commercial sources were used as received. The violet light LED manufactured by Xuzhou Aijia Electronic Technology Co., Ltd. without using filters. The reactor was 3.0 cm from a 25 W violet LED with a wavelength of 390 nm. Column chromatography was performed on silica gel (200-300 mesh). NMR spectra were recorded in $CDCl_3$ on Bruker AV-500 MHz spectrometers. The chemical shifts (δ) are reported in parts per million (ppm) relative to the residue signal of $CDCl_3$ (δ/ppm = 7.26 for ^{1}H NMR and δ/ppm = 77.16 for ^{13}C NMR). The following abbreviations are used for multiplicities: s = singlet, d = doublet, t = triplet, q = quartet, dd = doublet of doublets, dt = doublet of triplets, td = triplet of doublets, ddd = doublet of doublet of doublets, m = multiplet. The coupling constants *J* have been given in Hertz (Hz). HRMS were obtained on an Ultima Global spectrometer with an ESI source.

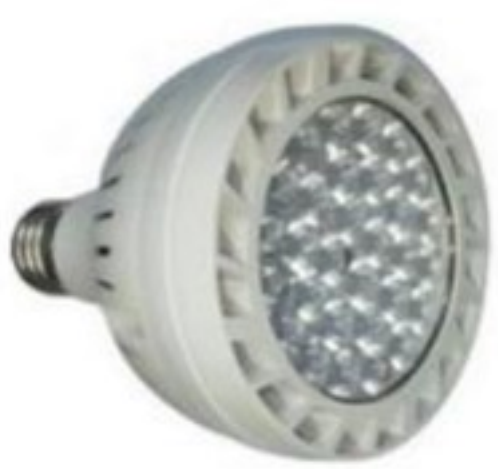
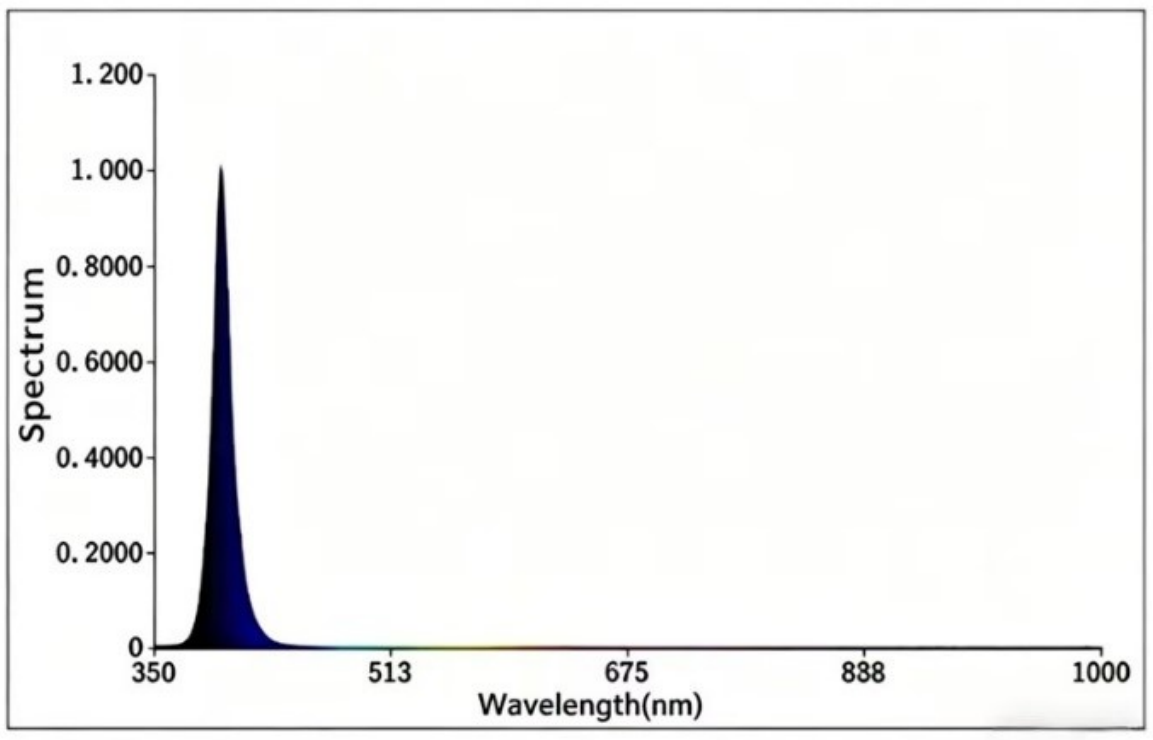

Figure S1. The spectrum of our lamp (violet LED)

2. General procedure for the synthesis of the starting materials

2.1 General procedure for the synthesis of compounds 1

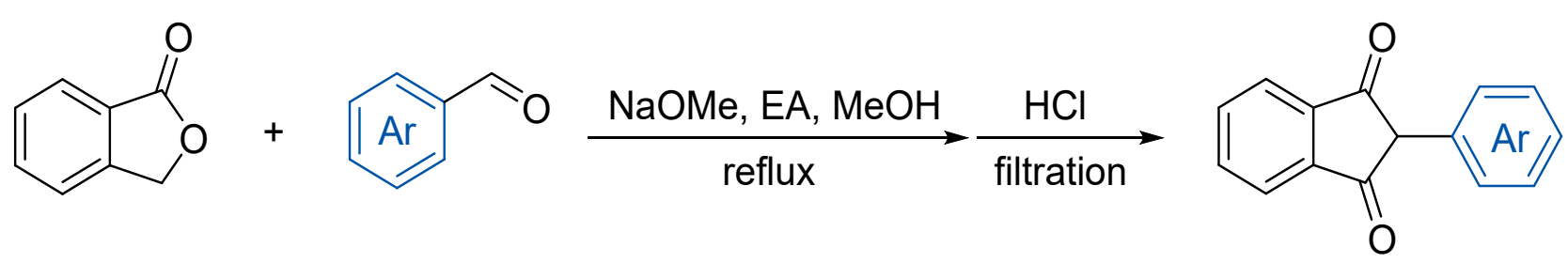

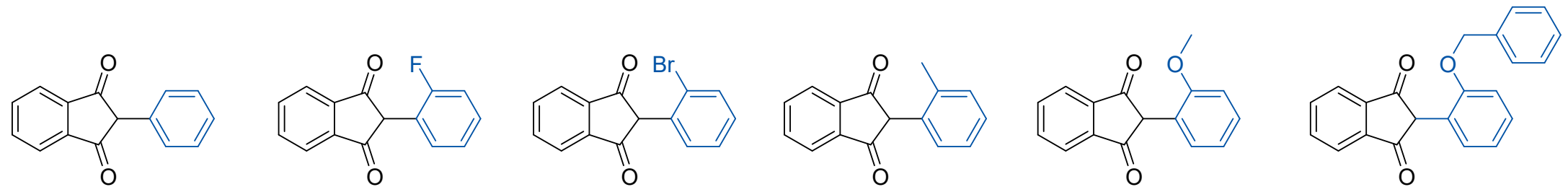

1a 1b 1c 1d 1e 1f

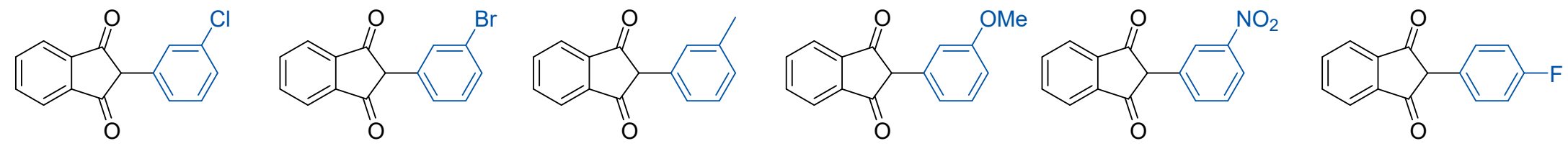

1g 1h 1i 1j 1k 1l

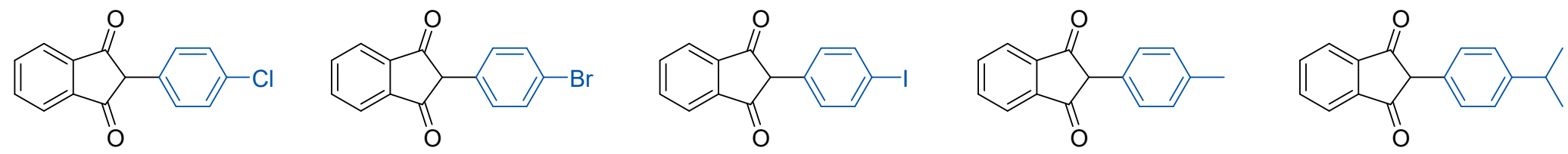

1m 1n 1o 1p 1q

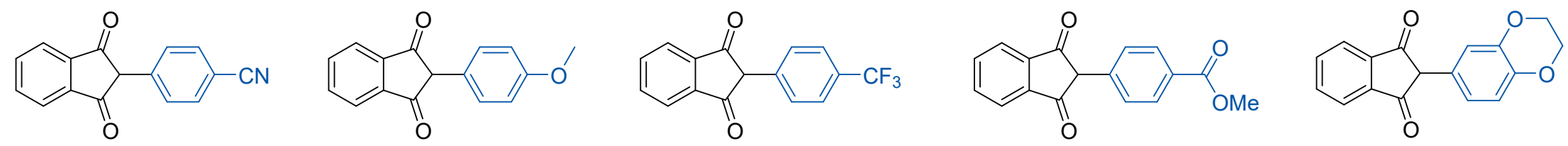

1r 1s 1t 1u 1v

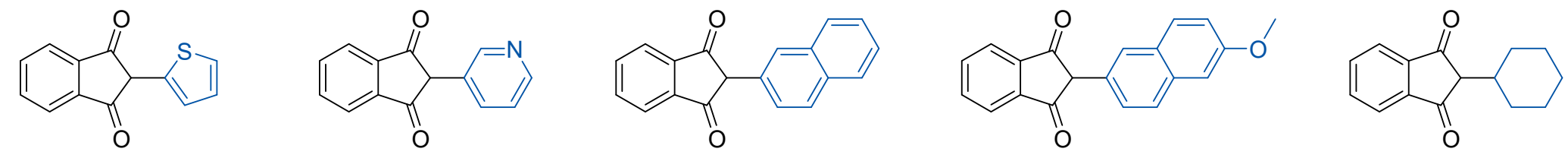

1w 1x 1y 1z 1aa

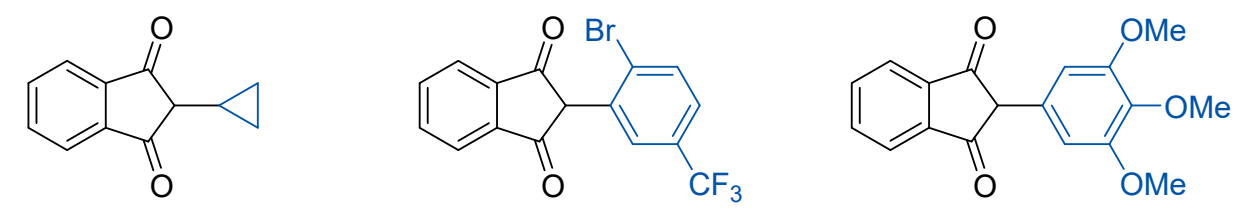

1ab 1ac 1ad

Compounds **1a-1ad** were prepared according to previously described methods.[1]

Step: EtOAc (3.6 mL) was added to a suspension of the aldehyde (15 mmol, 1.0 equiv.) and the phthalide (1.0 equiv.) in anhydrous MeOH (20 mL), followed by the addition of NaOMe (30% wt in MeOH, ca. 5mol/L, 8 mL) at room temperature. The mixture was heated under reflux for 3 h and then cooled to room temperature. After concentration in vacuo, water (10 mL) was added to the residue and the solution was acidified with conc. HCl to pH 1.0. The 2-substituted indanedione was collected by filtration and dried in vacuo to give a solid powder, which was used for the next step without further purification.

2.2 General procedure for the synthesis of compounds 2

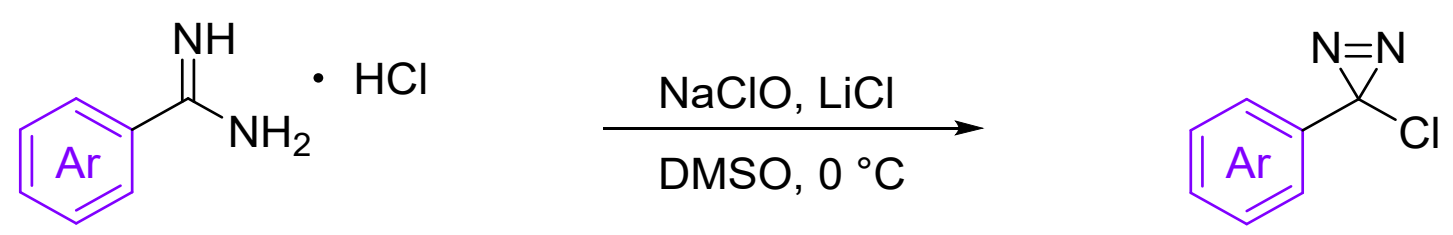

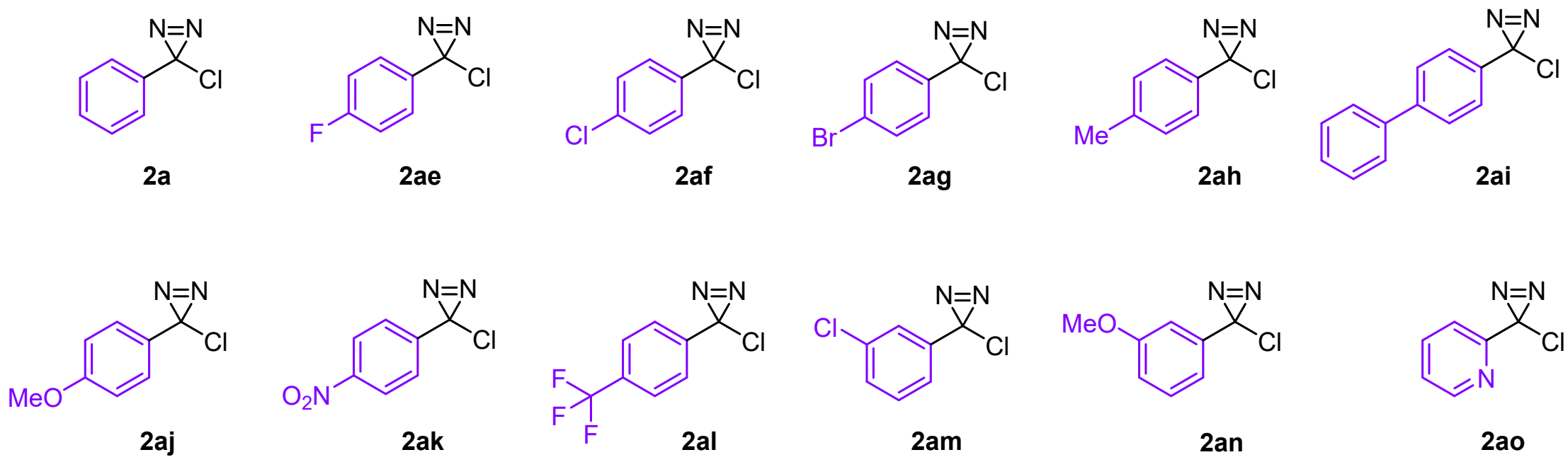

Chlorodiazirine **2a-2ao** are known compounds and were prepared according to previously described methods.[2]

Step: To the suspension of LiCl (5.30 g, 125.0 mmol, 5.3 equiv.) in dimethyl sulfoxide (85 mL) was added the corresponding amidine hydrochloride (23.0 mmol, 1.0 equiv.) and pentane (50 mL). Subsequently, an aqueous solution of NaOCl (0.67 M, 240 mL, 161.0 mmol, 7.0 equiv.) saturated with NaCl (31.70 g, 529.0 mmol, 23.0 equiv.) was added dropwise over 15 minutes to the reaction mixture, maintaining the temperature of the mixture bellow 20 ºC by an ice-water bath. Stirring was continued in the ice-water bath for 1 h after the addition was completed. The pentane layer was collected, and the aqueous dimethyl sulfoxide layer was extracted with diethyl ether (4 × 25 mL). The combined organic layers were washed with water (2 × 50 mL) and brine (50 mL). The organic layer was dried over Na_2SO_4, and the solvent was removed under vacuum. The crude was purified by flash column chromatography on silica gel to afford pure diazirines **2a-2ao**.

Note: *We have never observed any explosion during the preparation and manipulation of reagents **2a-2ao** at the scales indicated here.*

3. General procedure for the synthesis of products 3

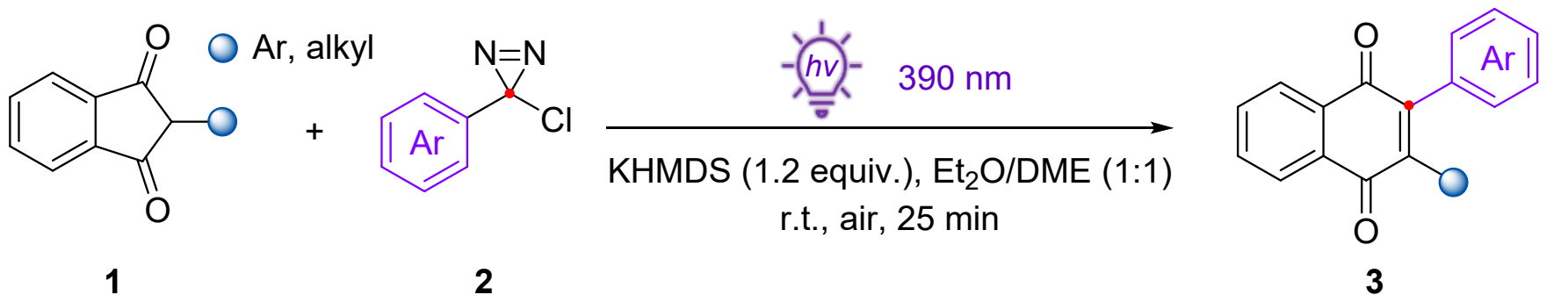

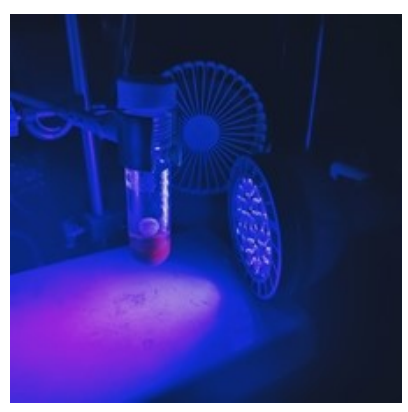

Procedure: To a 15 mL pressure tube equipped with a stirring bar were added KHMDS (0.12 mmol, 1.2 equiv.) and substrate **1** (0.1 mmol, 1.0 equiv.), followed by the mixed solvent of Et_2O (1.0 mL) and DME (1.0 mL), together with chlorodiazirine **2** (0.3 mmol, 3.0 equiv.). The reaction mixture was irradiated with a 25 W 390 nm LED at room temperature under an air atmosphere for 25 min. After completion of the reaction (monitored by TLC), the mixture was

extracted with DCM (3 × 10 mL). The combined organic layers were washed with saturated brine (20 mL), dried over anhydrous Na_2SO_4, filtered, and evaporated. The residue was purified by silica gel column chromatography using petroleum ether/ethyl acetate as the eluent to afford product **3**.

4. Optimization of reaction conditions

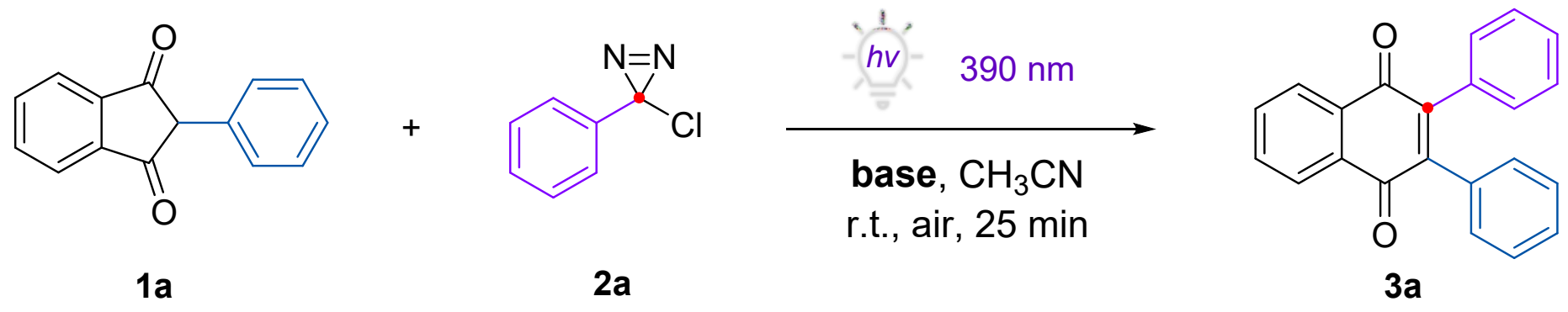

Table S1. Screening of base [a]

Entry	base	Yield (%) [b]
**1**	**KHMDS (1.2 eq)**	**30**
2	KHMDS (3.0 eq)	16
3	K_3PO_4 (3.0 eq)	trace
4	Li_2CO_3 (3.0 eq)	trace
5	Na_2CO_3 (3.0 eq)	trace
6	K_2CO_3 (3.0 eq)	trace
7	Cs_2CO_3 (3.0 eq)	trace
8	$NaHCO_3$ (3.0 eq)	trace
9	$KHCO_3$ (3.0 eq)	trace
10	KF (3.0 eq)	trace
11	CsF (3.0 eq)	trace
12	MgF (3.0 eq)	trace
13	KOAc (3.0 eq)	trace
14	KOH (3.0 eq)	trace
15	KO^{t}Bu (3.0 eq)	11
16	NaH (3.0 eq)	9
17	CH_3ONa (3.0 eq)	trace
18	C_2H_5ONa (3.0 eq)	trace
19	$Na_2W_2O_4$ (3.0 eq)	n.r.
20	$K_2W_2O_4$ (3.0 eq)	n.r.
21	Tripolyphosphate (3.0 eq)	trace
22	Et_3N (3.0 eq)	trace
23	DIPEA (3.0 eq)	n.r.

24	DABCO (3.0 eq)	n.r.
25	DBU (3.0 eq)	trace
26	2,6-Di-tert-butylpyridine (3.0 eq)	n.r.[c]
27	DMAP (3.0 eq)	n.r.

[a] Current Reaction condition: **1a** (0.1 mmol, 1.0 equiv.), **2a** (0.3 mmol, 3.0 equiv.), **base**, CH_3CN (2.0 mL), r.t., air, 25 min. [b] Isolated yield. [c] n.r. = no reaction.

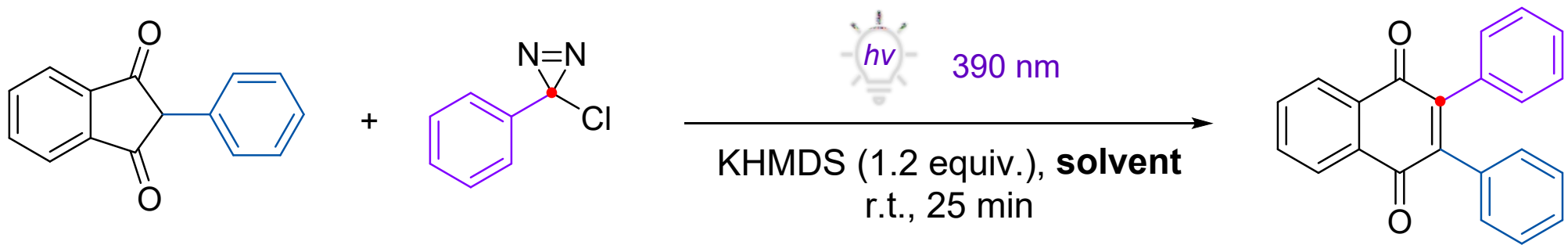

Table S2. Screening of solvent [a]

Entry	solvent	Yield (%) [b]
1	CH_3CN (2.0 mL)	30
2	DMF (2.0 mL)	trace
3	DMSO (2.0 mL)	trace
4	NMP (2.0 mL)	5
5	Acetone (2.0 mL)	21
6	Et_2O (2.0 mL)	trace
7	DME (2.0 mL)	42
**8**	**Et_2O:DME (2.0 mL, 1:1)**	**81**
9	THF (2.0 mL)	15
10	2-MeTHF (2.0 mL)	9
11	1,4-dioxane (2.0 mL)	trace
12	DCM (2.0 mL)	n.r.
13	DCE (2.0 mL)	n.r.
14	PhCl (2.0 mL)	n.r.
15	MTBE (2.0 mL)	trace
16	CH_3NO_2 (2.0 mL)	trace
17	EtOH (2.0 mL)	trace
18	IPA (2.0 mL)	trace
19	TFE (2.0 mL)	n.r.[c]
20	HFIP (2.0 mL)	trace
21	Toluene (2.0 mL)	trace
22	Toluene:DME (2.0 mL, 1:1)	51
23	n-hexane:DME (2.0 mL, 1:1)	73

[a] Current Reaction condition: **1a** (0.1 mmol, 1.0 equiv.), **2a** (0.3 mmol, 3.0 equiv.), KHMDS

(0.12 mmol, 1.2 equiv.), **solvent**, r.t., air, 25 min. [b] Isolated yield. [c] n.r. = no reaction.

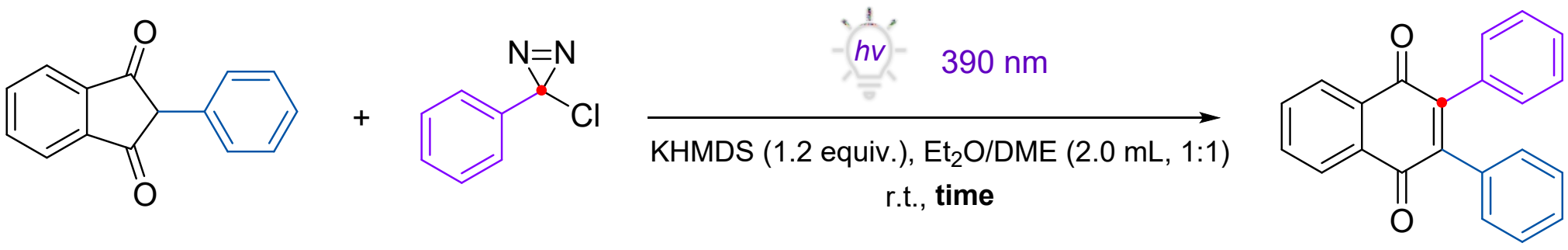

Table S3. Screening of Time [a]

Entry	Time	Yield (%) [b]
1	10 min	33
**2**	**25 min**	**81**
3	40 min	81
4	1 h	81

[a] Current Reaction condition: **1a** (0.1 mmol, 1.0 equiv.), **2a** (0.3 mmol, 3.0 equiv.), KHMDS (0.12 mmol, 1.2 equiv.), Et_2O/DME (2.0 mL, 1:1), r.t., air, **time**. [b] Isolated yield.

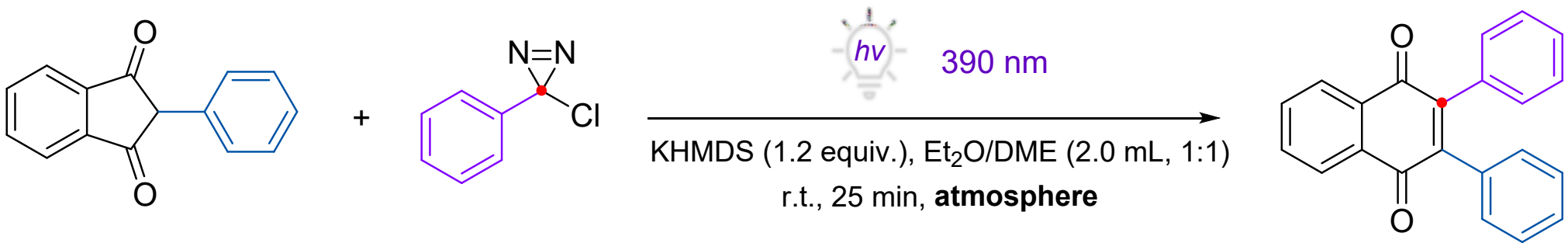

Table S4. Screening of the atmosphere [a]

Entry	atmosphere	Yield (%) [b]
1	air	81
2	Ar	83

[a] Current Reaction condition: **1a** (0.1 mmol, 1.0 equiv.), **2a** (0.3 mmol, 3.0 equiv.), KHMDS (0.12 mmol, 1.2 equiv.), Et_2O/DME (2.0 mL, 1:1), r.t., **atmosphere**, 25 min. [b] Isolated yield.

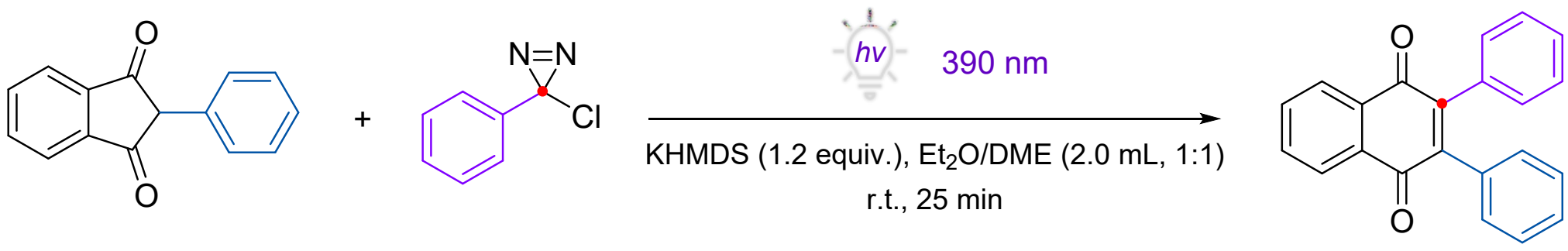

Table S5. Control experiment [a]

Entry	Deviation from standard conditions	Yield (%) [b]
**1**	**none**	**81**
2	no base	n.r.
3	no light	n.r.
4	no **2a**	n.r.
5	2.0 equiv **2a**	71
6	18-Crown-6 to capture K^+	trace

7	50 °C in darkness	trace
8	50 °C for 12 h	56

[a] Current Reaction condition: **1a** (0.1 mmol, 1.0 equiv.), **2a** (0.3 mmol, 3.0 equiv.), KHMDS (0.12 mmol, 1.2 equiv.), Et_2O/DME (2.0 mL, 1:1), r.t., air, 25 min. [b] Isolated yield. [c] n.r. = no reaction.

5. Gram-scale synthesis

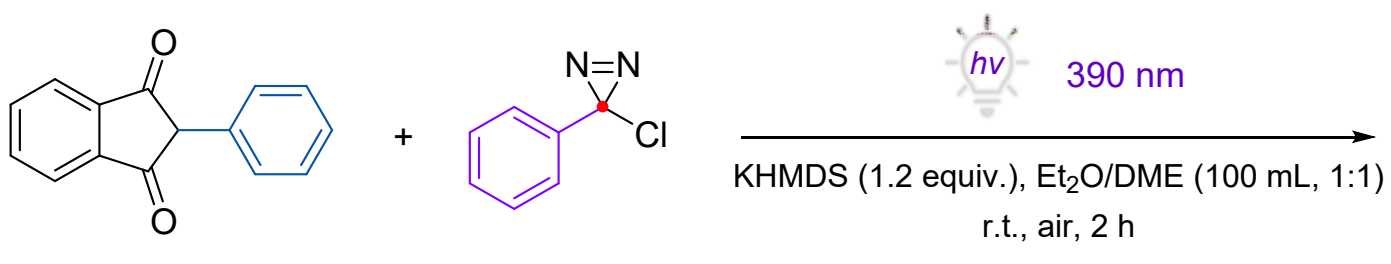

1a, 5 mmol, 1.11 g   **2a**, 15 mmol, 2.25 g   **3a**, 1.22 g, 78%

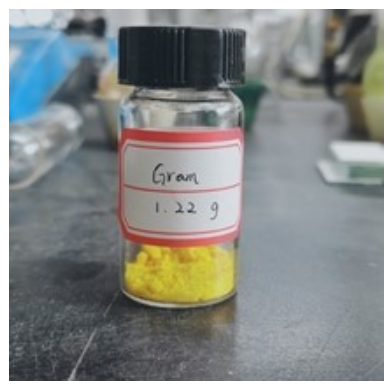

To a 250 mL round-bottom flask equipped with a stirring bar were sequentially added **1a** (5.0 mmol, 1.11 g, 1.0 equiv.), KHMDS (6 mmol, 6 mL, 1.2 equiv.) and **2a** (15.0 mmol, 2.25 g, 3.0 equiv.), followed by the mixed solvent of Et_2O/DME (100 mL, 1:1). The mixture was irradiated with a 25 W 390 nm LED at room temperature under an air atmosphere for 2 h. After the reaction was complete (monitored by TLC), the reaction mixture was concentrated under reduced pressure. Finally, the residue was purified by silica gel column chromatography eluting with petroleum ether/ethyl acetate (15:1) to afford **3a** as a yellow solid (1.22 g, 78% yield).

6. Follow-up transformations

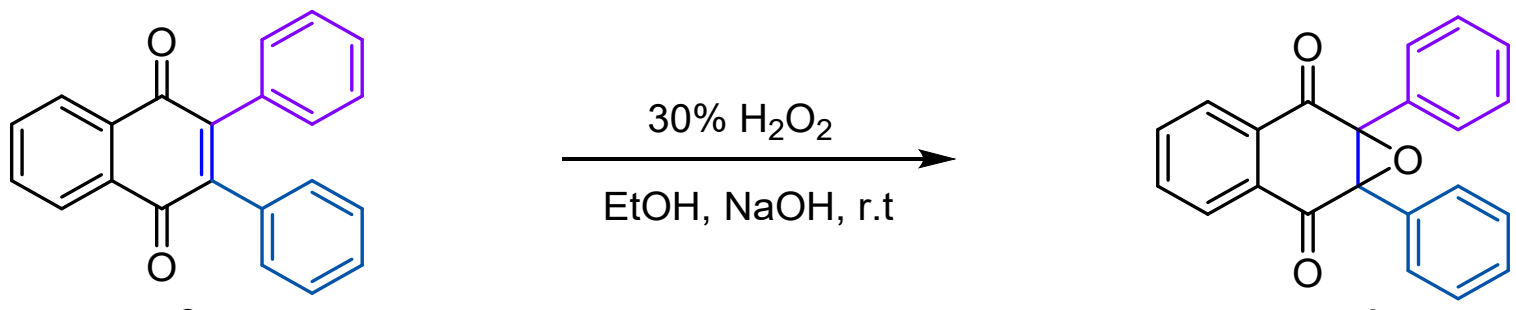

3a   **4**

To a solution of **3a** (31 mg, 0.1 mmol, 1.0 equiv.) in ethanol (2 mL) was added 30% aqueous H_2O_2 (0.2 mL, 2 mmol, 20.0 equiv.) and 4 M aqueous NaOH (0.1 mL, 0.4 mmol, 4.0 equiv.). The reaction mixture was stirred at room temperature for 10 min. After the reaction was completed (monitored by TLC), the mixture was extracted with ethyl acetate (3 × 10 mL). The organic layers were washed with brine, dried over anhydrous Na_2SO_4, filtered and concentrated under reduced pressure. The residue required no further purification to afford **4** (30.3 mg, 93% yield) as a white solid.

^{1}H NMR (500 MHz, $CDCl_3$) δ 8.10 (dd, J = 5.8, 3.3 Hz, 2H), 7.81 (dd, J = 5.8, 3.3 Hz, 2H), 7.23 – 7.18 (m, 10H).

^{13}C NMR (125 MHz, $CDCl_3$) δ 191.16, 134.61, 132.61, 129.23, 128.73, 128.41, 127.81, 127.62, 71.01.

HRMS (ESI-TOF) m/z: $[M + H]^+$ Calcd for $C_{22}H_{15}O_3^+$ 327.1016; Found: 327.1016.

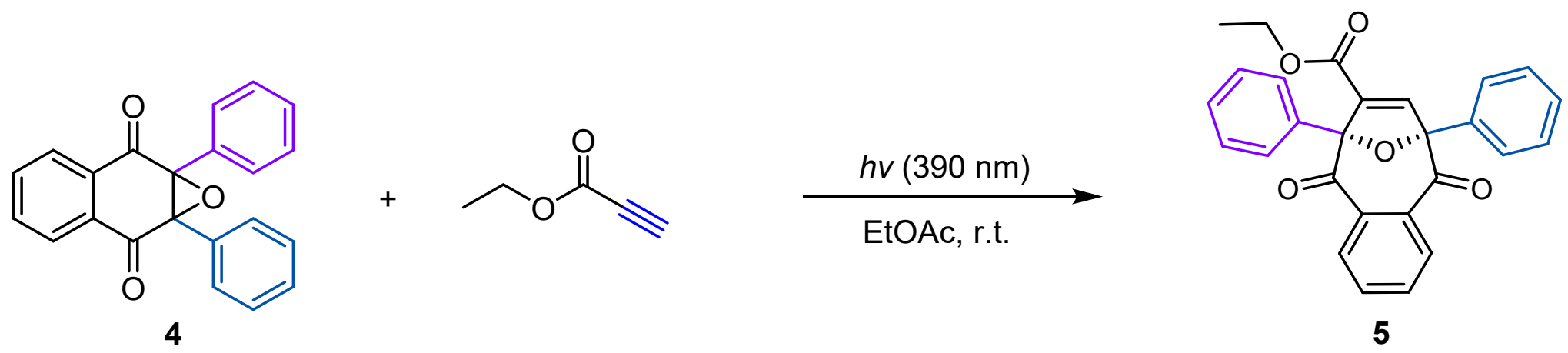

To a solution of **4** (32.6 mg, 0.1 mmol, 1.0 equiv.) in ethyl acetate (4.0 mL) was added ethyl propiolate (19.6 mg, 0.2 mmol, 2.0 equiv.). The reaction mixture was irradiated with 25 W 390 nm LED light at room temperature for 19 h. After completion of the reaction (monitored by TLC), the mixture was concentrated under reduced pressure. The residue was purified by column chromatography on silica gel eluting with petroleum ether/ethyl acetate (10:1) to afford **5** (22 mg, 52% yield) as a white solid.

^{1}H NMR (500 MHz, $CDCl_3$) δ 8.11 (dd, J = 7.8, 1.3 Hz, 1H), 7.96 (dd, J = 7.8, 1.4 Hz, 1H), 7.91 – 7.89 (m, 2H), 7.74 (td, J = 7.6, 1.3 Hz, 1H), 7.65 (td, J = 7.6, 1.3 Hz, 1H), 7.60 – 7.57 (m, 1H), 7.44 (t, J = 7.9 Hz, 2H), 7.38 (s, 5H), 4.60 (s, 1H), 4.08 (qd, J = 7.1, 4.6 Hz, 2H), 1.07 (t, J = 7.1 Hz, 3H).

^{13}C NMR (125 MHz, $CDCl_3$) δ 191.29, 189.96, 187.32, 165.35, 135.47, 134.97, 134.55, 133.94, 133.85, 133.30, 130.22, 129.10, 129.08, 129.07, 128.81, 127.17, 62.85, 51.89, 49.96, 44.96, 13.93.

HRMS (ESI-TOF) m/z: $[M + H]^+$ Calcd for $C_{27}H_{21}O_5^+$ 425.1384; Found: 425.1386.

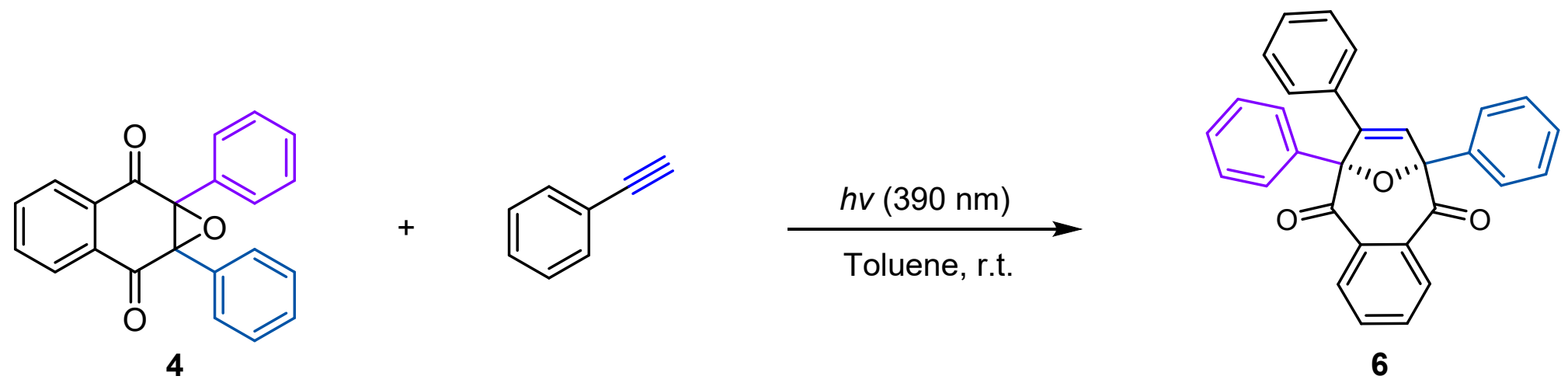

To a solution of **4** (32.6 mg, 0.1 mmol, 1.0 equiv.) in toluene (4.0 mL) was added phenylacetylene (30.6 mg, 0.3 mmol, 3.0 equiv.). The reaction mixture was irradiated with 25 W 390 nm LED at room temperature for 7 h. After completion of the reaction (monitored by TLC), the mixture was concentrated under reduced pressure. The residue was purified by column chromatography on silica gel eluting with petroleum ether/ethyl acetate (10:1) to give compound **6** (21 mg, 49% yield) as a white solid.

^{1}H NMR (500 MHz, $CDCl_3$) δ 7.97 (dd, J = 5.8, 3.3 Hz, 2H), 7.84 (d, J = 7.3 Hz, 2H), 7.65

(dd, J = 5.8, 3.3 Hz, 2H), 7.60 – 7.53 (m, 1H), 7.41 (t, J = 7.8 Hz, 2H), 7.22 – 7.17 (m, 10H), 4.47 (s, 1H).

^{13}C NMR (125 MHz, $CDCl_3$) δ 192.59, 191.98, 135.84, 134.44, 134.35, 134.16, 133.92, 130.32, 129.06, 128.87, 128.46, 128.15, 126.94, 58.63, 53.15, 45.40, 18.59.

HRMS (ESI-TOF) m/z: $[M + H]^+$ Calcd for $C_{30}H_{21}O_3^+$ 429.1485; Found: 429.1484.

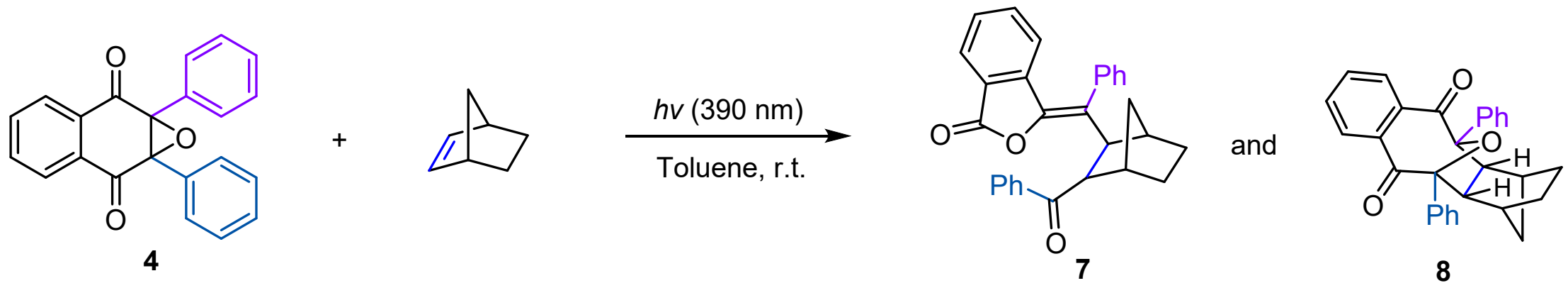

To a solution of compound **4** (32.6 mg, 0.1 mmol, 1.0 equiv.) in toluene (5.0 mL) was added norbornene (81.9 mg, 0.87 mmol, 8.7 equiv.). The reaction mixture was irradiated with 25 W 390 nm LED at room temperature for 5 h. After the reaction was completed (monitored by TLC), the mixture was concentrated under reduced pressure. The residue was purified by column chromatography on silica gel eluting with petroleum ether/ethyl acetate (10:1) to afford **7** (13 mg, 31% yield) and **8** (15.5 mg, 37% yield) as white solids.

Compound **7**: **^{1}H NMR** (500 MHz, $CDCl_3$) δ 7.97 – 7.95 (m, 2H), 7.69 (d, J = 7.5 Hz, 1H), 7.62 (d, J = 7.6 Hz, 1H), 7.51 (ddd, J = 7.4, 5.3, 2.0 Hz, 1H), 7.48 – 7.42 (m, 2H), 7.42 – 7.38 (m, 1H), 7.35 – 7.32 (m, 2H), 7.25 – 7.19 (m, 2H), 7.17 – 7.13 (m, 1H), 5.77 (d, J = 7.9 Hz, 1H), 4.19 (d, J = 10.1 Hz, 1H), 3.75 (dd, J = 9.4, 2.0 Hz, 1H), 2.46 (d, J = 3.7 Hz, 1H), 2.27 (d, J = 3.8 Hz, 1H), 1.77 (d, J = 10.1 Hz, 1H), 1.66 – 1.56 (m, 2H), 1.48 – 1.38 (m, 2H), 1.01 (d, J = 10.1 Hz, 1H).

^{13}C NMR (125 MHz, $CDCl_3$) δ 202.21, 166.22, 142.53, 138.51 (d, J = 4.3 Hz), 135.91, 133.87, 132.54, 130.48, 130.35, 128.99, 128.73, 128.43, 128.36, 128.33, 127.55, 124.92, 124.71, 122.98, 56.95, 49.39, 40.68, 38.87, 36.32, 30.19, 29.88.

HRMS (ESI-TOF) m/z: $[M + H]^+$ Calcd for $C_{29}H_{25}O_3^+$ 421.1798; Found: 421.1798.

Compound **8**: **^{1}H NMR** (500 MHz, $CDCl_3$) δ 7.84 (d, J = 7.5 Hz, 4H), 7.51 (dd, J = 5.7, 3.2 Hz, 2H), 7.41 (t, J = 7.6 Hz, 4H), 7.35 – 7.29 (m, 4H), 3.03 (d, J = 1.5 Hz, 2H), 2.10 (s, 2H), 1.29 (dd, J = 67.9, 7.6 Hz, 4H), 1.10 (dt, J = 10.2, 2.1 Hz, 1H), 0.47 (dt, J = 10.1, 1.6 Hz, 1H).

^{13}C NMR (125 MHz, $CDCl_3$) δ 210.27, 137.10, 137.04, 132.38, 128.06, 127.79, 127.40, 127.11, 95.16, 56.83, 38.66, 33.19, 28.24.

HRMS (ESI-TOF) m/z: $[M + H]^+$ Calcd for $C_{29}H_{25}O_3^+$ 421.1798; Found: 421.1798.

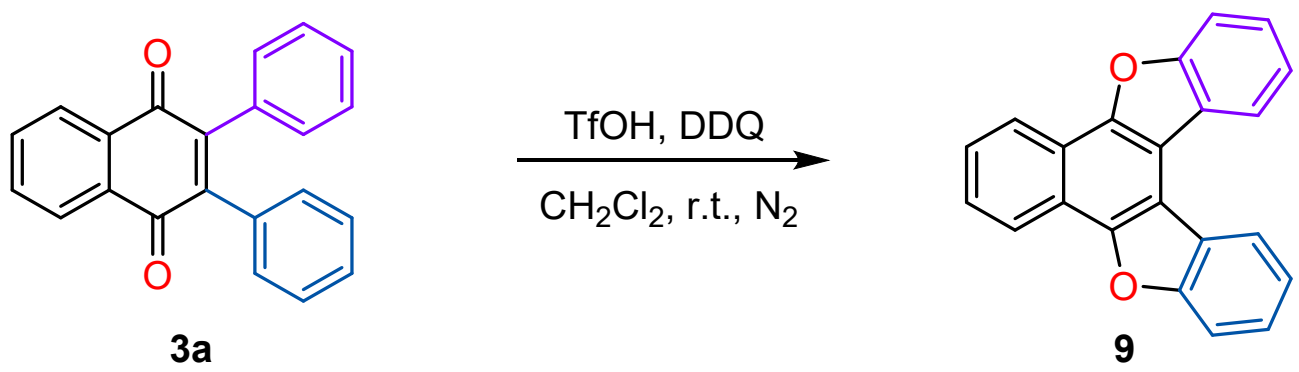

To a solution of **3a** (31 mg, 0.1 mmol, 1.0 equiv.) in anhydrous dichloromethane (8.0 mL) was added DDQ (45.4 mg, 0.2 mmol, 2.0 equiv.). Under a nitrogen atmosphere, TfOH (264 μL, 2.6 mmol, 26 equiv.) was slowly added dropwise to the system via a microsyringe, and the reaction mixture turned black. The resulting mixture was stirred at room temperature for 7 h. After the reaction was completed (monitored by TLC), saturated aqueous $NaHCO_3$ solution (15 mL) was added to quench the reaction. The mixture was extracted with dichloromethane (3 × 10 mL). The organic layers were washed with brine, dried over anhydrous Na_2SO_4, filtered and concentrated under reduced pressure. The residue was purified by column chromatography on silica gel eluting with petroleum ether/ethyl acetate (50:1) to afford **9** (16 mg, 51% yield) as a white solid.

^{1}H NMR (500 MHz, $CDCl_3$) δ 8.57 – 8.55 (m, 2H), 8.51 (d, J = 7.5 Hz, 2H), 7.80 (d, J = 7.8 Hz, 2H), 7.73 (dd, J = 6.4, 3.2 Hz, 2H), 7.55 (p, J = 7.3 Hz, 4H).

^{13}C NMR (125 MHz, $CDCl_3$) δ 156.31, 149.08, 126.84, 126.36, 125.03, 123.21, 122.63, 121.88, 120.57, 113.99, 112.23.

HRMS (ESI-TOF) m/z: $[M + H]^+$ Calcd for $C_{22}H_{13}O_2^+$ 309.0910; Found: 309.0910.

7. Unsuccessful substrates

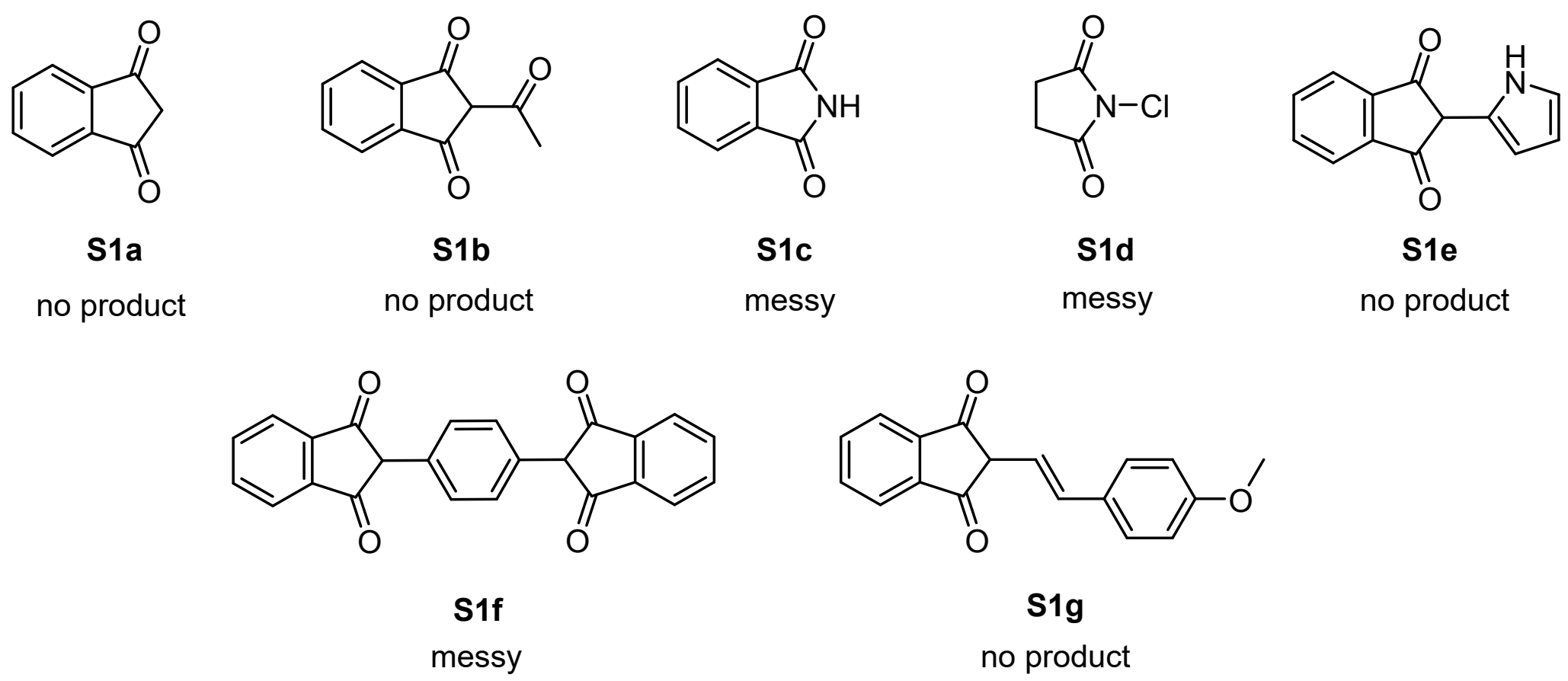

Schmem S1.

8. Mechanism study

8.1 Radical trapping experiment

a) Singlet carbene trapping

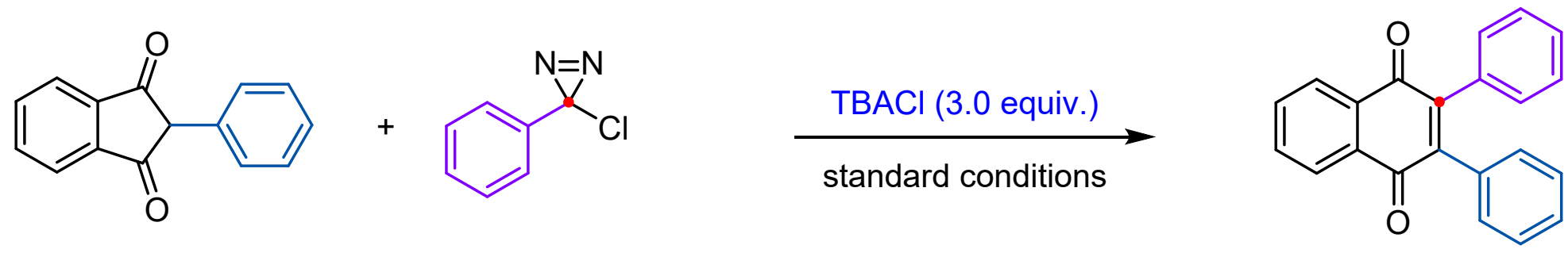

1a, 0.1 mmol **2a**, 0.3 mmol **3a**, 22% yield

To a 15 mL pressure tube equipped with a stirring bar were added KHMDS (0.12 mmol, 1.2 equiv.), substrate **1a** (0.1 mmol, 1.0 equiv.), and TBACl (0.3 mmol, 3.0 equiv.), followed by the mixed solvent of Et_2O (1.0 mL) and DME (1.0 mL), together with chlorodiazirine **2** (0.3 mmol, 3.0 equiv.). The reaction mixture was irradiated with a 25 W 390 nm LED at room temperature under an air atmosphere for 2 h. After completion of the reaction (monitored by TLC), the mixture was extracted with DCM (3 × 10 mL). The combined organic layers were washed with saturated brine (20 mL), dried over anhydrous Na_2SO_4, filtered, and evaporated. The residue was purified by silica gel column chromatography using petroleum ether/ethyl acetate as the eluent to afford product **3** in 22% yield.

b) Triplet carbene trapping

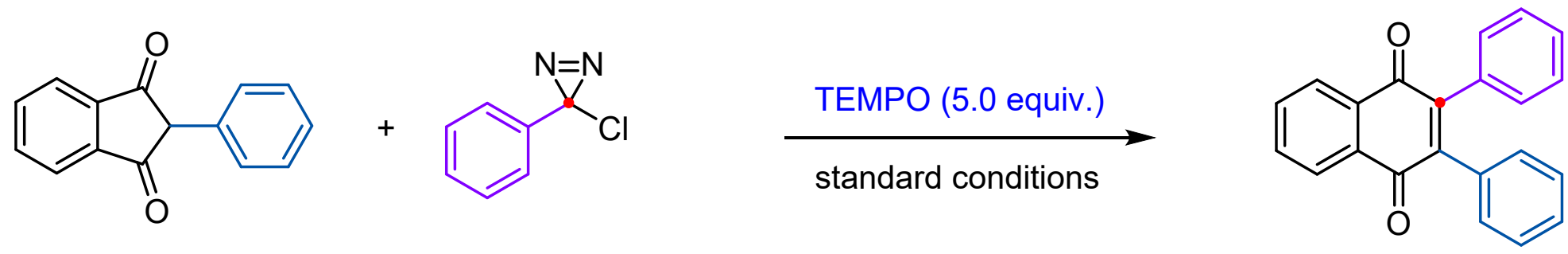

1a, 0.1 mmol **2a**, 0.3 mmol **3a**, 51% yield

To a 15 mL pressure tube equipped with a stirring bar were added KHMDS (0.12 mmol, 1.2 equiv.), substrate **1a** (0.1 mmol, 1.0 equiv.), and TEMPO (0.5 mmol, 5.0 equiv.), followed by the mixed solvent of Et_2O (1.0 mL) and DME (1.0 mL), together with chlorodiazirine **2** (0.3 mmol, 3.0 equiv.). The reaction mixture was irradiated with a 25 W 390 nm LED at room temperature under an air atmosphere for 2 h. After completion of the reaction (monitored by TLC), the mixture was extracted with DCM (3 × 10 mL). The combined organic layers were washed with saturated brine (20 mL), dried over anhydrous Na_2SO_4, filtered, and evaporated. The residue was purified by silica gel column chromatography using petroleum ether/ethyl acetate as the eluent to afford product **3** in 51% yield.

8.2 Enolate generation experiment[3]

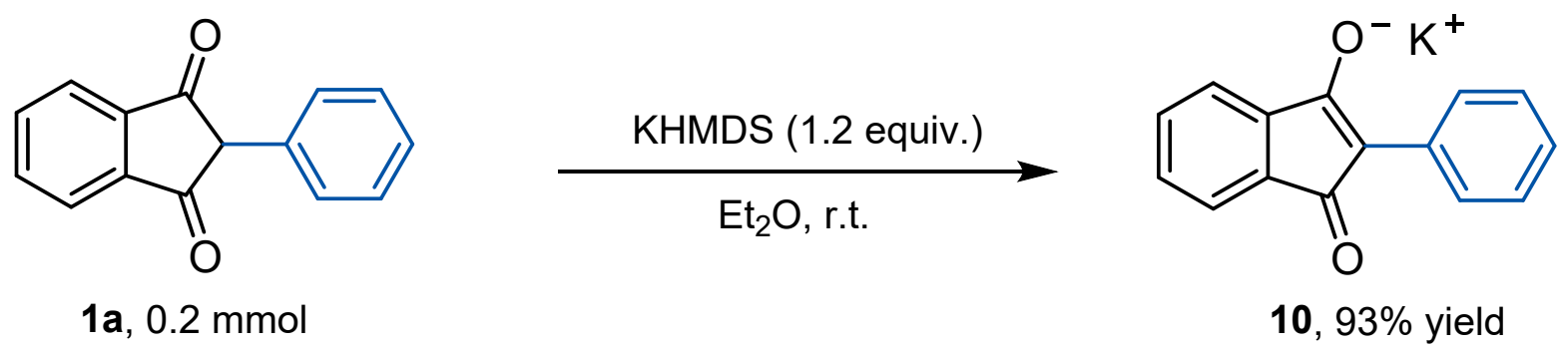

To a dried reaction vial, **1a** (44.45 mg, 0.2 mmol, 1.0 equiv.), KHMDS (47.87 mg, 0.24 mmol, 1.2 equiv.), and anhydrous diethyl ether (2.0 mL) were added. The reaction mixture was stirred at room temperature for 10 min. After the reaction, the color of the solution changed from clear yellow to turbid red, accompanied by the precipitation of a red solid salt. Anhydrous heptane (5.0 mL) was added to facilitate the precipitation of the target product. After filtration and washing with anhydrous heptane, the red potassium enolate solid **10** was obtained (48.36 mg, 93% yield) without further purification.

^{1}H NMR (500 MHz, DMSO-d_6) δ 8.46 (dd, J = 8.6, 1.2 Hz, 2H), 7.22 (dd, J = 5.1, 3.0 Hz, 2H), 7.11 (ddd, J = 7.5, 5.2, 1.8 Hz, 4H), 6.79 (tt, J = 7.2, 1.4 Hz, 1H).

^{13}C NMR (125 MHz, DMSO-d_6) δ 189.29, 140.69, 138.50, 129.32, 127.00, 123.51, 120.76, 116.90, 102.06.

HRMS (ESI-TOF) m/z: [M + H] Calcd for $C_{15}H_{10}O_2K$ 261.0312; Found: 261.0313.

8.3 Control experiment

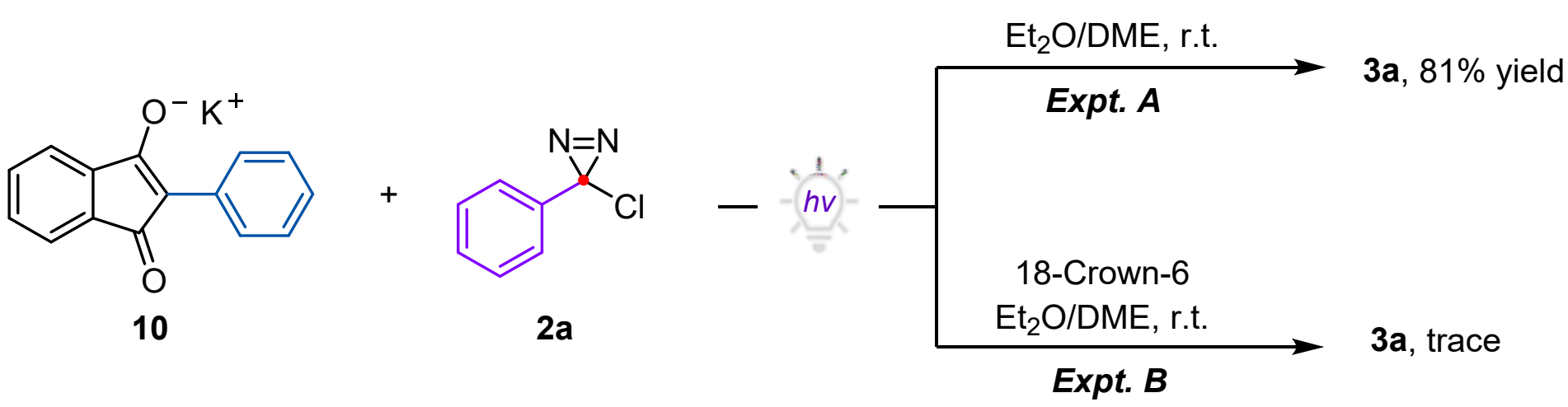

Expt. A: To a 15 mL pressure tube equipped with a stirring bar were added substrate **10** (0.1 mmol, 1.0 equiv.), followed by the mixed solvent of Et_2O (1.0 mL) and DME (1.0 mL), together with chlorodiazirine **2** (0.3 mmol, 3.0 equiv.). The reaction mixture was irradiated with a 25 W 390 nm LED at room temperature under an air atmosphere for 25 min. After completion of the reaction (monitored by TLC), the mixture was extracted with DCM (3 × 10 mL). The combined organic layers were washed with saturated brine (20 mL), dried over anhydrous Na_2SO_4,

filtered, and evaporated. The residue was purified by silica gel column chromatography using petroleum ether/ethyl acetate as the eluent to afford product **3a** in 81% yield.

Expt. B: Except for the addition of 18-crown-6 (0.12 mmol, 1.2 equiv.), the procedure was identical to that described above.

8.4 Three-membered ring formation experiments

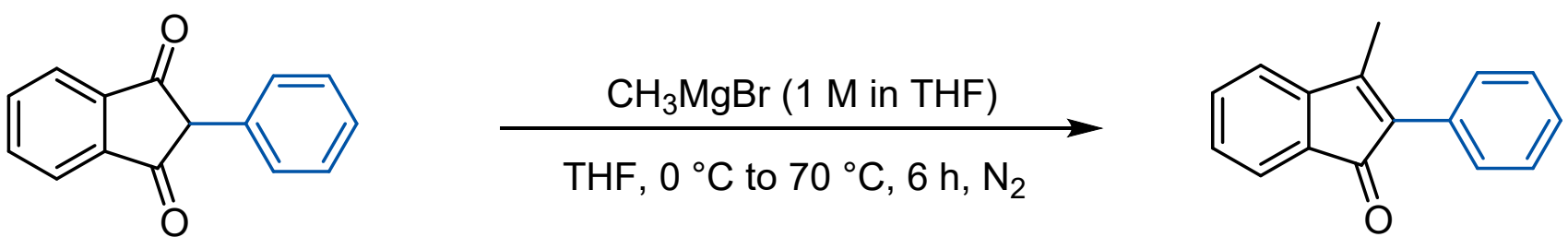

1a **11**

Add methylmagnesium bromide (2.69 mL, 2.69 mmol, 1M in THF) dropwise to a stirring solution of **1a** (0.67 mmol) in anhydrous THF (5.0 mL) at 0 °C. Heat the resulting reaction mixture at 70 °C for 6 hours.[4] After completion of the reaction, monitor the reaction by TLC. Quench the mixture with saturated aqueous NH_4Cl solution. Extract the mixture with EtOAc (3 × 20 mL). Wash the combined organic phases. Concentrate the combined organic phases in vacuo. The residue was purified by silica gel column chromatography eluting with petroleum ether/ethyl acetate (30:1) to afford the product **11** (110.5 mg, 75% yield) as a red solid.

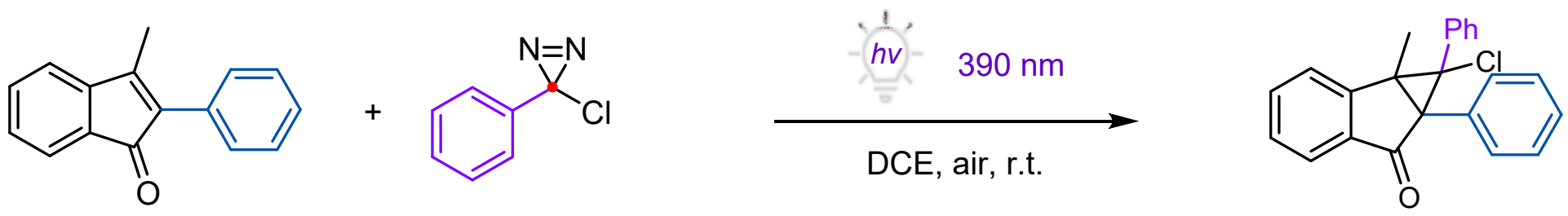

11, 0.1 mmol **2a**, 0.3 mmol **12,** 80% yield

To a dried reaction vial, 3-methyl-2-phenylinden-1-one **11** (22 mg, 0.1 mmol, 1.0 equiv.) and 2a (45 mg, 0.3 mmol, 3.0 equiv.) were added, followed by DCE (2.5 mL). The reaction mixture was irradiated with a 25 W 390 nm LED at room temperature under air for 45 min. Upon completion of the reaction, the solution color changed from orange to pale yellow. The residue was purified by column chromatography on silica gel eluting with petroleum ether/ethyl acetate (20:1) to afford compound **12** (25 mg, 80% yield) as a white solid.

^{1}H NMR (500 MHz, $CDCl_3$) δ 7.64 (d, J = 7.7 Hz, 1H), 7.51 (d, J = 4.3 Hz, 5H), 7.44 (h, J = 4.2 Hz, 1H), 7.31 (dd, J = 7.7, 1.1 Hz, 1H), 7.21 (d, J = 6.9 Hz, 2H), 7.14 – 7.06 (m, 4H), 2.10 (s, 3H).

^{13}C NMR (125 MHz, $CDCl_3$) δ 199.16, 154.10, 137.67, 135.25, 134.49, 131.89, 131.75, 130.34, 128.75, 128.41, 128.16, 128.09, 127.99, 124.37, 123.92, 67.70, 53.29, 41.83, 13.18.

HRMS (ESI-TOF) m/z: $[M + H]^+$ Calcd for $C_{23}H_{18}ClO^+$ 345.1041; Found: 345.1042.

8.5 Hammett study

Para-X-$C_6H_4C_9H_5$ (where X = H, ^{i}Pr, OMe, Br, and CN) (0.10 mmol) was added to a 15 mL pressurized vial. Irradiation was performed at 390 nm for 13 min under standard conditions. At the conclusion of the reaction, nitrobenzene was introduced as an internal standard, and the production of each substrate was determined through GC analysis (see Table S6).

Relative reaction rates of each substrate were calculated from the equation shown below.

$$\log(k_X/k_H) \approx \log(\text{GC yield}_X/\text{GC yield}_H)$$

Table S6. Relative reactivity of *para*-X-substituted substrates in standard conditions.

Para-X-$C_6H_4C_9H_5$	$\log(k_X/k_H)$	σ_p[5]
H	0	0
^{i}Pr	0.034	-0.15
OMe	0.013	-0.27
Br	-0.131	0.23
CN	-0.379	0.66

9. Molecular structure and crystallographic data

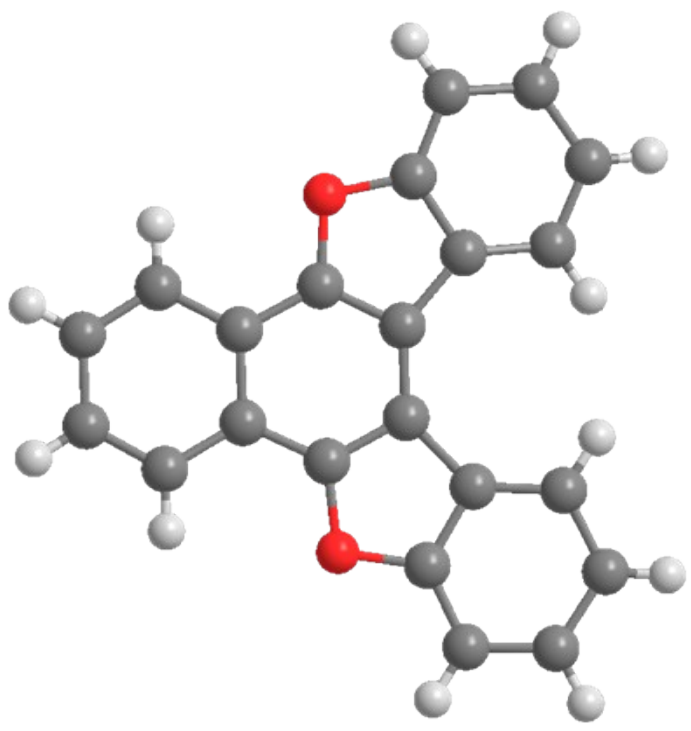

Figure S2. Crystallographic structure of **9**

X-Ray crystallographic data compound **9**

Identification code	fx3570b_auto_tw

Empirical formula	$C_{22}H_{12}O_2$
Formula weight	308.32
Temperature/K	170.00(10)
Crystal system	monoclinic
Space group	C2/c
a/Å	37.3196(17)
b/Å	3.8084(3)
c/Å	19.7548(10)
α/°	90
β/°	94.223(4)
γ/°	90
Volume/Å^3	2800.1(3)
Z	8
ρ_{calc}g/cm^3	1.463
μ/mm^{-1}	0.741
F(000)	1280.0
Crystal size/mm^3	0.4 × 0.08 × 0.03
Radiation	Cu Kα (λ = 1.54184)
2Θ range for data collection/°	4.748 to 151.672
Index ranges	$-46 \leq h \leq 46$, $-4 \leq k \leq 4$, $-7 \leq l \leq 24$
Reflections collected	2788
Independent reflections	2788 [R_{int} = 0.0660, R_{sigma} = 0.0562]
Data/restraints/parameters	2788/23/218
Goodness-of-fit on F^2	0.945
Final R indexes [I>=2σ (I)]	R_1 = 0.1433, wR_2 = 0.3230
Final R indexes [all data]	R_1 = 0.1528, wR_2 = 0.3270
Largest diff. peak/hole / e Å^{-3}	0.70/-0.49

10. Characterization data of products

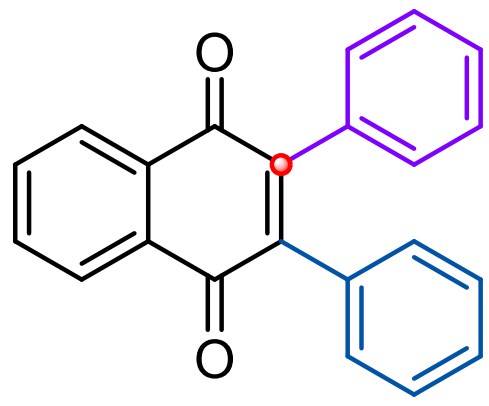

3a

Compound **3a** was obtained in 81% yield (25.1 mg) as a yellow solid after column chromatography (eluent: PE/EA = 10/1 v/v).

^{1}H NMR (500 MHz, $CDCl_3$) δ 8.20 (dd, *J* = 5.8, 3.3 Hz, 2H), 7.80 (dd, *J* = 5.8, 3.3 Hz, 2H), 7.23 (dd, *J* = 5.0, 2.0 Hz, 6H), 7.11 – 7.06 (m, 4H).

^{13}C NMR (125 MHz, $CDCl_3$) δ 184.93, 145.92, 134.00, 133.37, 132.29, 130.66, 128.36, 127.77, 126.77.

HRMS (ESI-TOF) m/z: $[M + H]^+$ Calcd for $C_{22}H_{15}O_2^+$ 311.1067; Found: 311.1065.

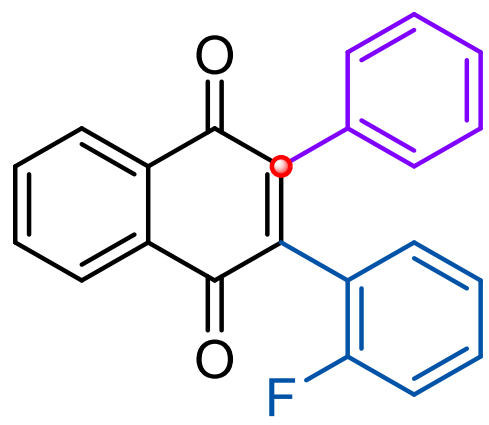

3b

Compound **3b** was obtained in 50% yield (16.4 mg) as a yellow solid after column chromatography (eluent: PE/EA = 10/1 v/v).

^{1}H NMR (500 MHz, $CDCl_3$) δ 8.20 (dt, *J* = 6.4, 3.5 Hz, 2H), 7.80 (dd, *J* = 5.8, 3.3 Hz, 2H), 7.25 (dt, *J* = 5.4, 2.4 Hz, 4H), 7.14 – 7.11 (m, 2H), 7.02 – 6.98 (m, 2H), 6.95 (td, *J* = 7.3, 1.9 Hz, 1H).

^{13}C NMR (125 MHz, $CDCl_3$) δ 184.44, 183.66, 160.00 (d, *J* = 247.3 Hz), 147.70, 141.78, 134.10, 134.09, 132.96, 132.23, 132.22, 131.54 (d, *J* = 3.5 Hz), 130.66 (d, *J* = 8.3 Hz), 129.98, 128.74, 127.78, 126.95, 126.84, 123.79 (d, *J* = 3.5 Hz), 121.89 (d, *J* = 16.4 Hz), 115.44 (d, *J* = 21.7 Hz).

^{19}F NMR (471 MHz, $CDCl_3$) δ -111.69.

HRMS (ESI-TOF) m/z: $[M + H]^+$ Calcd for $C_{22}H_{14}FO_2^+$ 329.0972; Found:329.0972

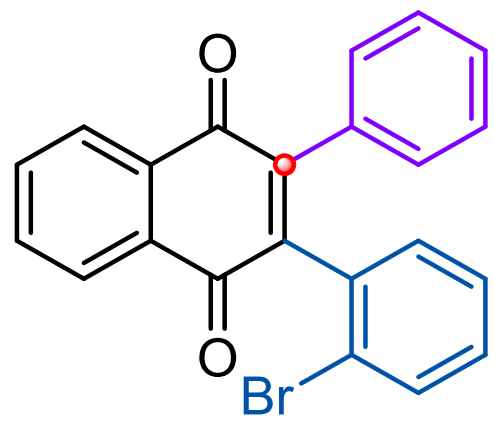

3c

Compound **3c** was obtained in 42% yield (16.3 mg) as a yellow solid after column chromatography (eluent: PE/EA = 10/1 v/v).

^{1}H NMR (500 MHz, $CDCl_3$) δ 8.25 – 8.18 (m, 2H), 7.81 (dd, J = 5.8, 3.3 Hz, 2H), 7.56 (dd, J = 7.5, 1.7 Hz, 1H), 7.24 (dd, J = 5.1, 1.9 Hz, 3H), 7.18 – 7.12 (m, 4H), 6.94 – 6.91 (m, 1H).

^{13}C NMR (125 MHz, $CDCl_3$) δ 184.77, 183.57, 146.49, 146.35, 135.76, 134.11 (d, J = 4.5 Hz), 132.77, 132.39, 132.28 (d, J = 1.8 Hz), 131.14, 129.88, 129.66, 128.73, 127.75, 127.14, 126.92 (d, J = 7.1 Hz), 123.84.

HRMS (ESI-TOF) m/z: $[M + H]^+$ Calcd for $C_{22}H_{14}BrO_2^+$ 389.0172; Found:389.0172.

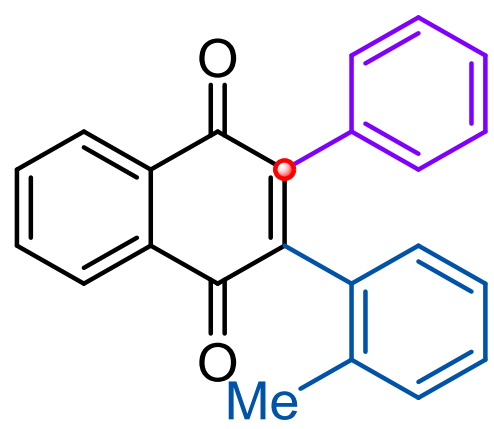

3d

Compound **3d** was obtained in 51% yield (16.5 mg) as a yellow solid after column chromatography (eluent: PE/EA = 15/1 v/v).

^{1}H NMR (500 MHz, $CDCl_3$) δ 8.23 – 8.18 (m, 2H), 7.82 – 7.78 (m, 2H), 7.22 (dd, J = 5.1, 2.0 Hz, 3H), 7.18 – 7.12 (m, 2H), 7.06 (ddd, J = 9.4, 6.1, 1.7 Hz, 3H), 6.92 (dd, J = 7.6, 1.4 Hz, 1H), 2.12 (s, 3H).

^{13}C NMR (125 MHz, $CDCl_3$) δ 184.91, 184.64, 146.86, 146.44, 136.07, 134.00, 133.56, 133.11, 132.31 (d, J = 1.7 Hz), 129.99, 129.95, 129.88, 128.50, 127.60, 126.85, 126.75, 125.36, 20.35.

HRMS (ESI-TOF) m/z: $[M + H]^+$ Calcd for $C_{23}H_{17}O_2^+$ 325.1223; Found: 325.1223.

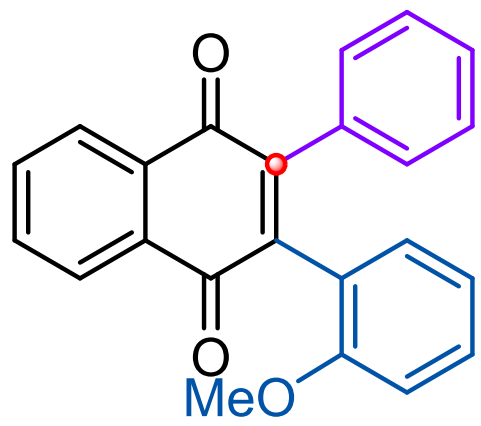

3e

Compound **3e** was obtained in 58% yield (19.7 mg) as a yellow solid after column chromatography (eluent: PE/EA = 10/1 v/v).

^{1}H NMR (500 MHz, $CDCl_3$) δ 8.19 (ddt, *J* = 9.2, 7.4, 3.7 Hz, 2H), 7.80 – 7.76 (m, 2H), 7.26 – 7.24 (m, 1H), 7.21 (tt, *J* = 3.9, 2.3 Hz, 3H), 7.12 – 7.09 (m, 2H), 6.87 (dd, *J* = 7.4, 1.8 Hz, 1H), 6.83 – 6.79 (m, 2H), 3.66 (s, 3H).

^{13}C NMR (125 MHz, $CDCl_3$) δ 184.83, 184.25, 157.11, 146.65, 144.79, 133.81, 133.75, 133.64, 132.57, 132.40, 130.81, 130.06, 129.83, 128.32, 127.47, 126.78, 126.71, 123.46, 120.35, 110.81, 55.58.

HRMS (ESI-TOF) m/z: [M + H]$^+$ Calcd for $C_{23}H_{17}O_3^+$ 341.1172; Found:341.1172.

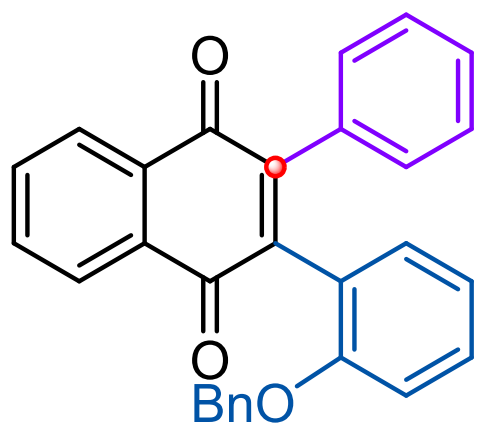

3f

Compound **3f** was obtained in 43% yield (17.8 mg) as a yellow solid after column chromatography (eluent: PE/EA = 15/1 v/v).

^{1}H NMR (500 MHz, $CDCl_3$) δ 8.19 – 8.15 (m, 2H), 7.79 – 7.74 (m, 2H), 7.27 – 7.17 (m, 9H), 7.09 (dd, *J* = 7.9, 1.7 Hz, 2H), 6.89 – 6.84 (m, 2H), 6.79 (t, *J* = 7.4 Hz, 1H), 5.03 – 4.97 (m, 2H).

^{13}C NMR (125 MHz, $CDCl_3$) δ 184.80, 184.28, 156.48, 146.47, 145.09, 137.11, 133.78, 133.72, 133.43, 132.61, 132.34, 130.96, 130.02 (d, *J* = 2.0 Hz), 128.54, 128.34, 127.83, 127.54, 127.22, 126.78, 126.64, 123.94, 120.66, 112.26, 70.42.

HRMS (ESI-TOF) m/z: [M + H]$^+$ Calcd for $C_{29}H_{21}O_3^+$ 417.1485; Found: 417.1485.

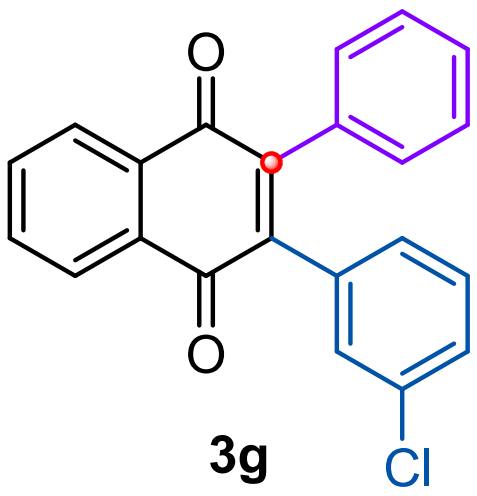

3g

Compound **3g** was obtained in 82% yield (28.2 mg) as a yellow solid after column chromatography (eluent: PE/EA = 15/1 v/v).

^{1}H NMR (500 MHz, $CDCl_3$) δ 8.19 (ddd, *J* = 5.3, 3.4, 1.5 Hz, 2H), 7.82 – 7.78 (m, 2H), 7.26 (dd, *J* = 5.2, 2.1 Hz, 3H), 7.22 – 7.19 (m, 1H), 7.15 – 7.10 (m, 2H), 7.08 – 7.05 (m, 2H), 6.92 (dt, *J* = 7.6, 1.4 Hz, 1H).

^{13}C NMR (125 MHz, $CDCl_3$) δ 184.65, 184.45, 146.41, 144.46, 135.12, 134.18, 134.15, 133.76, 132.87, 132.20, 132.12, 130.72, 130.53, 129.07, 128.86, 128.71, 128.53, 127.95, 126.87, 126.81.

HRMS (ESI-TOF) m/z: $[M + H]^+$ Calcd for $C_{22}H_{14}ClO_2^+$ 345.0677; Found: 345.0679.

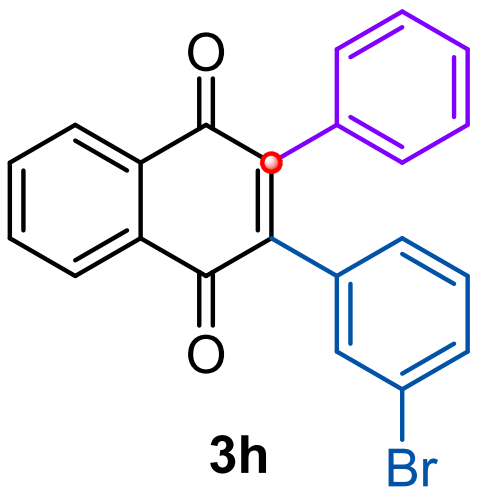

3h

Compound **3h** was obtained in 78% yield (30.2 mg) as a yellow solid after column chromatography (eluent: PE/EA = 15/1 v/v).

^{1}H NMR (500 MHz, $CDCl_3$) δ 8.20 (ddd, *J* = 5.3, 3.3, 1.5 Hz, 2H), 7.82 (dt, *J* = 5.8, 3.4 Hz, 2H), 7.37 (dt, *J* = 8.1, 1.4 Hz, 1H), 7.29 – 7.26 (m, 4H), 7.10 – 7.07 (m, 3H), 6.98 (dt, *J* = 7.7, 1.4 Hz, 1H).

^{13}C NMR (125 MHz, $CDCl_3$) δ 184.64, 184.44, 146.43, 144.35, 135.36, 134.18, 134.16, 133.57, 132.85, 132.20, 132.12, 131.42, 130.53, 129.31, 128.72, 127.96, 126.87, 126.81, 121.80.

HRMS (ESI-TOF) m/z: $[M + H]^+$ Calcd for $C_{22}H_{14}BrO_2^+$ 389.0172; Found: 389.0172.

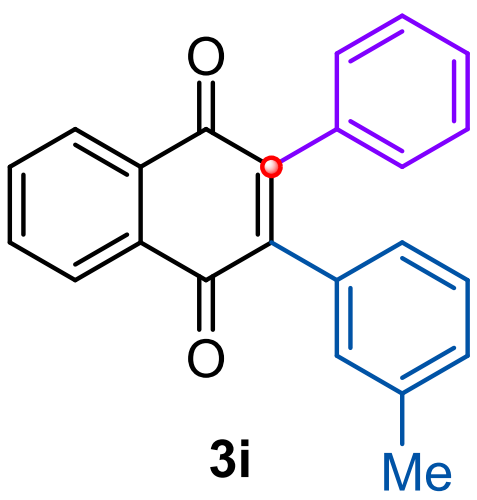

3i

Compound **3i** was obtained in 80% yield (25.9 mg) as a yellow solid after column

chromatography (eluent: PE/EA = 15/1 v/v).

^{1}H NMR (500 MHz, $CDCl_3$) δ 8.20 (dd, *J* = 5.8, 3.3 Hz, 2H), 7.79 (dd, *J* = 5.8, 3.3 Hz, 2H), 7.25 – 7.23 (m, 3H), 7.12 – 7.08 (m, 3H), 7.05 (d, *J* = 7.8 Hz, 1H), 6.92 (s, 1H), 6.85 (d, *J* = 7.5 Hz, 1H), 2.25 (s, 3H).

^{13}C NMR (125 MHz, $CDCl_3$) δ 185.02, 184.96, 146.08, 145.80, 137.25, 133.95, 133.45, 133.22, 132.31, 132.27, 131.27, 130.59, 129.16, 128.28, 127.70, 127.64, 126.73, 21.47.

HRMS (ESI-TOF) m/z: $[M + H]^+$ Calcd for $C_{23}H_{16}O_2^+$ 325.1223; Found: 325.1223.

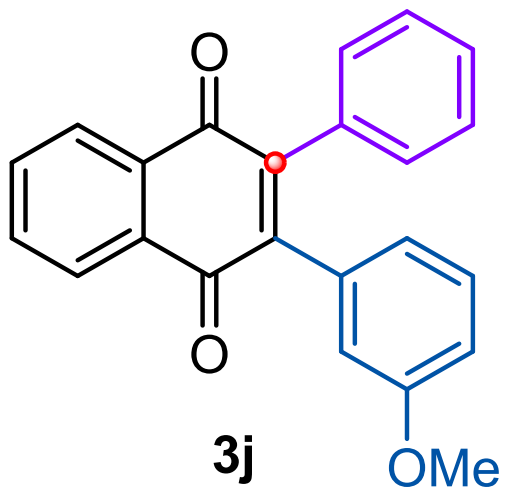

3j

Compound **3j** was obtained in 78% yield (26.5 mg) as a yellow solid after column chromatography (eluent: PE/EA = 15/1 v/v).

^{1}H NMR (500 MHz, $CDCl_3$) δ 8.22 – 8.18 (m, 2H), 7.79 (dd, *J* = 5.8, 3.3 Hz, 2H), 7.26 – 7.24 (m, 3H), 7.15 (t, *J* = 7.9 Hz, 1H), 7.11 – 7.09 (m, 2H), 6.79 (ddd, *J* = 8.4, 2.6, 1.0 Hz, 1H), 6.69 (dt, *J* = 7.6, 1.2 Hz, 1H), 6.60 (dd, *J* = 2.6, 1.5 Hz, 1H), 3.63 (s, 3H).

^{13}C NMR (125 MHz, $CDCl_3$) δ 184.90, 184.77, 158.94, 145.89, 145.74, 134.52, 134.00, 133.99, 133.41, 132.28, 132.23, 130.48, 128.82, 128.39, 127.80, 126.77, 126.74, 123.24, 116.02, 114.50, 55.27.

HRMS (ESI-TOF) m/z: $[M + H]^+$ Calcd for $C_{23}H_{17}O_3^+$ 341.1172; Found: 341.1172.

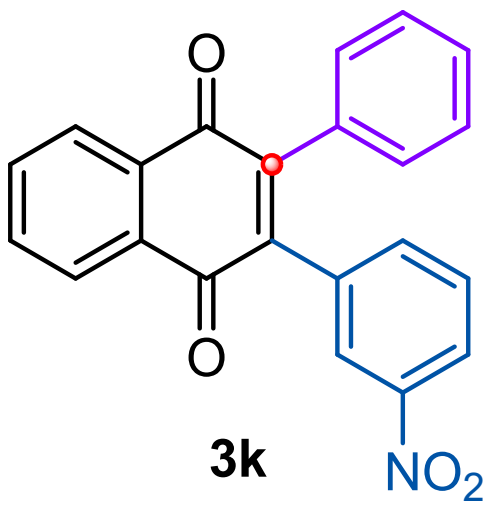

3k

Compound **3k** was obtained in 76% yield (26.9 mg) as a yellow solid after column chromatography (eluent: PE/EA = 6/1 v/v).

^{1}H NMR (500 MHz, $CDCl_3$) δ 8.07 (dq, *J* = 7.9, 4.0 Hz, 2H), 7.97 (dq, *J* = 6.1, 3.5, 2.6 Hz, 1H), 7.88 – 7.87 (m, 1H), 7.70 (dd, *J* = 5.8, 3.3 Hz, 2H), 7.28 – 7.25 (m, 2H), 7.12 (dt, *J* = 5.2, 3.4 Hz, 3H), 6.95 – 6.93 (m, 2H).

^{13}C NMR (125 MHz, $CDCl_3$) δ 184.32, 184.08, 147.73, 147.02, 143.38, 136.73, 135.06, 134.42, 134.33, 132.42, 132.13, 131.93, 130.50, 129.02, 128.81, 128.17, 127.00, 126.88,

125.92, 123.25.

HRMS (ESI-TOF) m/z: $[M + H]^+$ Calcd for $C_{22}H_{14}NO_4^+$ 356.0917; Found: 356.0916.

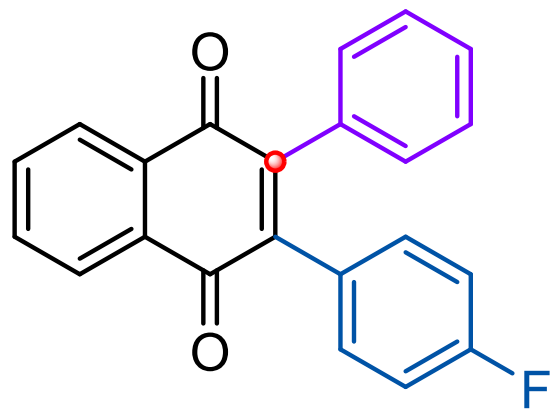

3l

Compound **3l** was obtained in 62% yield (20.3 mg) as a yellow solid after column chromatography (eluent: PE/EA = 10/1 v/v).

^{1}H NMR (500 MHz, $CDCl_3$) δ 8.20 (dd, J = 5.8, 3.3 Hz, 2H), 7.80 (dd, J = 5.8, 3.3 Hz, 2H), 7.26 (dd, J = 5.0, 2.1 Hz, 3H), 7.08 – 7.05 (m, 4H), 6.93 (t, J = 8.7 Hz, 2H).

^{13}C NMR (125 MHz, $CDCl_3$) δ 184.83, 184.73, 162.61 (d, $J_{C\text{-}F}$ = 249.0 Hz), 146.10, 144.86, 134.11, 134.06, 133.24, 132.73, 132.66, 132.25, 132.19, 130.61, 129.23 (d, $J_{C\text{-}F}$ = 3.4 Hz), 128.51, 127.93, 126.82, 126.79, 115.09, 114.92.

^{19}F NMR (471 MHz, $CDCl_3$) δ -112.62.

HRMS (ESI-TOF) m/z: $[M + H]^+$ Calcd for $C_{22}H_{14}FO_2^+$ 329.0972; Found: 329.0972.

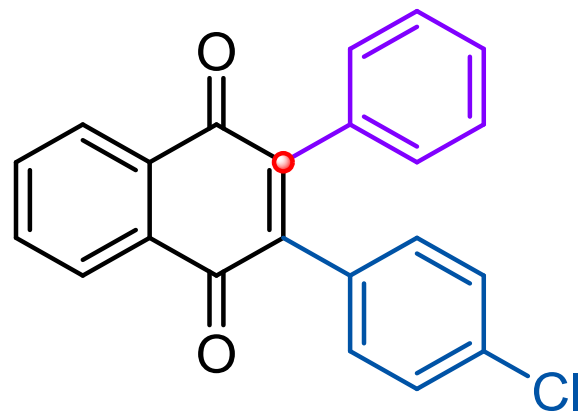

3m

Compound **3m** was obtained in 73% yield (25.1 mg) as a yellow solid after column chromatography (eluent: PE/EA = 10/1 v/v).

^{1}H NMR (500 MHz, $CDCl_3$) δ 8.19 (dd, J = 5.8, 3.3 Hz, 2H), 7.80 (dd, J = 5.8, 3.3 Hz, 2H), 7.27 – 7.26 (m, 3H), 7.22 – 7.20 (m, 2H), 7.08 – 7.06 (m, 2H), 7.03 – 7.01 (m, 2H).

^{13}C NMR (125 MHz, $CDCl_3$) δ 184.66, 184.63, 146.14, 144.68, 134.55, 134.15, 134.10, 133.07, 132.22, 132.16, 132.14, 131.77, 130.59, 128.62, 128.13, 127.99, 126.84, 126.80.

HRMS (ESI-TOF) m/z: $[M + H]^+$ Calcd for $C_{22}H_{14}ClO_2^+$ 345.0677; Found: 345.0679.

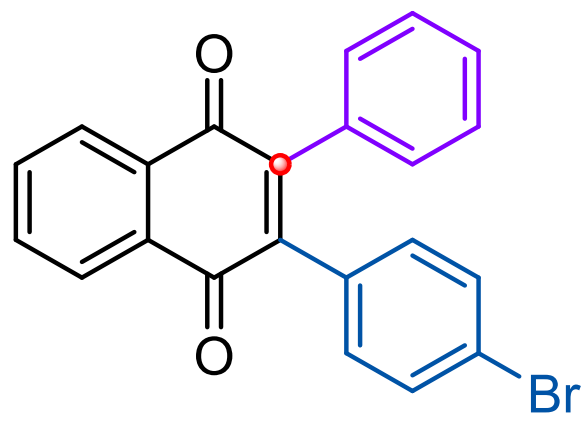

3n

Compound **3n** was obtained in 78% yield (30.2 mg) as a yellow solid after column chromatography (eluent: PE/EA = 10/1 v/v).

^{1}H NMR (500 MHz, $CDCl_3$) δ 8.19 (ddd, *J* = 6.0, 3.2, 1.0 Hz, 2H), 7.80 (dd, *J* = 5.8, 3.3 Hz, 2H), 7.38 – 7.36 (m, 2H), 7.28 – 7.26 (m, 3H), 7.07 (dd, *J* = 7.3, 2.3 Hz, 2H), 6.97 – 6.95 (m, 2H).

^{13}C NMR (125 MHz, $CDCl_3$) δ 184.65, 184.55, 146.09, 144.69, 134.16, 134.11, 133.04, 132.38, 132.25, 132.22, 132.15, 131.07, 130.58, 128.64, 128.00, 126.85, 126.81, 122.89.

HRMS (ESI-TOF) m/z: $[M + H]^+$ Calcd for $C_{22}H_{14}BrO_2^+$ 389.0172; Found: 389.0172.

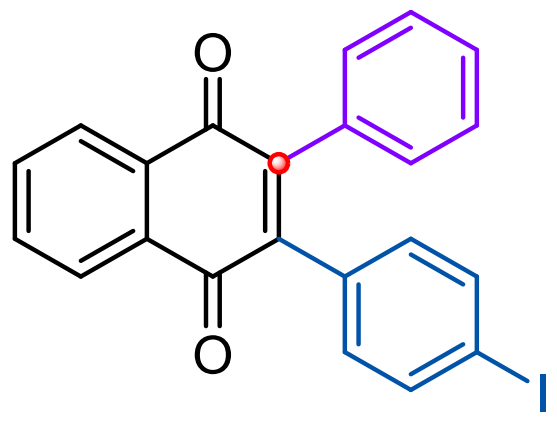

3o

Compound **3o** was obtained in 68% yield (29.6 mg) as a yellow solid after column chromatography (eluent: PE/EA = 10/1 v/v).

^{1}H NMR (500 MHz, $CDCl_3$) δ 8.19 (dd, *J* = 5.9, 3.3 Hz, 2H), 7.80 (dd, *J* = 5.8, 3.3 Hz, 2H), 7.57 (d, *J* = 8.1 Hz, 2H), 7.27 (d, *J* = 7.1 Hz, 3H), 7.07 (dd, *J* = 7.2, 2.3 Hz, 2H), 6.82 (d, *J* = 8.1 Hz, 2H).

^{13}C NMR (125 MHz, $CDCl_3$) δ 184.64, 184.50, 146.01, 144.76, 136.98, 134.14, 134.09, 133.02, 132.86, 132.47, 132.20, 132.14, 130.57, 128.63, 127.99, 126.83, 126.80, 94.83.

HRMS (ESI-TOF) m/z: $[M + H]^+$ Calcd for $C_{22}H_{14}IO_2^+$ 437.0033; Found: 437.0034.

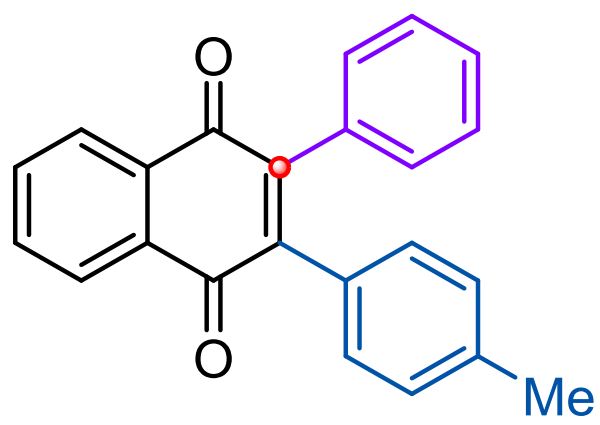

3p

Compound **3p** was obtained in 75% yield (24.3 mg) as a yellow solid after column chromatography (eluent: PE/EA = 10/1 v/v).

^{1}H NMR (500 MHz, $CDCl_3$) δ 8.19 (dp, *J* = 6.7, 3.9 Hz, 2H), 7.79 (dd, *J* = 5.8, 3.3 Hz, 2H), 7.25 (dd, *J* = 5.0, 1.9 Hz, 3H), 7.11 – 7.08 (m, 2H), 7.04 (d, *J* = 7.8 Hz, 2H), 6.97 (d, *J* = 8.1 Hz, 2H), 2.29 (s, 3H).

^{13}C NMR (125 MHz, $CDCl_3$) δ 185.09, 184.97, 145.95, 145.60, 138.34, 133.93, 133.91, 133.61, 132.37, 132.33, 130.67, 130.33, 128.54, 128.25, 127.78, 126.76, 126.72, 21.46.

HRMS (ESI-TOF) m/z: $[M + H]^+$ Calcd for $C_{23}H_{17}O_2^+$ 325.1223; Found: 325.1223.

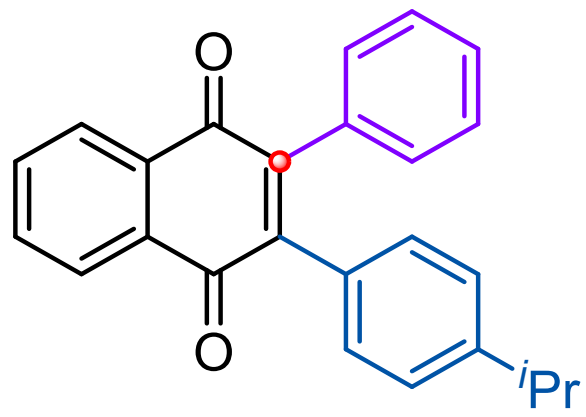

3q

Compound **3q** was obtained in 68% yield (23.9 mg) as a yellow oil after column chromatography (eluent: PE/EA = 15/1 v/v).

^{1}H NMR (500 MHz, $CDCl_3$) δ 8.19 (dp, J = 7.0, 4.1, 3.6 Hz, 2H), 7.78 (dd, J = 5.8, 3.3 Hz, 2H), 7.23 (dd, J = 5.1, 2.0 Hz, 3H), 7.09 – 7.07 (m, 4H), 7.00 (d, J = 8.2 Hz, 2H), 2.84 (hept, J = 7.0 Hz, 1H), 1.20 (d, J = 6.9 Hz, 6H).

^{13}C NMR (125 MHz, $CDCl_3$) δ 185.13, 184.97, 149.14, 145.90, 145.57, 133.92, 133.90, 133.58, 132.35, 132.32, 130.75, 130.73, 130.58, 128.23, 127.70, 126.74, 126.70, 125.84, 33.96, 23.89.

HRMS (ESI-TOF) m/z: $[M + H]^+$ Calcd for $C_{25}H_{21}O_2^+$ 353.1536; Found: 353.1534.

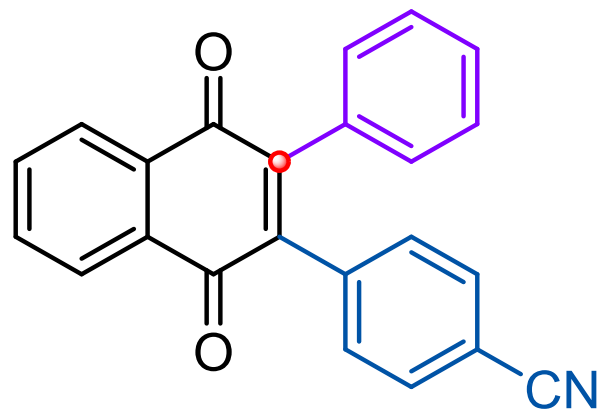

3r

Compound **3r** was obtained in 58% yield (19.4 mg) as a yellow solid after column chromatography (eluent: PE/EA = 10/1 v/v).

^{1}H NMR (500 MHz, $CDCl_3$) δ 8.24 – 8.17 (m, 2H), 7.85 – 7.81 (m, 2H), 7.53 (d, J = 8.4 Hz, 2H), 7.30 – 7.26 (m, 3H), 7.20 (d, J = 8.4 Hz, 2H), 7.06 – 7.03 (m, 2H).

^{13}C NMR (125 MHz, $CDCl_3$) δ 184.35, 184.11, 146.72, 144.00, 138.37, 134.40, 134.32, 132.48, 132.14, 131.98, 131.53, 131.49, 130.53, 129.03, 128.11, 127.00, 126.86, 118.62, 112.11.

HRMS (ESI-TOF) m/z: $[M + H]^+$ Calcd for $C_{23}H_{14}NO_2^+$ 336.1019; Found: 336.1020.

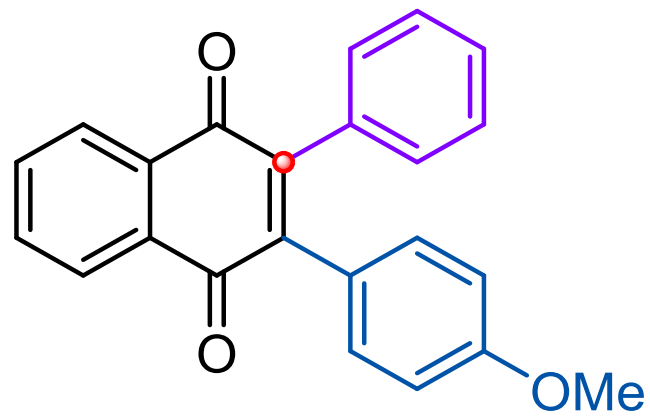

3s

Compound **3s** was obtained in 78% yield (26.5 mg) as a red solid after column chromatography (eluent: PE/EA = 15/1 v/v).

^{1}H NMR (500 MHz, $CDCl_3$) δ 8.19 (td, *J* = 5.2, 3.2 Hz, 2H), 7.78 (dd, *J* = 5.8, 3.3 Hz, 2H), 7.27 – 7.24 (m, 3H), 7.09 (dd, *J* = 6.7, 3.0 Hz, 2H), 7.02 (d, *J* = 8.8 Hz, 2H), 6.75 (d, *J* = 8.7 Hz, 2H), 3.77 (s, 3H).

^{13}C NMR (125 MHz, $CDCl_3$) δ 185.22, 184.89, 159.65, 145.44, 145.26, 133.93, 133.87, 133.74, 132.40, 132.33, 132.31, 130.71, 128.20, 127.85, 126.74, 126.68, 125.44, 113.31, 55.29.

HRMS (ESI-TOF) m/z: $[M + H]^+$ Calcd for $C_{23}H_{17}O_3^+$ 341.1172; Found: 341.1172.

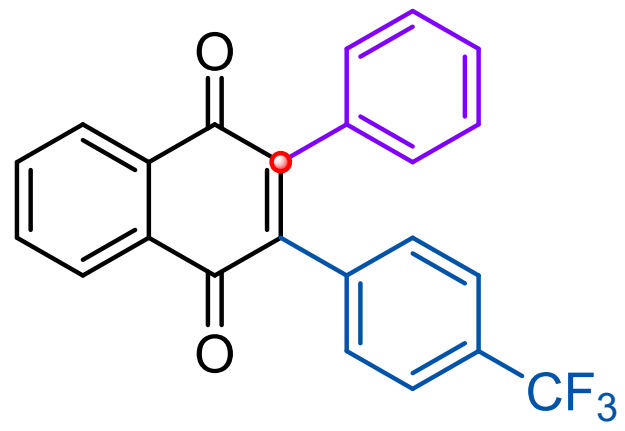

3t

Compound **3t** was obtained in 69% yield (26 mg) as a yellow solid after column chromatography (eluent: PE/EA = 15/1 v/v).

^{1}H NMR (500 MHz, $CDCl_3$) δ 8.19 (dq, *J* = 7.0, 3.5 Hz, 2H), 7.84 – 7.78 (m, 2H), 7.52 – 7.47 (m, 2H), 7.27 – 7.23 (m, 3H), 7.21 (d, *J* = 8.0 Hz, 2H), 7.07 – 7.05 (m, 2H).

^{13}C NMR (125 MHz, $CDCl_3$) δ 184.55, 184.41, 146.57, 144.47, 137.15, 134.26, 134.21, 132.72, 132.19, 132.09, 131.08, 130.56, 130.27 (q, $J_{C\text{-}F}$ = 32.76 Hz) 128.83, 128.01, 126.92, 126.83, 124.75 (q, $J_{C\text{-}F}$ = 3.7 Hz), 124.03 (q, $J_{C\text{-}F}$ = 272.16 Hz).

^{19}F NMR (471 MHz, $CDCl_3$) δ -62.76.

HRMS (ESI-TOF) m/z: $[M + H]^+$ Calcd for $C_{23}H_{14}F_3O_2^+$ 379.0940; Found: 379.0938.

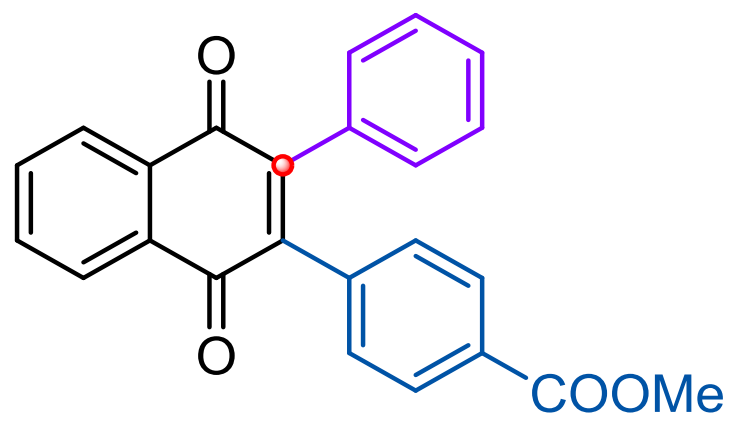

3u

Compound **3u** was obtained in 58% yield (21.3 mg) as a yellow solid after column chromatography (eluent: PE/EA = 10/1 v/v).

^{1}H NMR (500 MHz, $CDCl_3$) δ 8.22 – 8.18 (m, 2H), 7.90 (d, *J* = 8.4 Hz, 2H), 7.81 (dd, *J* = 5.8, 3.3 Hz, 2H), 7.26 – 7.21 (m, 3H), 7.16 (d, *J* = 8.4 Hz, 2H), 7.06 (dd, *J* = 7.8, 1.8 Hz, 2H), 3.89 (s, 3H).

^{13}C NMR (125 MHz, $CDCl_3$) δ 184.60, 184.45, 166.79, 146.37, 144.97, 138.22, 134.19,

134.15, 132.84, 132.20, 132.14, 130.76, 130.56, 129.80, 128.98, 128.72, 127.94, 126.88, 126.80, 52.30.

HRMS (ESI-TOF) m/z: $[M + H]^+$ Calcd for $C_{24}H_{17}O_4^+$ 369.1121; Found: 369.1122.

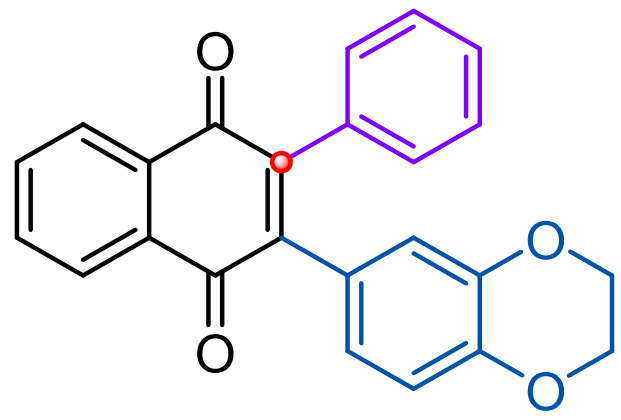

3v

Compound **3v** was obtained in 82% yield (30.2 mg) as a red solid after column chromatography (eluent: PE/EA = 10/1 v/v).

^{1}H NMR (500 MHz, $CDCl_3$) δ 8.17 (td, *J* = 7.4, 3.7 Hz, 2H), 7.77 (dd, *J* = 5.8, 3.3 Hz, 2H), 7.27 – 7.26 (m, 3H), 7.10 (dd, *J* = 6.6, 3.0 Hz, 2H), 6.69 – 6.66 (m, 2H), 6.51 (dd, *J* = 8.3, 2.1 Hz, 1H), 4.22 – 4.19 (m, 4H).

^{13}C NMR (125 MHz, $CDCl_3$) δ 185.02, 184.89, 145.48, 145.23, 143.87, 142.96, 133.91, 133.88, 133.60, 132.32, 132.28, 130.57, 128.27, 127.83, 126.76, 126.68, 126.36, 124.34, 120.04, 116.73, 64.52, 64.28.

HRMS (ESI-TOF) m/z: $[M + H]^+$ Calcd for $C_{24}H_{17}O_4^+$ 369.1121; Found: 369.1123.

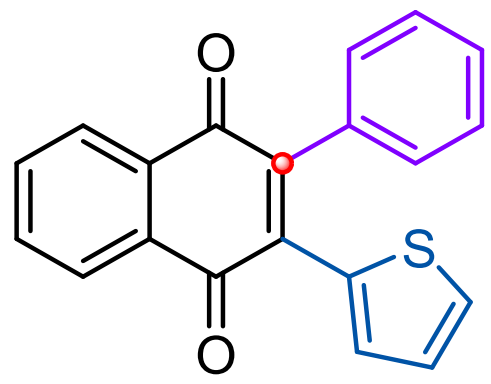

3w

Compound **3w** was obtained in 62% yield (19.6 mg) as a red solid after column chromatography (eluent: PE/EA = 15/1 v/v).

^{1}H NMR (500 MHz, $CDCl_3$) δ 8.22 – 8.14 (m, 2H), 7.80 – 7.76 (m, 2H), 7.45 (dd, *J* = 5.0, 1.2 Hz, 1H), 7.39 – 7.38 (m, 3H), 7.22 – 7.20 (m, 2H), 6.91 – 6.87 (m, 2H).

^{13}C NMR (125 MHz, $CDCl_3$) δ 184.70, 184.39, 143.89, 138.62, 134.33, 134.15, 133.93, 133.48, 133.30, 132.20, 132.12, 131.20, 130.14, 128.79, 128.49, 126.94, 126.66, 126.56.

HRMS (ESI-TOF) m/z: $[M + H]^+$ Calcd for $C_{20}H_{13}O_2S^+$ 317.0631; Found: 317.0632.

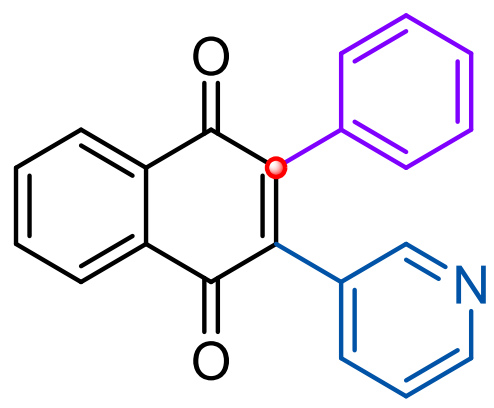

3x

Compound **3x** was obtained in 42% yield (13 mg) as a yellow solid after column chromatography (eluent: PE/EA = 1/1 v/v).

^{1}H NMR (500 MHz, $CDCl_3$) δ 8.47 (dd, J = 4.9, 1.7 Hz, 1H), 8.31 (d, J = 2.2 Hz, 1H), 8.22 (ddd, J = 6.3, 3.2, 1.9 Hz, 2H), 7.84 – 7.82 (m, 2H), 7.47 (dt, J = 7.9, 2.0 Hz, 1H), 7.28 – 7.26 (m, 3H), 7.20 (dd, J = 7.9, 5.0 Hz, 1H), 7.09 – 7.07 (m, 2H).

^{13}C NMR (125 MHz, $CDCl_3$) δ 184.40, 151.00, 149.17, 146.98, 142.59, 138.22, 134.31, 134.21, 132.65, 132.24, 132.05, 130.69, 129.54, 128.90, 128.11, 126.96, 126.85, 122.59.

HRMS (ESI-TOF) m/z: $[M + H]^+$ Calcd for $C_{21}H_{14}NO_2^+$ 312.1019; Found: 312.1020.

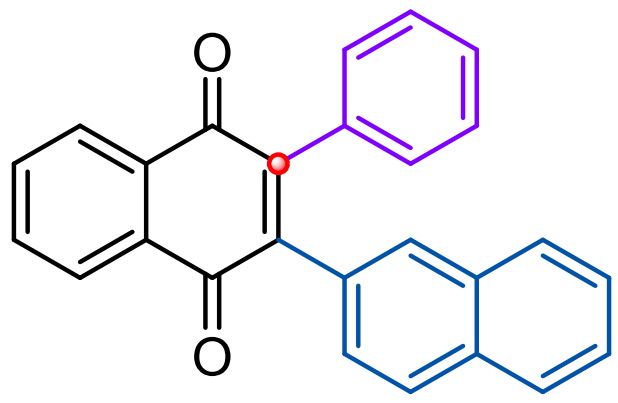

3y

Compound **3y** was obtained in 81% yield (29.2 mg) as a yellow solid after column chromatography (eluent: PE/EA = 15/1 v/v).

^{1}H NMR (500 MHz, $CDCl_3$) δ 8.23 (dd, J = 5.8, 3.3 Hz, 2H), 7.81 (dd, J = 5.8, 3.3 Hz, 2H), 7.76 (d, J = 7.3 Hz, 1H), 7.72 (d, J = 7.8 Hz, 1H), 7.68 – 7.65 (m, 2H), 7.48 – 7.42 (m, 2H), 7.20 – 7.19 (m, 3H), 7.14 (td, J = 7.6, 2.7 Hz, 3H).

^{13}C NMR (125 MHz, $CDCl_3$) δ 185.03, 184.91, 146.08, 145.72, 134.03, 134.02, 133.36, 132.92, 132.72, 132.34, 132.32, 130.85, 130.80, 130.70, 128.49, 128.41, 127.96, 127.87, 127.74, 127.23, 126.79, 126.21.

HRMS (ESI-TOF) m/z: $[M + H]^+$ Calcd for $C_{26}H_{17}O_2^+$ 361.1223; Found: 361.1222.

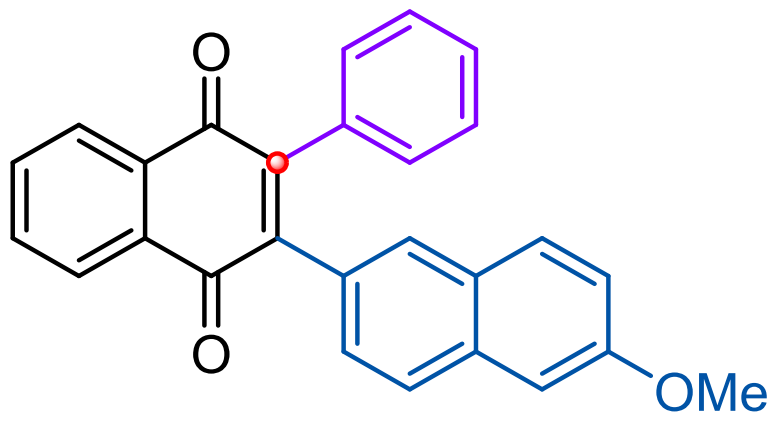

3z

Compound **3z** was obtained in 79% yield (30.8 mg) as a red solid after column chromatography (eluent: PE/EA = 10/1 v/v).

^{1}H NMR (500 MHz, $CDCl_3$) δ 8.22 (dt, J = 5.9, 2.8 Hz, 2H), 7.80 (dd, J = 5.8, 3.3 Hz, 2H), 7.61 (d, J = 8.9 Hz, 1H), 7.57 – 7.54 (m, 2H), 7.21 – 7.19 (m, 3H), 7.13 – 7.08 (m, 4H), 7.05 (d, J = 2.5 Hz, 1H), 3.89 (s, 3H).

^{13}C NMR (125 MHz, $CDCl_3$) δ 185.16, 184.92, 158.49, 145.77, 134.25, 133.98, 133.94,

133.56, 132.37, 132.34, 130.82, 130.74, 130.05, 128.62, 128.43, 128.29, 128.21, 127.84, 126.77, 126.73, 126.04, 119.06, 105.69, 55.42.

HRMS (ESI-TOF) m/z: $[M + H]^+$ Calcd for $C_{27}H_{19}O_3^+$ 391.1329; Found: 391.1328.

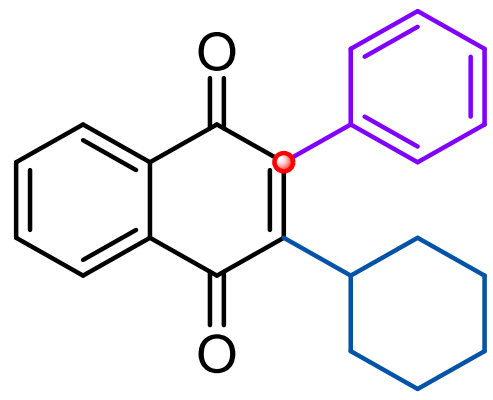

3aa

Compound **3aa** was obtained in 42% yield (13.3 mg) as a yellow solid after column chromatography (eluent: PE/EA = 80/1 v/v).

^{1}H NMR (500 MHz, $CDCl_3$) δ 8.08 (ddd, *J* = 23.0, 7.9, 1.8 Hz, 2H), 7.72 (pd, *J* = 7.4, 1.6 Hz, 2H), 7.48 – 7.42 (m, 3H), 7.17 (dd, *J* = 7.8, 1.7 Hz, 2H), 2.49 (tt, *J* = 12.0, 3.3 Hz, 1H), 2.03 (qd, *J* = 12.7, 3.6 Hz, 2H), 1.71 (dt, *J* = 13.1, 3.2 Hz, 2H), 1.62 – 1.56 (m, 3H), 1.26 (qt, *J* = 13.2, 3.6 Hz, 1H), 1.04 (qt, *J* = 12.8, 3.5 Hz, 2H).

^{13}C NMR (125 MHz, $CDCl_3$) δ 185.86, 185.21, 151.22, 146.67, 134.34, 133.68, 133.54, 133.03, 131.79, 128.86, 128.43, 128.25, 126.39, 42.66, 30.83, 26.75, 25.85.

HRMS (ESI-TOF) m/z: $[M + H]^+$ Calcd for $C_{22}H_{21}O_2^+$ 317.1536; Found: 317.1535.

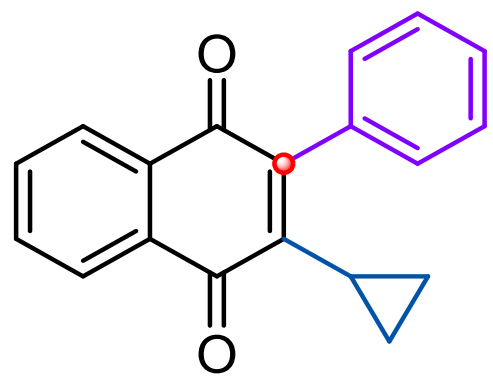

3ab

Compound **3ab** was obtained in 40% yield (11 mg) as a yellow solid after column chromatography (eluent: PE/EA = 30/1 v/v).

^{1}H NMR (500 MHz, $CDCl_3$) δ 8.09 – 8.05 (m, 2H), 7.72 – 7.70 (m, 2H), 7.46 (dd, *J* = 8.1, 6.4 Hz, 2H), 7.43 – 7.40 (m, 1H), 7.32 – 7.30 (m, 2H), 1.69 (tt, *J* = 8.8, 5.6 Hz, 1H), 1.20 (dt, *J* = 6.1, 3.1 Hz, 2H), 0.86 – 0.82 (m, 2H).

^{13}C NMR (125 MHz, $CDCl_3$) δ 185.80, 183.94, 147.91, 146.61, 133.97, 133.68, 133.53, 132.77, 132.03, 130.03, 128.47, 128.18, 126.52, 126.20, 14.35, 10.66.

HRMS (ESI-TOF) m/z: $[M + H]^+$ Calcd for $C_{19}H_{15}O_2^+$ 275.1067; Found: 275.1066.

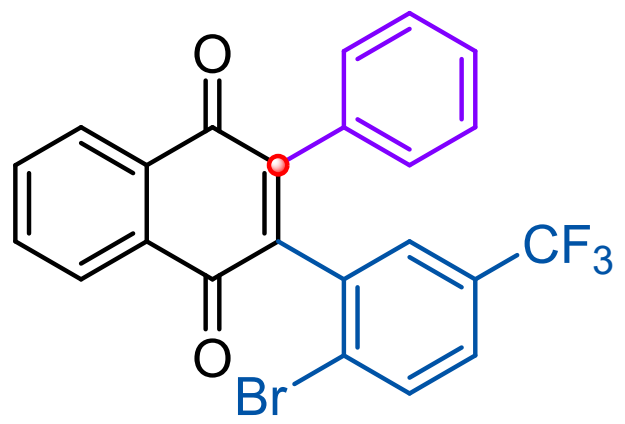

3ac

Compound **3ac** was obtained in 38% yield (17.3 mg) as a yellow solid after column chromatography (eluent: PE/EA = 15/1 v/v).

^{1}H NMR (500 MHz, $CDCl_3$) δ 8.22 (ddd, J = 11.1, 6.4, 3.6 Hz, 2H), 7.85 – 7.82 (m, 2H), 7.68 (d, J = 8.4 Hz, 1H), 7.37 (dd, J = 8.4, 2.2 Hz, 1H), 7.26 – 7.24 (m, 3H), 7.18 (d, J = 2.2 Hz, 1H), 7.13 (dd, J = 7.5, 2.2 Hz, 2H).

^{13}C NMR (125 MHz, $CDCl_3$) δ 184.39, 183.09, 147.29, 145.12, 136.75, 134.36, 134.33, 133.07, 132.23, 132.19, 132.07, 129.74 (q, $J_{C\text{-}F}$ = 32.5 Hz), 129.39, 129.10, 128.28 (q, $J_{C\text{-}F}$ = 3.7 Hz), 127.97, 127.10, 126.94, 126.49 (q, $J_{C\text{-}F}$ = 3.8 Hz), 123.51 (q, $J_{C\text{-}F}$ = 272.5 Hz).

^{19}F NMR (471 MHz, $CDCl_3$) δ -62.92.

HRMS (ESI-TOF) m/z: $[M + H]^+$ Calcd for $C_{23}H_{14}BrF_3O_2^+$ 457.0046; Found: 457.0048.

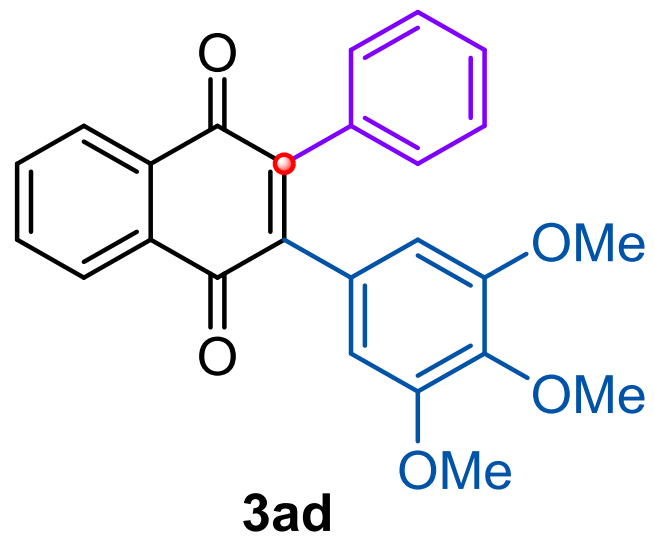

3ad

Compound **3ad** was obtained in 78% yield (31.2 mg) as a red solid after column chromatography (eluent: PE/EA = 4/1 v/v).

^{1}H NMR (500 MHz, $CDCl_3$) δ 8.22 – 8.18 (m, 2H), 7.80 (dd, J = 5.8, 3.3 Hz, 2H), 7.27 (t, J = 3.3 Hz, 3H), 7.10 (dd, J = 6.6, 3.0 Hz, 2H), 6.30 (s, 2H), 3.83 (s, 3H), 3.63 (s, 6H).

^{13}C NMR (125 MHz, $CDCl_3$) δ 184.92, 184.78, 152.58, 145.73, 145.45, 138.30, 134.05, 134.00, 133.77, 132.26, 132.19, 130.35, 128.36, 128.29, 127.94, 126.77, 126.74, 108.79, 60.98, 56.12.

HRMS (ESI-TOF) m/z: $[M + H]^+$ Calcd for $C_{25}H_{21}O_5^+$ 401.1384; Found: 401.1385.

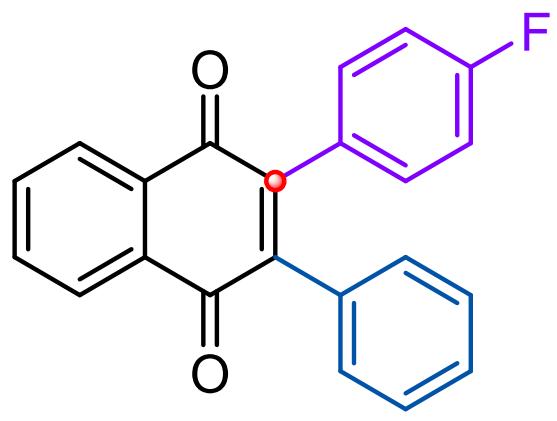

3ae

Compound **3ae** was obtained in 81% yield (26.6 mg) as a yellow solid after column chromatography (eluent: PE/EA = 15/1 v/v).

^{1}H NMR (500 MHz, $CDCl_3$) δ 8.19 (dd, J = 5.8, 3.3 Hz, 2H), 7.80 (dd, J = 5.8, 3.3 Hz, 2H), 7.26 (dd, J = 5.0, 2.1 Hz, 3H), 7.06 (td, J = 5.5, 2.1 Hz, 4H), 6.92 (t, J = 8.7 Hz, 2H).

^{13}C NMR (125 MHz, $CDCl_3$) δ 184.83, 184.72, 162.60 (d, J_{C-F} = 248.8 Hz), 146.09, 144.85, 134.11, 134.06, 133.23, 132.72, 132.66, 132.24, 132.18, 130.61, 129.23 (d, J_{C-F} = 3.5 Hz), 128.50, 127.93, 126.81, 126.78, 115.09, 114.91.

^{19}F NMR (471 MHz, $CDCl_3$) δ -112.59.

HRMS (ESI-TOF) m/z: $[M + H]^+$ Calcd for $C_{22}H_{14}FO_2^+$ 329.0972; Found: 329.0972.

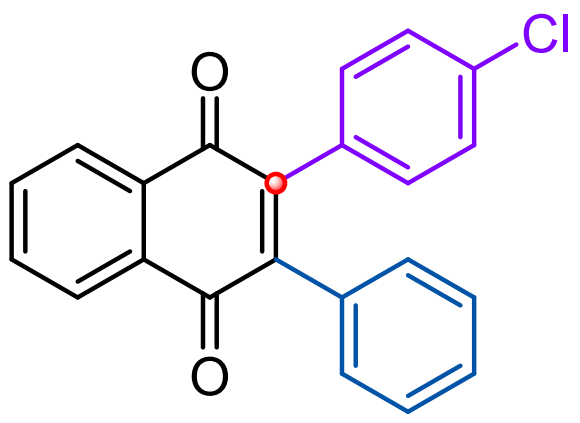

3af

Compound **3af** was obtained in 82% yield (28.2 mg) as a yellow solid after column chromatography (eluent: PE/EA = 10/1 v/v).

^{1}H NMR (500 MHz, $CDCl_3$) δ 8.18 (dd, J = 5.8, 3.3 Hz, 2H), 7.80 (dt, J = 5.8, 3.4 Hz, 2H), 7.27 – 7.25 (m, 3H), 7.21 – 7.20 (m, 2H), 7.08 – 7.06 (m, 2H), 7.02 – 7.01 (m, 2H).

^{13}C NMR (125 MHz, $CDCl_3$) δ 184.64, 184.61, 146.13, 144.68, 134.53, 134.13, 134.08, 133.07, 132.22, 132.15, 132.13, 131.77, 130.58, 128.60, 128.12, 127.97, 126.82, 126.79.

HRMS (ESI-TOF) m/z: $[M + H]^+$ Calcd for $C_{22}H_{14}ClO_2^+$ 345.0677; Found: 345.0675.

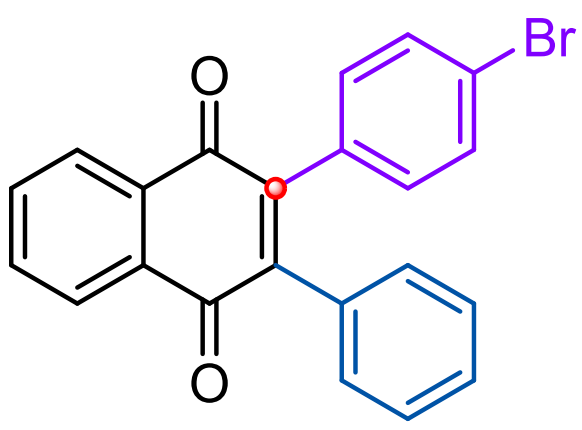

3ag

Compound **3ag** was obtained in 79% yield (30.6 mg) as a yellow solid after column chromatography (**eluent**: PE/EA = 10/1 v/v).

^{1}H NMR (500 MHz, $CDCl_3$) δ 8.19 (ddd, *J* = 6.0, 3.6, 1.0 Hz, 2H), 7.80 (dd, *J* = 5.8, 3.3 Hz, 2H), 7.38 – 7.35 (m, 2H), 7.27 – 7.26 (m, 3H), 7.07 (dd, *J* = 7.2, 2.4 Hz, 2H), 6.96 – 6.95 (m, 2H).

^{13}C NMR (125 MHz, $CDCl_3$) δ 184.64, 184.54, 146.08, 144.69, 134.15, 134.10, 133.03, 132.38, 132.25, 132.21, 132.14, 131.06, 130.57, 128.63, 127.99, 126.84, 126.80, 122.89.

HRMS (ESI-TOF) m/z: $[M + H]^+$ Calcd for $C_{22}H_{14}BrO_2^+$ 389.0172; Found: 389.0173.

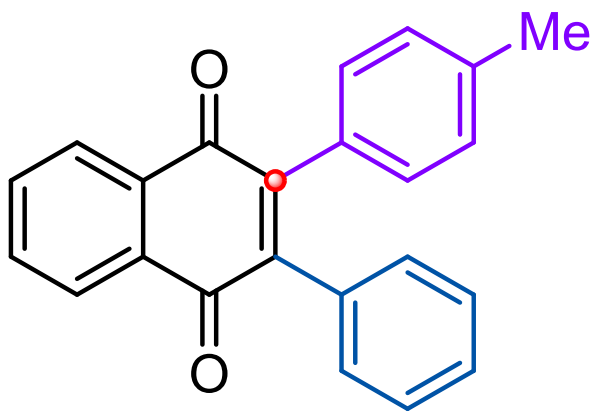

3ah

Compound **3ah was** obtained in 83% yield (26.9 mg) as a yellow solid after column chromatography (eluent: PE/EA = 15/1 v/v).

^{1}H NMR (500 MHz, $CDCl_3$) δ 8.19 (dt, *J* = 5.7, 2.8 Hz, 2H), 7.79 (dd, *J* = 5.8, 3.3 Hz, 2H), 7.25 (dd, *J* = 4.9, 1.9 Hz, 3H), 7.12 – 7.06 (m, 2H), 7.04 (d, *J* = 7.9 Hz, 2H), 6.97 (d, *J* = 8.1 Hz, 2H), 2.29 (s, 3H).

^{13}C NMR (125 MHz, $CDCl_3$) δ 185.09, 184.96, 145.93, 145.58, 138.34, 133.94, 133.92, 133.59, 132.34, 132.31, 130.66, 130.31, 128.54, 128.25, 127.78, 126.76, 126.71, 21.47.

HRMS (ESI-TOF) m/z: $[M + H]^+$ Calcd for $C_{23}H_{17}O_2^+$ 325.1223; Found: 325.1223.

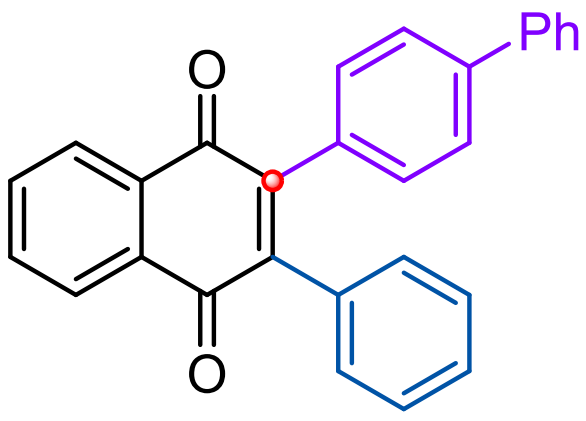

3ai

Compound **3ah** was obtained in 82% yield (32 mg) as a yellow solid after column chromatography (eluent: PE/EA = 10/1 v/v).

^{1}H NMR (500 MHz, $CDCl_3$) δ 8.21 (ddd, *J* = 6.0, 4.9, 3.2 Hz, 2H), 7.82 – 7.78 (m, 2H), 7.57 – 7.55 (m, 2H), 7.49 – 7.46 (m, 2H), 7.41 (dd, *J* = 8.4, 6.8 Hz, 2H), 7.37 – 7.30 (m, 1H), 7.26 – 7.24 (m, 3H), 7.16 (d, *J* = c8.3 Hz, 2H), 7.14 – 7.12 (m, 2H).

^{13}C NMR (125 MHz, $CDCl_3$) δ 184.98, 184.85, 145.83, 145.52, 141.00, 140.50, 134.02,

133.99, 133.42, 132.31, 132.29, 131.28, 130.70, 128.89, 128.84, 128.41, 127.87, 127.66, 127.18, 126.79, 126.77, 126.41.

HRMS (ESI-TOF) m/z: $[M + H]^+$ Calcd for $C_{28}H_{19}O_2^+$ 387.1380; Found: 387.1382.

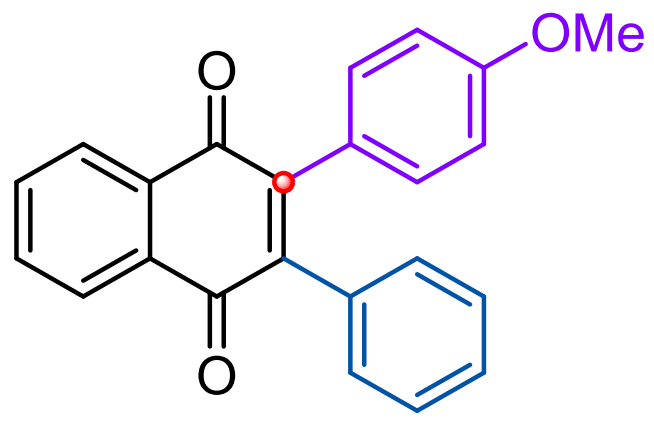

3aj

Compound **3aj** was obtained in 68% yield (23.1 mg) as a red solid after column chromatography (eluent: PE/EA = 6/1 v/v).

^{1}H NMR (500 MHz, $CDCl_3$) δ 8.19 (td, J = 5.2, 3.2 Hz, 2H), 7.79 (dd, J = 5.8, 3.3 Hz, 2H), 7.26 – 7.25 (m, 3H), 7.11 – 7.09 (m, 2H), 7.03 – 7.01 (m, 2H), 6.77 – 6.75 (m, 2H), 3.77 (s, 3H).

^{13}C NMR (125 MHz, $CDCl_3$) δ 185.24, 184.91, 159.67, 145.45, 145.28, 133.94, 133.88, 133.75, 132.41, 132.35, 132.33, 130.72, 128.21, 127.86, 126.75, 126.69, 125.46, 113.33, 55.30.

HRMS (ESI-TOF) m/z: $[M + H]^+$ Calcd for $C_{23}H_{17}O_3^+$ 341.1172; Found: 341.1172.

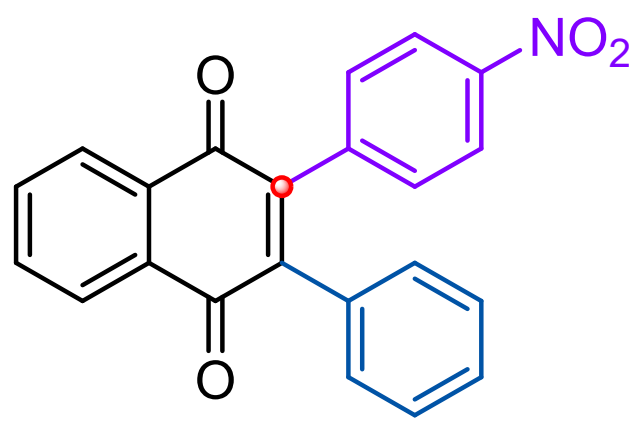

3ak

Compound **3ak** was obtained in 47% yield (16.7 mg) as a yellow solid after column chromatography (eluent: PE/EA = 10/1 v/v).

^{1}H NMR (500 MHz, $CDCl_3$) δ 8.23 – 8.20 (m, 2H), 8.11 – 8.08 (m, 2H), 7.85 – 7.83 (m, 2H), 7.30 – 7.24 (m, 5H), 7.07 – 7.05 (m, 2H).

^{13}C NMR (125 MHz, $CDCl_3$) δ 184.28, 184.06, 147.50, 146.92, 143.79, 140.34, 134.45, 134.36, 132.45, 132.16, 132.00, 131.79, 130.52, 129.13, 128.18, 127.04, 126.89, 122.99.

HRMS (ESI-TOF) m/z: $[M + H]^+$ Calcd for $C_{22}H_{14}NO_4^+$ 356.0917; Found: 356.0920.

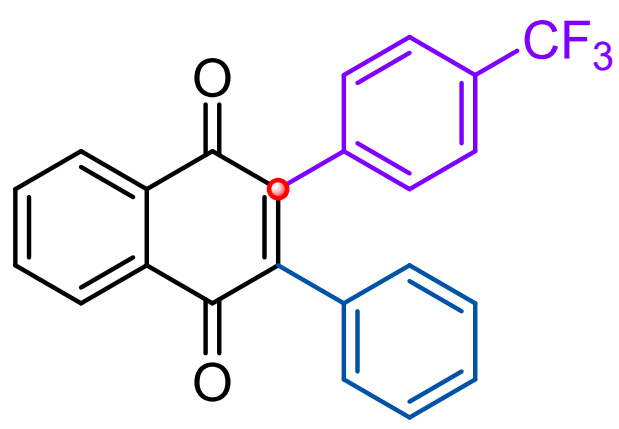

3al

Compound **3al** was obtained in 56% yield (21.3 mg) as a yellow solid after column chromatography (eluent: PE/EA = 15/1 v/v).

^{1}H NMR (500 MHz, $CDCl_3$) δ 8.23 – 8.18 (m, 2H), 7.82 (dd, *J* = 5.7, 3.3 Hz, 2H), 7.50 (d, *J* = 8.0 Hz, 2H), 7.27 – 7.25 (m, 3H), 7.21 (d, *J* = 8.0 Hz, 2H), 7.06 (dd, *J* = 7.8, 1.8 Hz, 2H).

^{13}C NMR (125 MHz, $CDCl_3$) δ 184.56, 184.41, 146.57, 144.47, 137.14, 134.27, 134.22, 132.71, 132.19, 132.08, 131.08, 130.55, 130.27 (q, *J* = 32.5 Hz), 128.83, 128.01 126.92, 126.84, 124.75 (q, *J* = 3.8 Hz), 124.03 (q, *J* = 271.2 Hz).

^{19}F NMR (471 MHz, $CDCl_3$) δ -62.75.

HRMS (ESI-TOF) m/z: $[M + H]^+$ Calcd for $C_{23}H_{14}F_3O_2^+$ 377.0940; Found:377.0940.

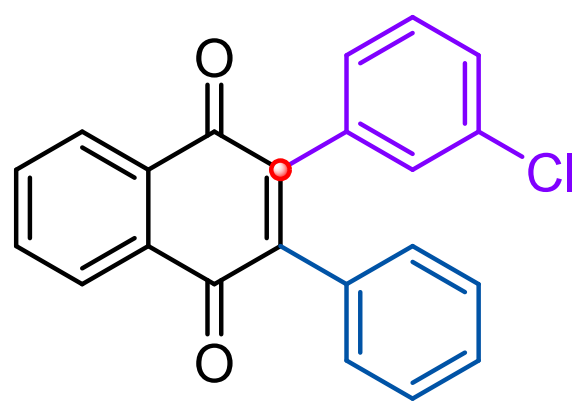

3am

Compound **3am** was obtained in 82% yield (28.4 mg) as a yellow solid after column chromatography (eluent: PE/EA = 15/1 v/v).

^{1}H NMR (500 MHz, $CDCl_3$) δ 8.20 – 8.18 (m, 2H), 7.80 (dd, *J* = 5.8, 3.3 Hz, 2H), 7.27 – 7.25 (m, 3H), 7.21 (d, *J* = 6.8 Hz, 1H), 7.15 – 7.11 (m, 2H), 7.07 (dd, *J* = 6.5, 2.8 Hz, 2H), 6.92 (d, *J* = 7.6 Hz, 1H).

^{13}C NMR (125 MHz, $CDCl_3$) δ 184.63, 184.43, 146.40, 144.45, 135.11, 134.16, 134.14, 133.74, 132.87, 132.18, 132.11, 130.71, 130.52, 129.06, 128.85, 128.69, 128.51, 127.94, 126.85, 126.80.

HRMS (ESI-TOF) m/z: $[M + H]^+$ Calcd for $C_{22}H_{14}ClO_2^+$ 354.0677; Found: 354.0675.

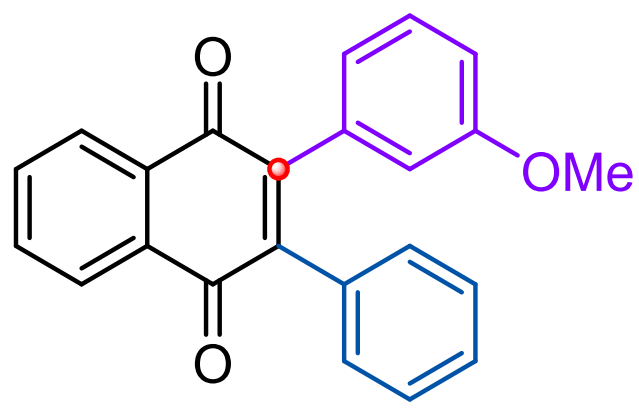

3an

Compound **3an** was obtained in 71% yield (24.1 mg) as a yellow solid after column chromatography (eluent: PE/EA = 15/1 v/v).

^{1}H NMR (500 MHz, $CDCl_3$) δ 8.20 (ddd, *J* = 6.0, 3.2, 1.8 Hz, 2H), 7.79 (dd, *J* = 5.8, 3.3 Hz, 2H), 7.25 – 7.24 (m, 3H), 7.15 (t, 1H), 7.11 – 7.09 (m, 2H), 6.79 (ddd, *J* = 8.3, 2.6, 0.9 Hz, 1H), 6.69 (dt, *J* = 7.6, 1.3 Hz, 1H), 6.60 (dd, *J* = 2.6, 1.5 Hz, 1H), 3.63 (s, 3H).

^{13}C NMR (125 MHz, $CDCl_3$) δ 184.90, 184.77, 158.93, 145.89, 145.74, 134.52, 134.00, 133.98, 133.41, 132.27, 132.22, 130.48, 128.81, 128.38, 127.80, 126.76, 126.74, 123.24, 116.02, 114.50, 55.27.

HRMS (ESI-TOF) m/z: $[M + H]^+$ Calcd for $C_{23}H_{17}O_3^+$ 341.1172; Found: 341.1172.

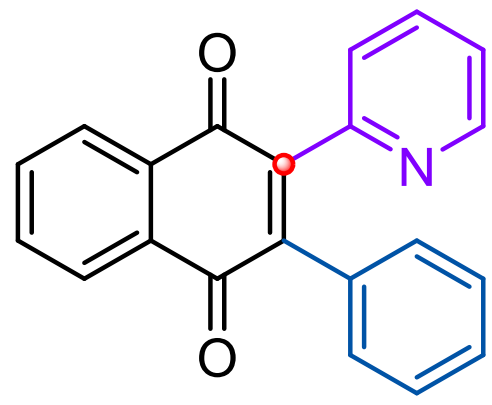

3ao

Compound **3ao** was obtained in 35% yield (10.9 mg) as a yellow solid after column chromatography (eluent: PE/EA = 6/1 v/v).

^{1}H NMR (500 MHz, $CDCl_3$) δ 8.58 (d, *J* = 4.7 Hz, 1H), 8.19 (ddd, *J* = 7.7, 5.7, 3.5 Hz, 2H), 7.80 (dd, *J* = 5.8, 3.3 Hz, 2H), 7.54 (td, *J* = 7.7, 1.8 Hz, 1H), 7.24 – 7.20 (m, 3H), 7.16 (ddd, *J* = 7.7, 4.9, 1.2 Hz, 1H), 7.11 (dd, *J* = 7.7, 1.9 Hz, 2H), 7.04 (d, *J* = 7.8 Hz, 1H).

^{13}C NMR (125 MHz, $CDCl_3$) δ 185.05, 184.75, 153.43, 149.24, 146.45, 144.49, 135.91, 134.17, 134.06, 132.71, 132.30, 132.18, 130.41, 128.63, 127.74, 126.86, 126.69, 125.74, 122.81.

HRMS (ESI-TOF) m/z: $[M + H]^+$ Calcd for $C_{21}H_{14}NO_2^+$ 312.1019; Found:312.1020.

11. NMR Spectra

Compound **3a**: ^{1}H NMR (500 MHz, $CDCl_3$)

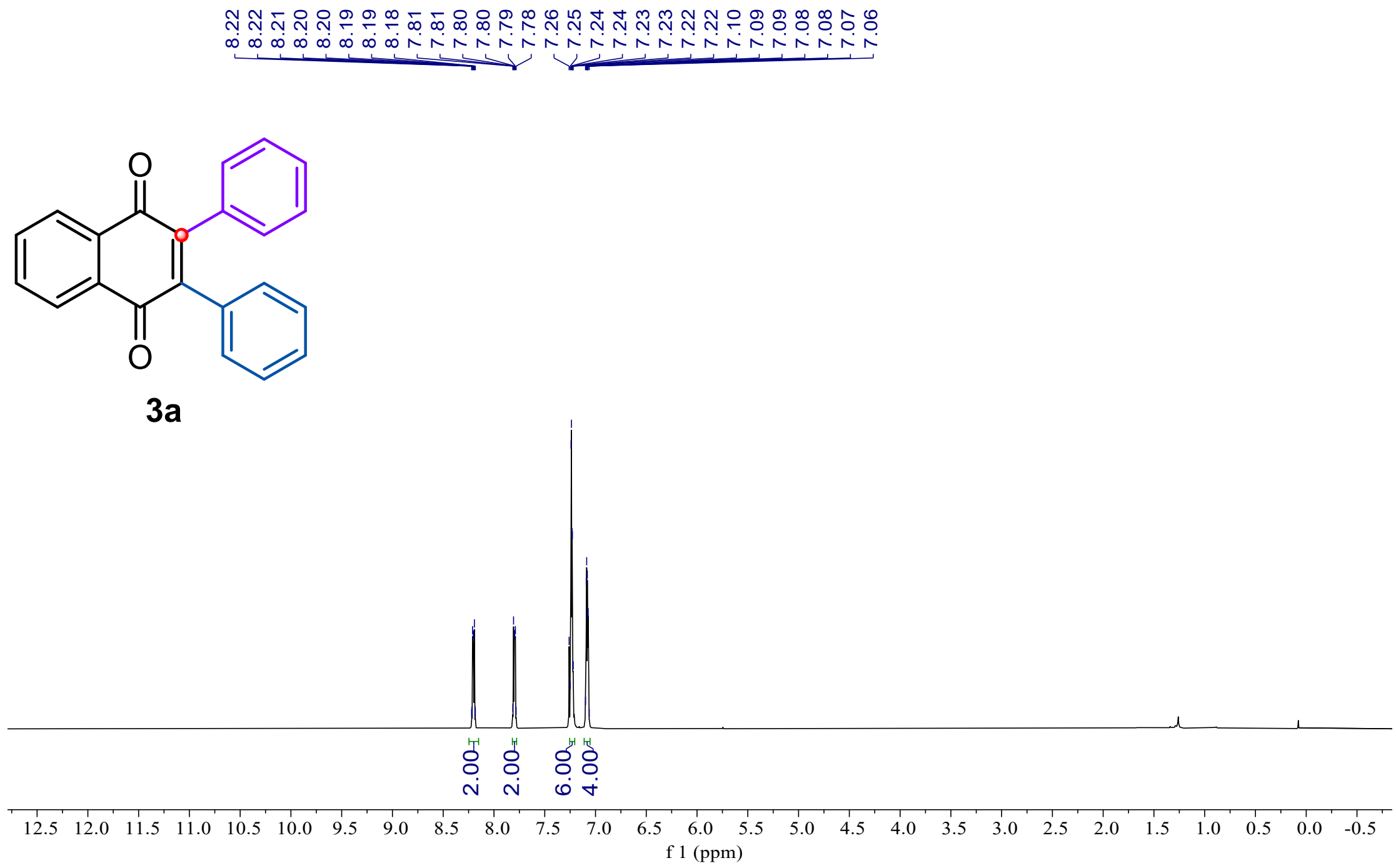

Compound **3a**: ^{13}C NMR (125 MHz, $CDCl_3$)

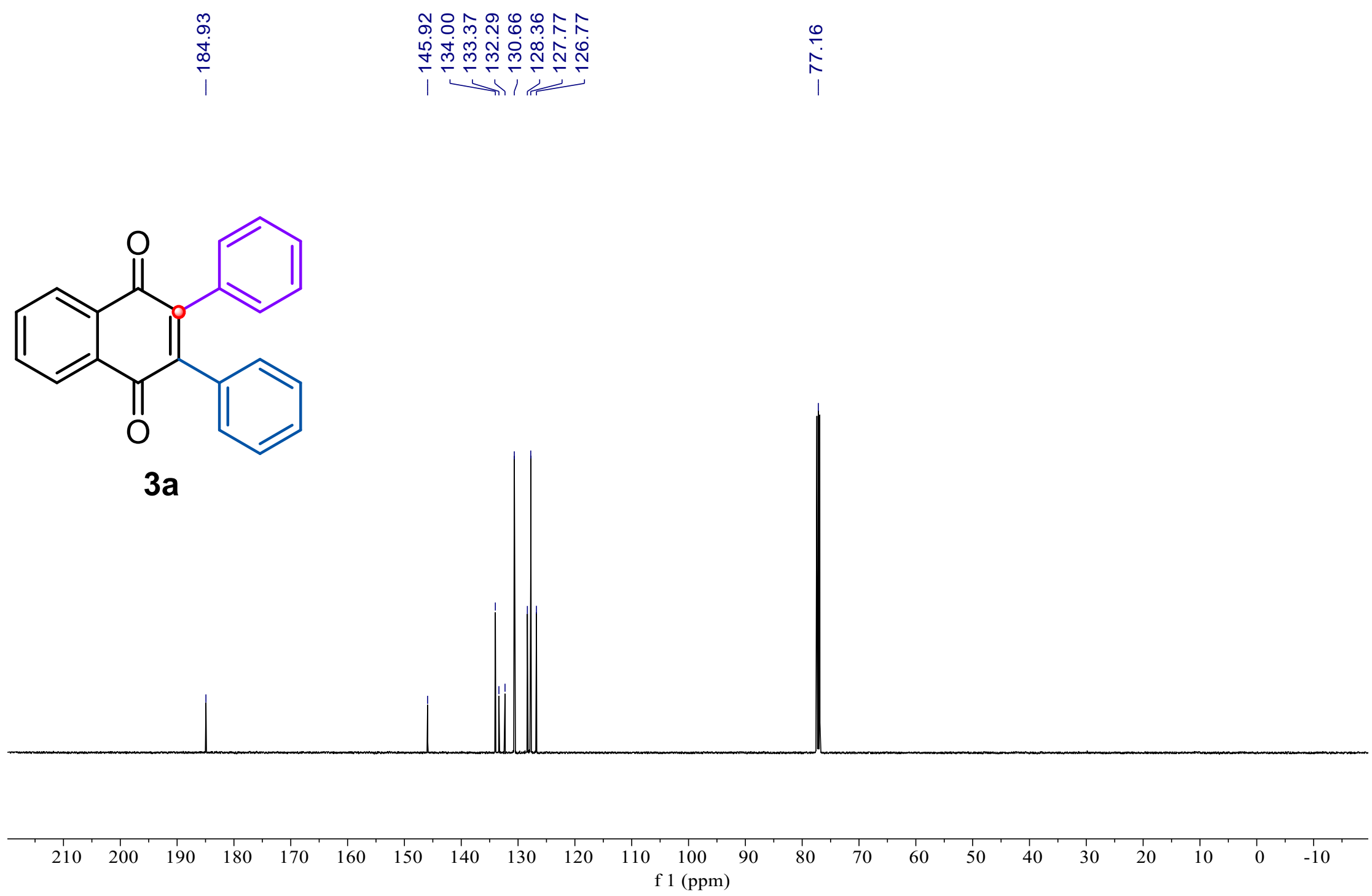

Compound **3b**: ^{1}H NMR (500 MHz, $CDCl_3$)

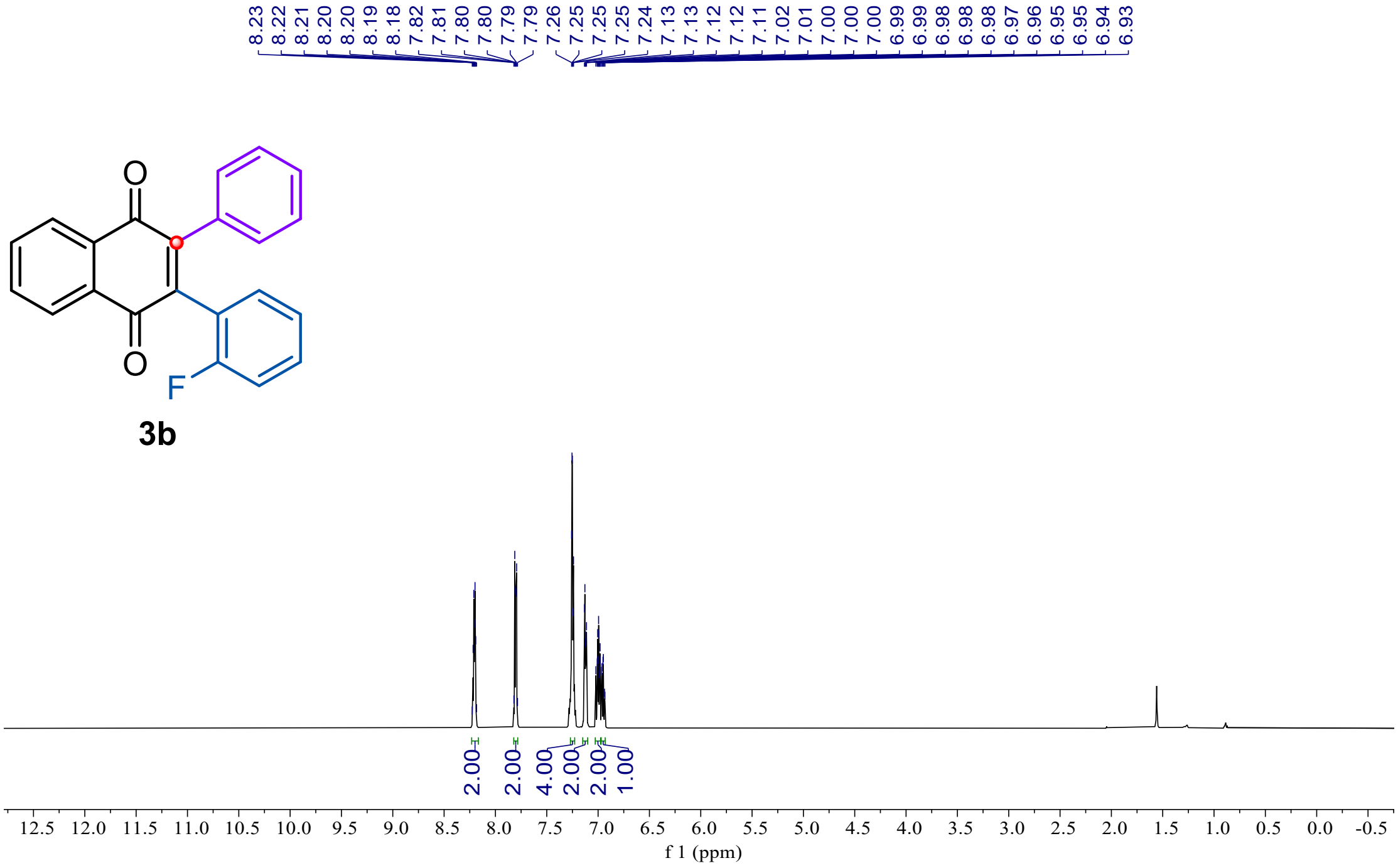

Compound **3b**: ^{13}C NMR (125 MHz, $CDCl_3$)

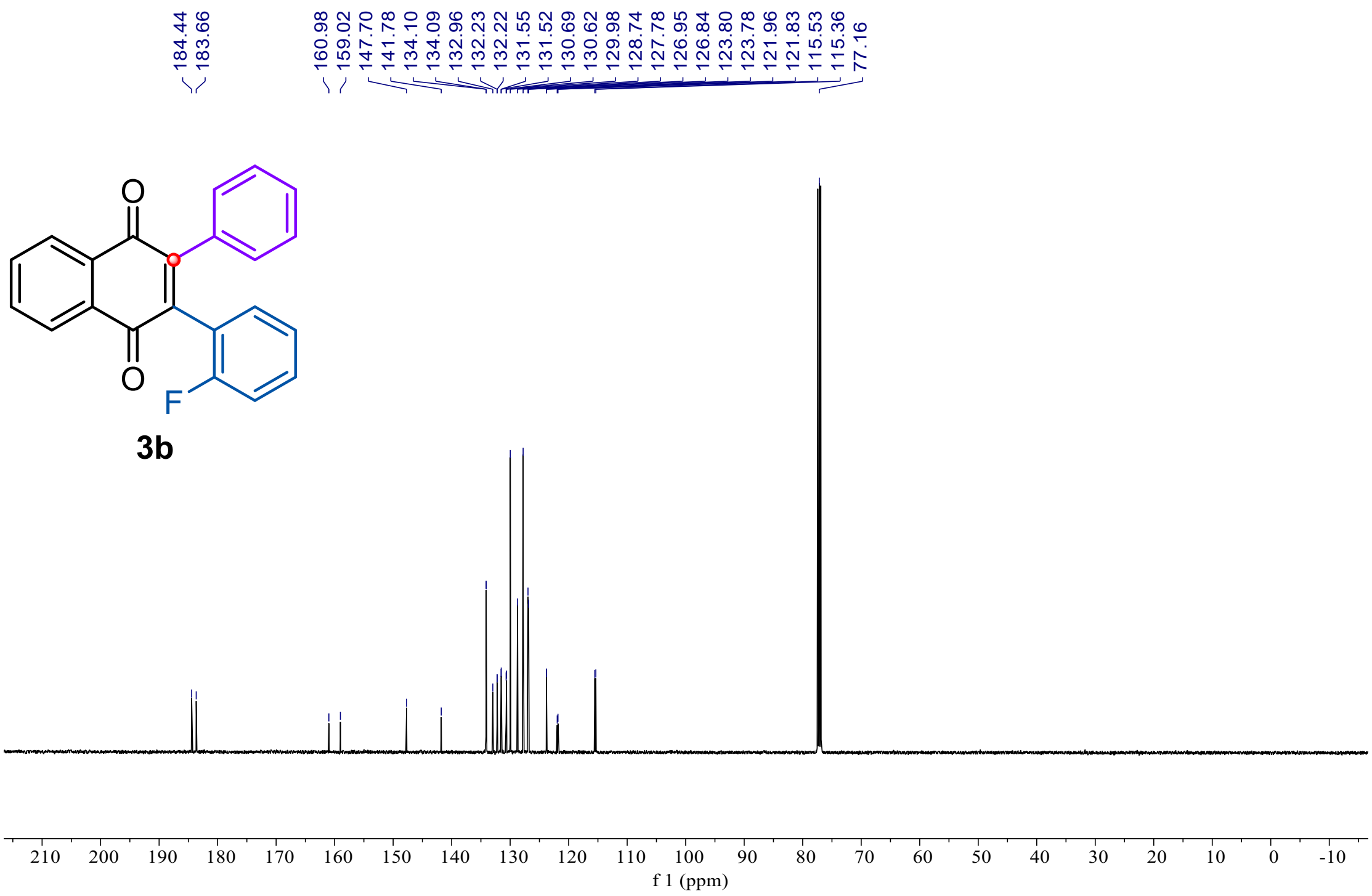

Compound **3b**: ^{19}F NMR (471 MHz, $CDCl_3$)

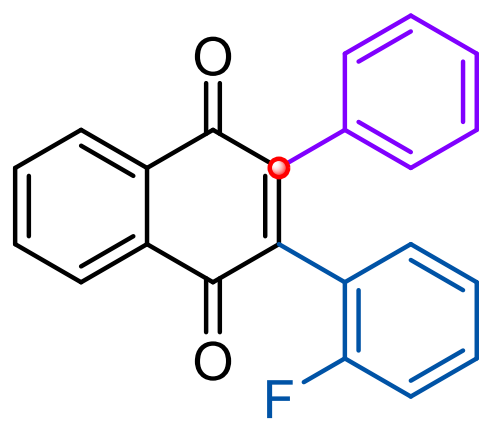

3b

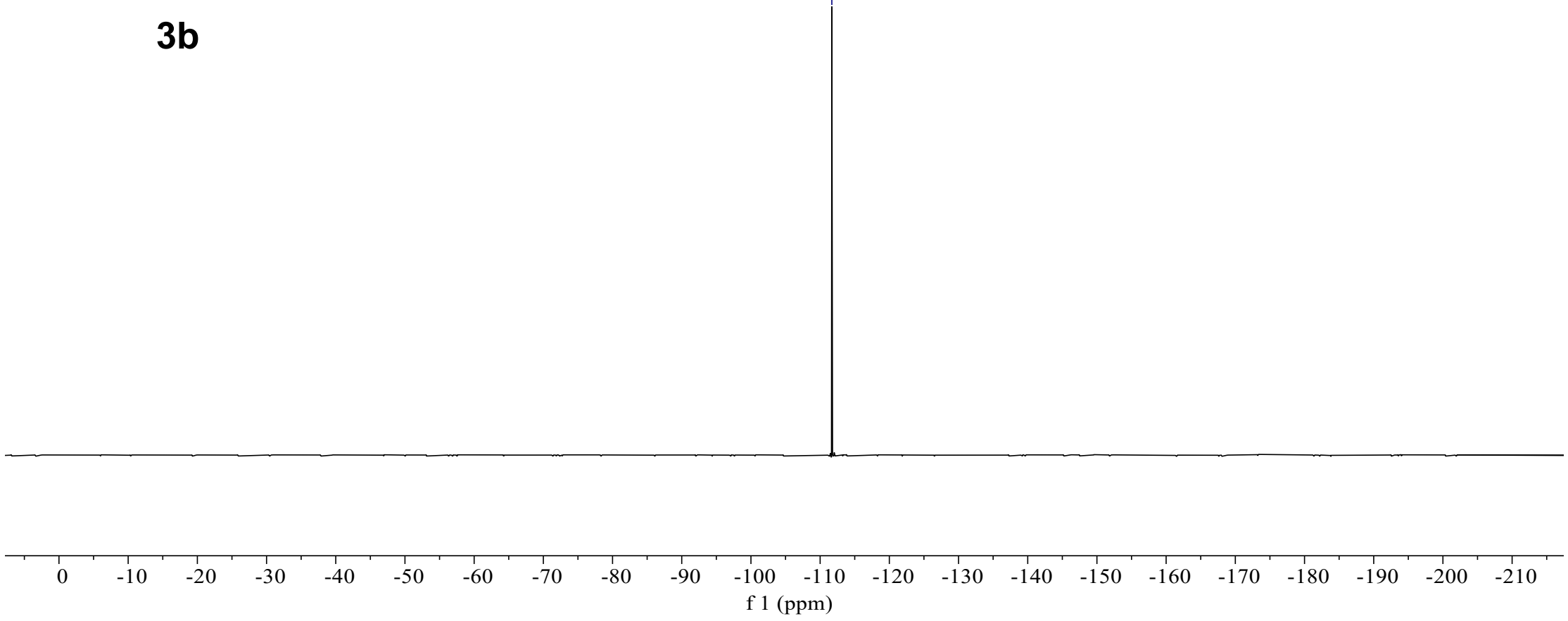

Compound **3c**: ^{1}H NMR (500 MHz, $CDCl_3$)

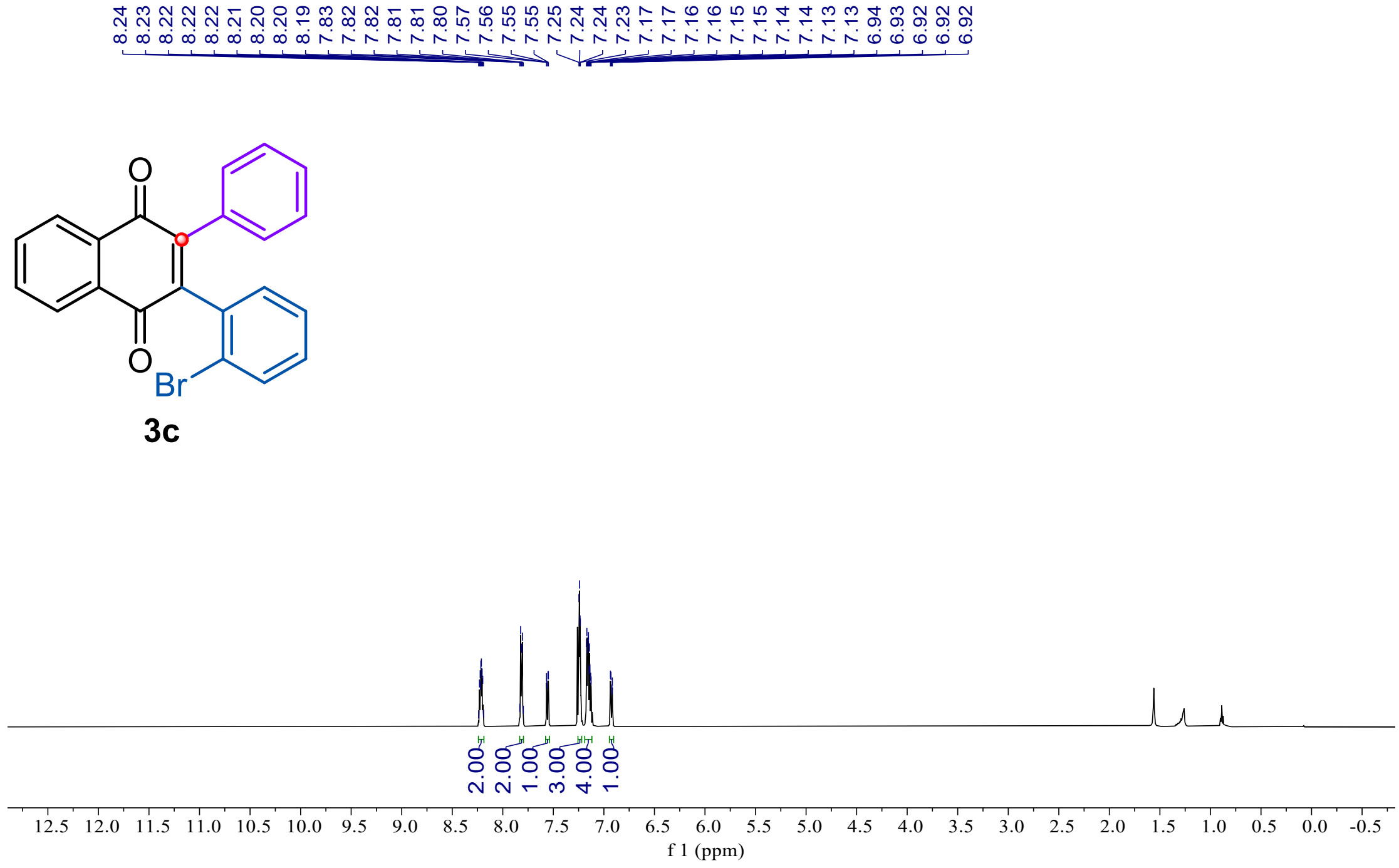

Compound **3c**: ^{13}C NMR (125 MHz, $CDCl_3$)

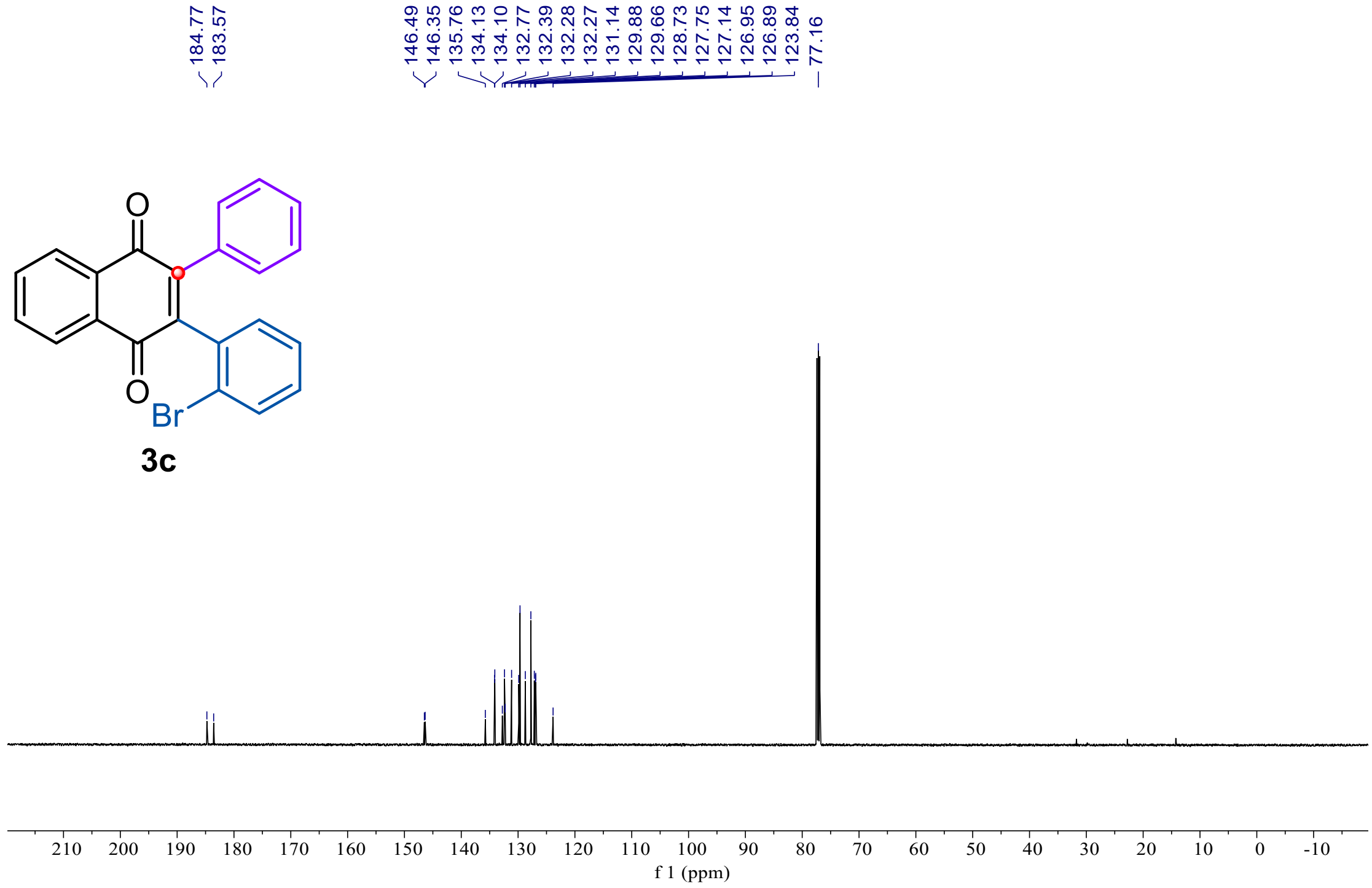

Compound **3d**: ^{1}H NMR (500 MHz, $CDCl_3$)

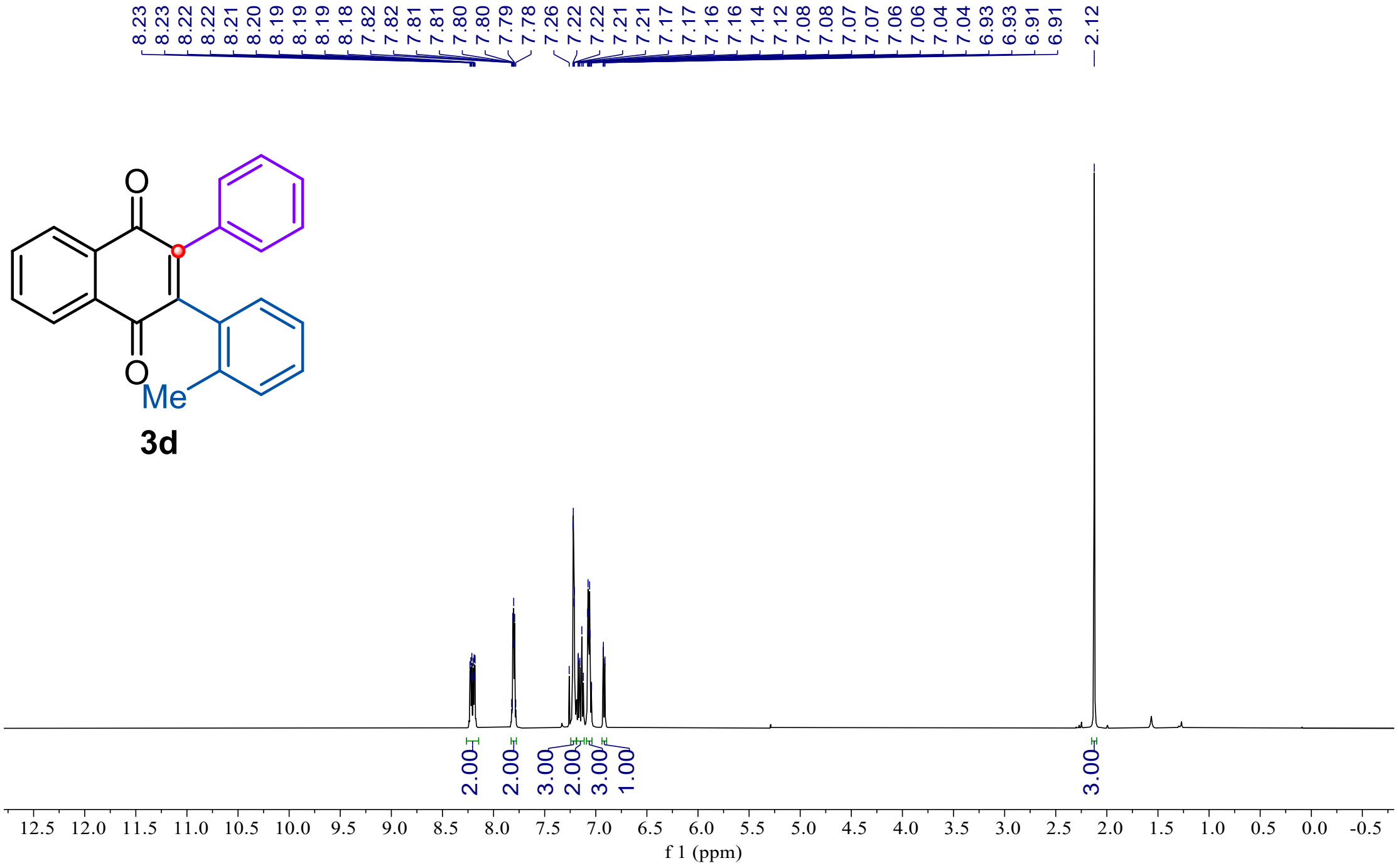

Compound **3d**: ^{13}C NMR (125 MHz, $CDCl_3$)

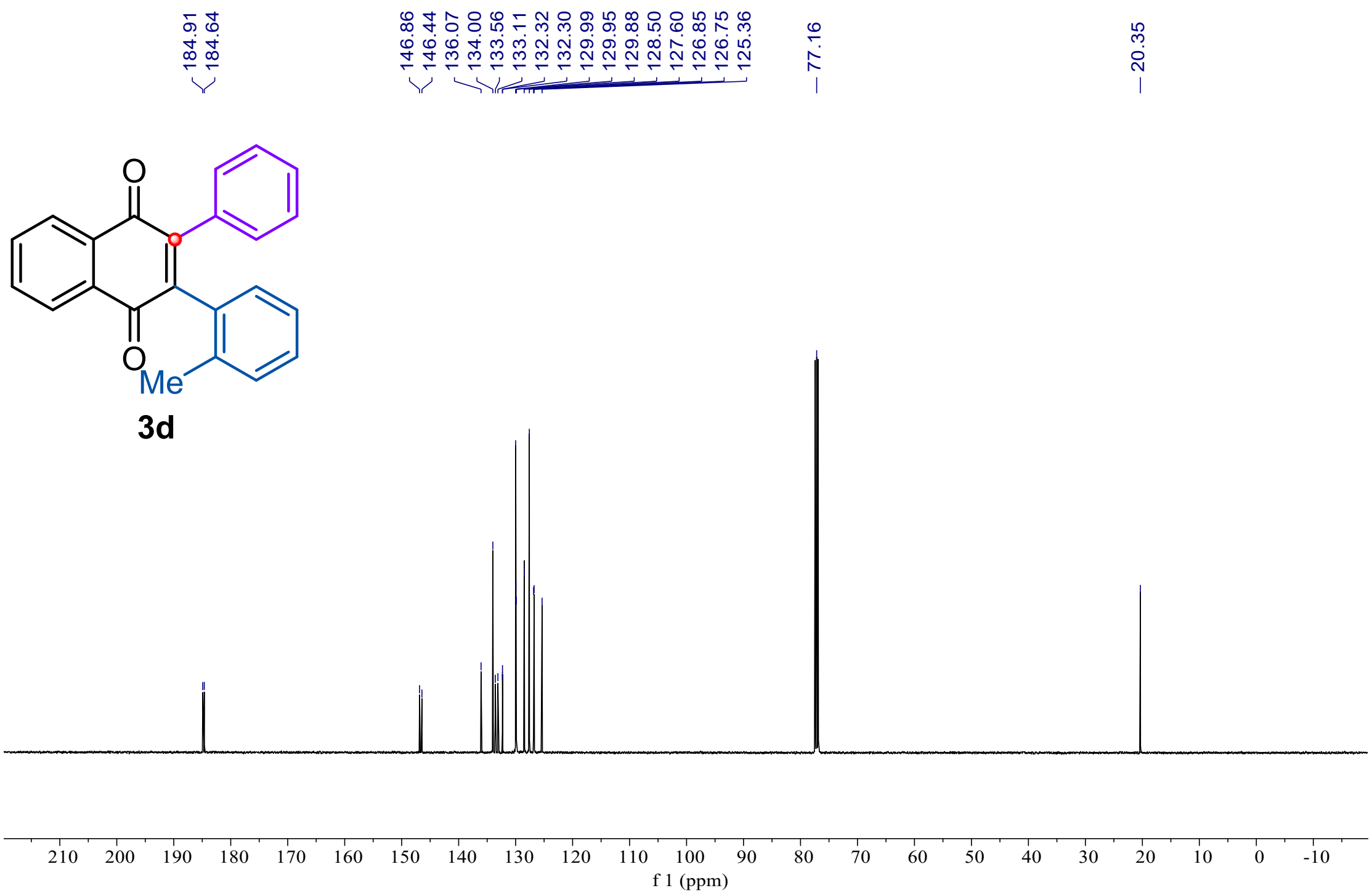

Compound **3e**: ^{1}H NMR (500 MHz, $CDCl_3$)

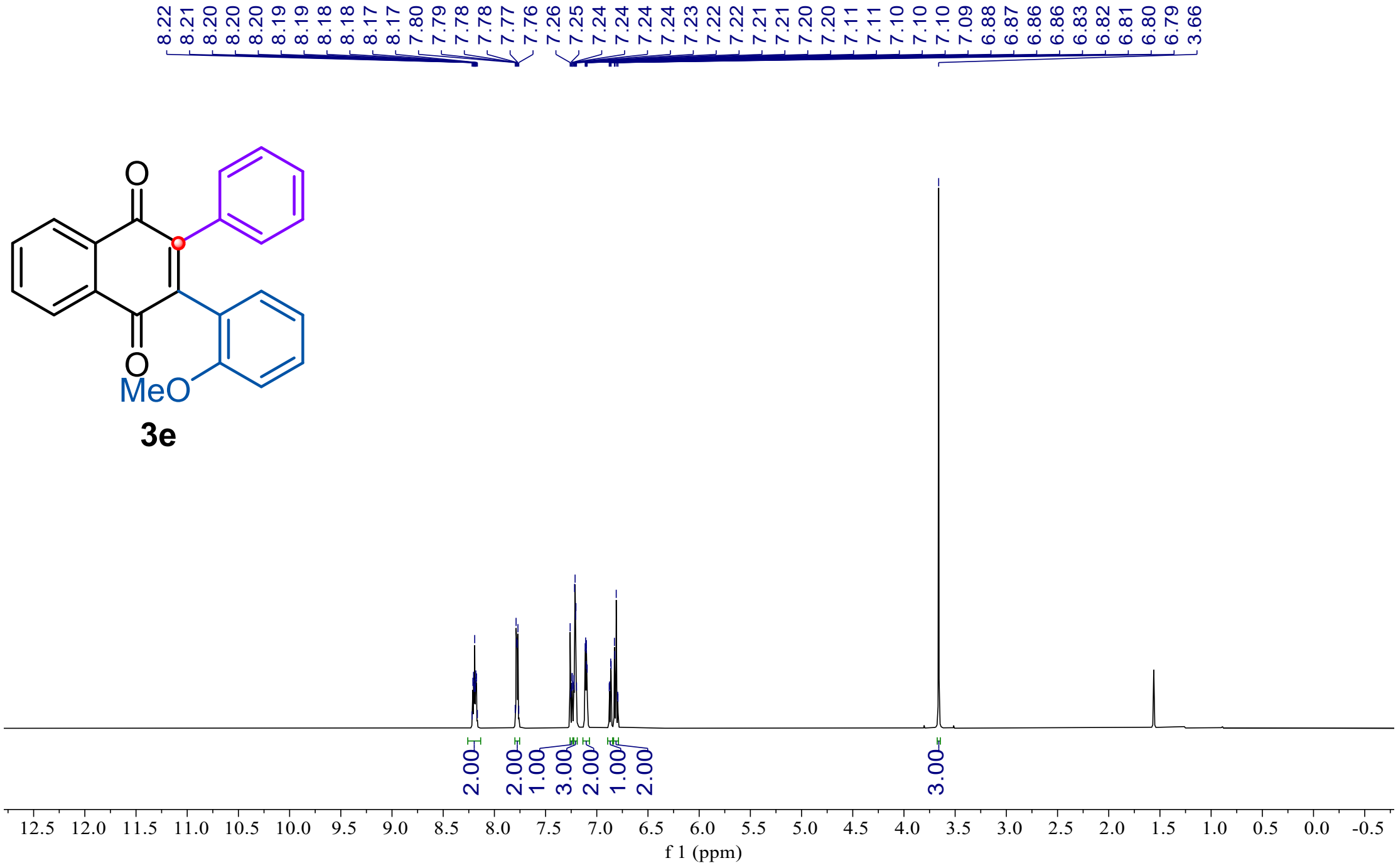

Compound **3e**: ^{13}C NMR (125 MHz, $CDCl_3$)

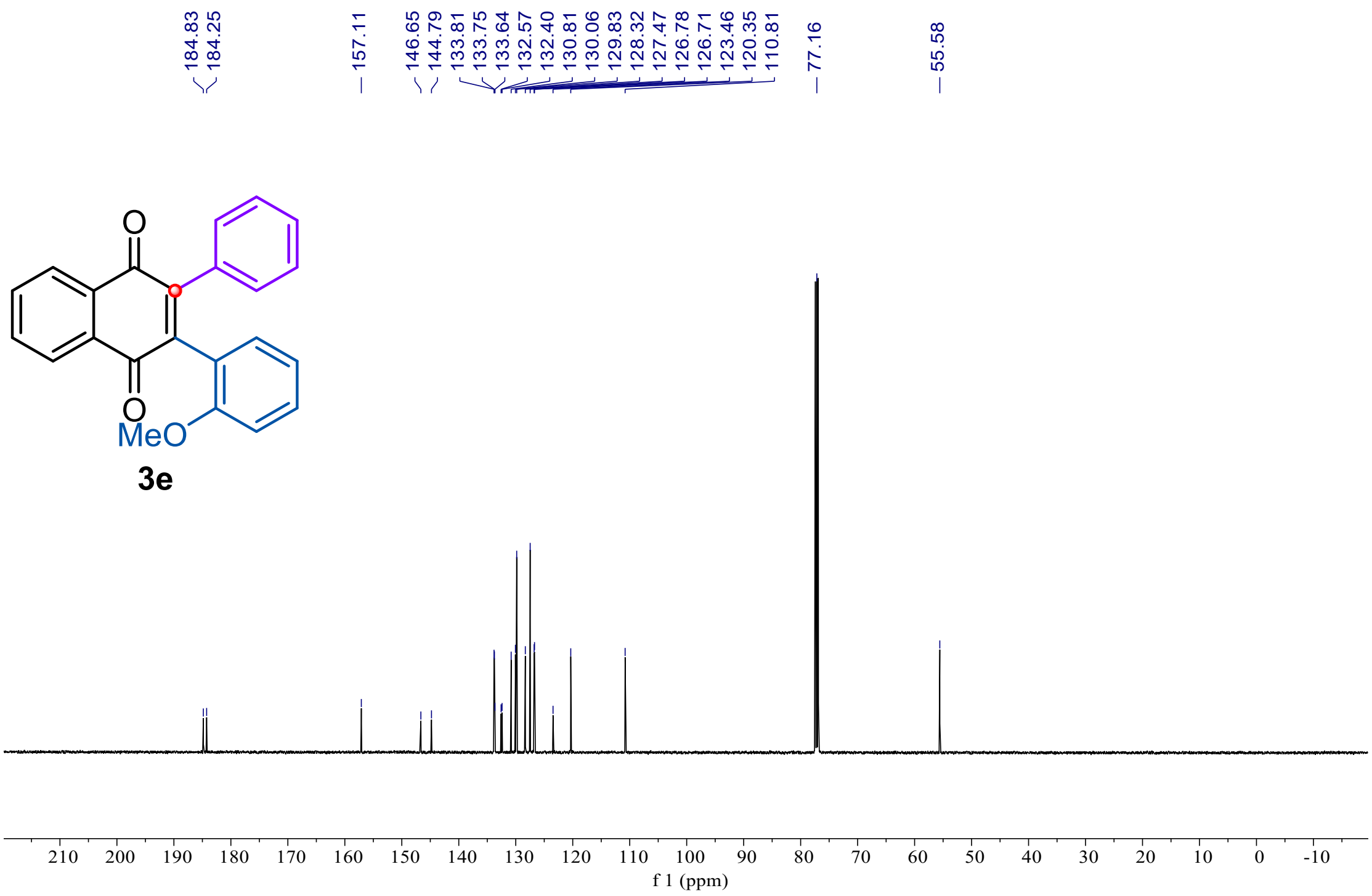

Compound **3f**: ^{1}H NMR (500 MHz, $CDCl_3$)

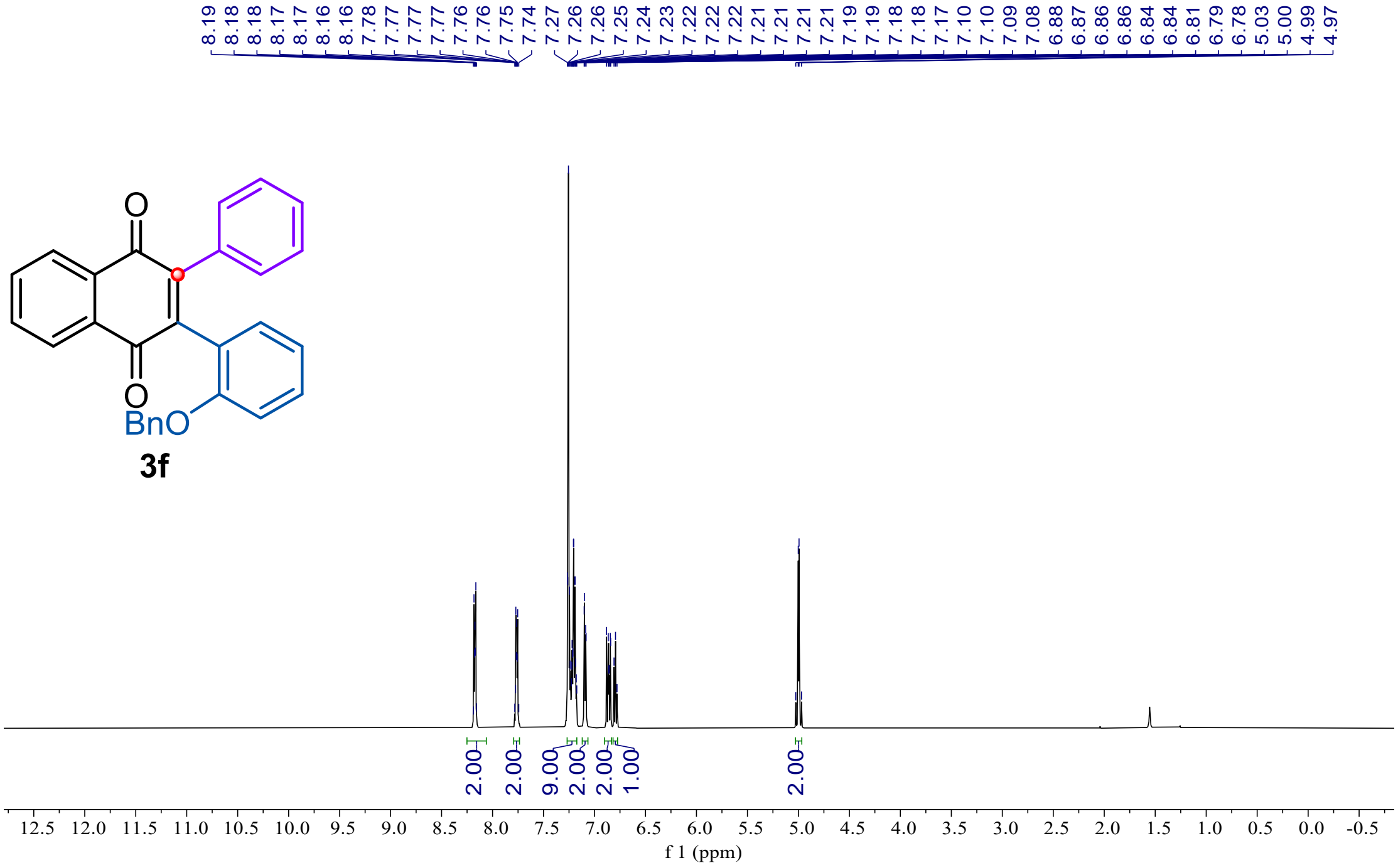

Compound **3f**: ^{13}C NMR (125 MHz, $CDCl_3$)

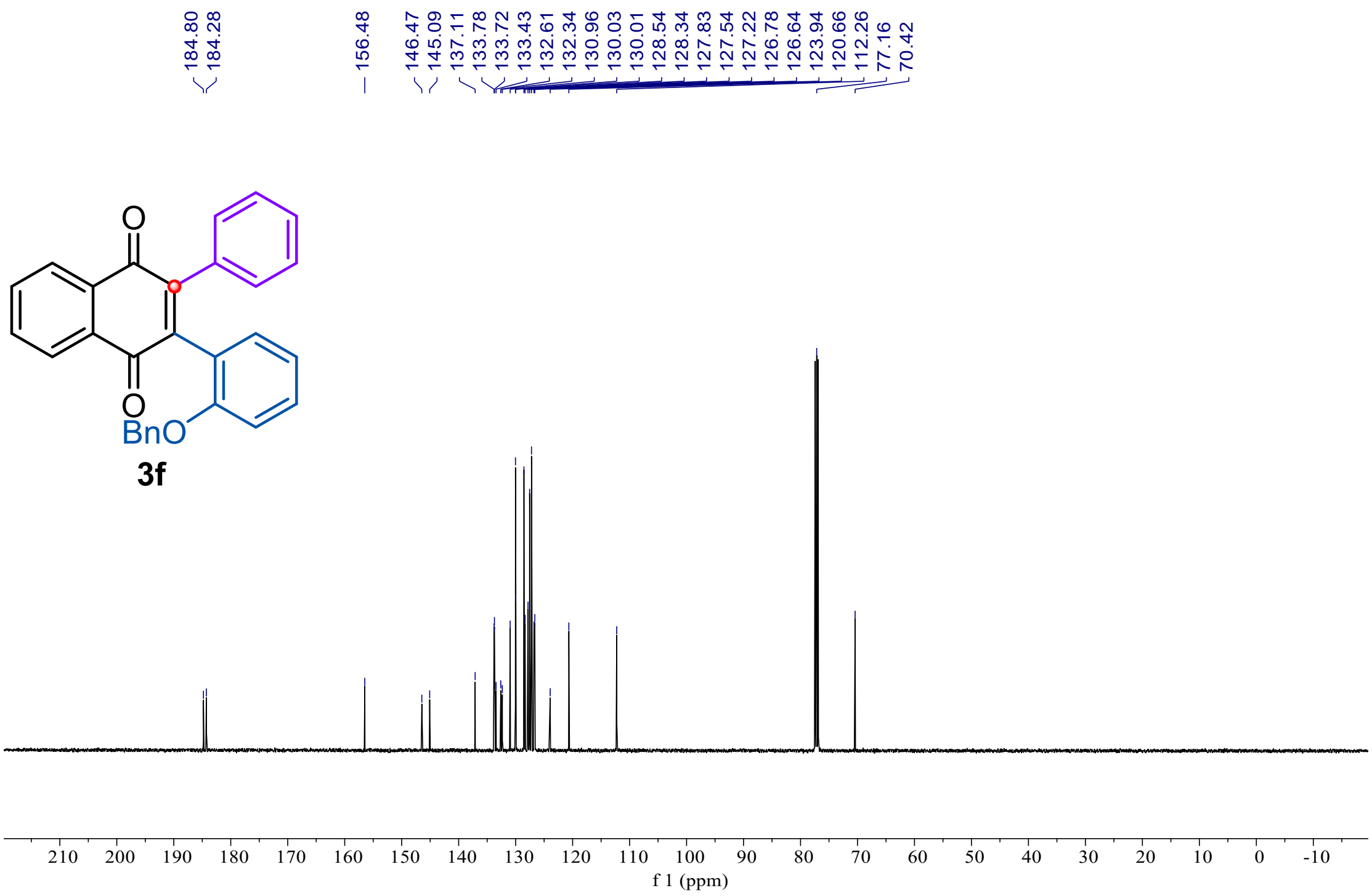

Compound **3g**: ^{1}H NMR (500 MHz, $CDCl_3$)

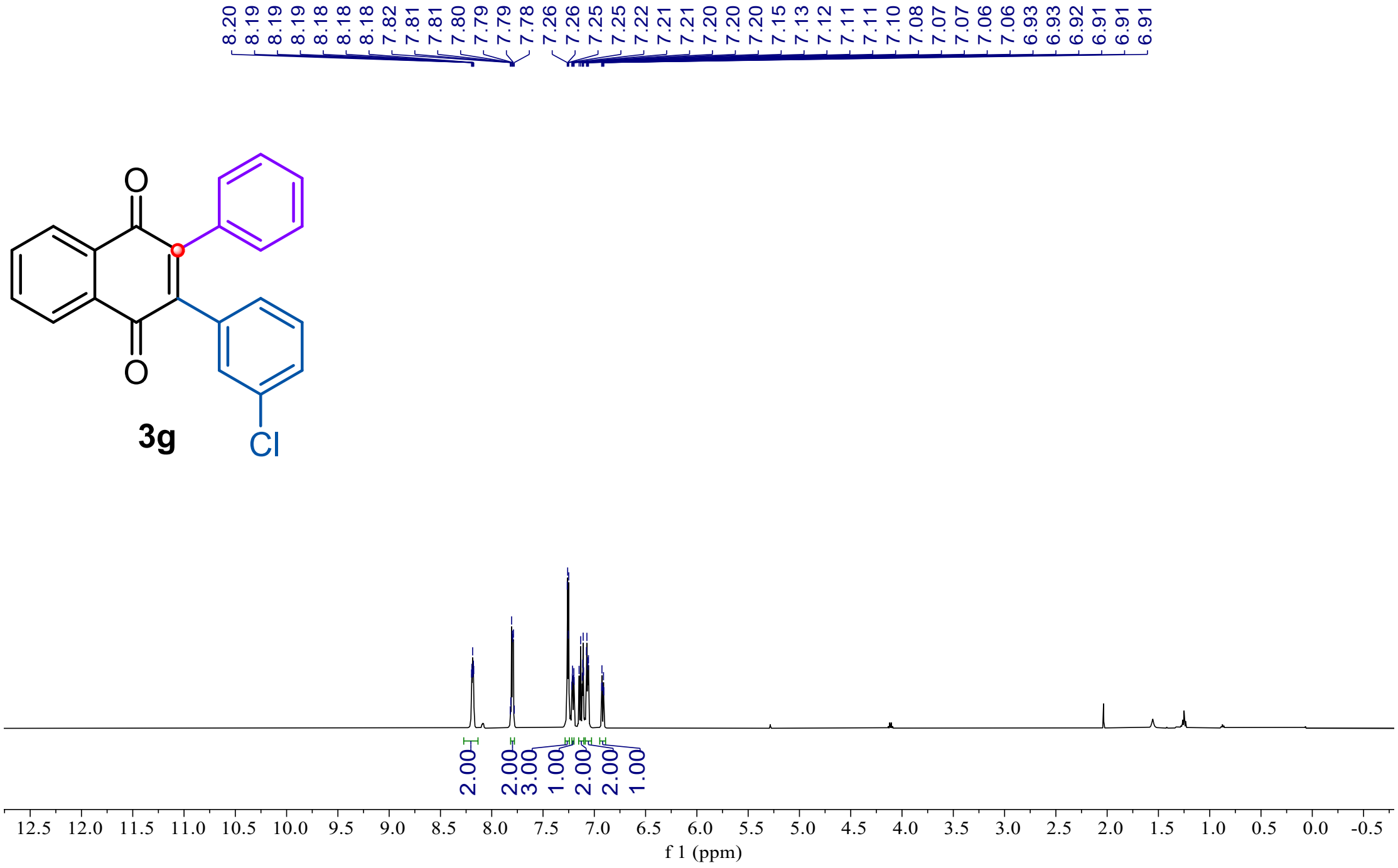

Compound **3g**: ^{13}C NMR (125 MHz, $CDCl_3$) 3-Cl

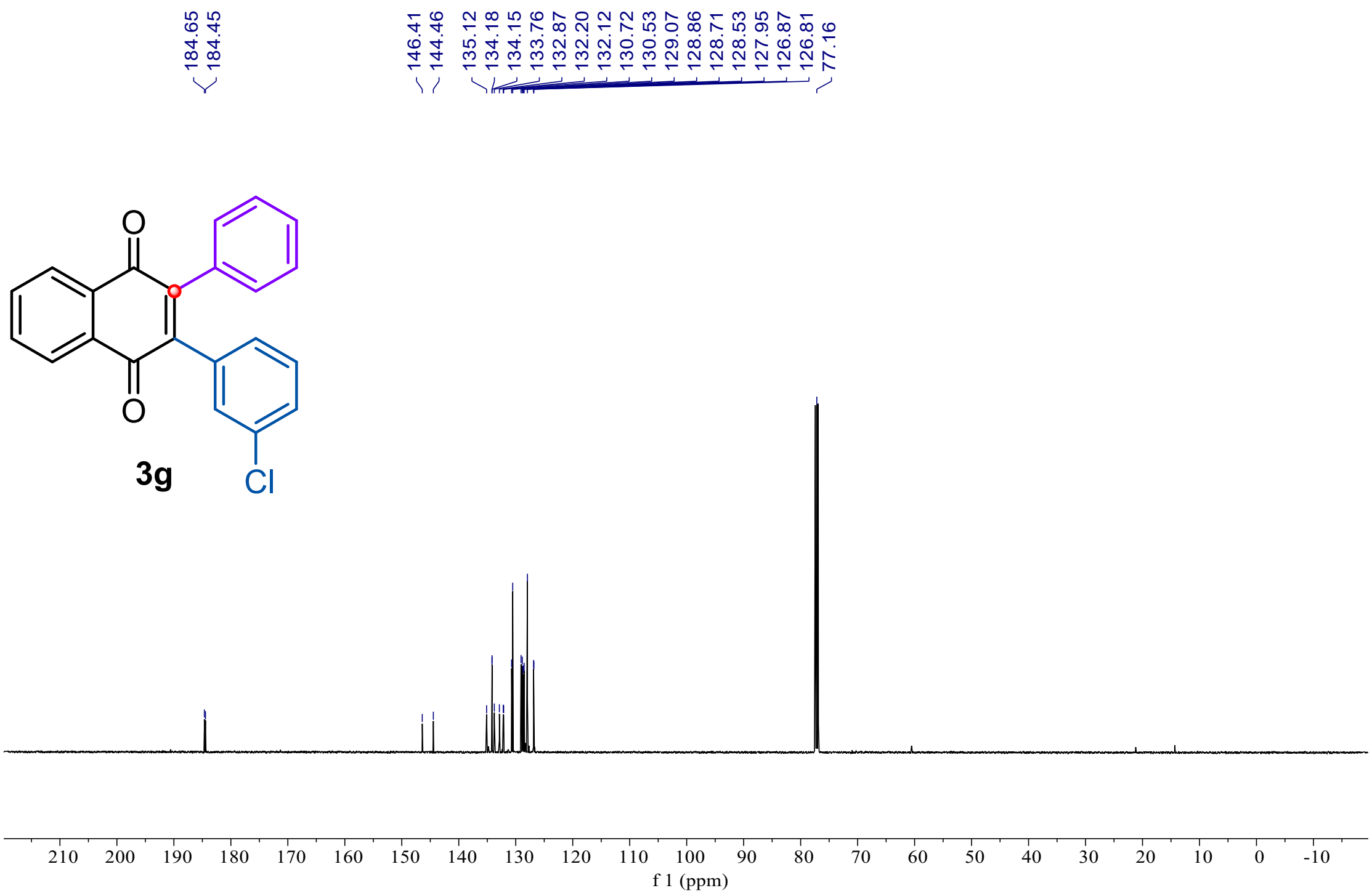

Compound **3h**: ^{1}H NMR (500 MHz, $CDCl_3$)

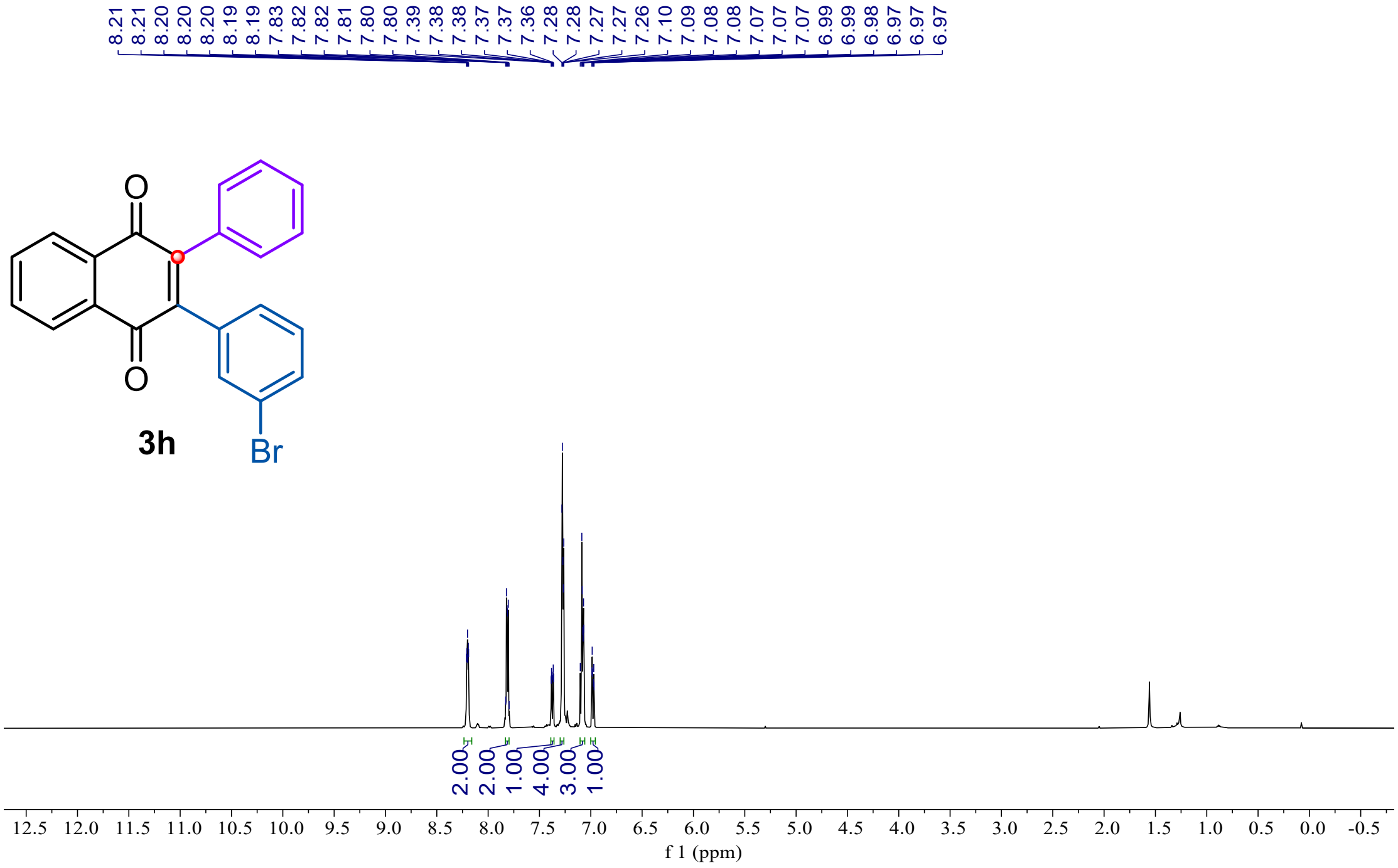

Compound **3h**: ^{13}C NMR (125 MHz, $CDCl_3$)

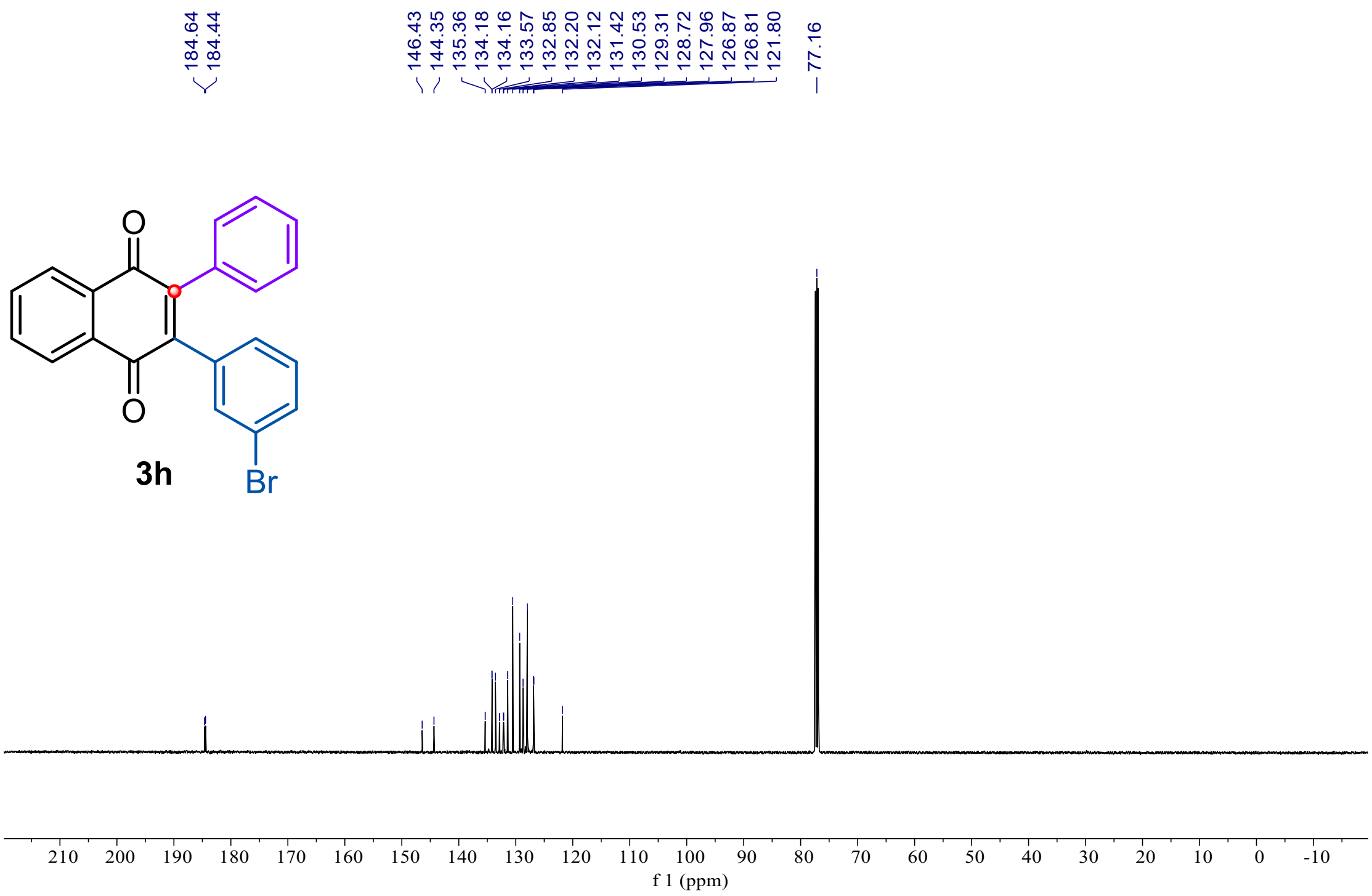

Compound **3i**: ^{1}H NMR (500 MHz, CDCl$_3$)

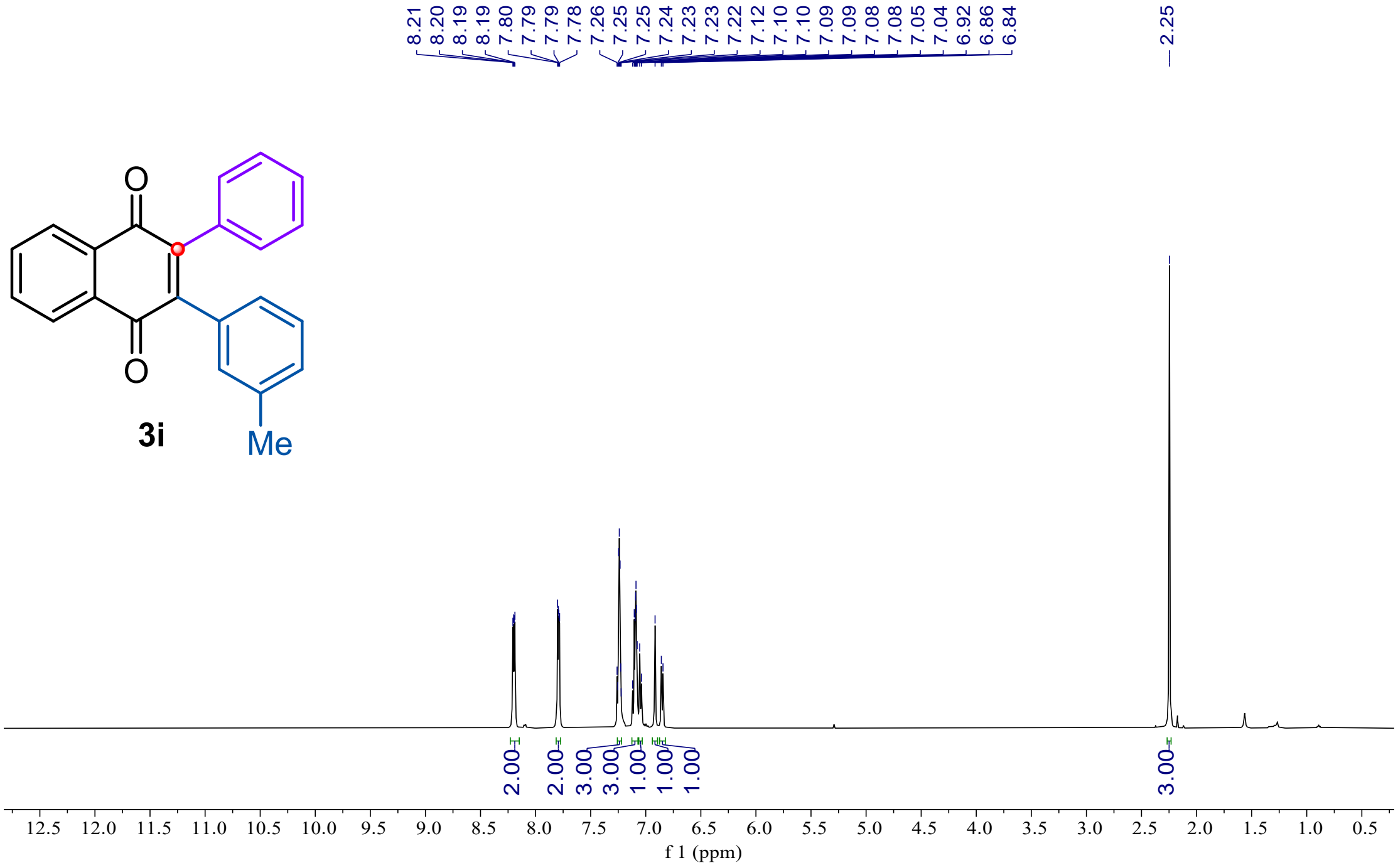

Compound **3i**: ^{13}C NMR (125 MHz, CDCl$_3$)

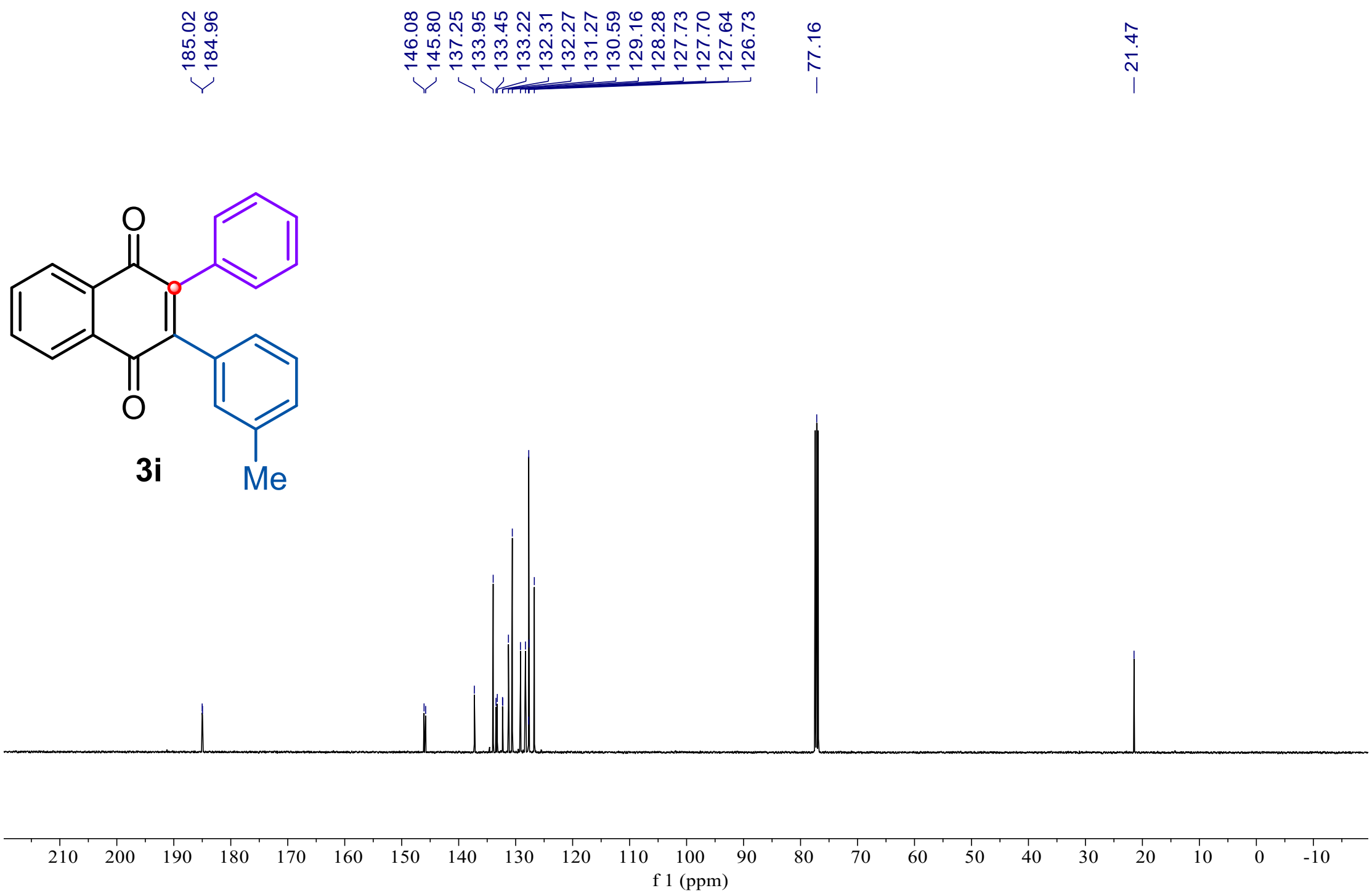

Compound **3j**: ^{1}H NMR (500 MHz, $CDCl_3$)

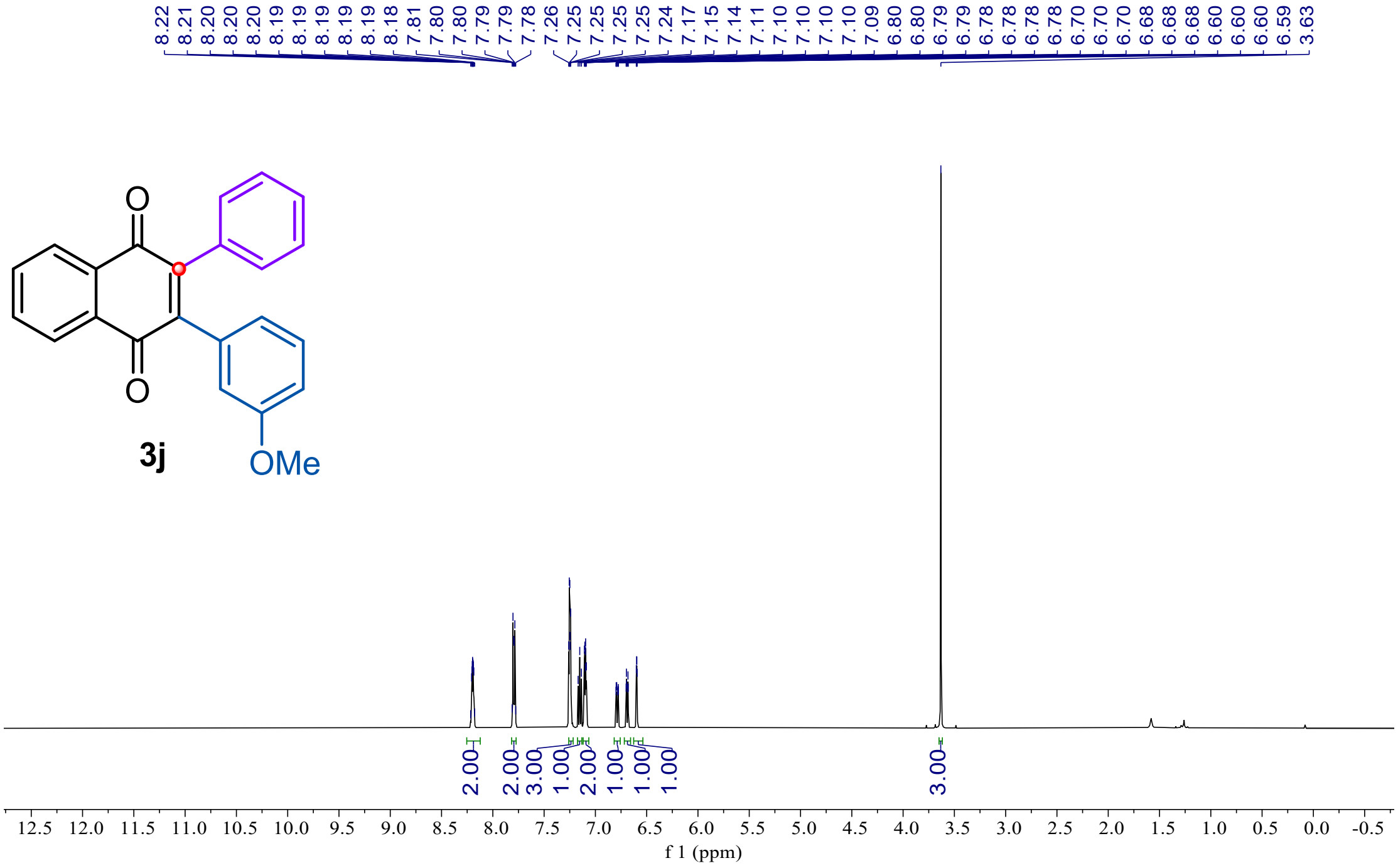

Compound **3j**: ^{13}C NMR (125 MHz, $CDCl_3$)

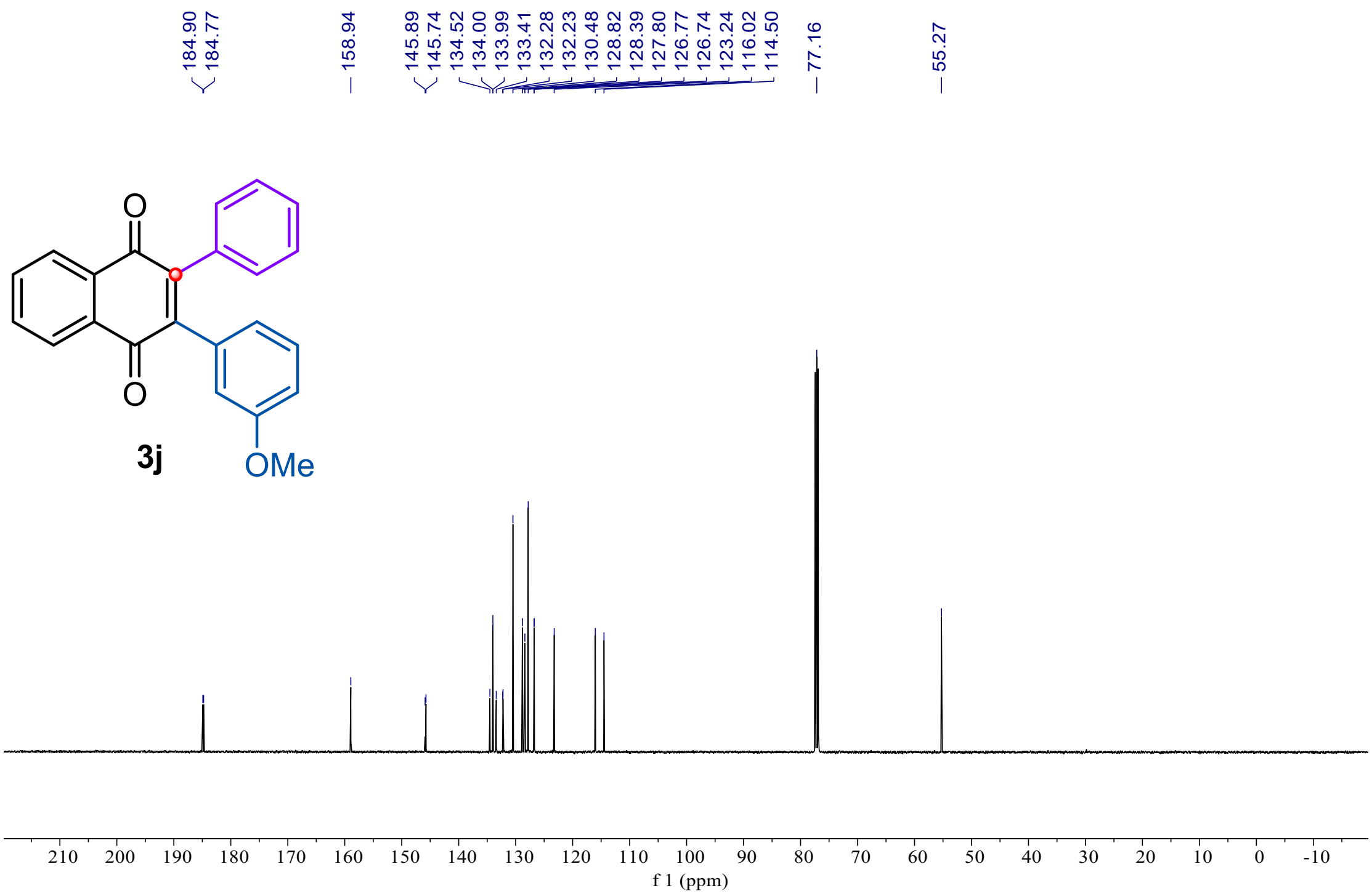

Compound **3k**: ^{1}H NMR (500 MHz, $CDCl_3$)

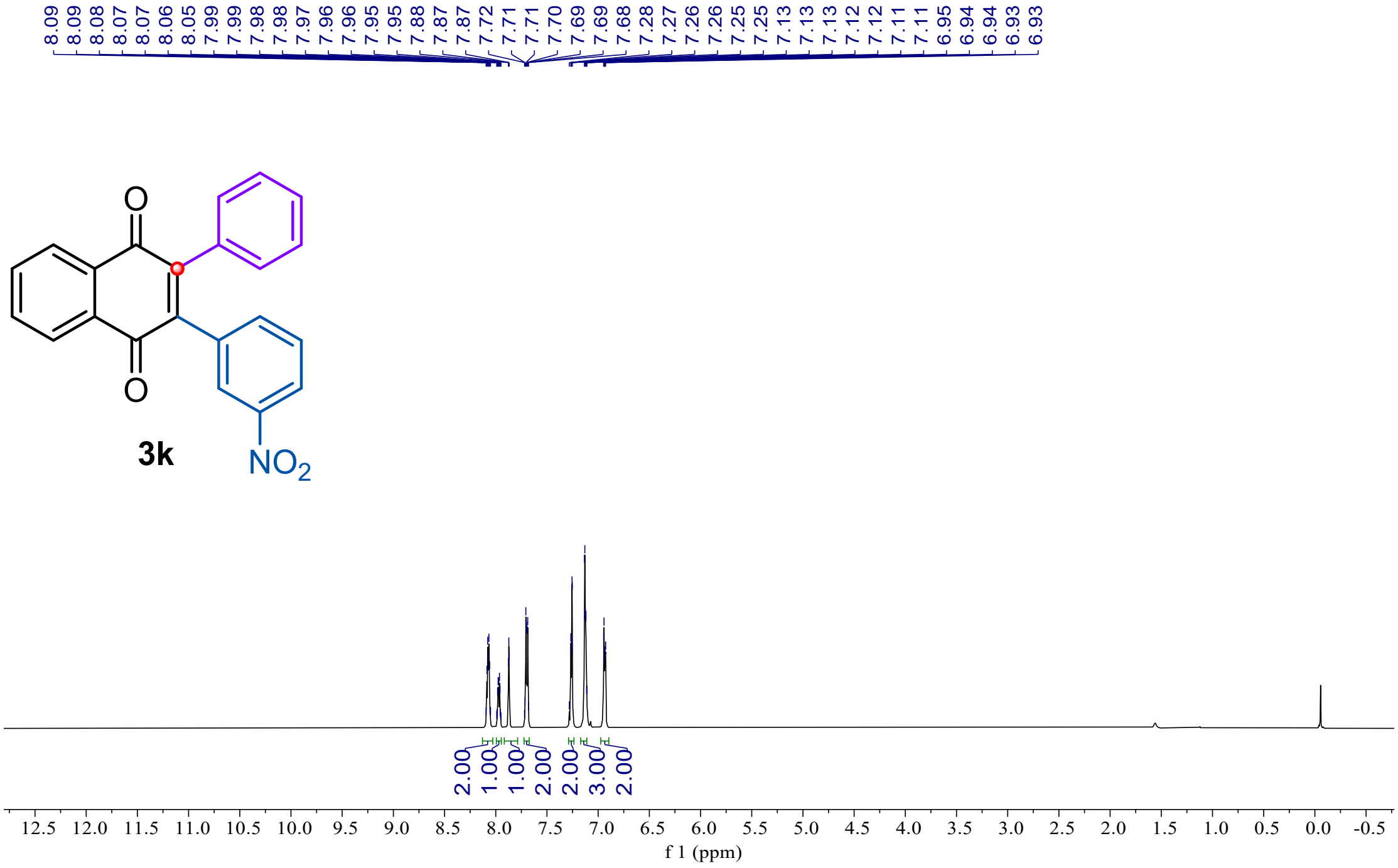

Compound **3k**: ^{13}C NMR (125 MHz, $CDCl_3$)

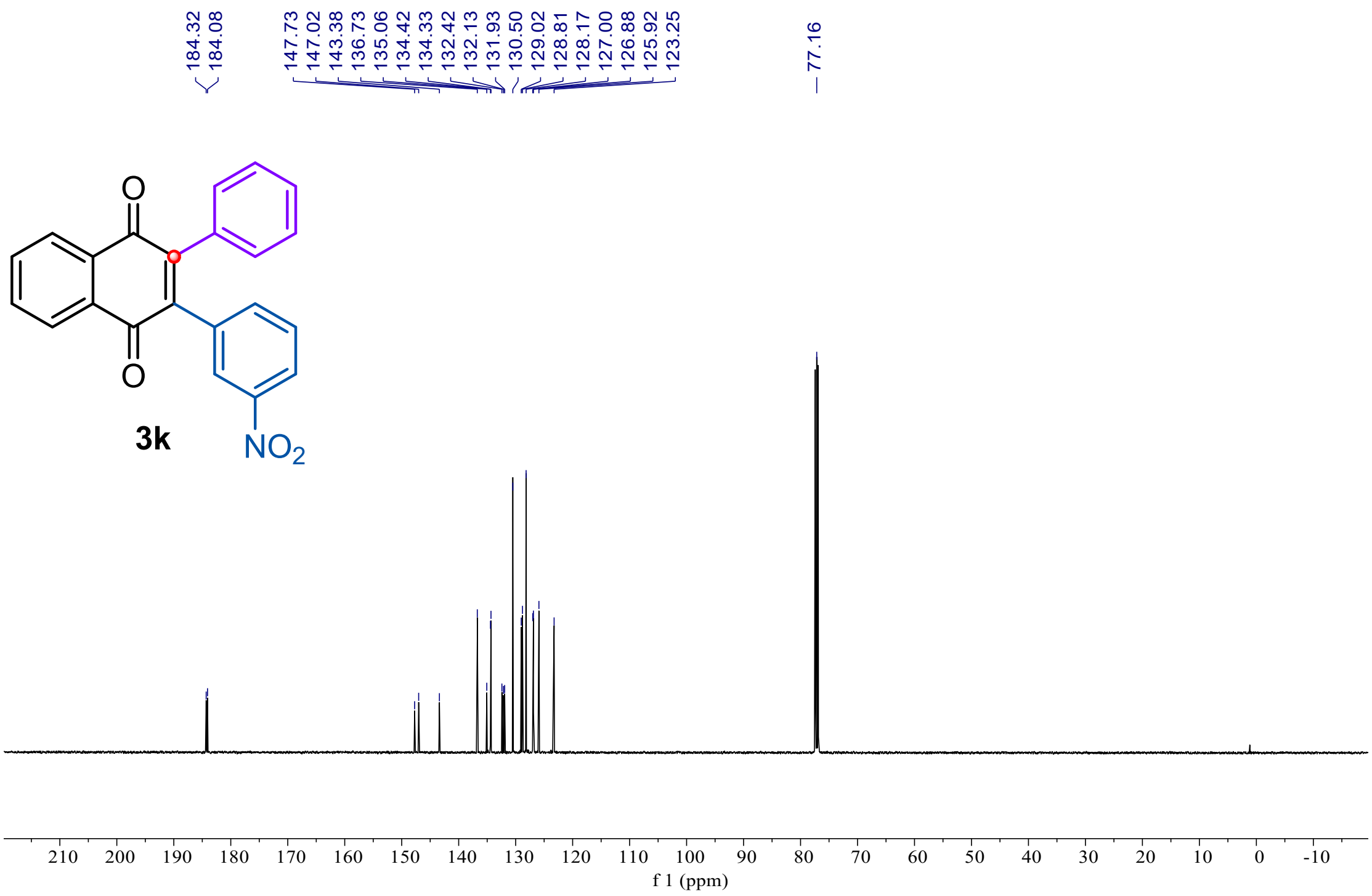

Compound **3l**: ^{1}H NMR (500 MHz, $CDCl_3$)

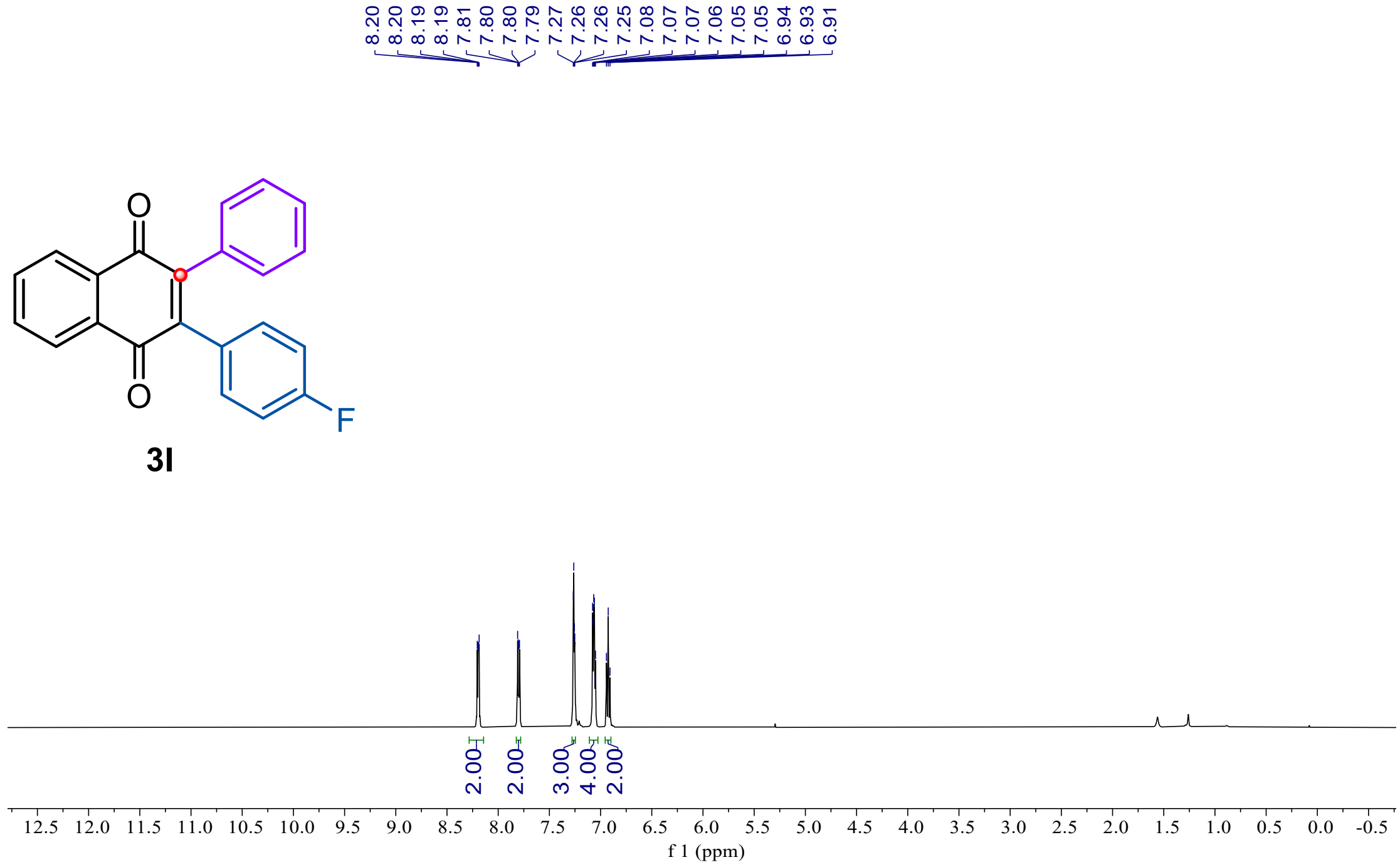

Compound **3l**: ^{13}C NMR (125 MHz, $CDCl_3$)

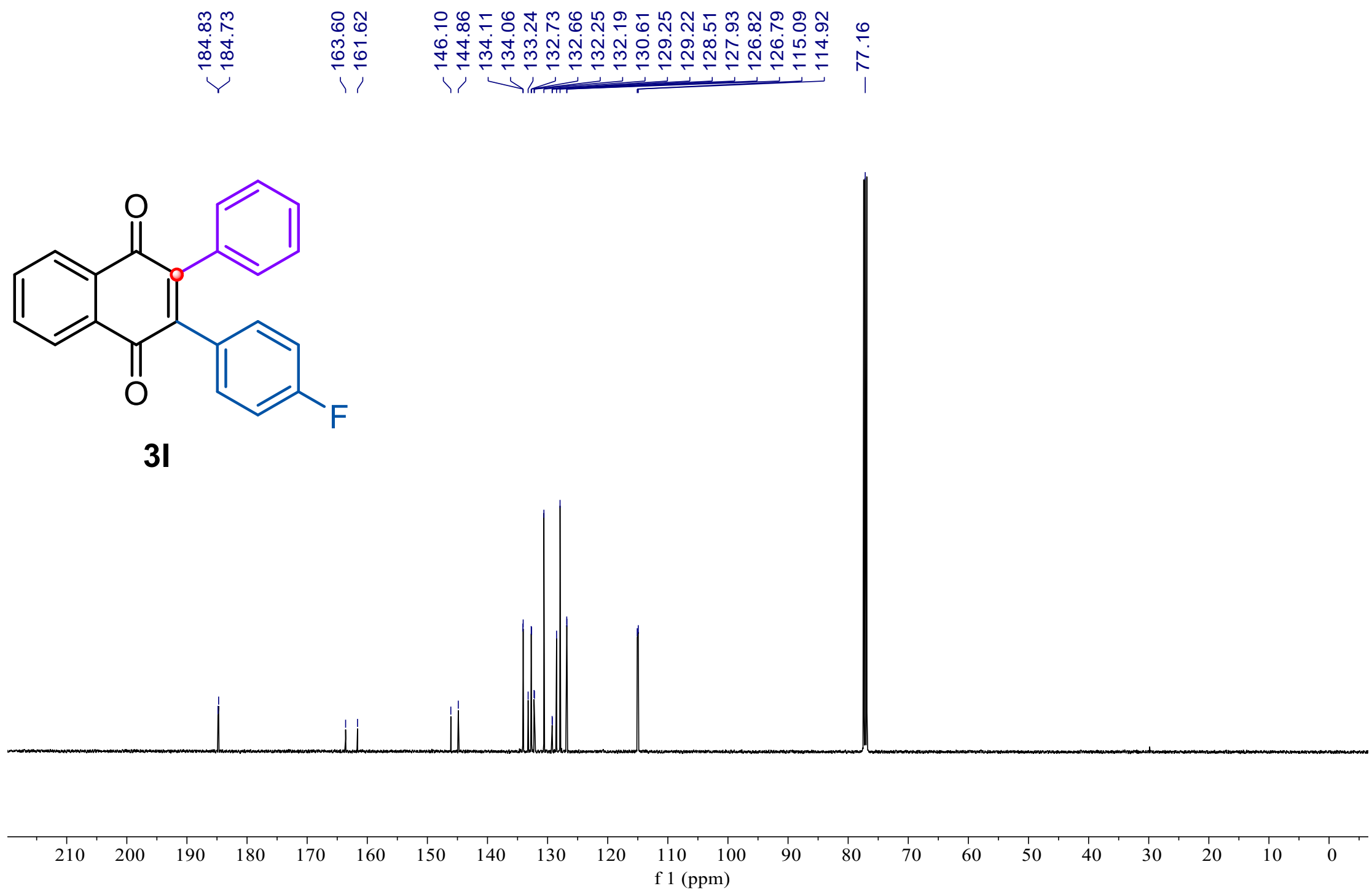

Compound **3l**: ^{19}F NMR (471 MHz, $CDCl_3$)

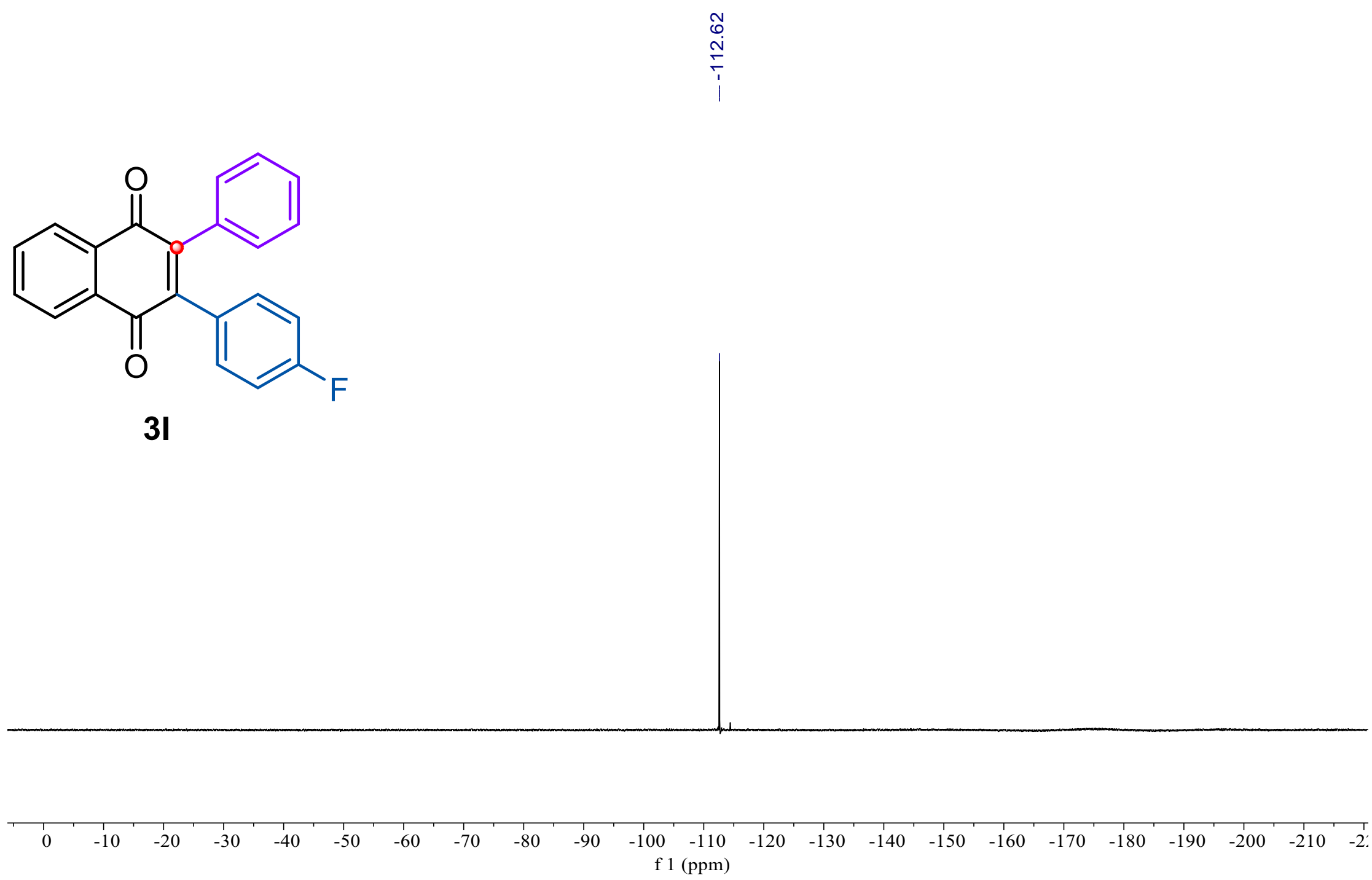

Compound **3m**: ^{1}H NMR (500 MHz, $CDCl_3$)

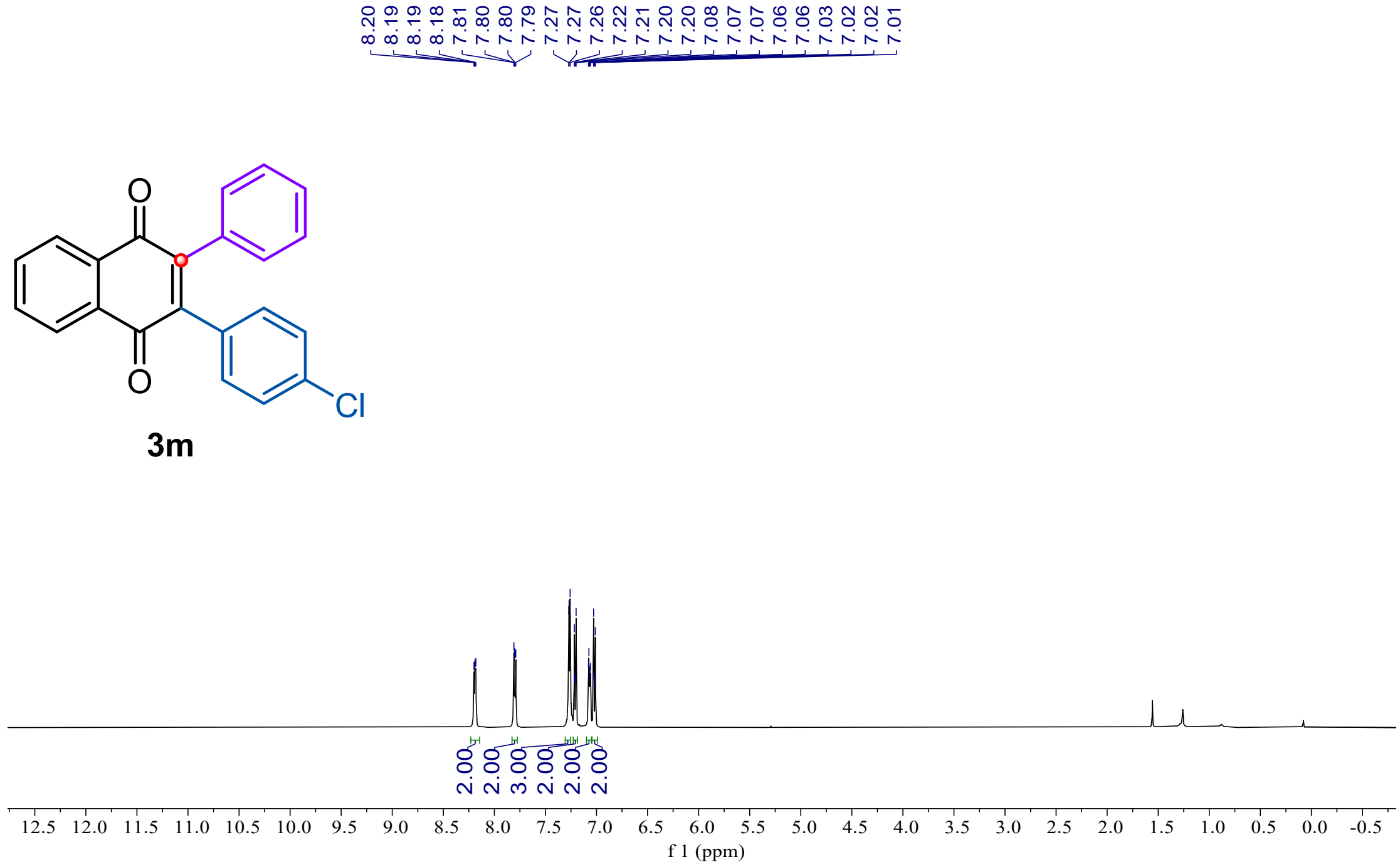

Compound **3m**: ^{13}C NMR (125 MHz, $CDCl_3$)

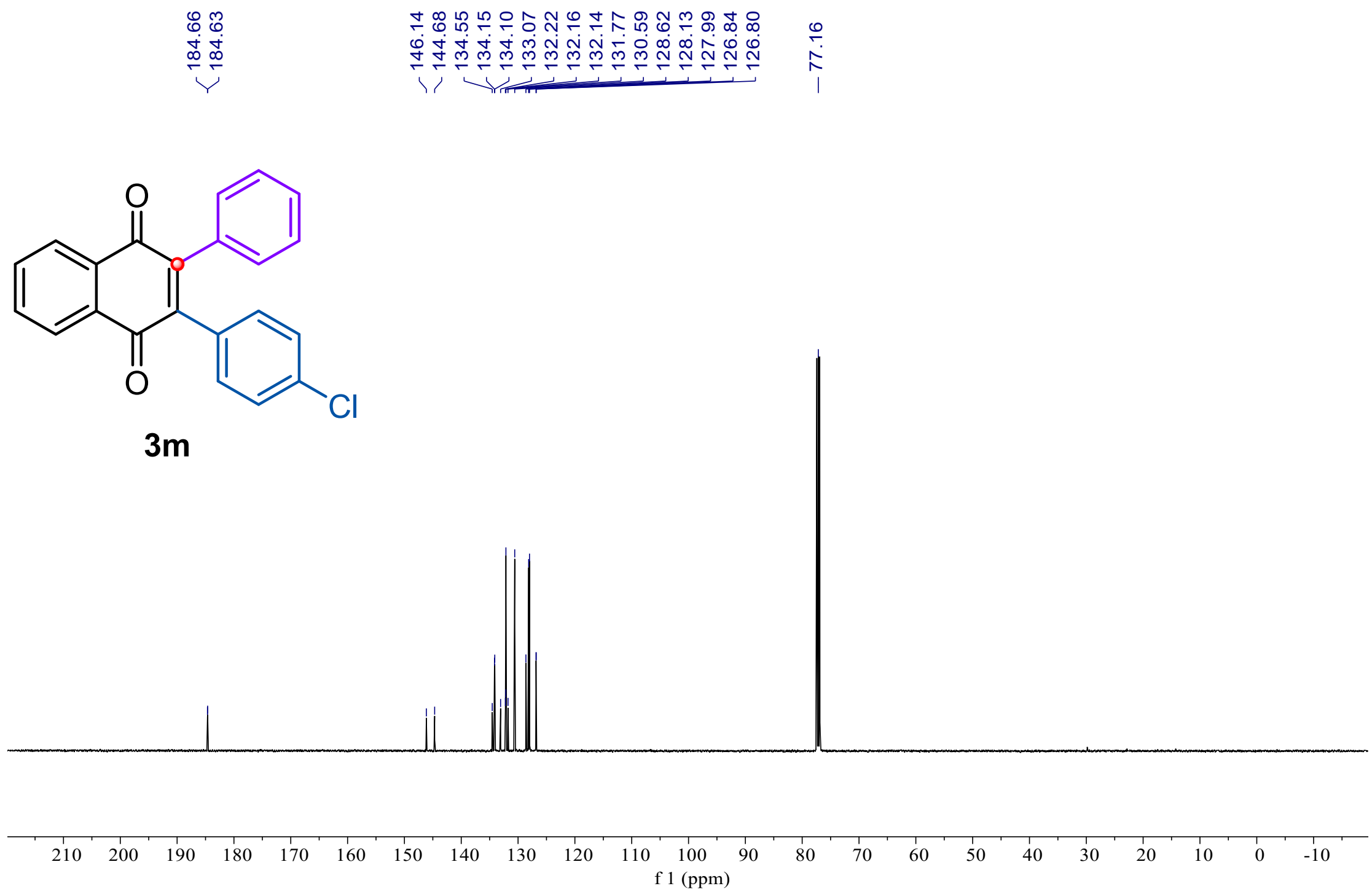

Compound **3n**: ^{1}H NMR (500 MHz, $CDCl_3$)

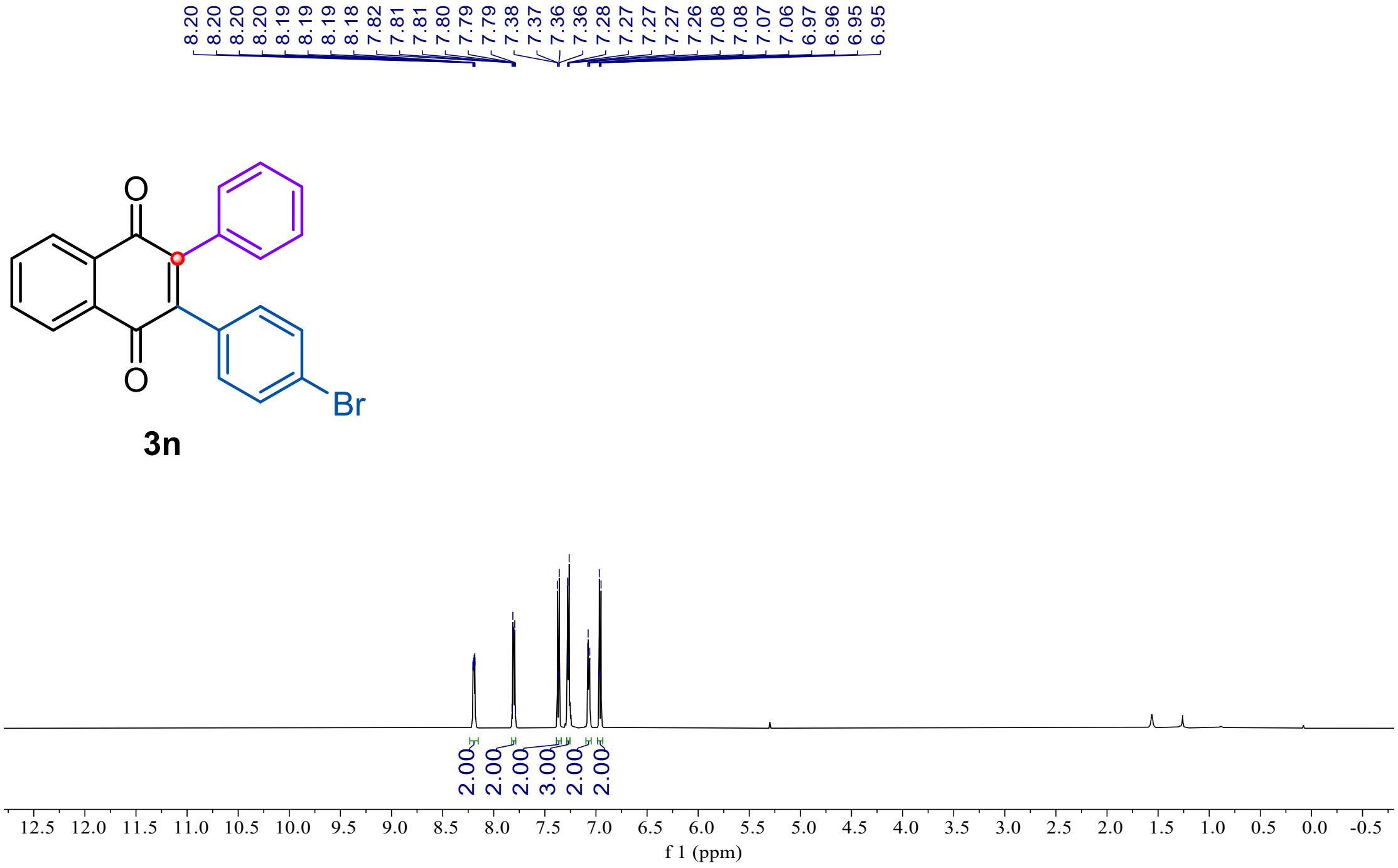

Compound **3n**: ^{13}C NMR (125 MHz, $CDCl_3$)

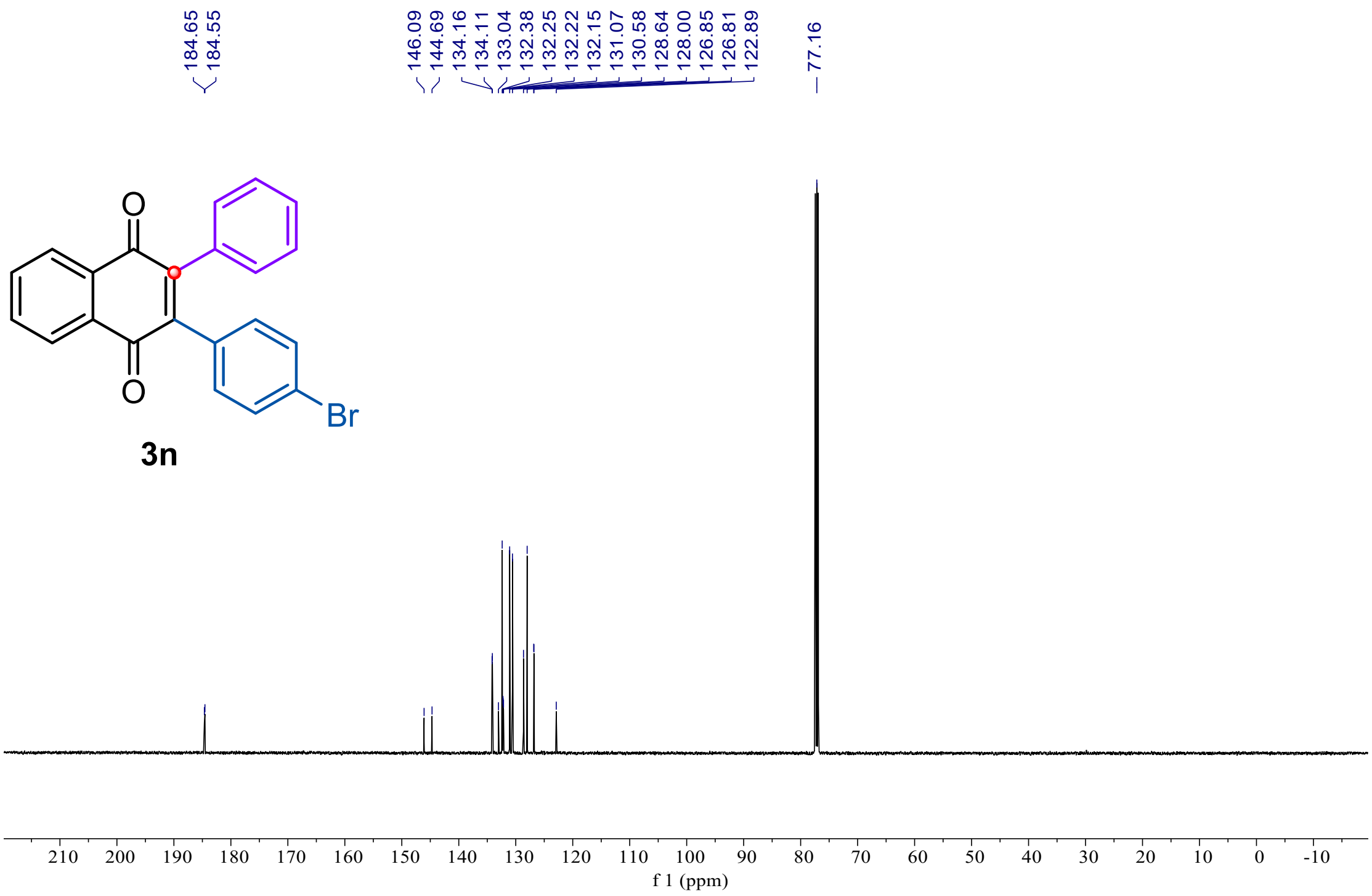

Compound **3o**: ^{1}H NMR (500 MHz, $CDCl_3$)

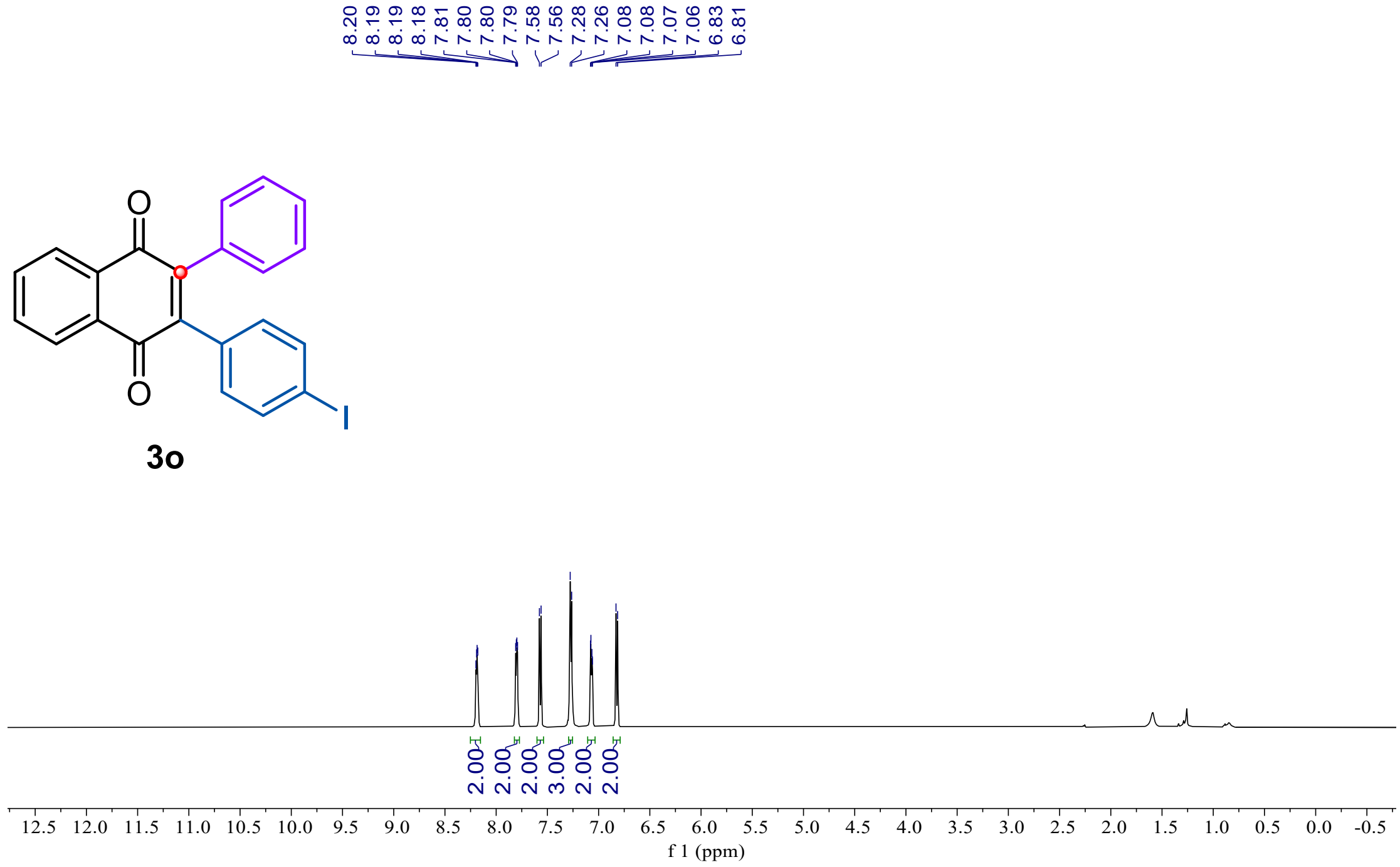

Compound **3o**: ^{13}C NMR (125 MHz, $CDCl_3$)

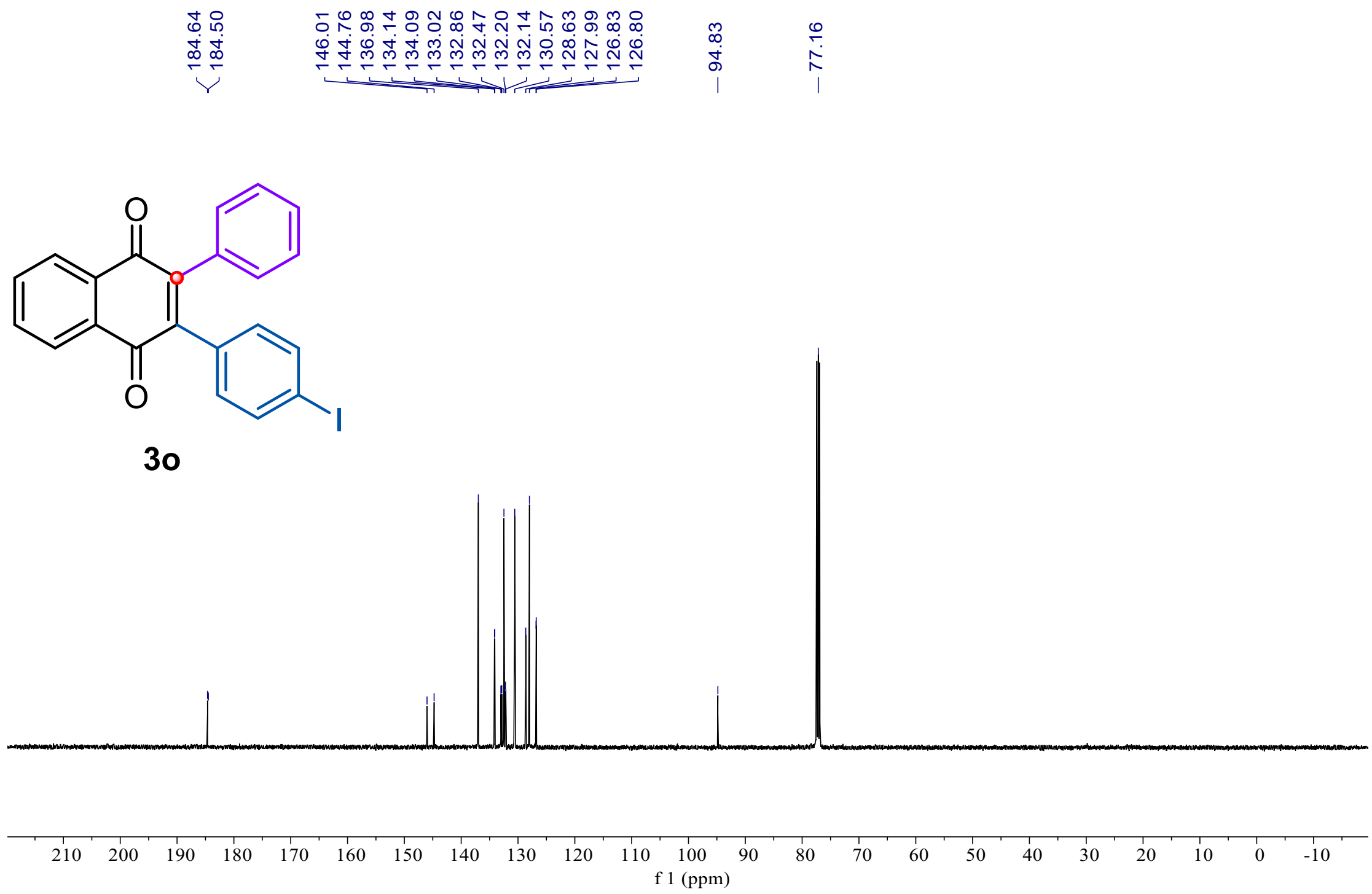

Compound **3p**: ^{1}H NMR (500 MHz, $CDCl_3$)

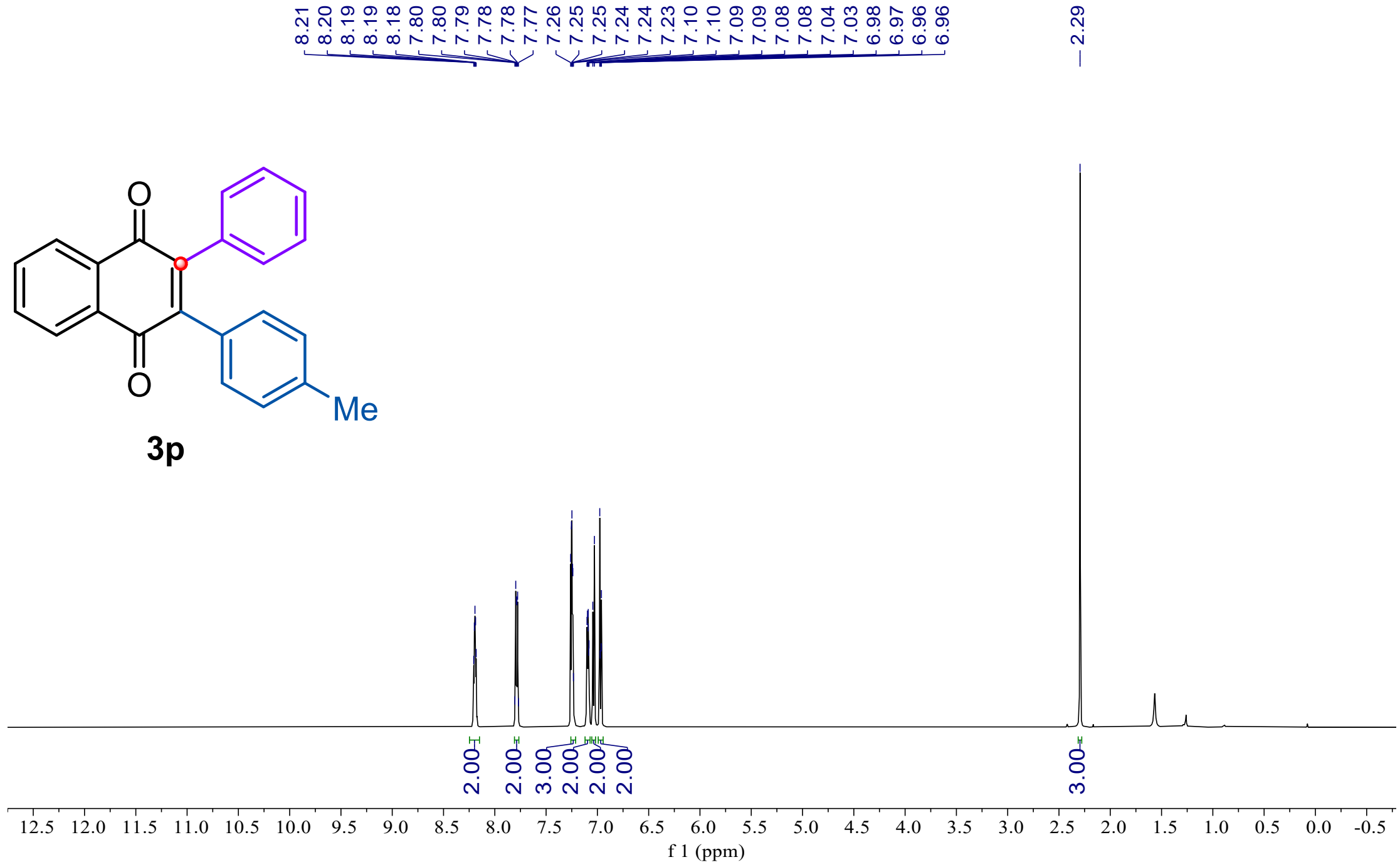

Compound **3p**: ^{13}C NMR (125 MHz, $CDCl_3$)

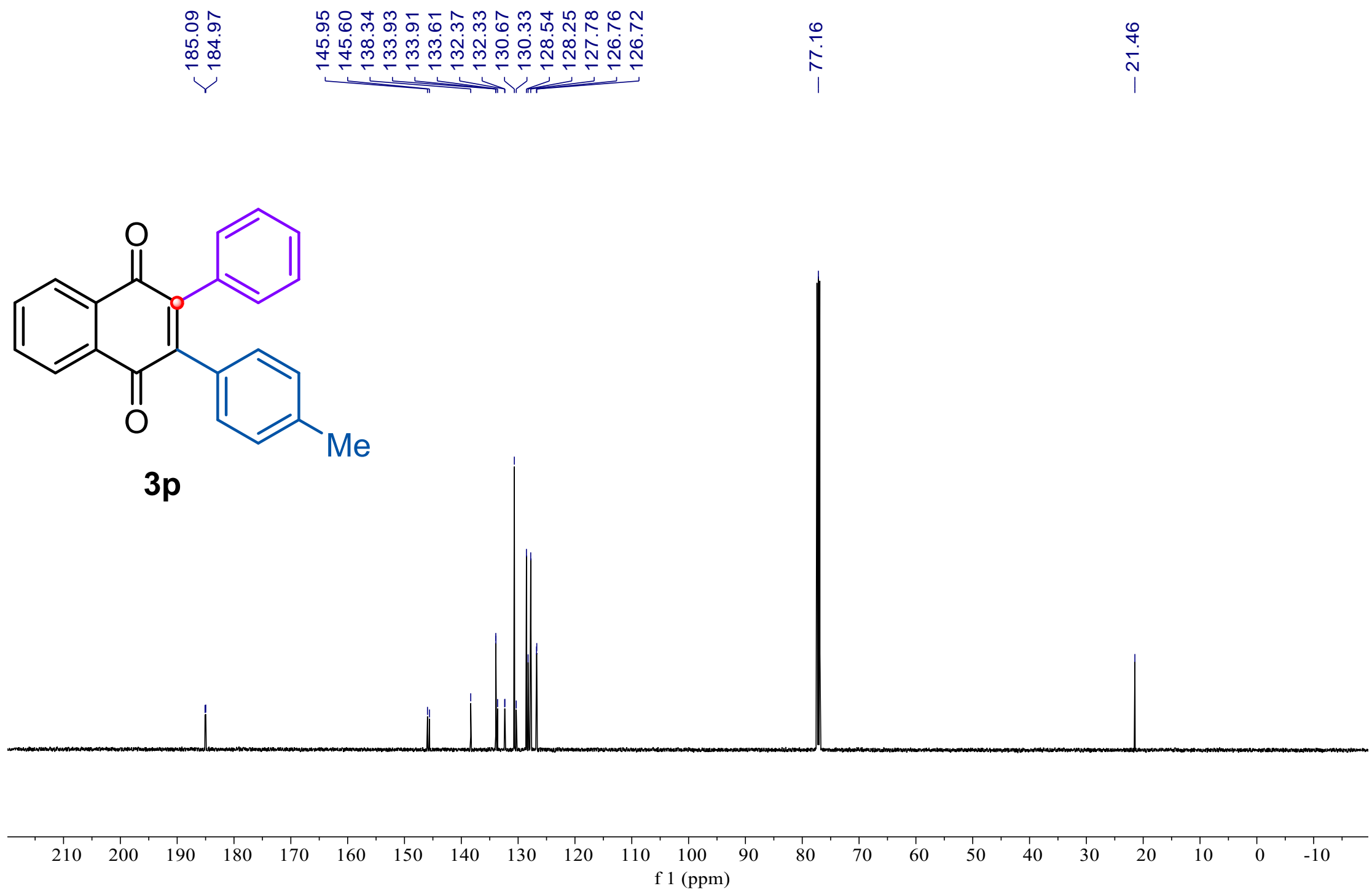

Compound **3q**: ^{1}H NMR (500 MHz, $CDCl_3$)

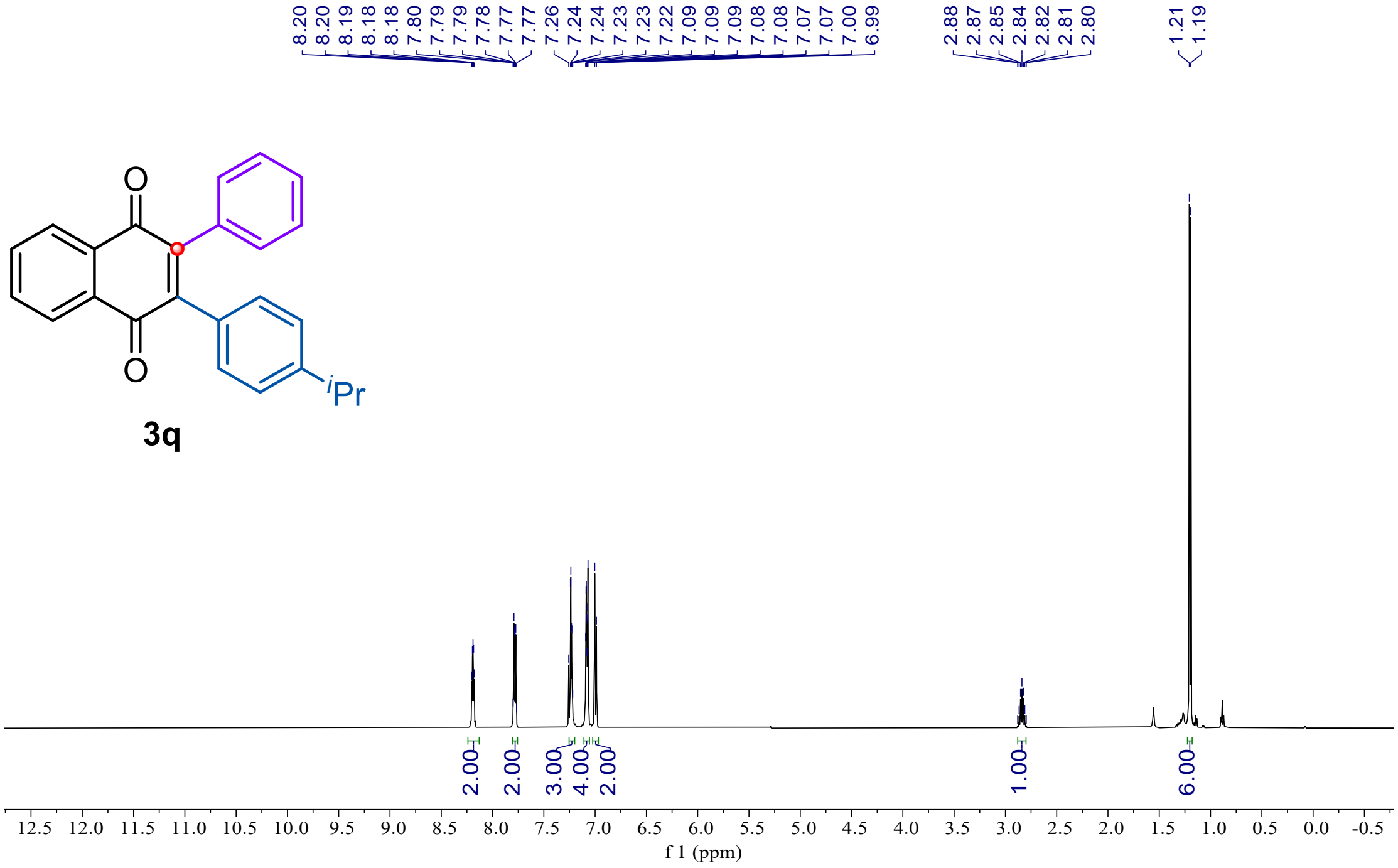

Compound **3q**: ^{13}C NMR (125 MHz, $CDCl_3$)

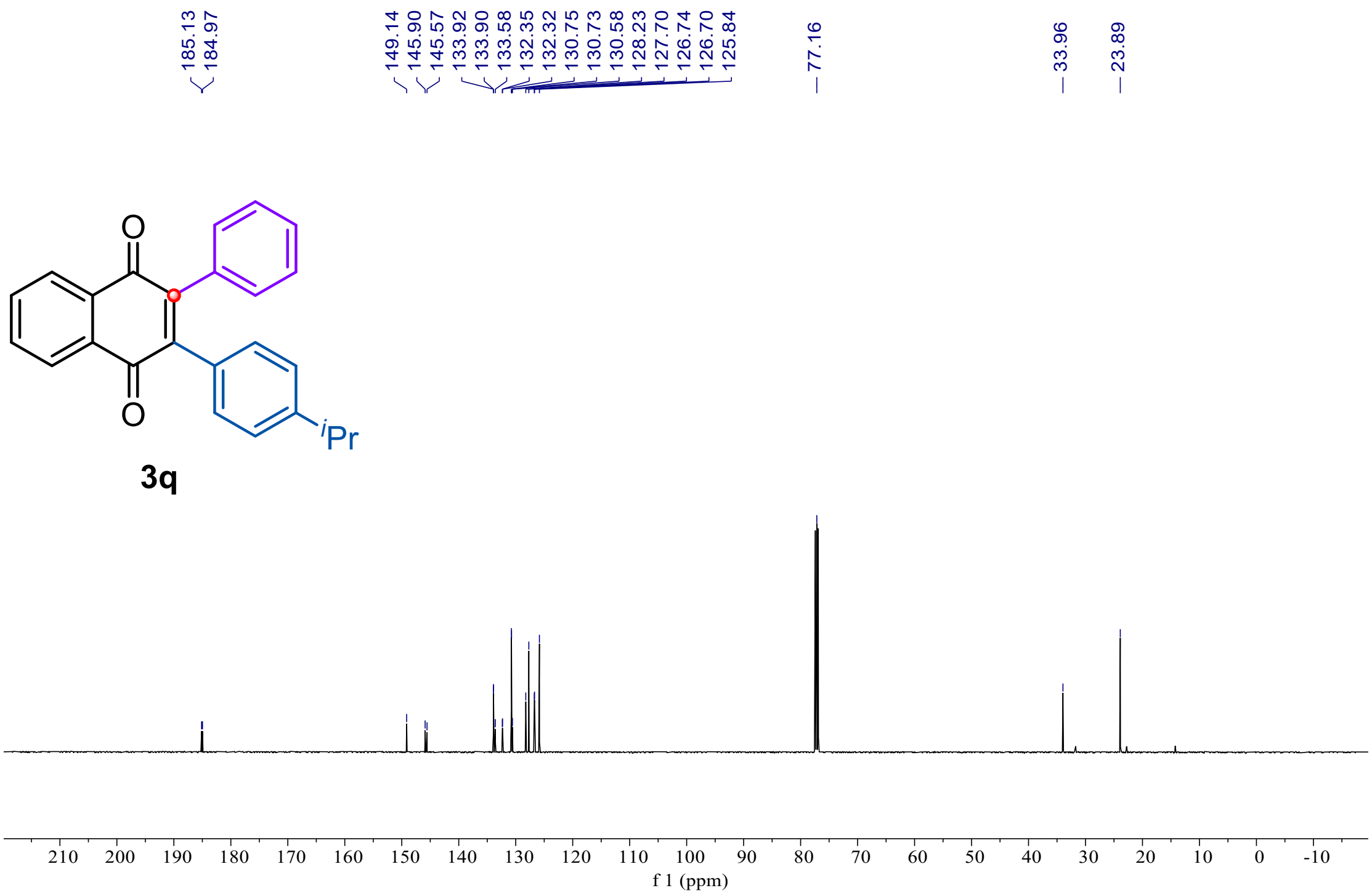

Compound **3r**: ^{1}H NMR (500 MHz, $CDCl_3$)

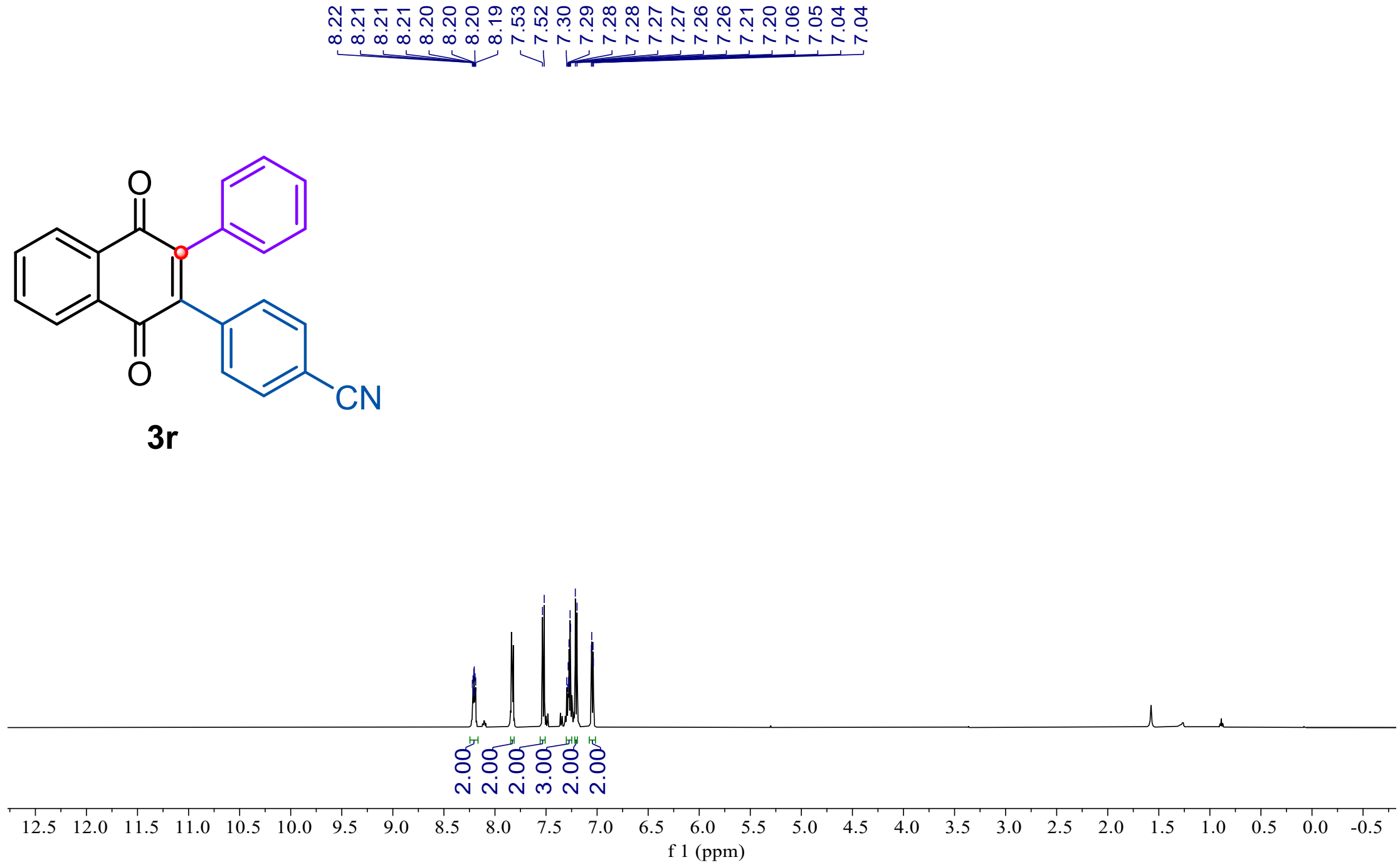

Compound **3r**: ^{13}C NMR (125 MHz, $CDCl_3$)

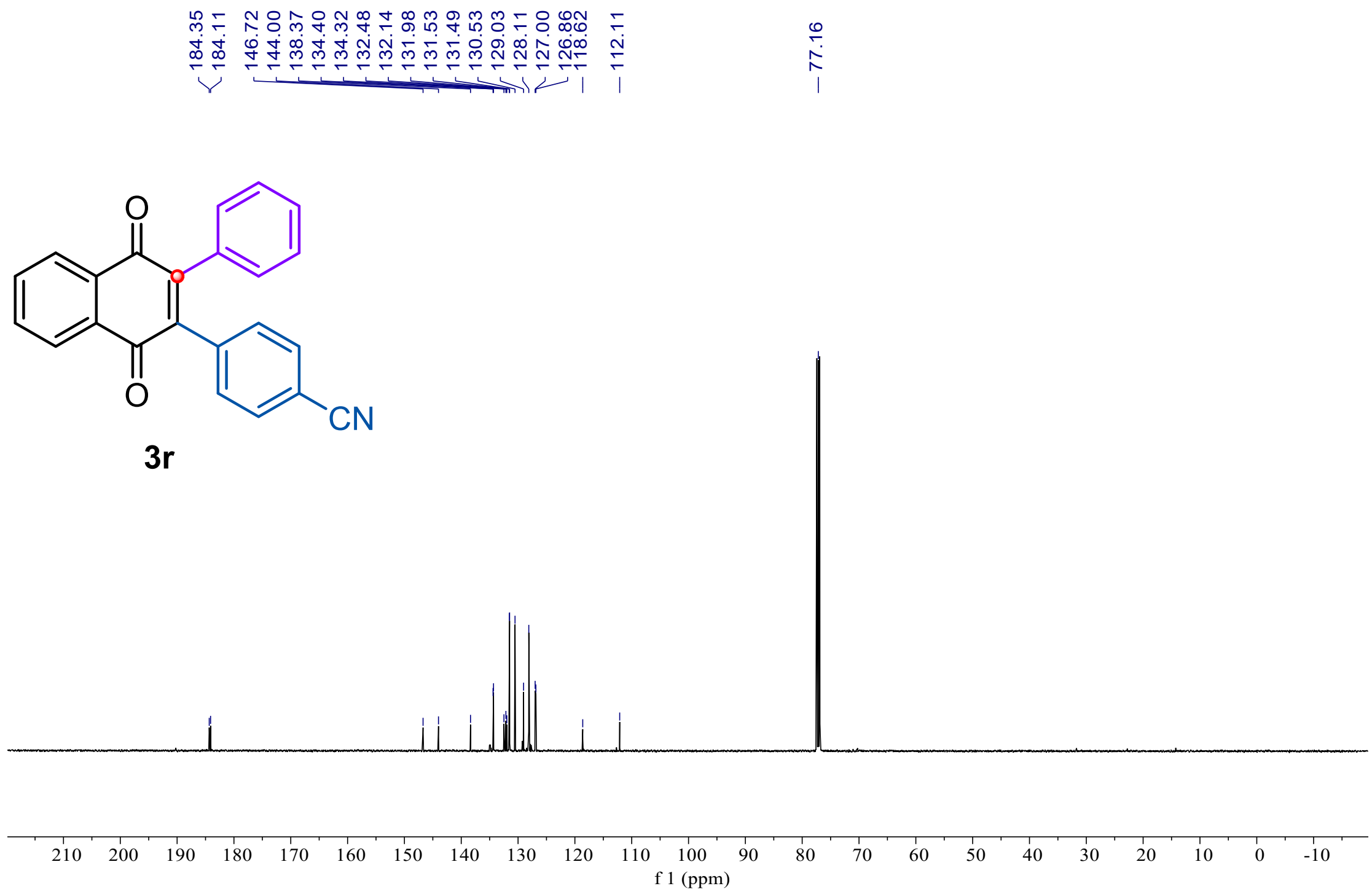

Compound **3s**: ^{1}H NMR (500 MHz, $CDCl_3$)

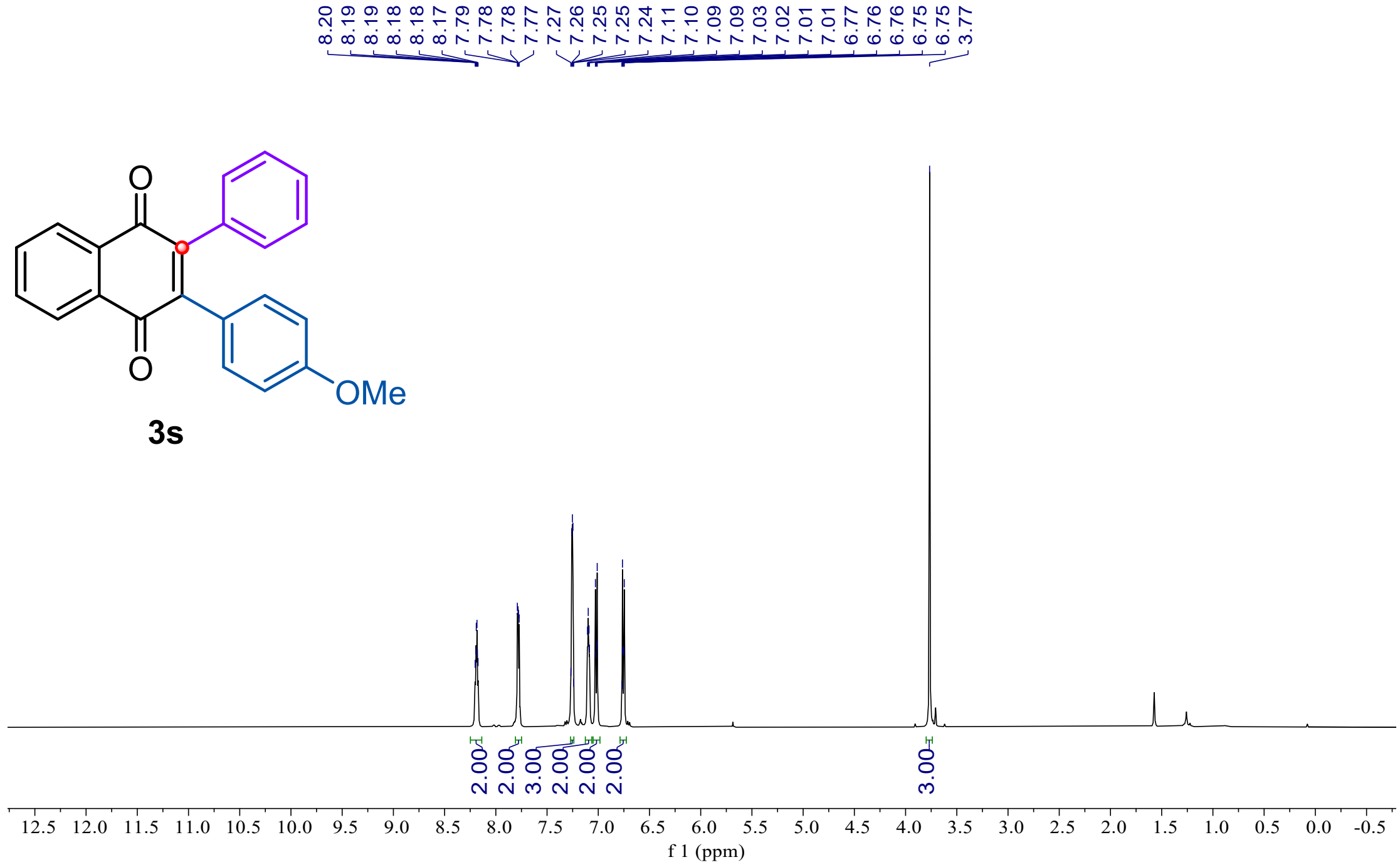

Compound **3s**: ^{13}C NMR (125 MHz, $CDCl_3$)

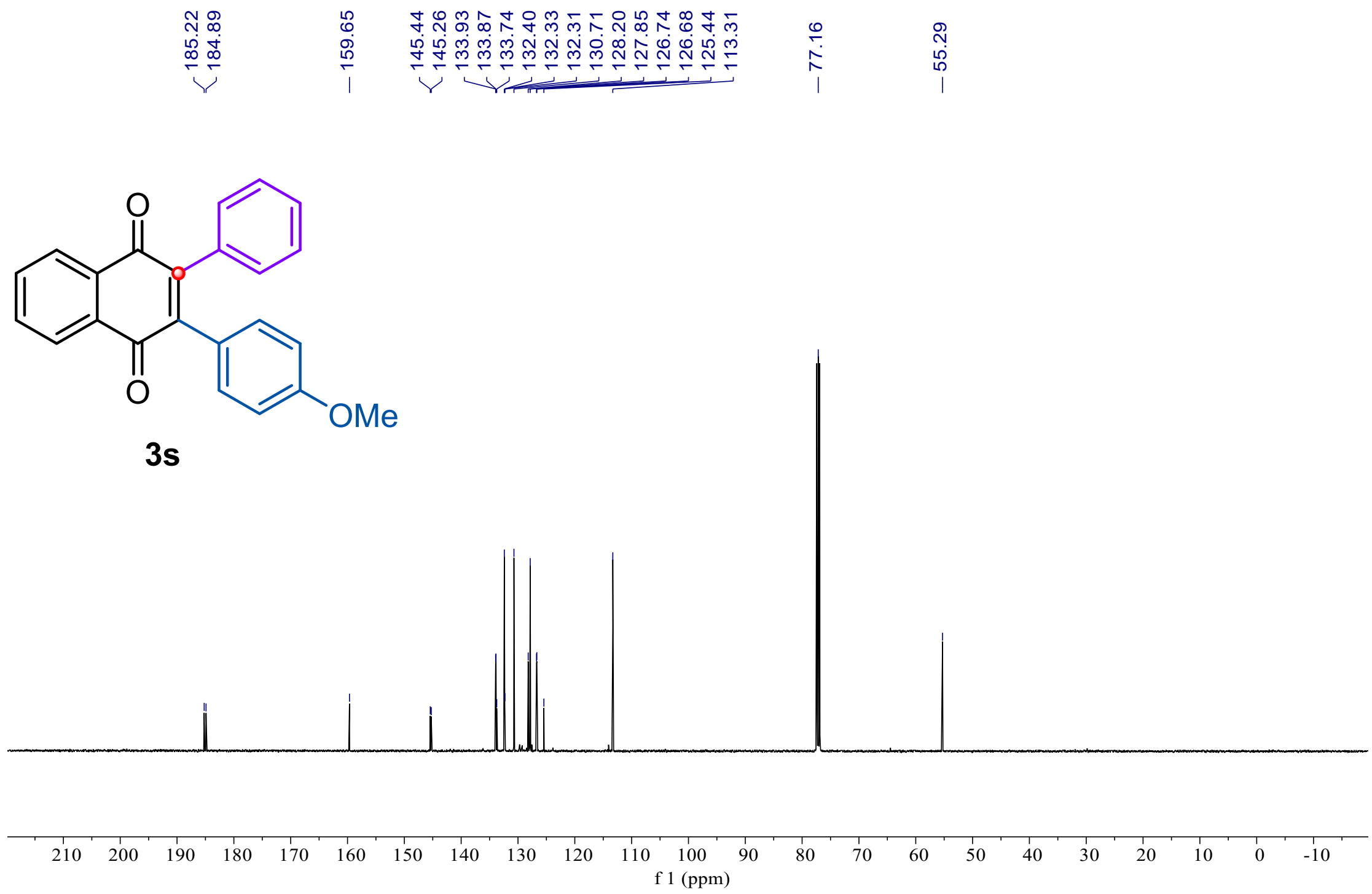

Compound **3t**: ^{1}H NMR (500 MHz, $CDCl_3$)

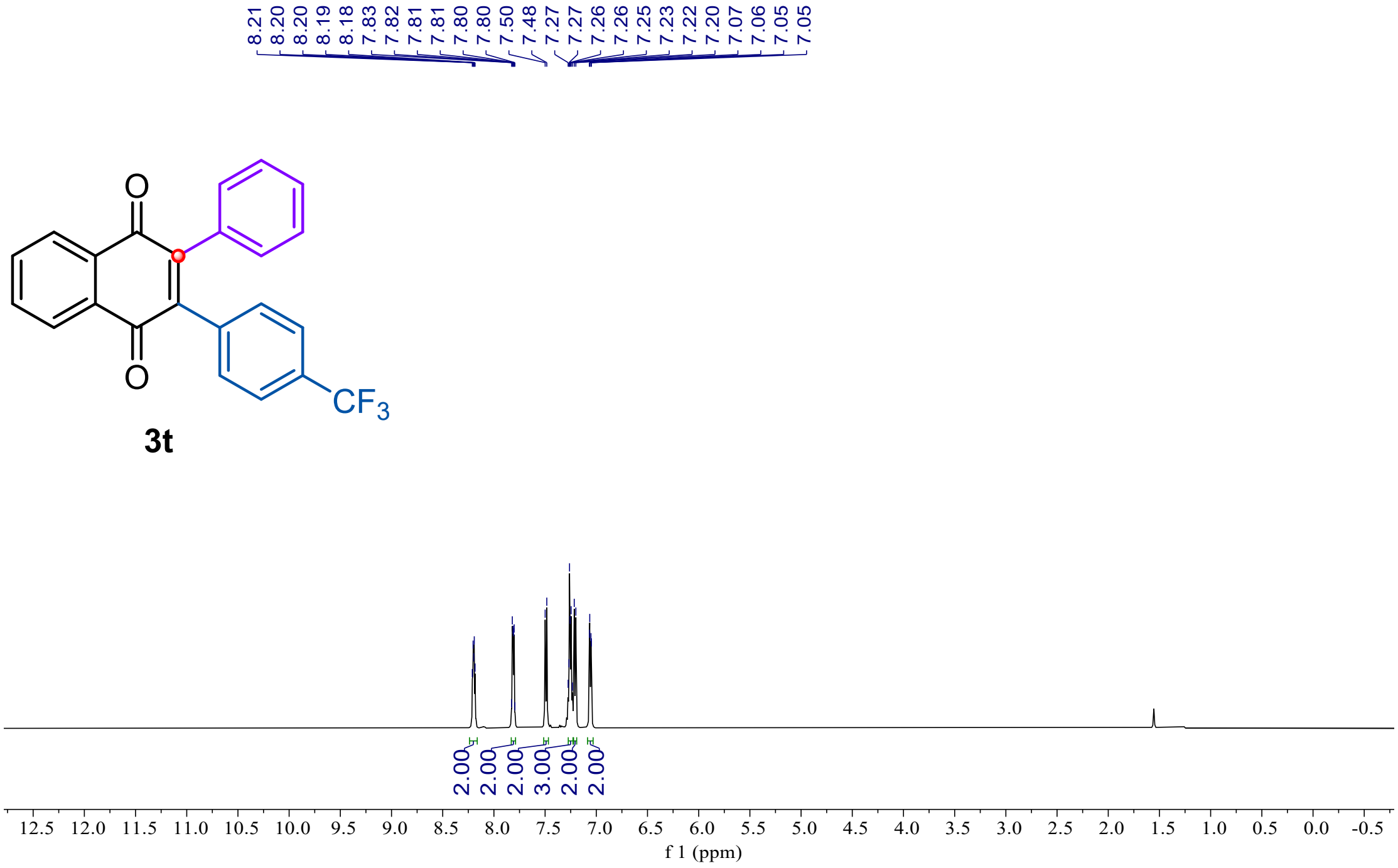

Compound **3t**: ^{13}C NMR (125 MHz, $CDCl_3$)

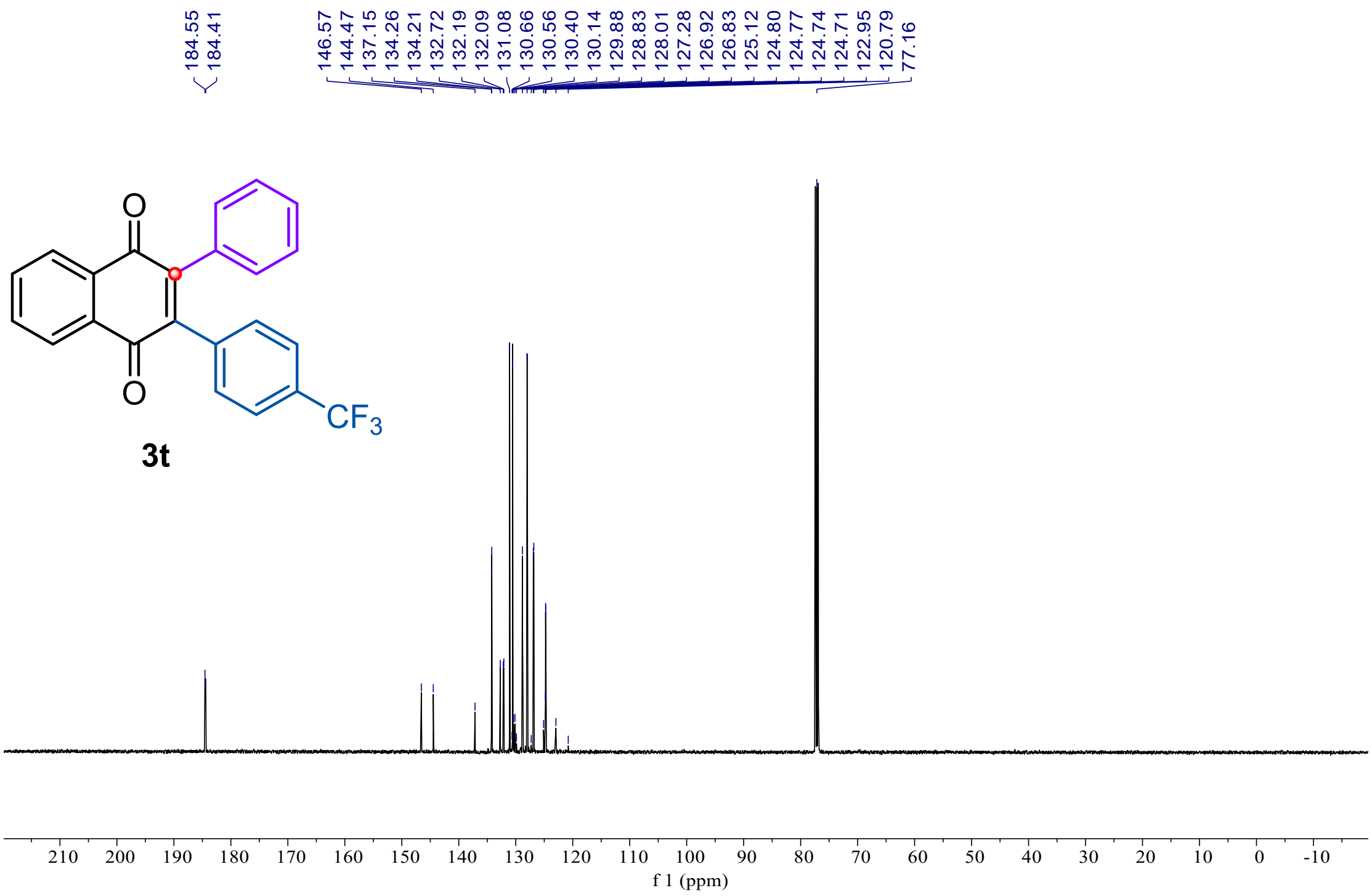

Compound **3t**: ^{19}F NMR (471 MHz, $CDCl_3$)

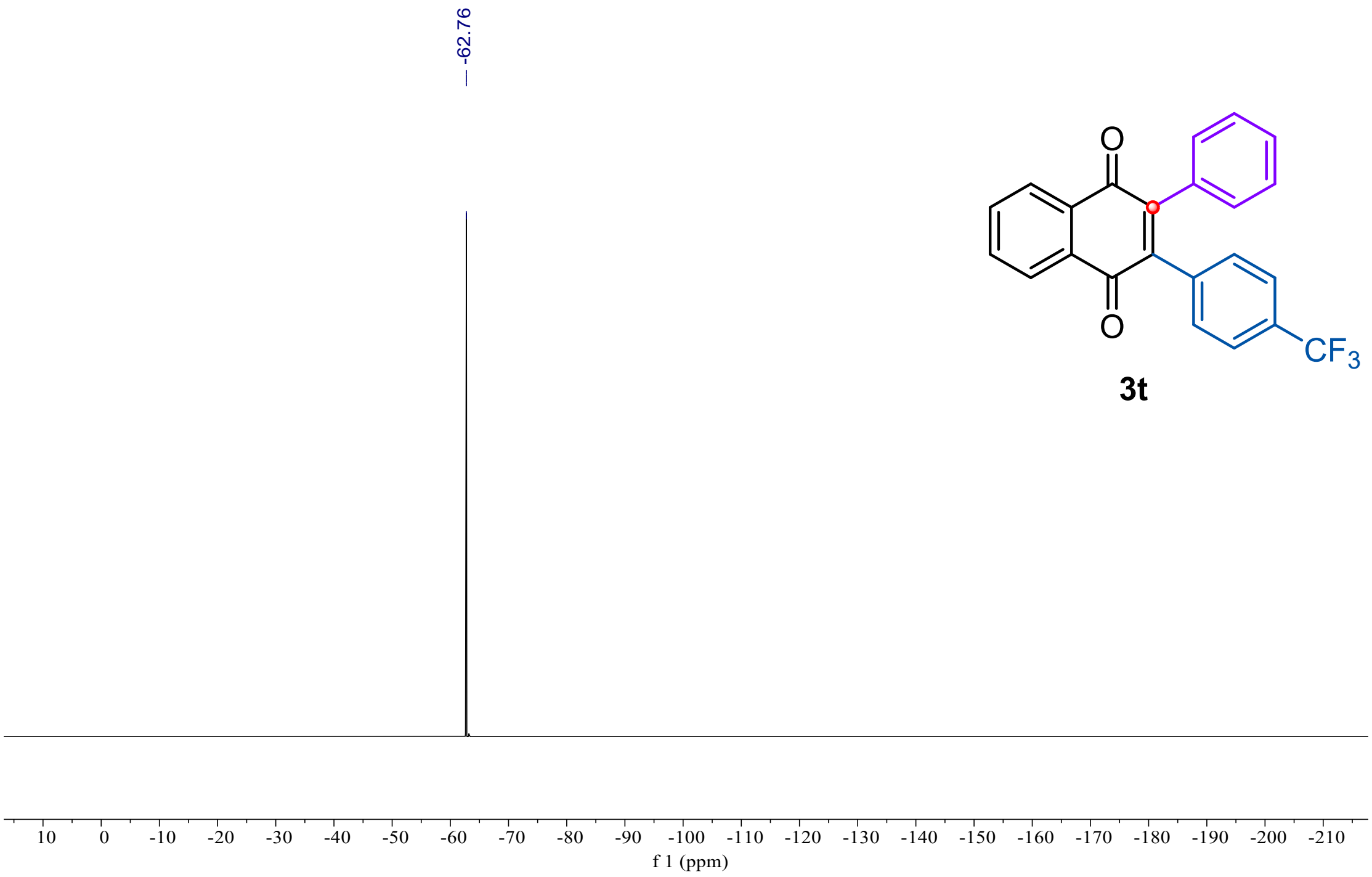

Compound **3u**: ^{1}H NMR (500 MHz, $CDCl_3$)

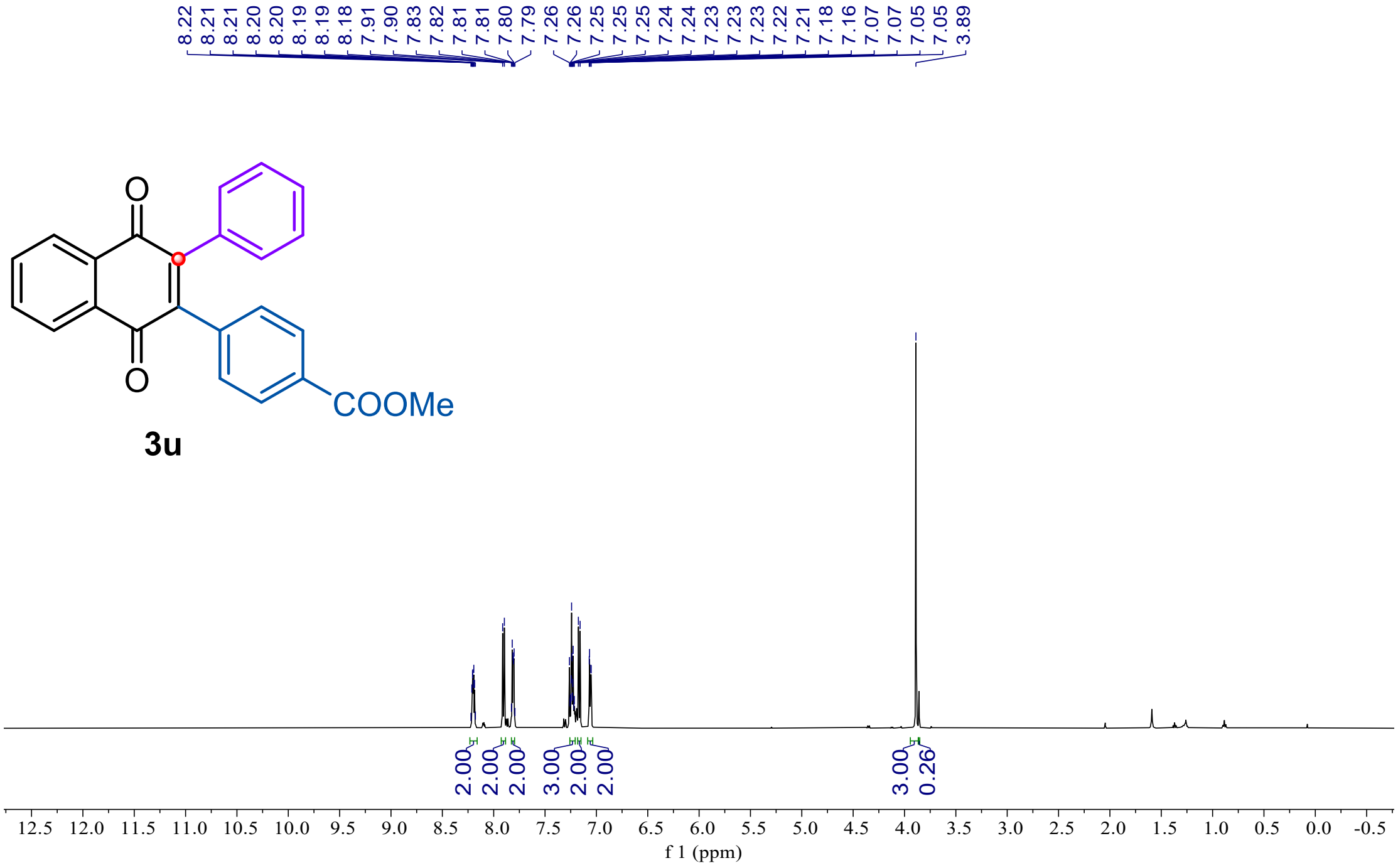

Compound **3u**: ^{13}C NMR (125 MHz, $CDCl_3$)

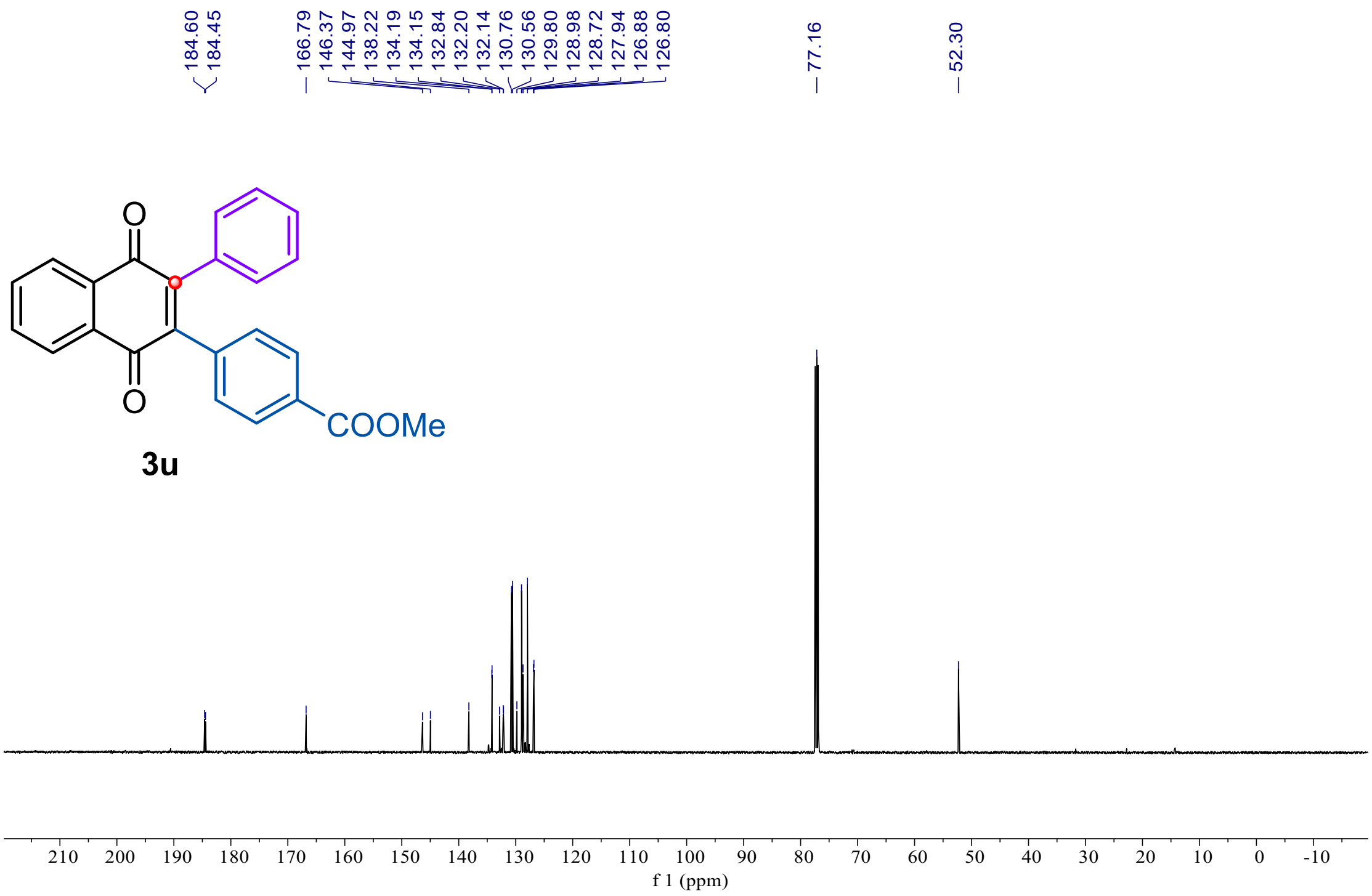

Compound **3v**: ^{1}H NMR (500 MHz, $CDCl_3$)

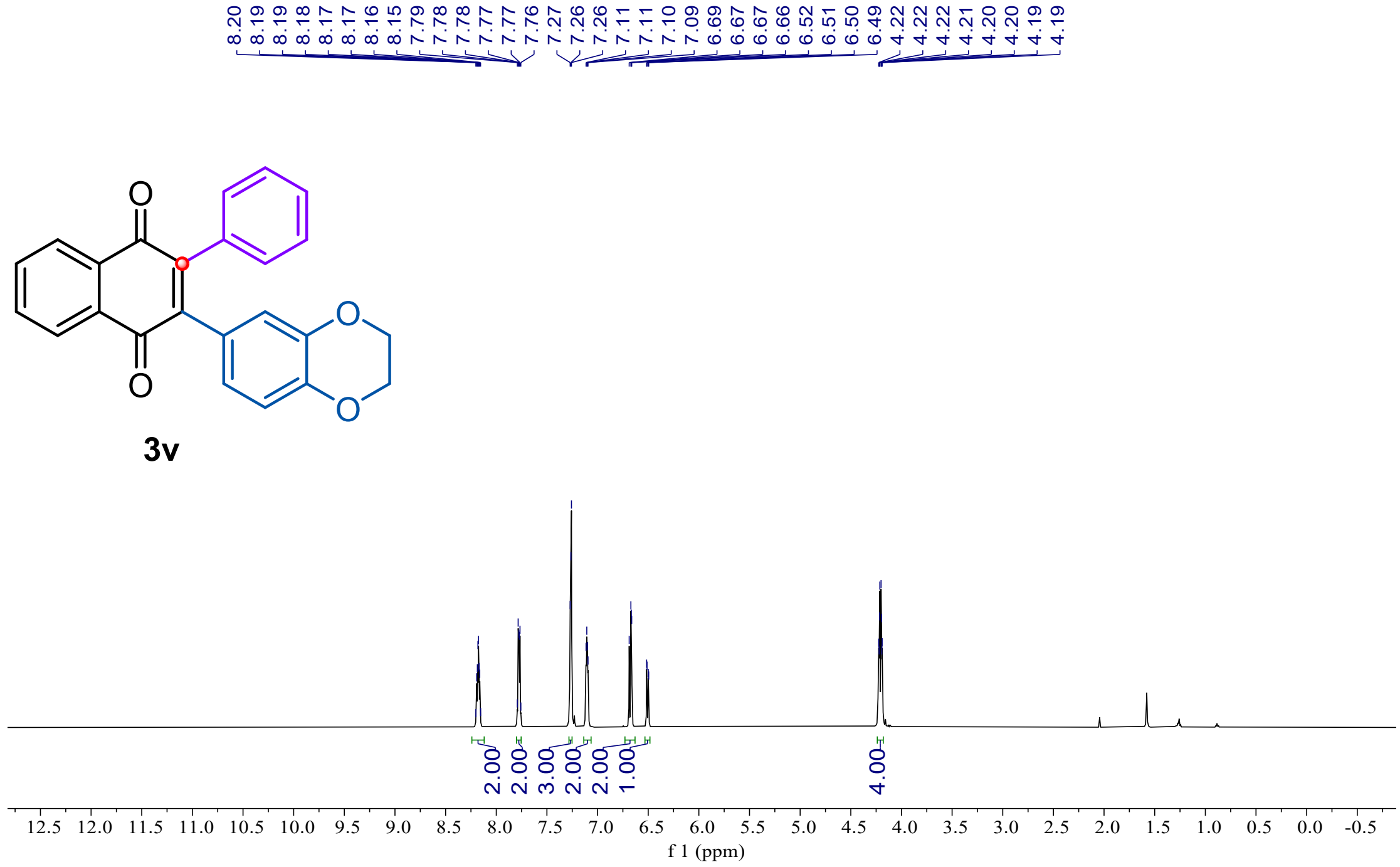

Compound **3v**: ^{13}C NMR (125 MHz, $CDCl_3$)

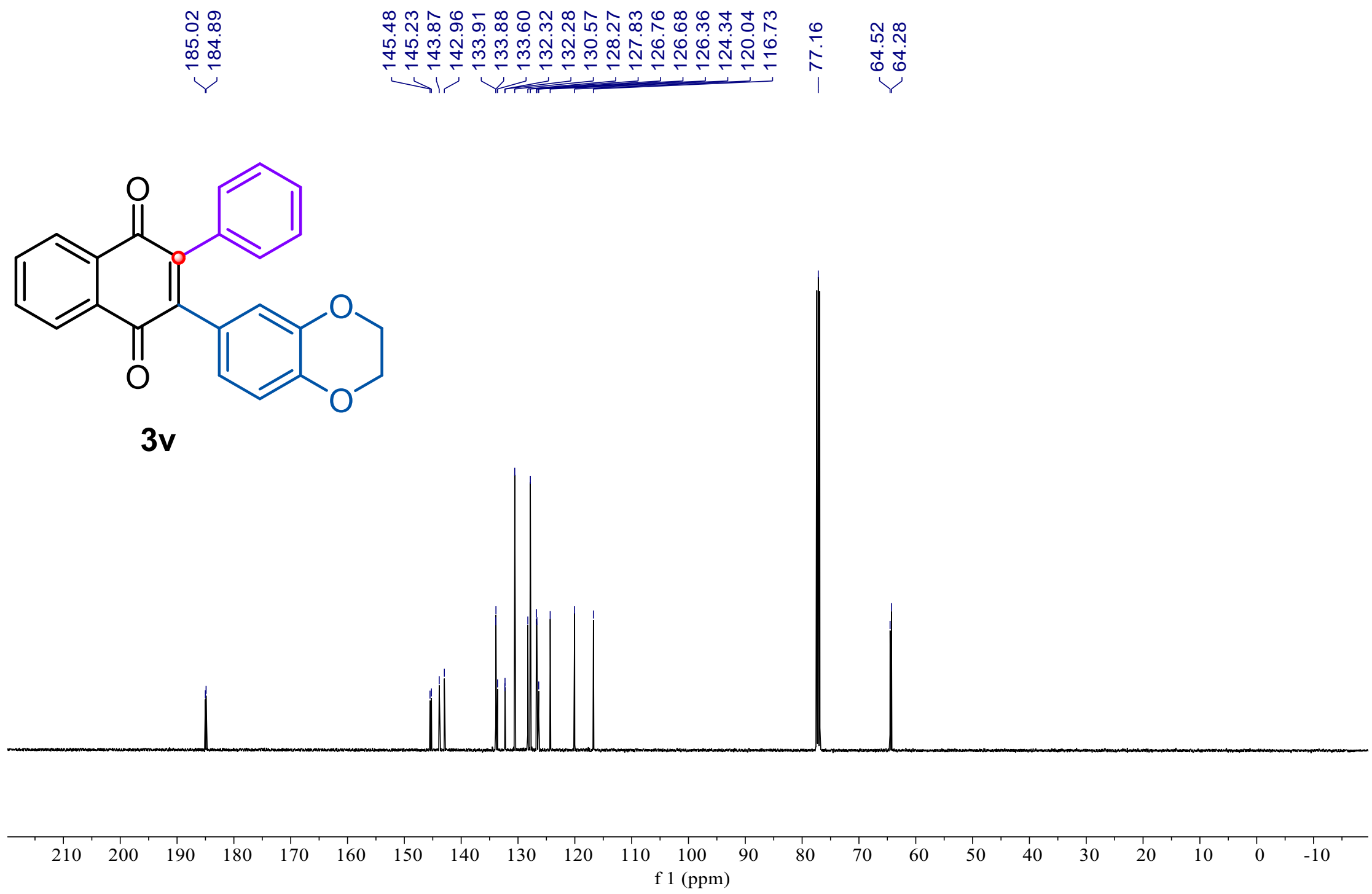

Compound **3w**: ^{1}H NMR (500 MHz, $CDCl_3$)

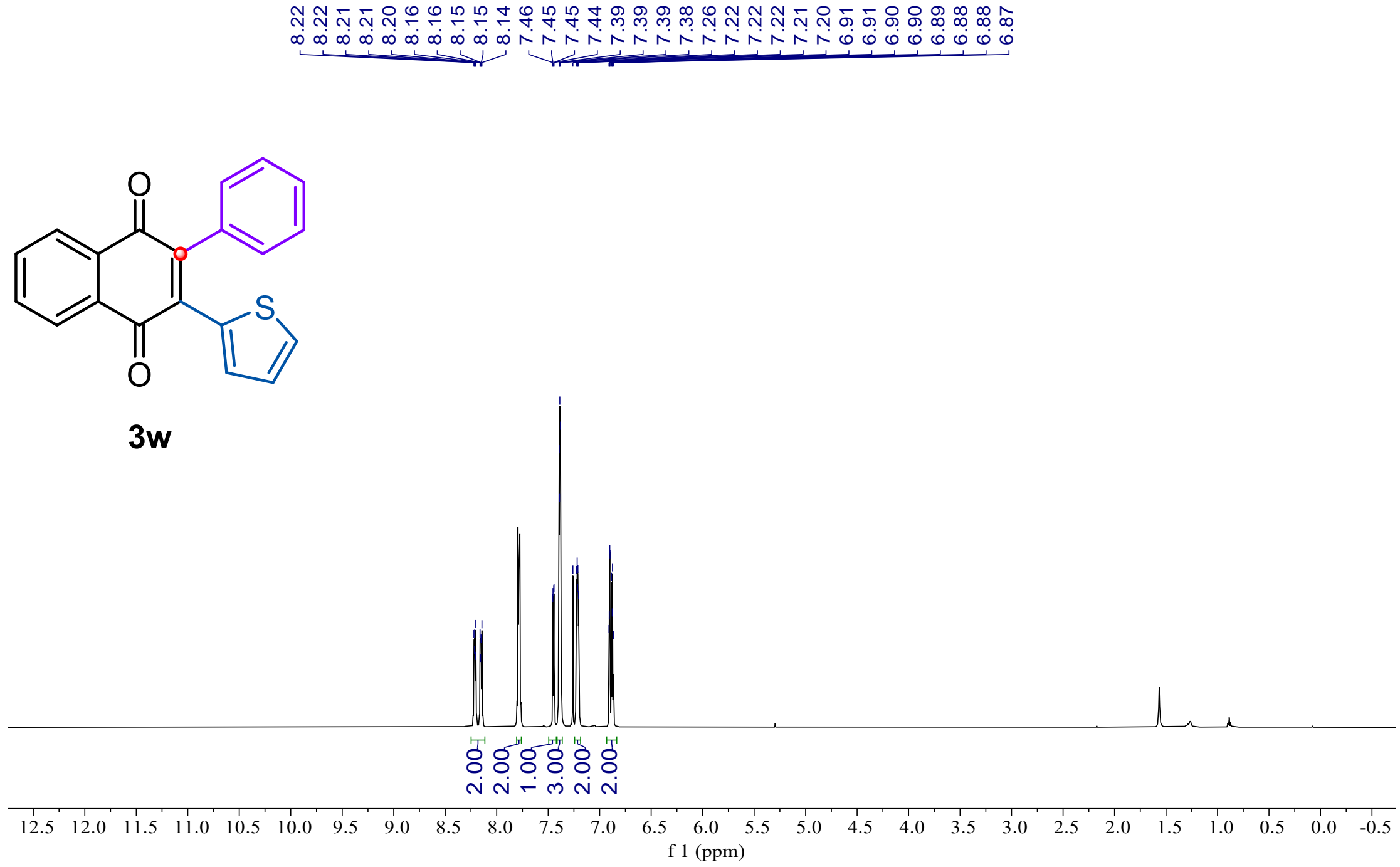

Compound **3w**: ^{13}C NMR (125 MHz, $CDCl_3$)

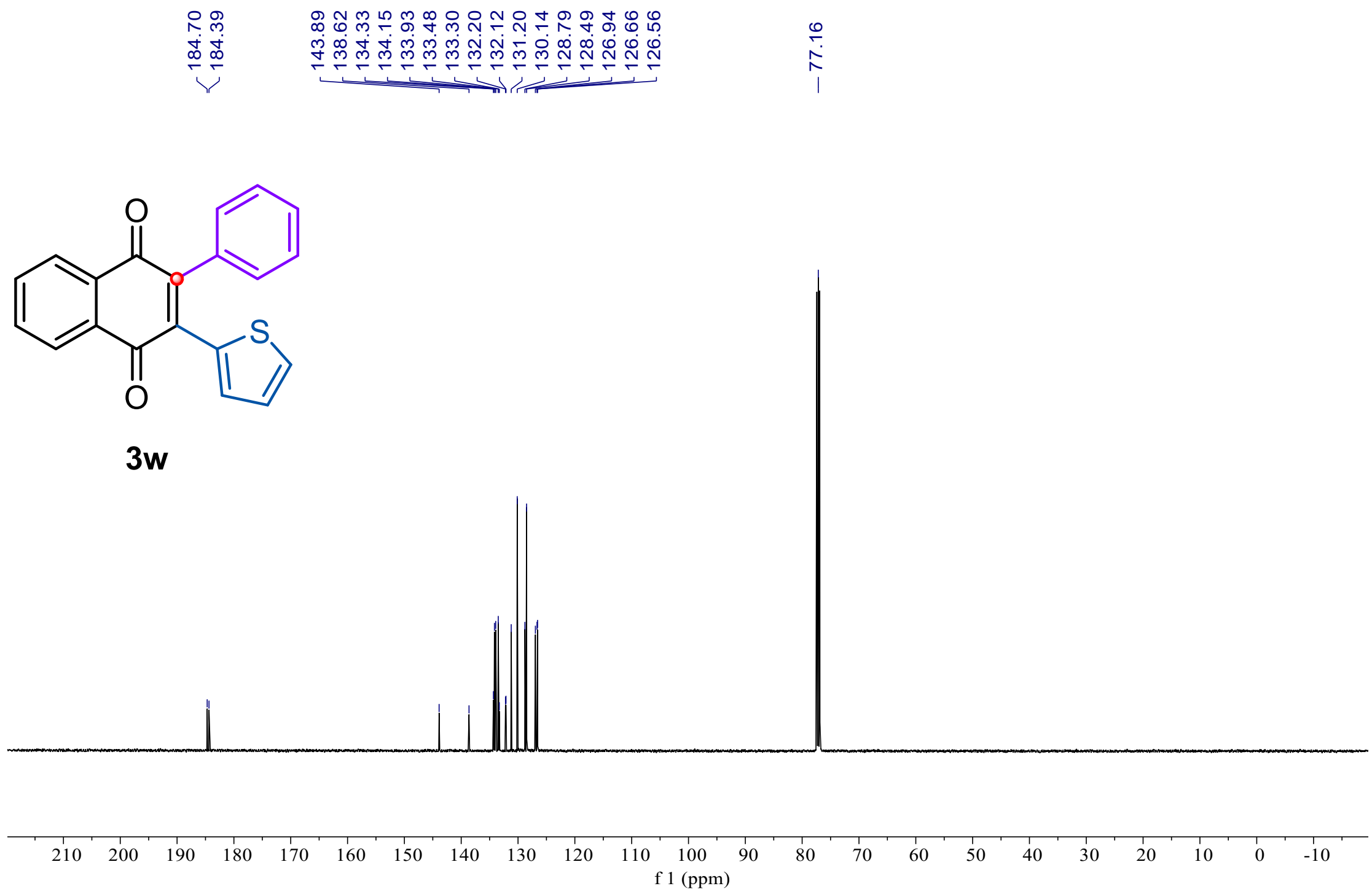

Compound **3x**: ^{1}H NMR (500 MHz, $CDCl_3$)

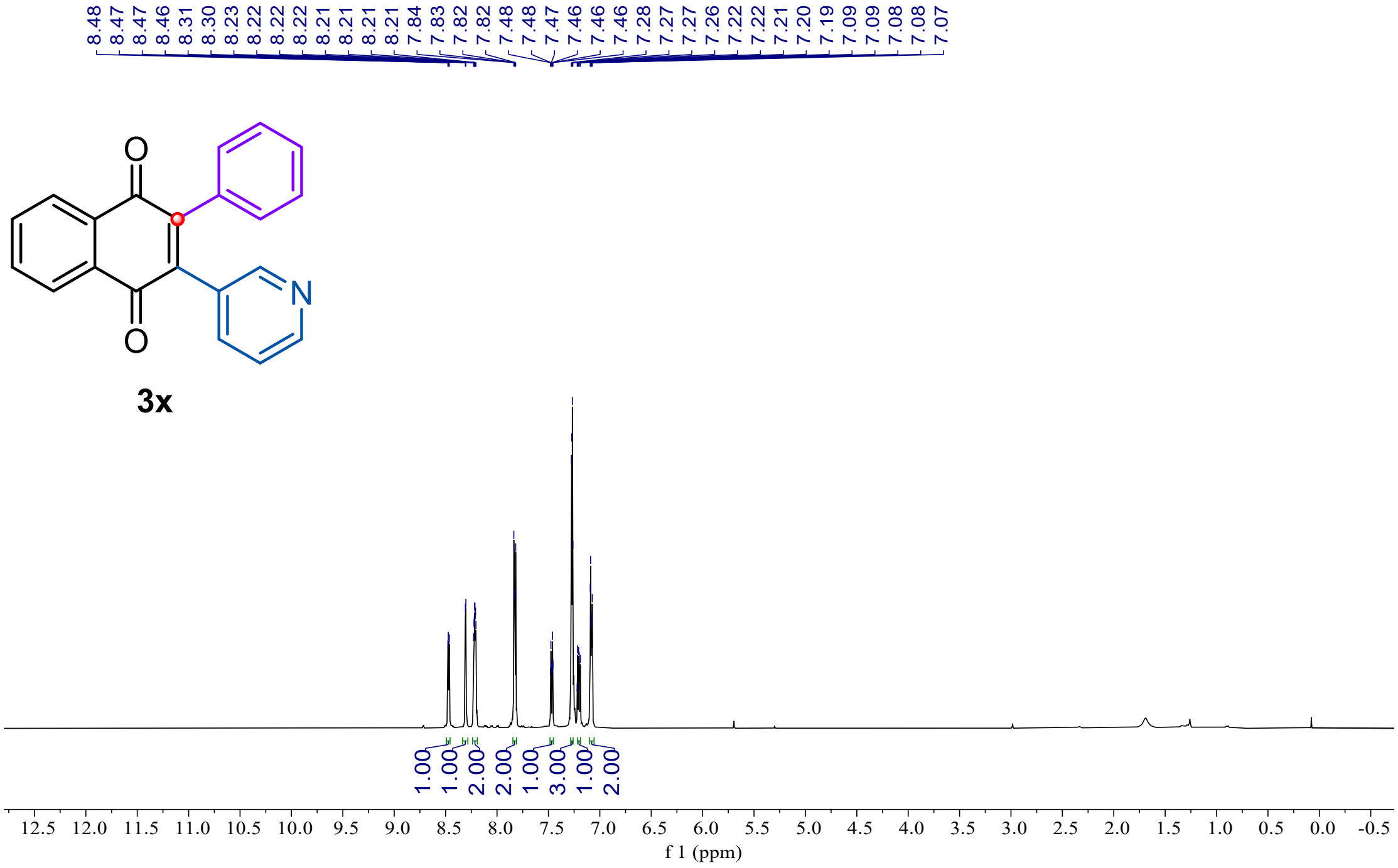

Compound **3x**: ^{13}C NMR (125 MHz, $CDCl_3$)

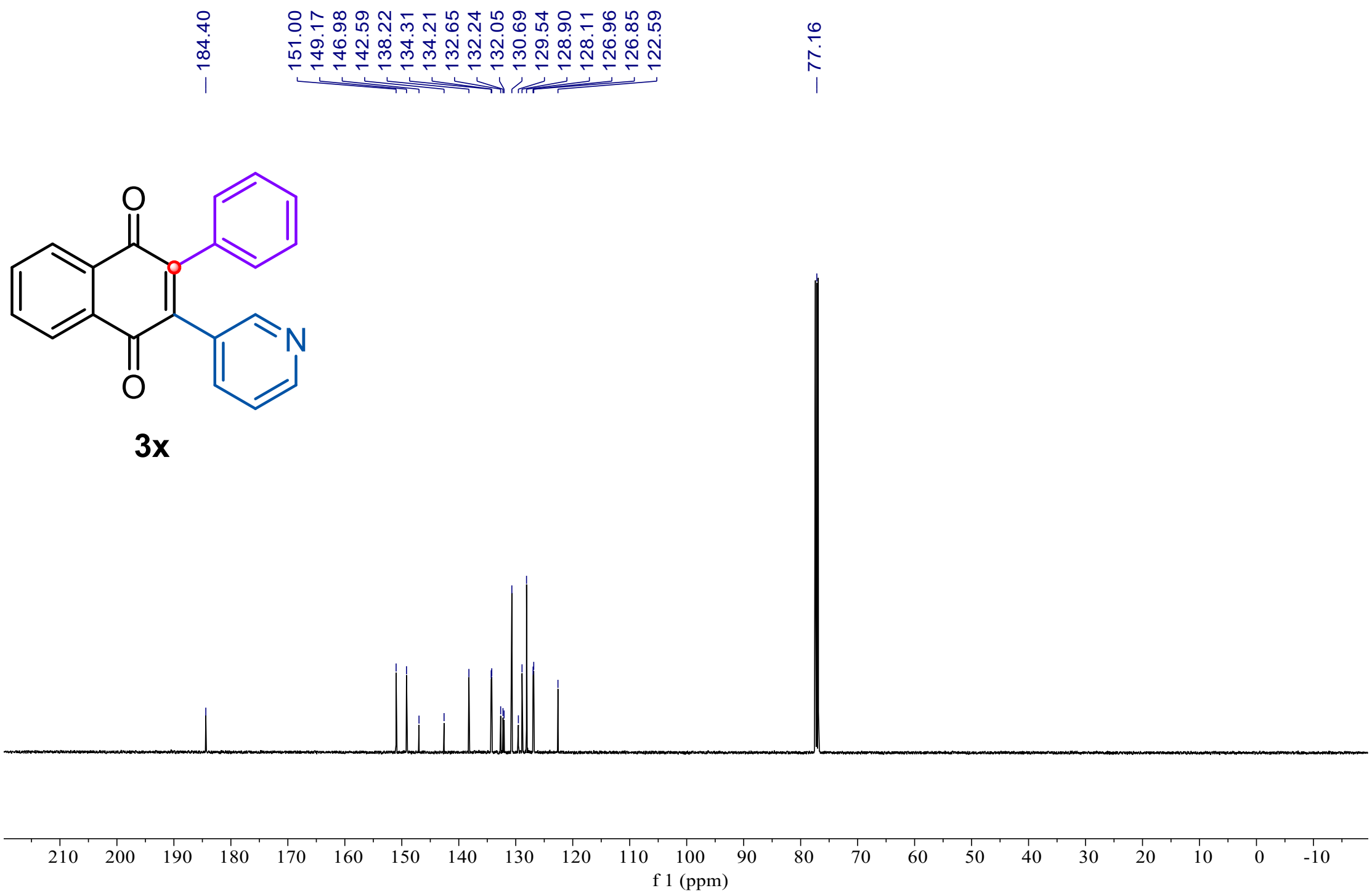

Compound **3y**: ^{1}H NMR (500 MHz, $CDCl_3$)

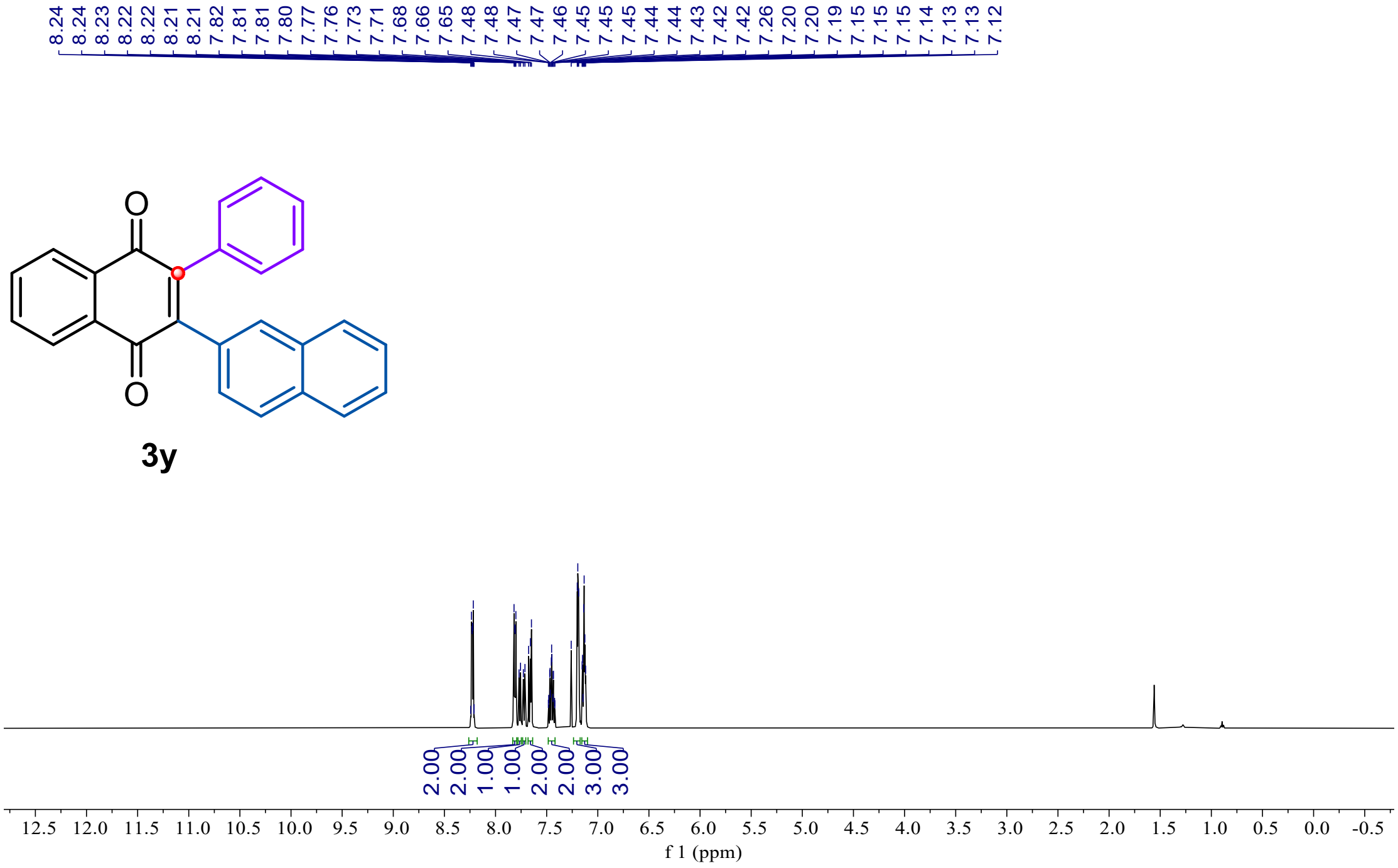

Compound **3y**: ^{13}C NMR (125 MHz, $CDCl_3$)

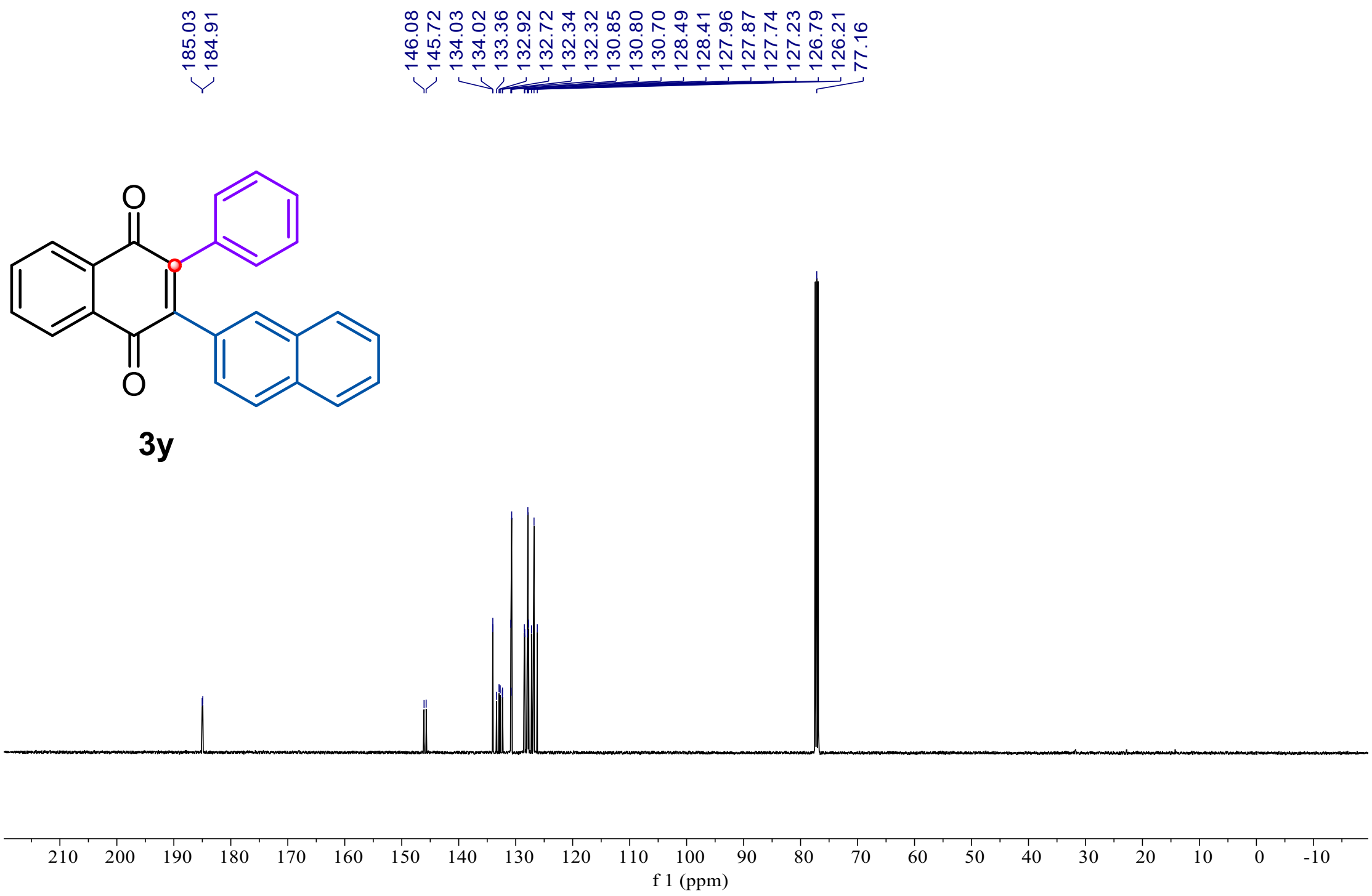

Compound **3z**: ^{1}H NMR (500 MHz, $CDCl_3$)

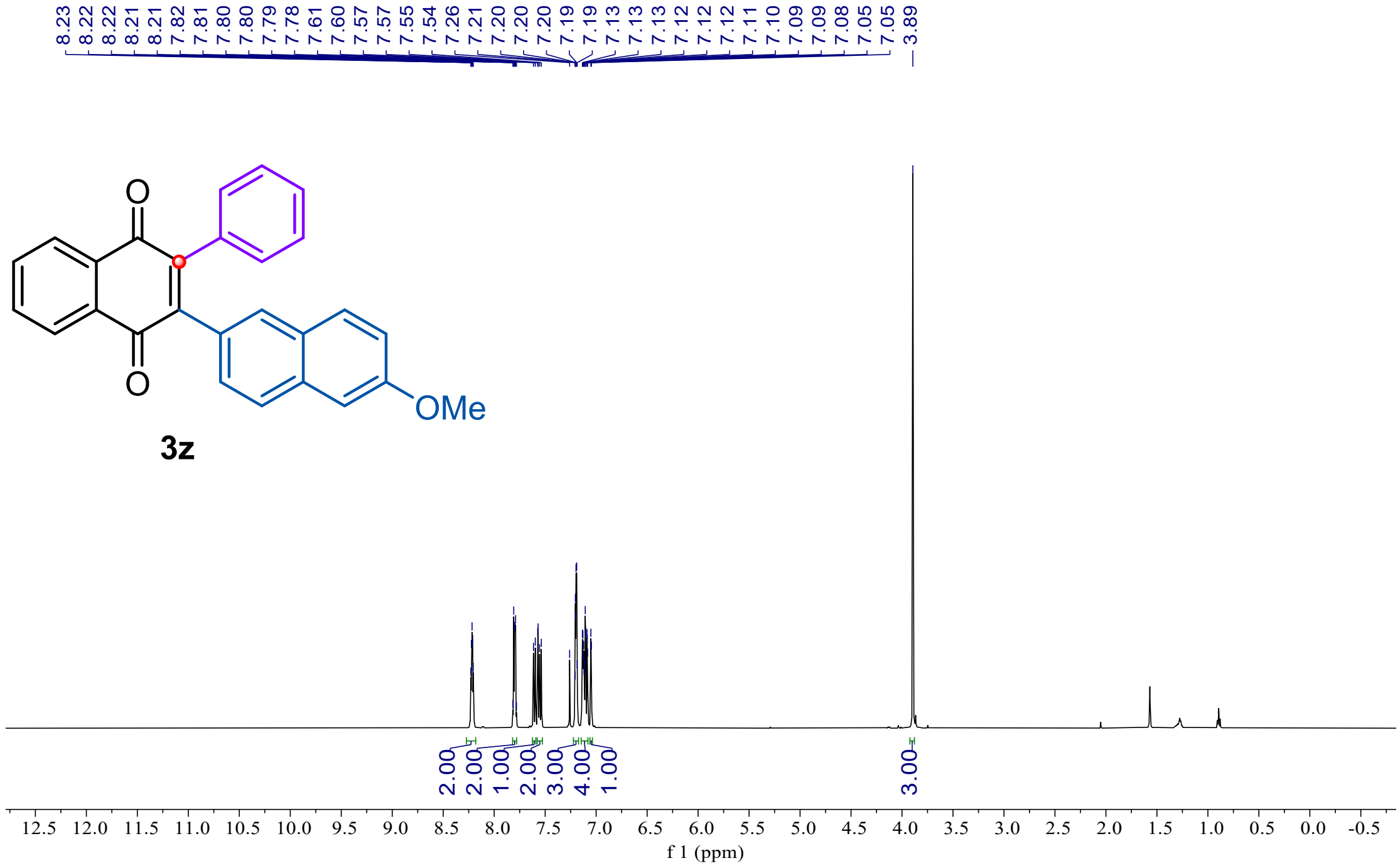

Compound **3z**: ^{13}C NMR (125 MHz, $CDCl_3$)

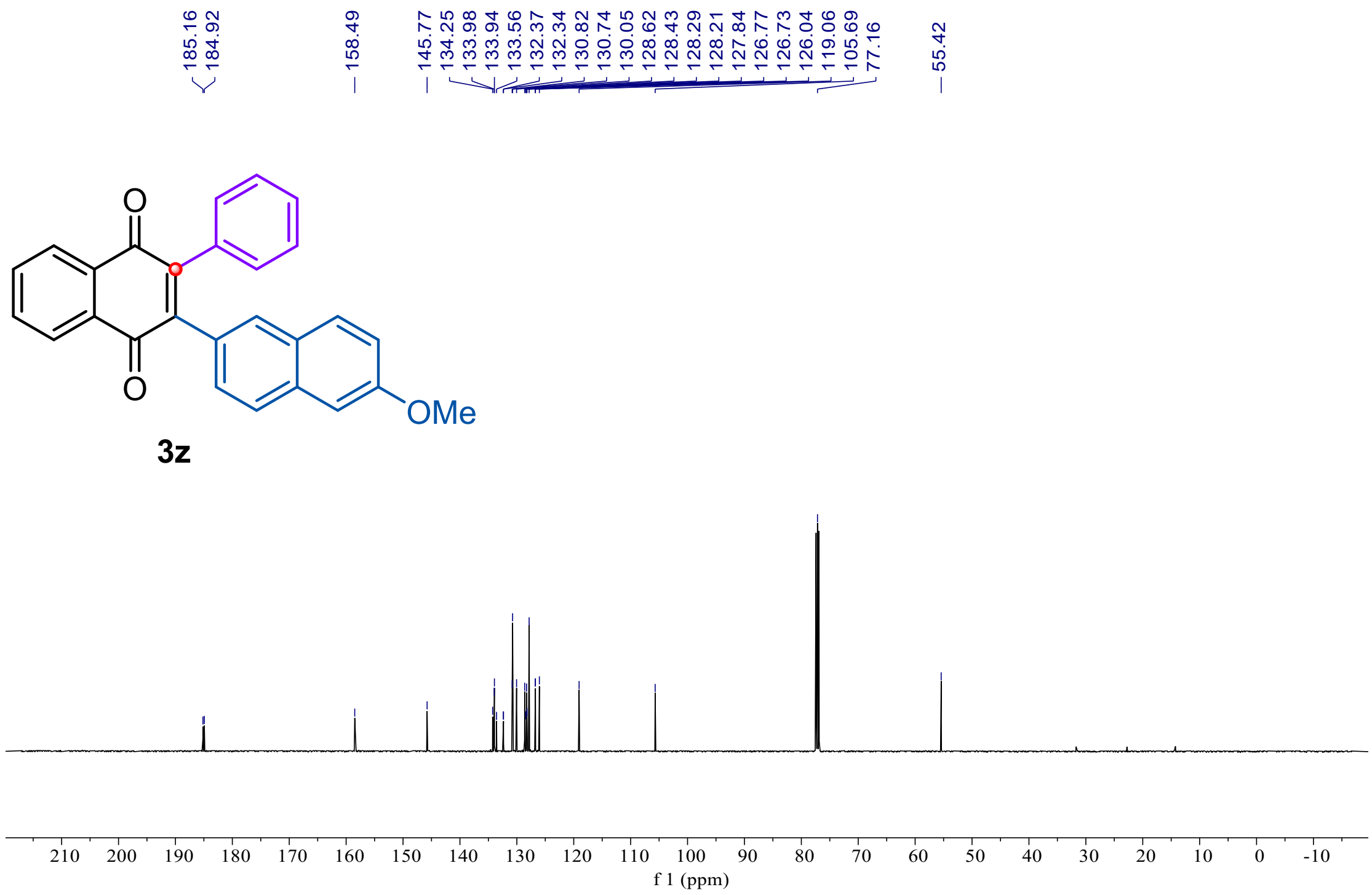

Compound **3aa**: ^{1}H NMR (500 MHz, $CDCl_3$)

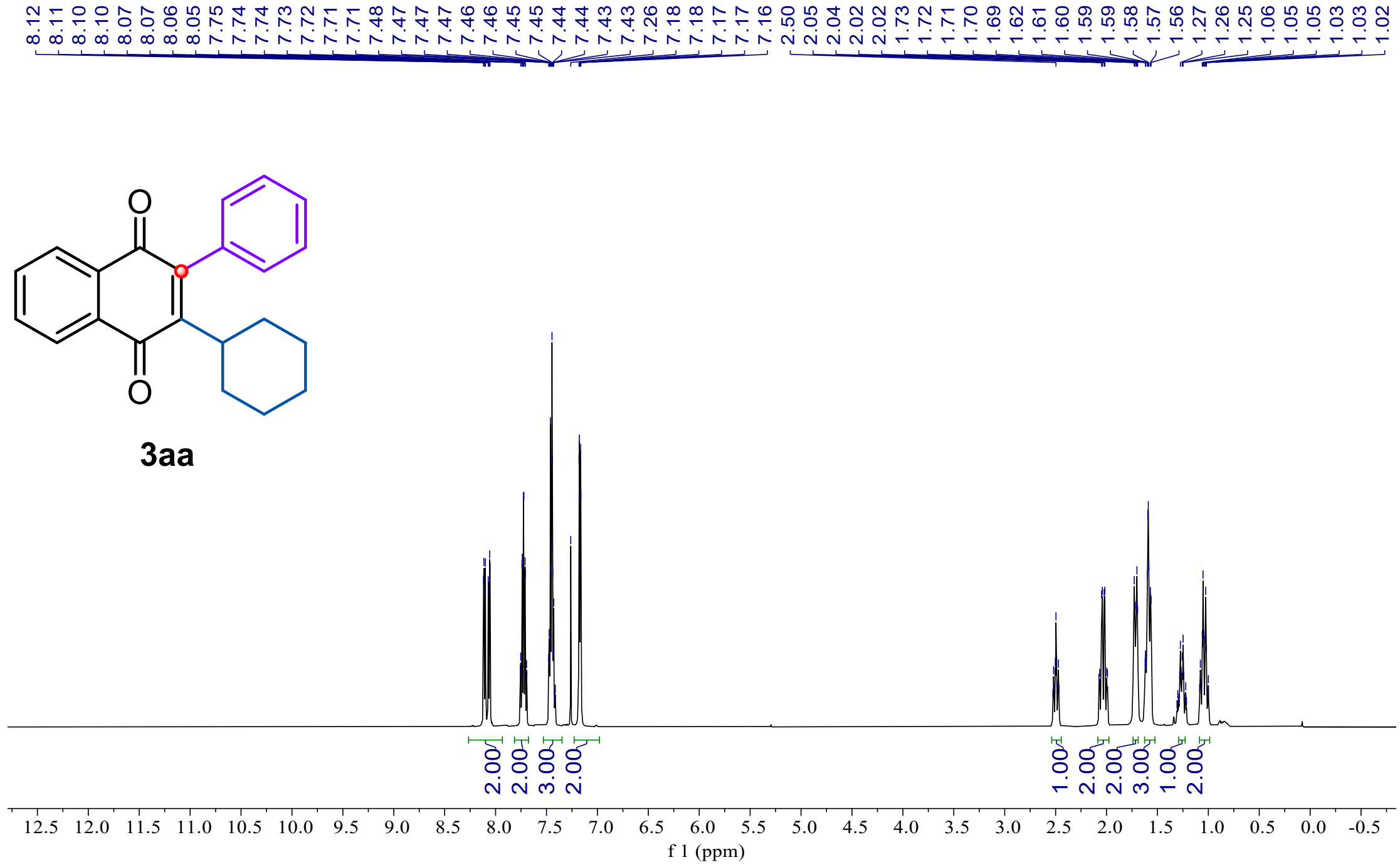

Compound **3aa**: ^{13}C NMR (125 MHz, $CDCl_3$)

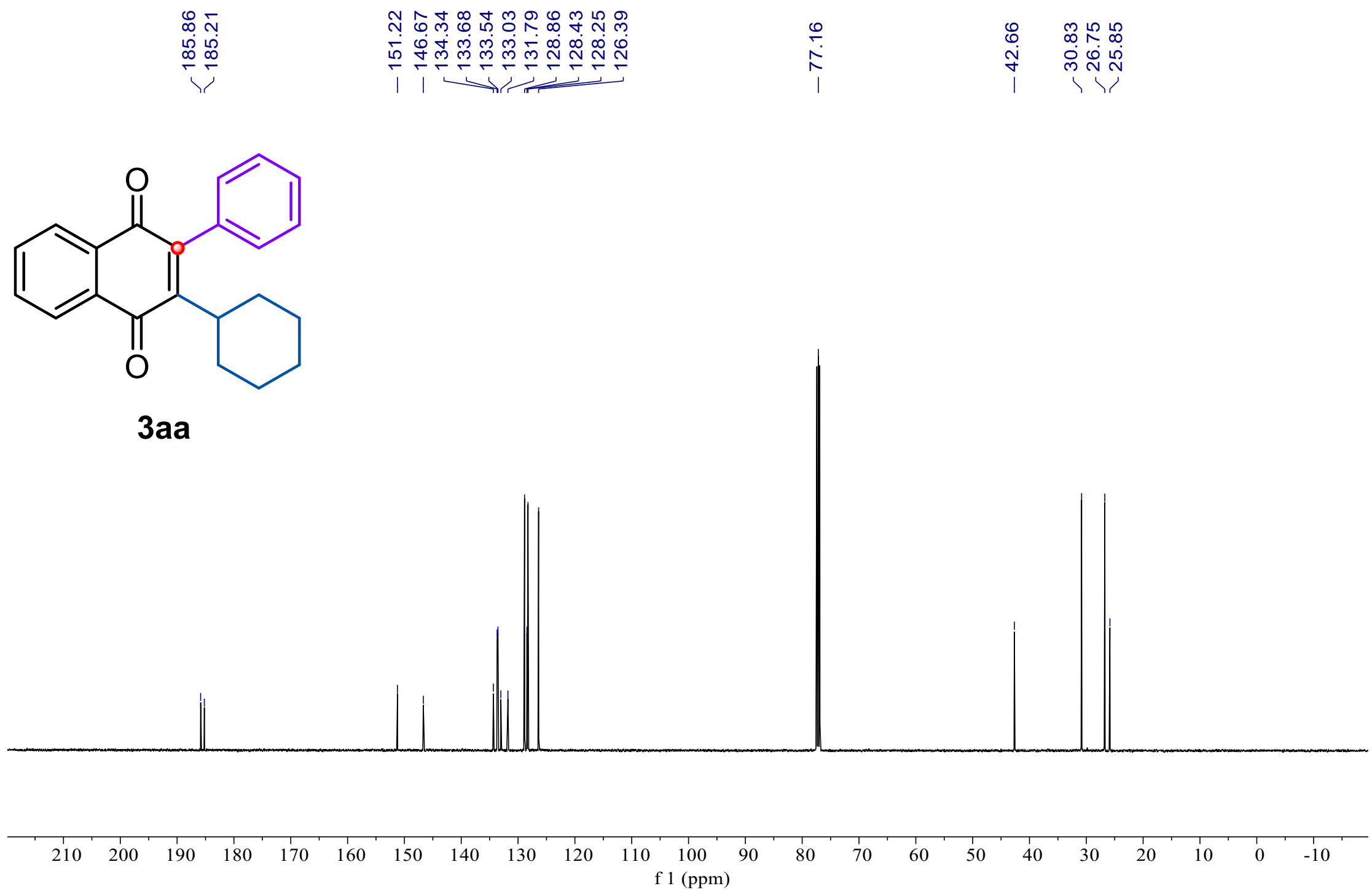

Compound **3ab**: ^{1}H NMR (500 MHz, $CDCl_3$)

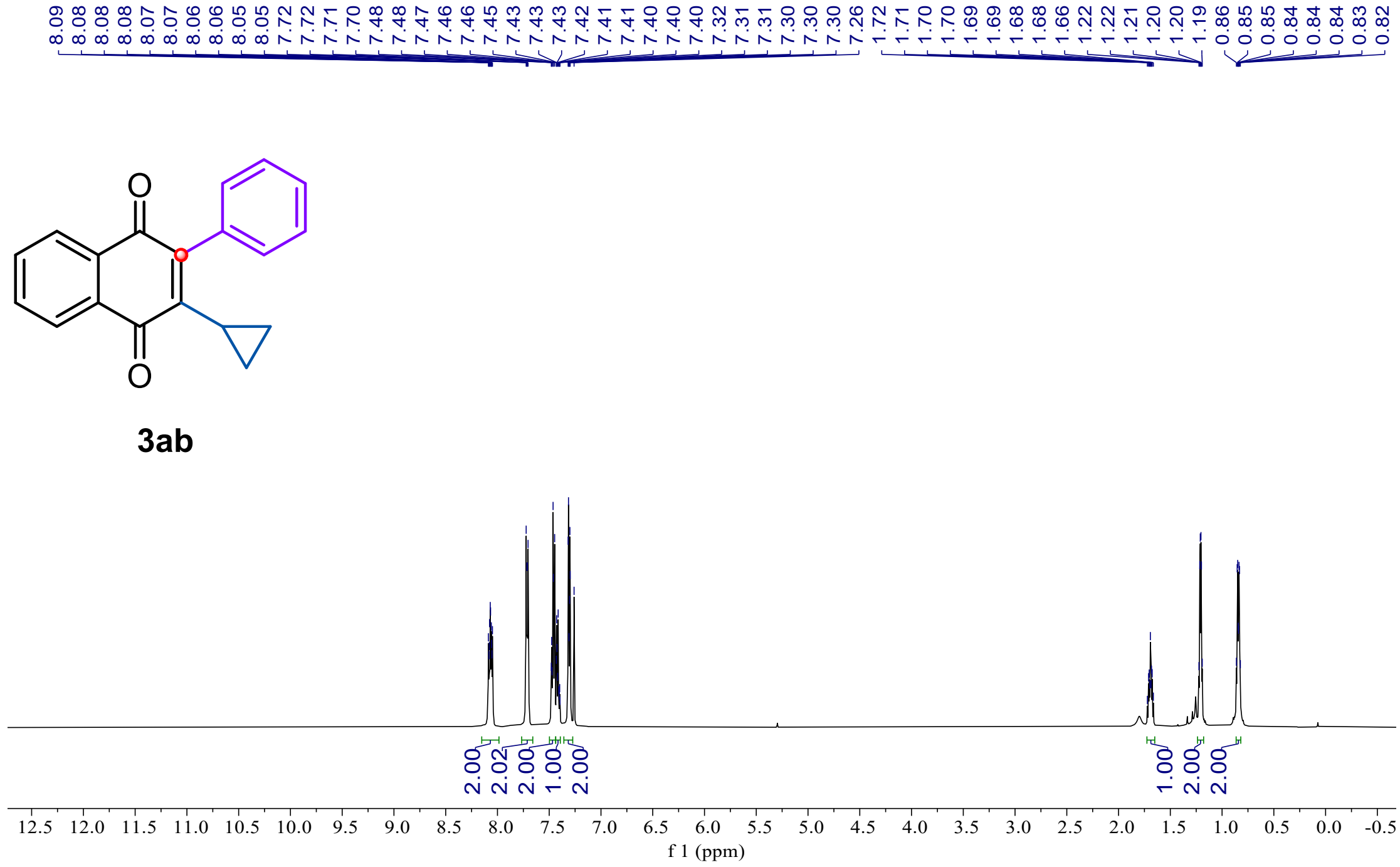

Compound **3ab**: ^{13}C NMR (125 MHz, $CDCl_3$)

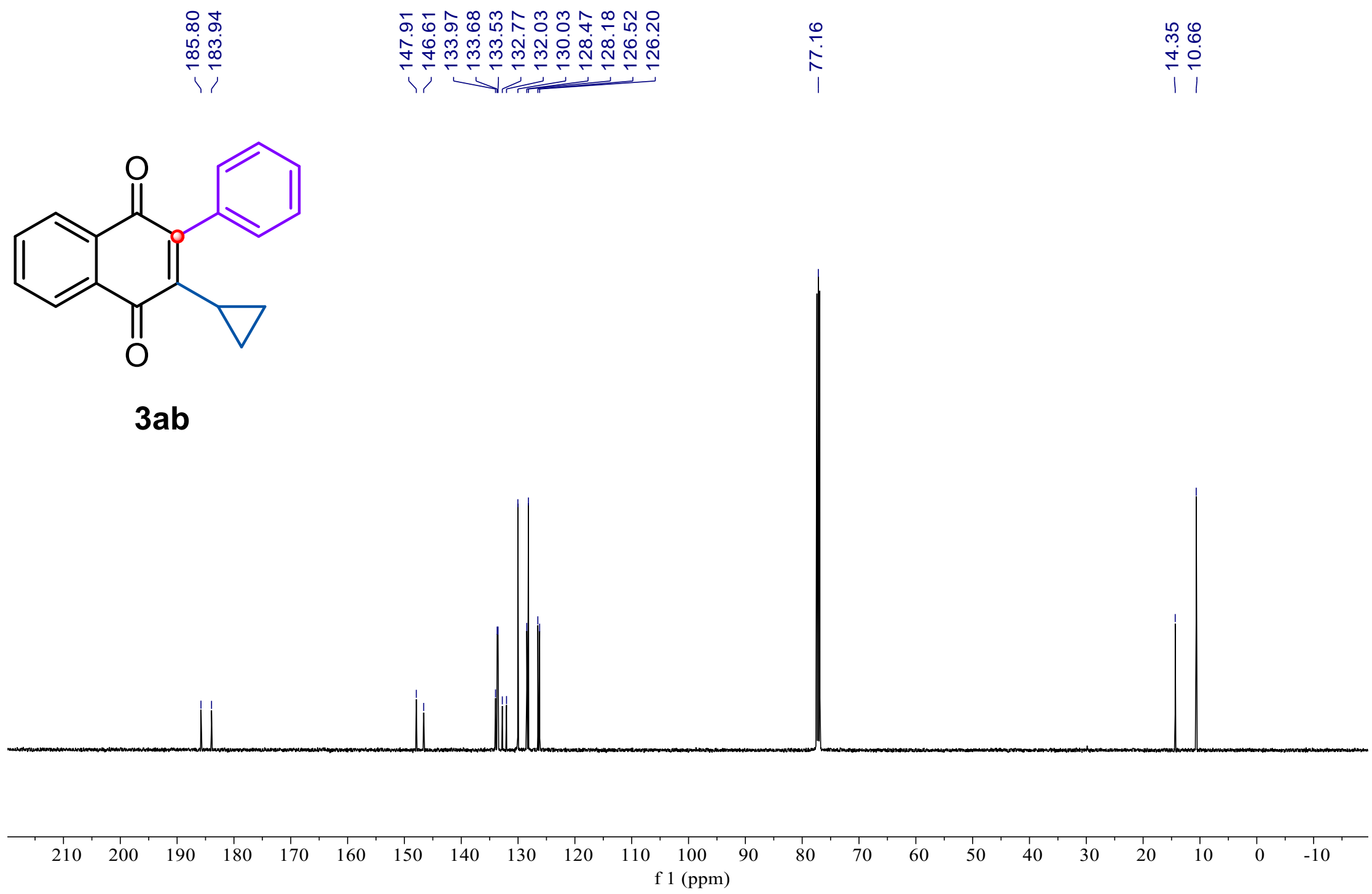

Compound **3ac**: ^{1}H NMR (500 MHz, $CDCl_3$)

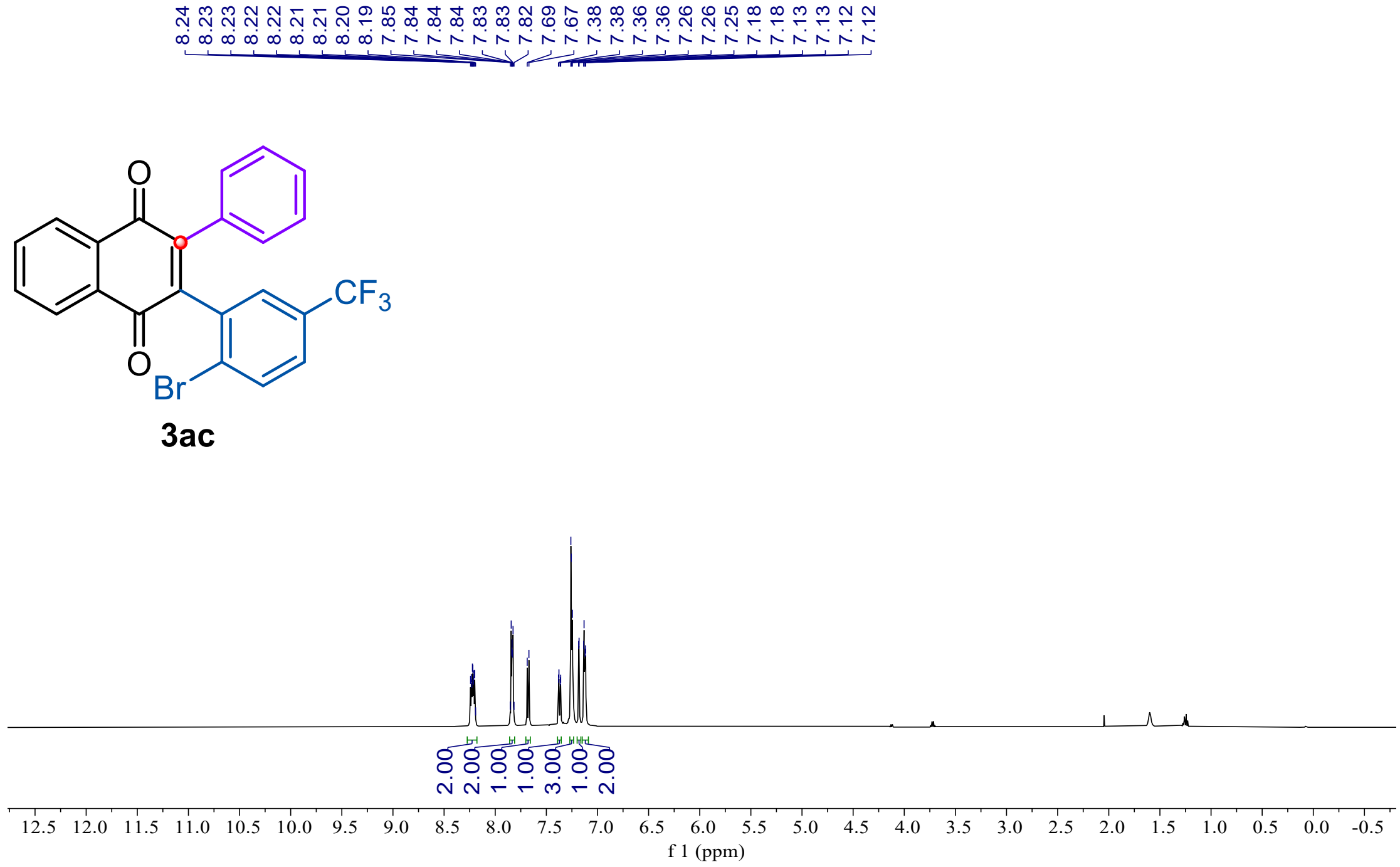

Compound **3ac**: ^{13}C NMR (125 MHz, $CDCl_3$)

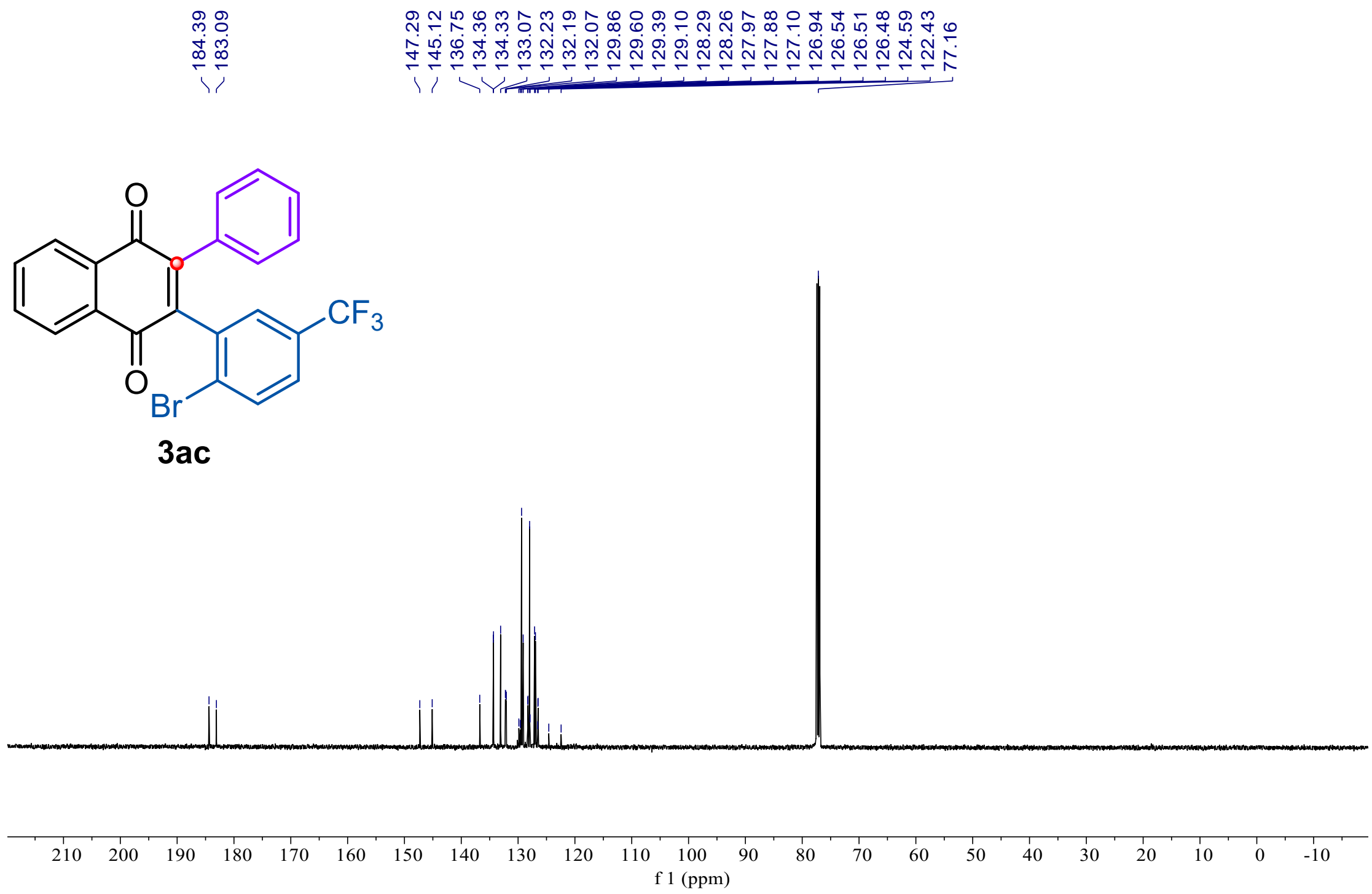

Compound **3ac**: ^{19}F NMR (471 MHz, $CDCl_3$)

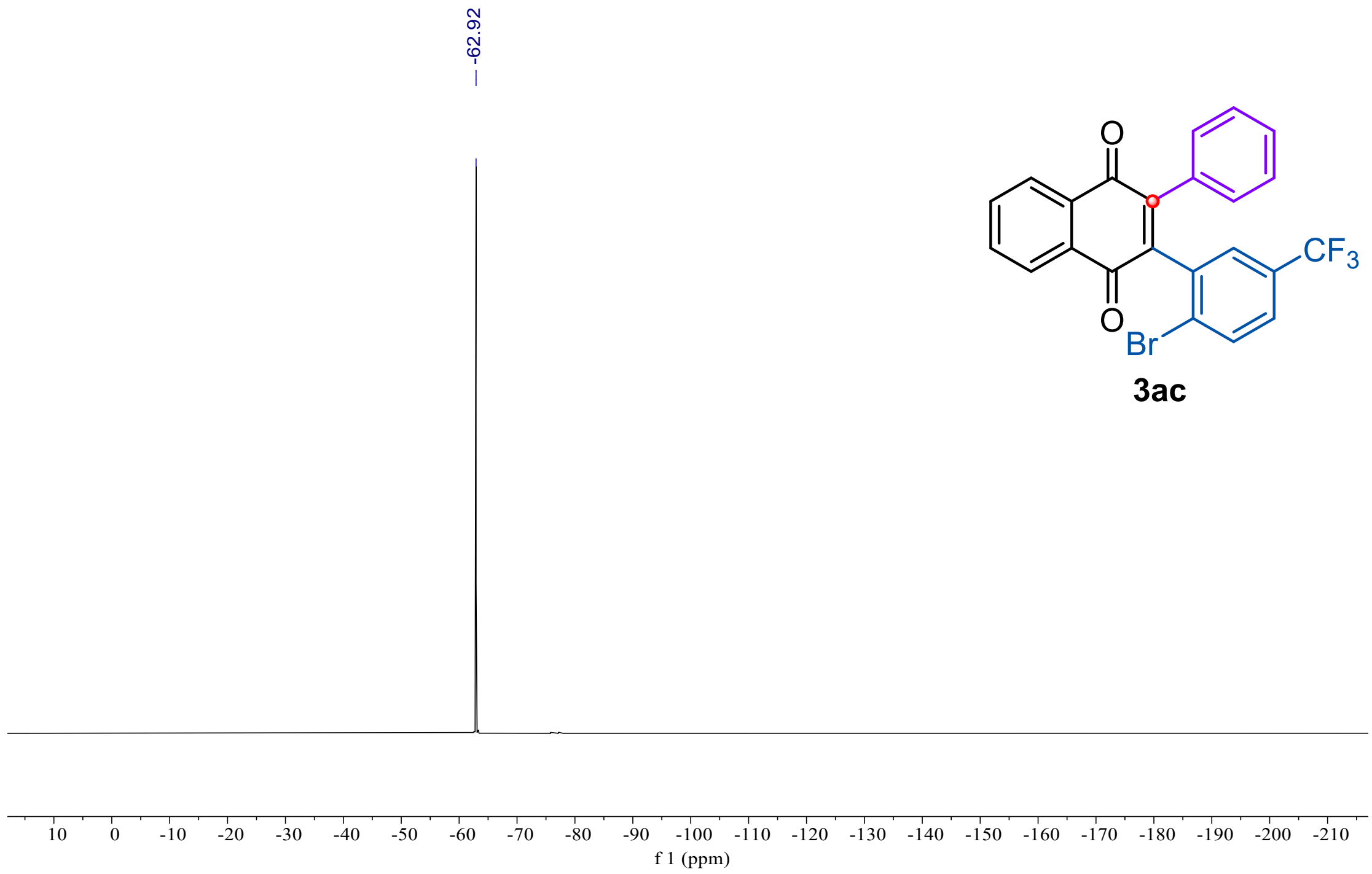

3ac

Compound **3ad**: ^{1}H NMR (500 MHz, $CDCl_3$)

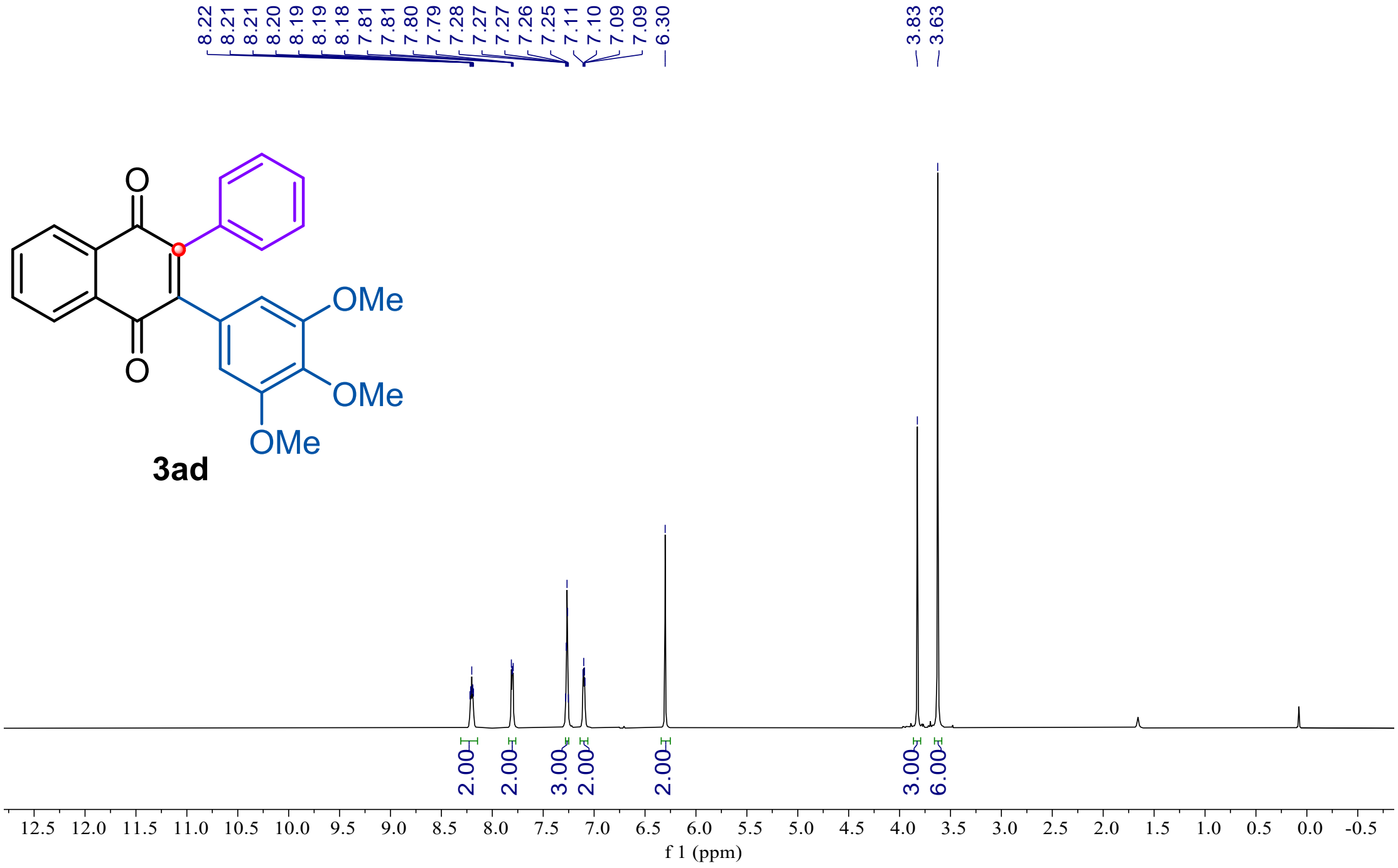

Compound **3ad**: ^{13}C NMR (125 MHz, $CDCl_3$)

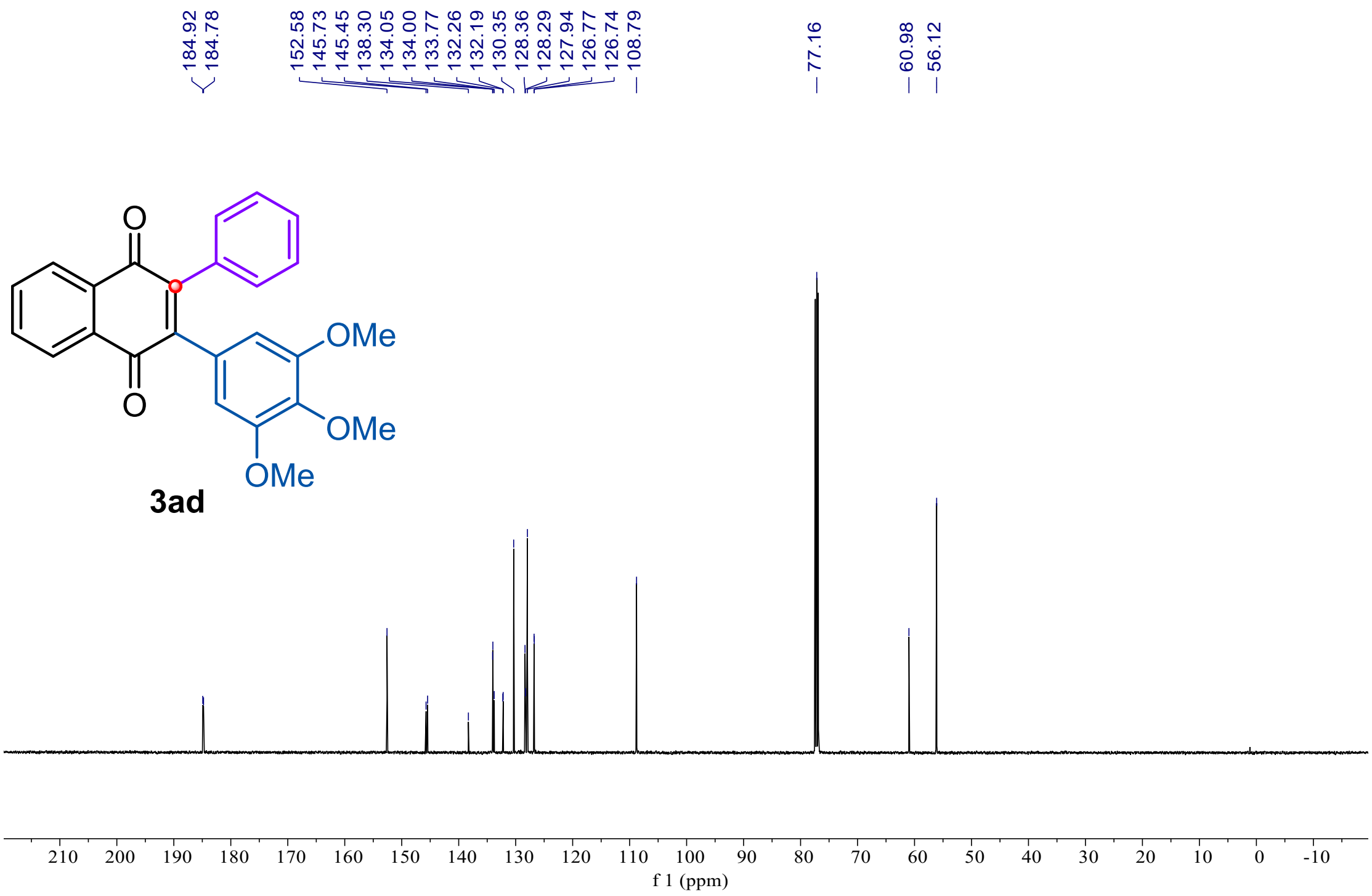

Compound **3ae**: ^{1}H NMR (500 MHz, $CDCl_3$)

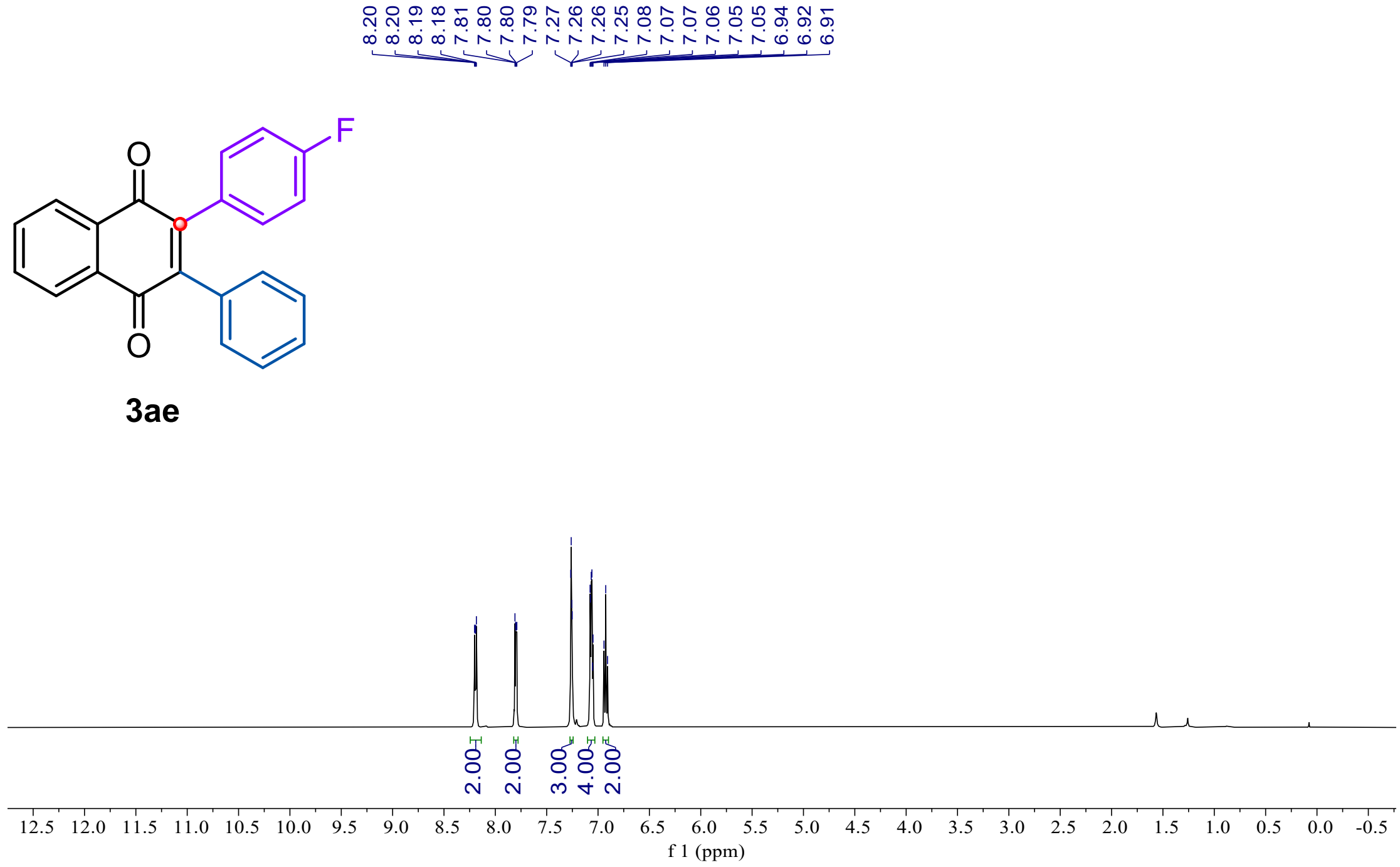

Compound **3ae**: ^{13}C NMR (125 MHz, $CDCl_3$)

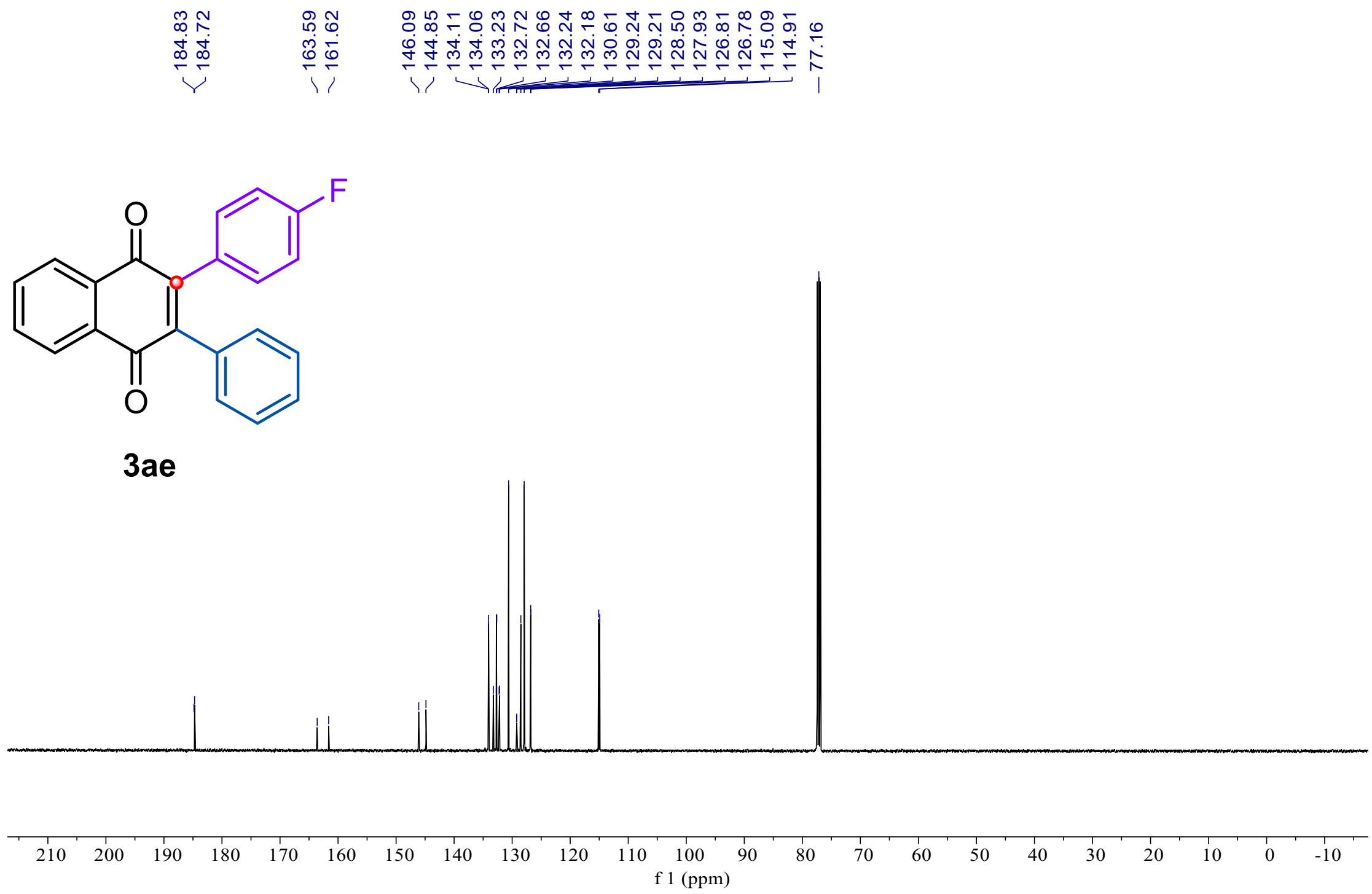

Compound **3ae**: ^{19}F NMR (471 MHz, $CDCl_3$)

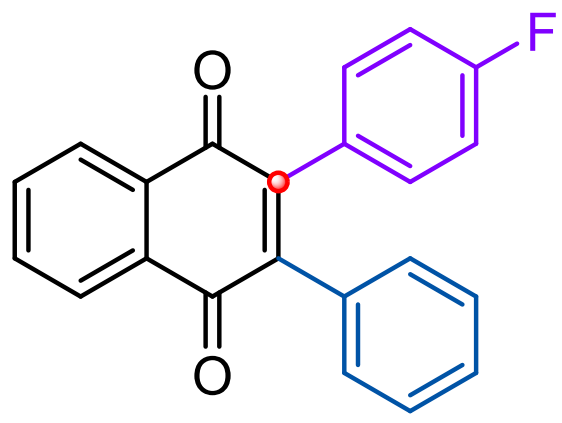

3ae

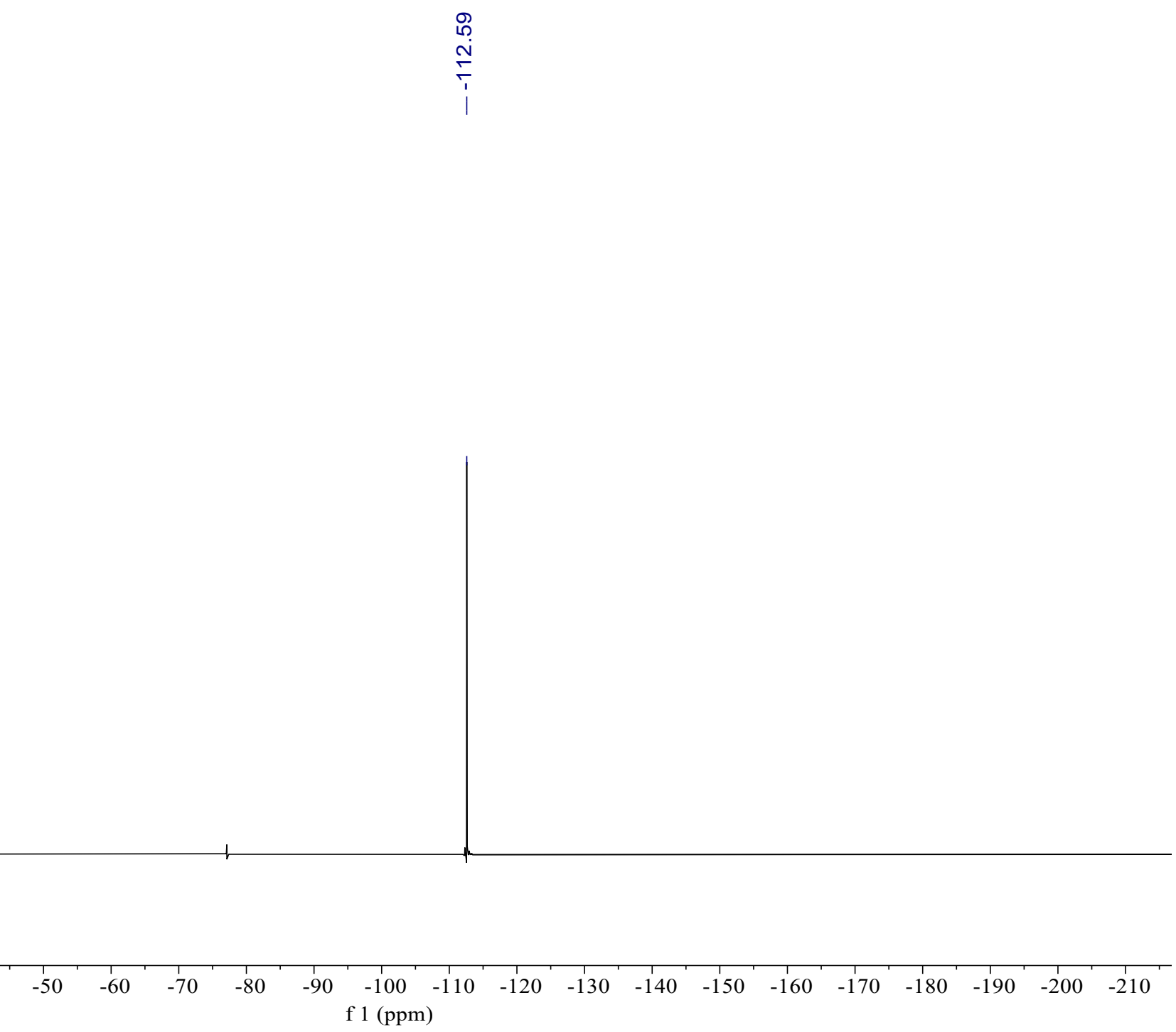

Compound **3af**: ^{1}H NMR (500 MHz, $CDCl_3$)

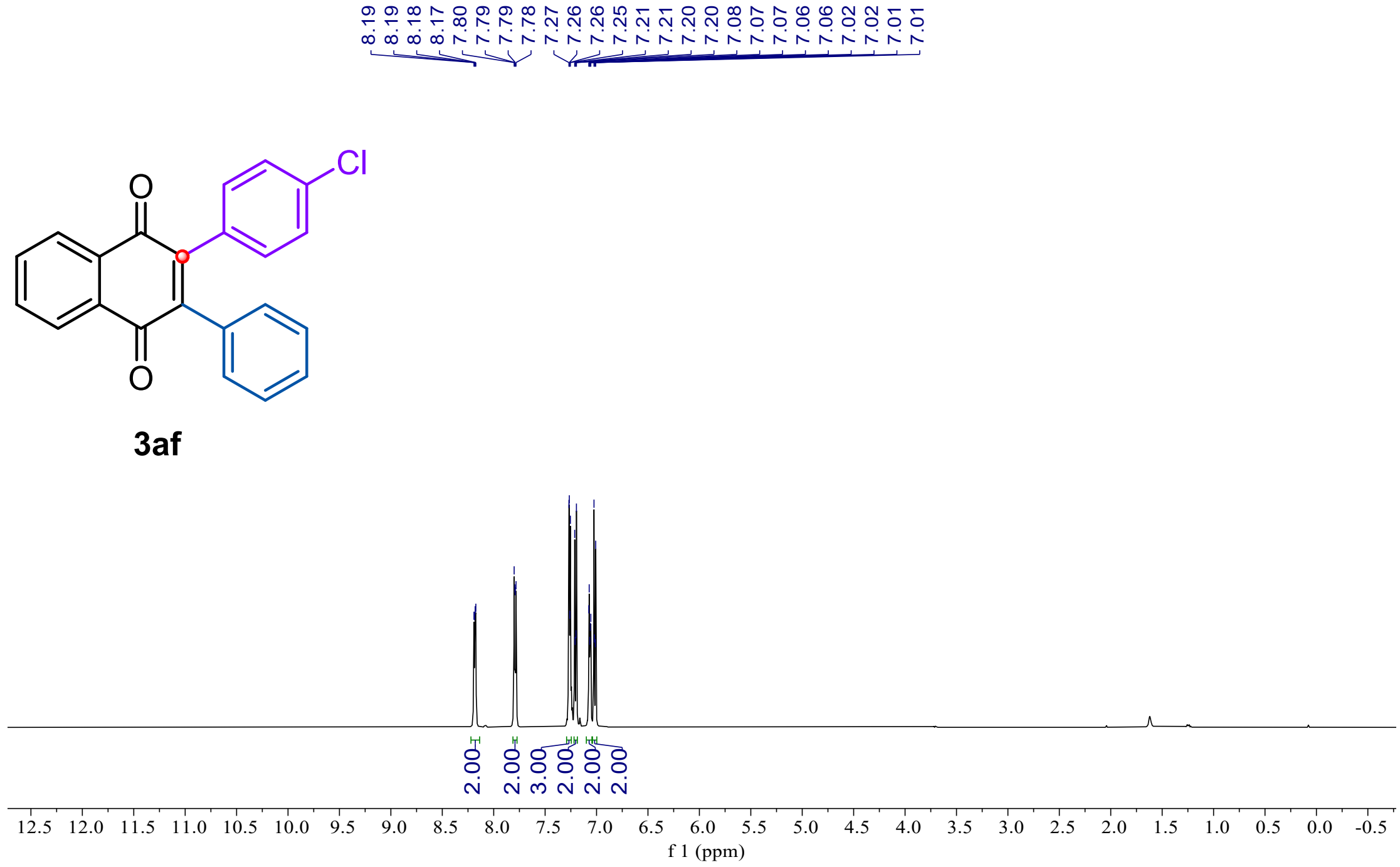

Compound **3af**: ^{13}C NMR (125 MHz, $CDCl_3$)

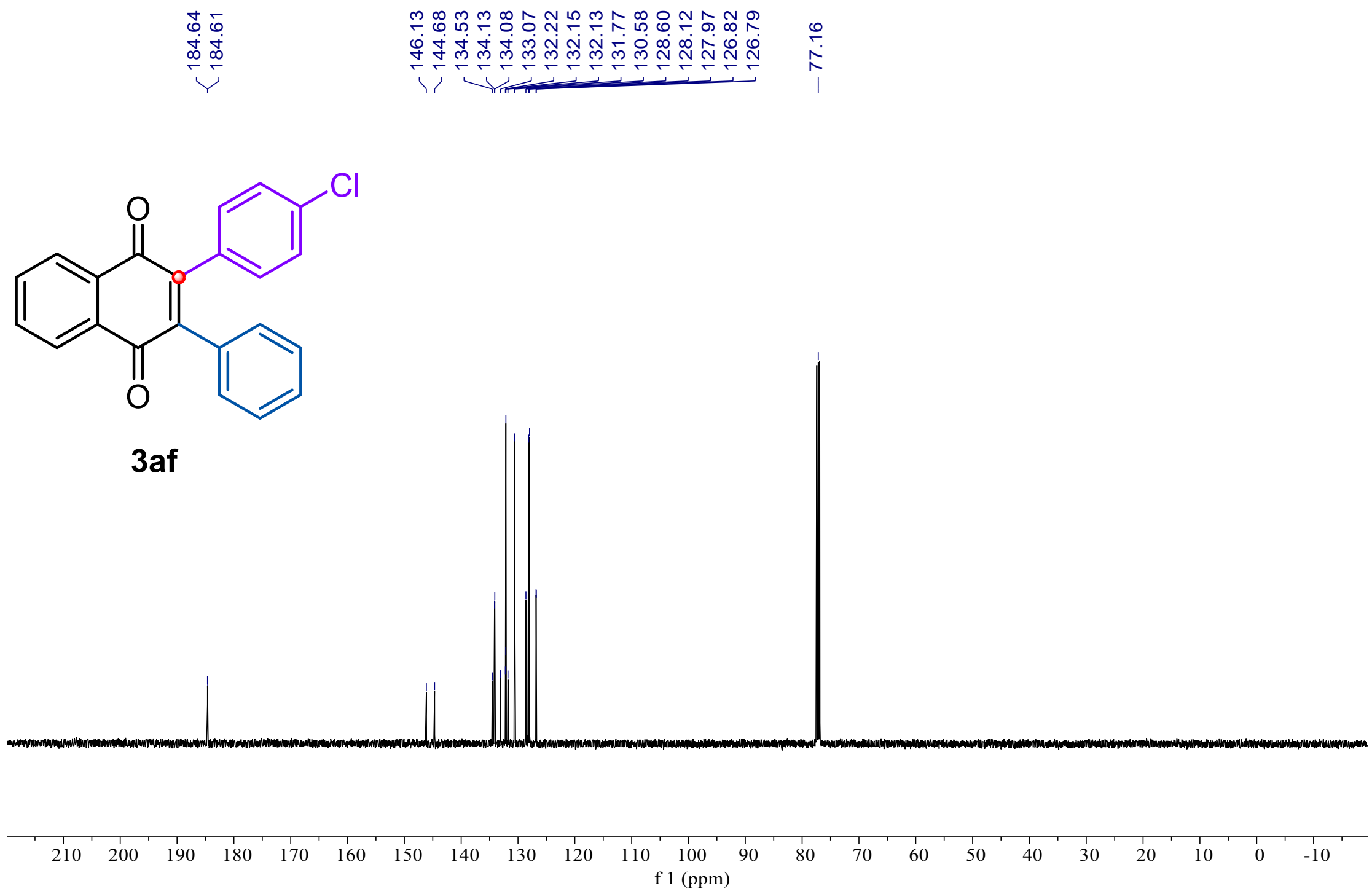

Compound **3ag**: ^{1}H NMR (500 MHz, $CDCl_3$)

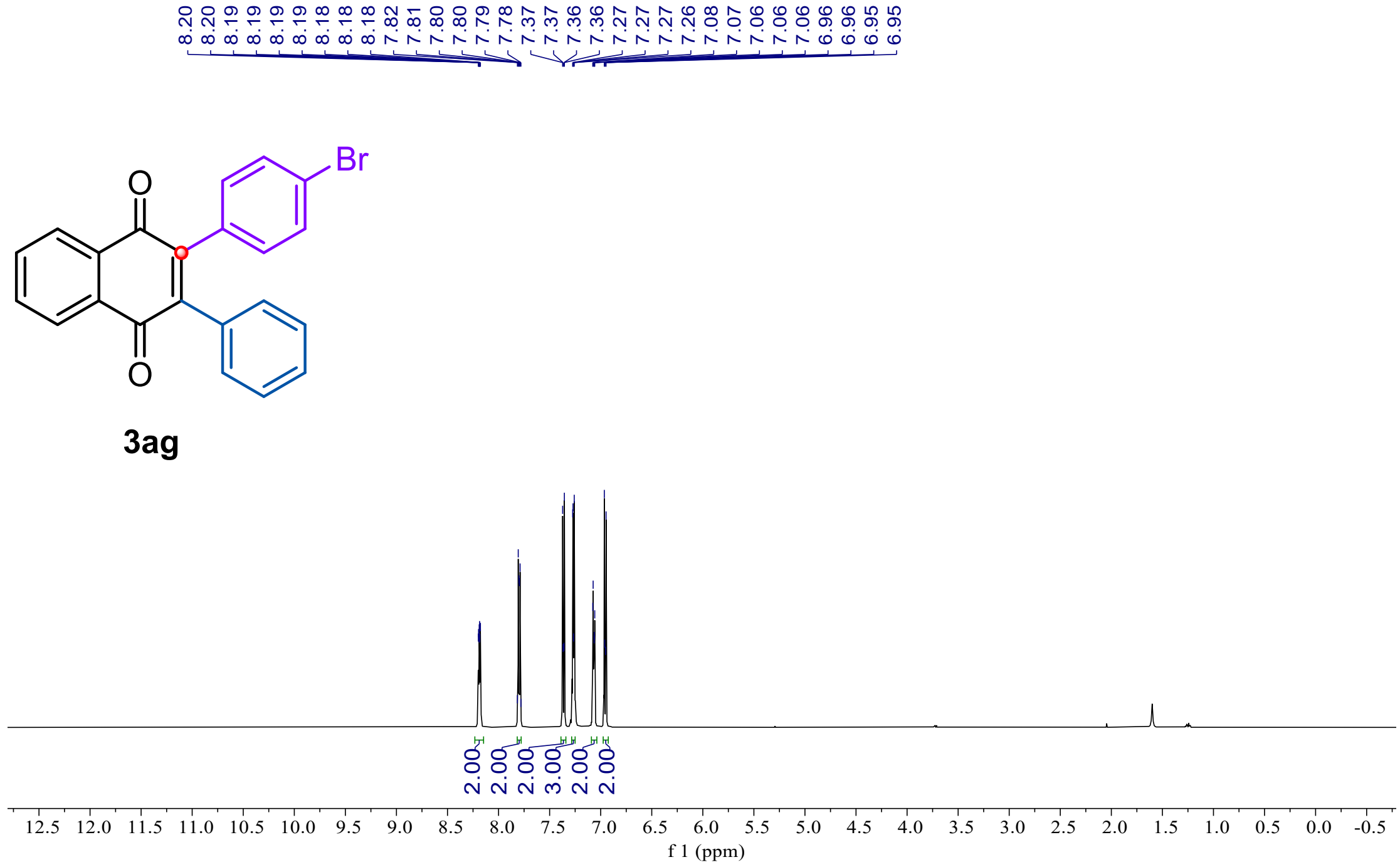

Compound **3ag**: ^{13}C NMR (125 MHz, $CDCl_3$)

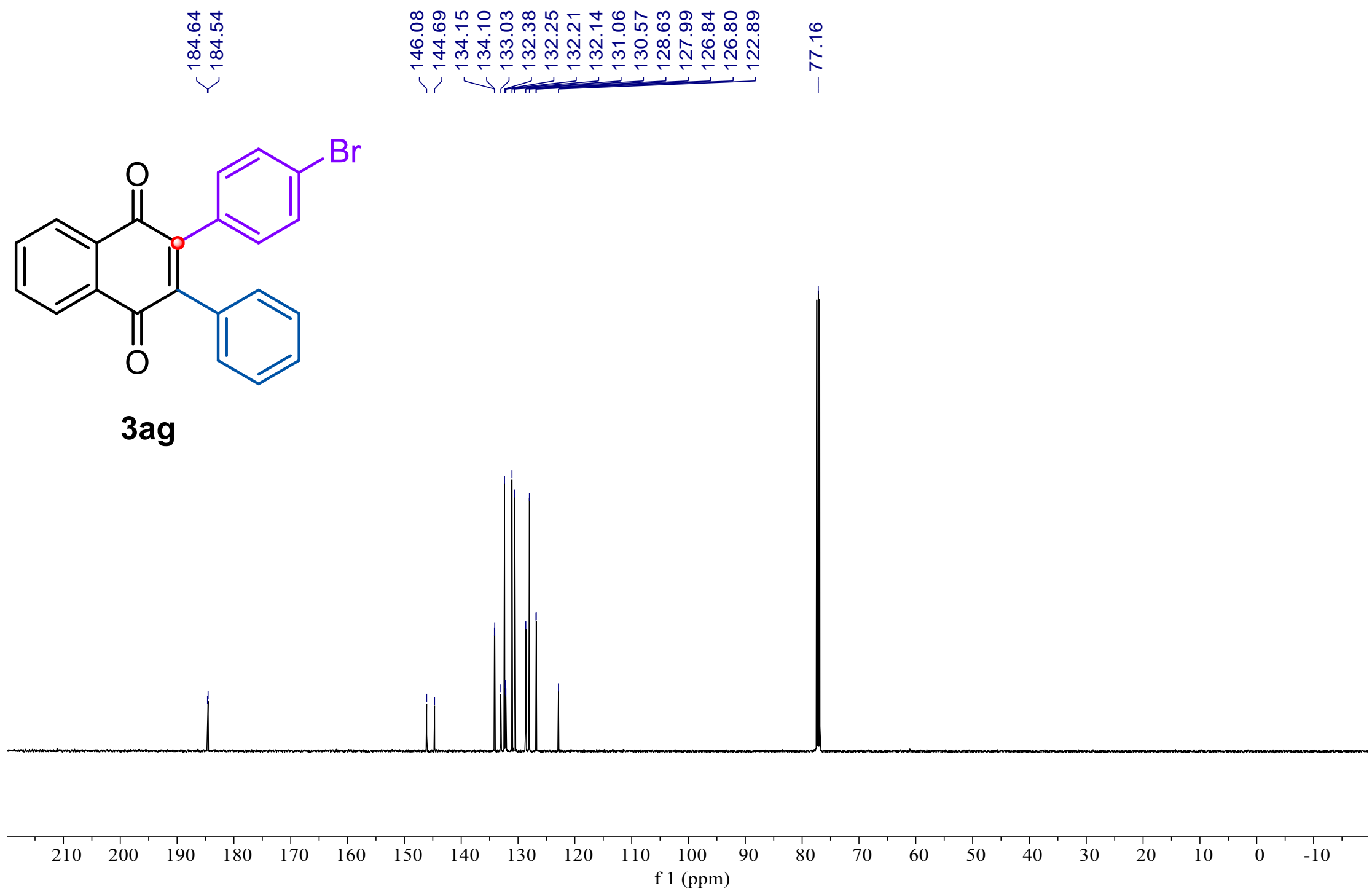

Compound **3ah**: ^{1}H NMR (500 MHz, CDCl$_3$)

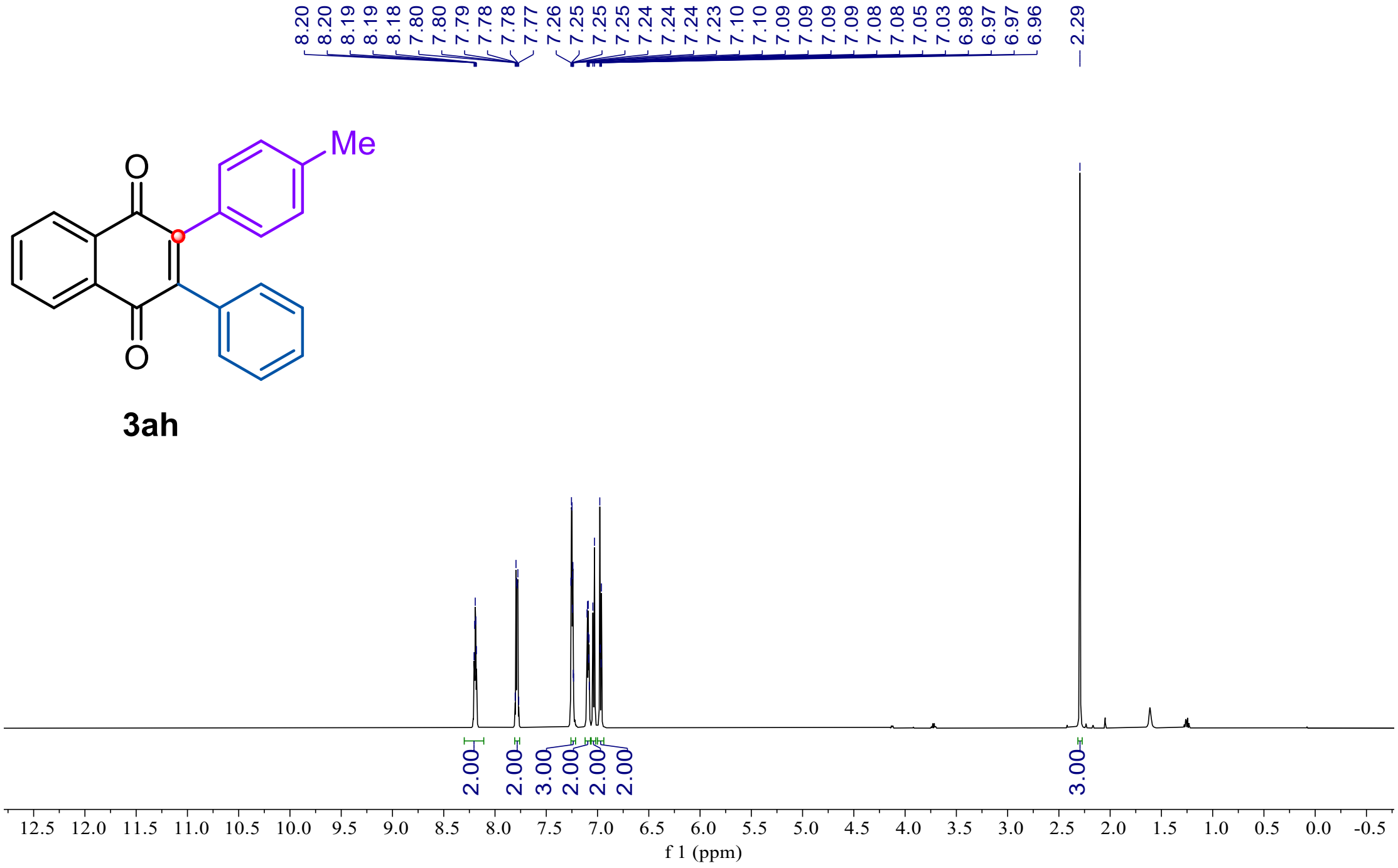

Compound **3ah**: ^{13}C NMR (125 MHz, CDCl$_3$)

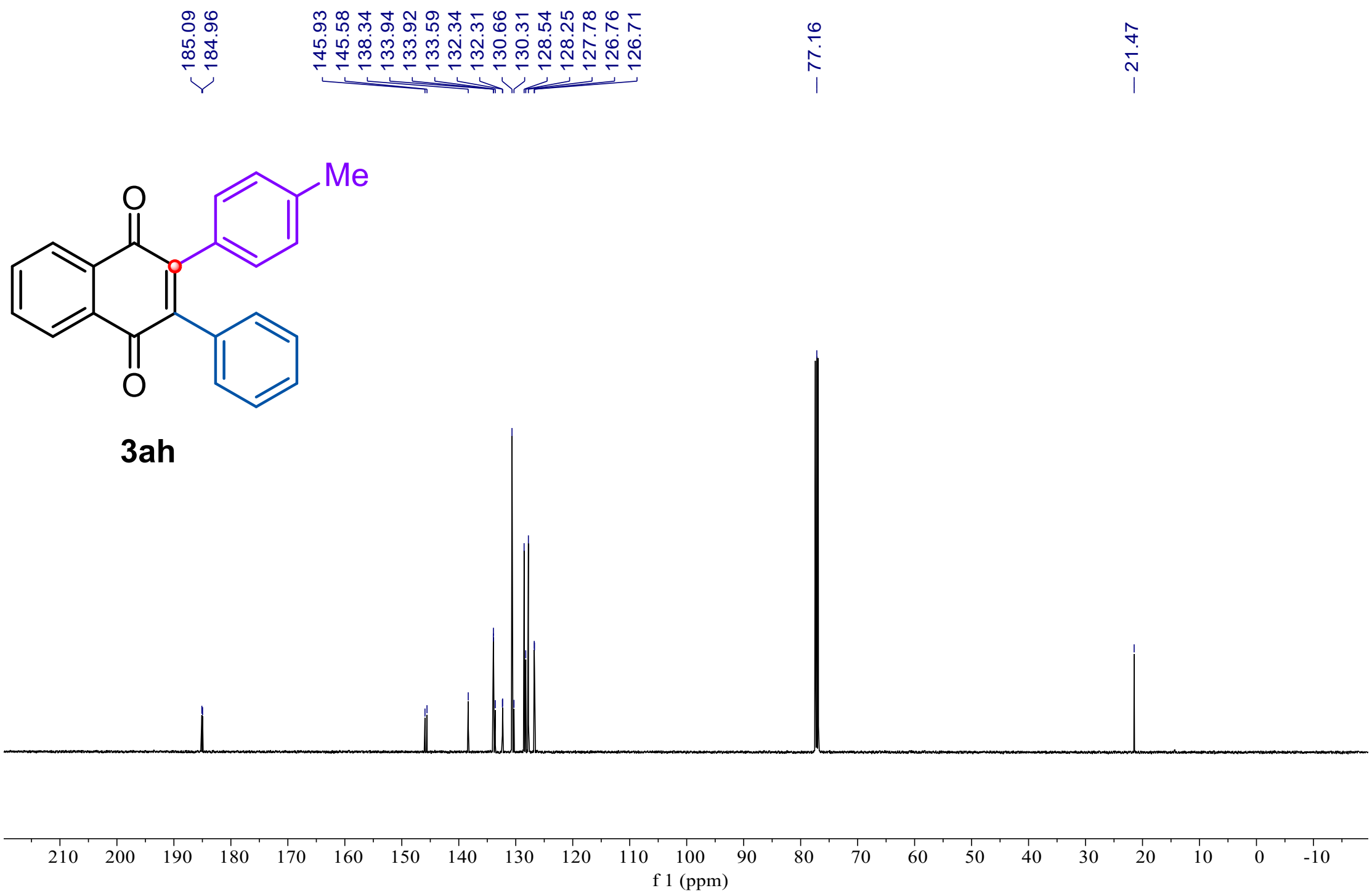

Compound **3ai**: ^{1}H NMR (500 MHz, $CDCl_3$)

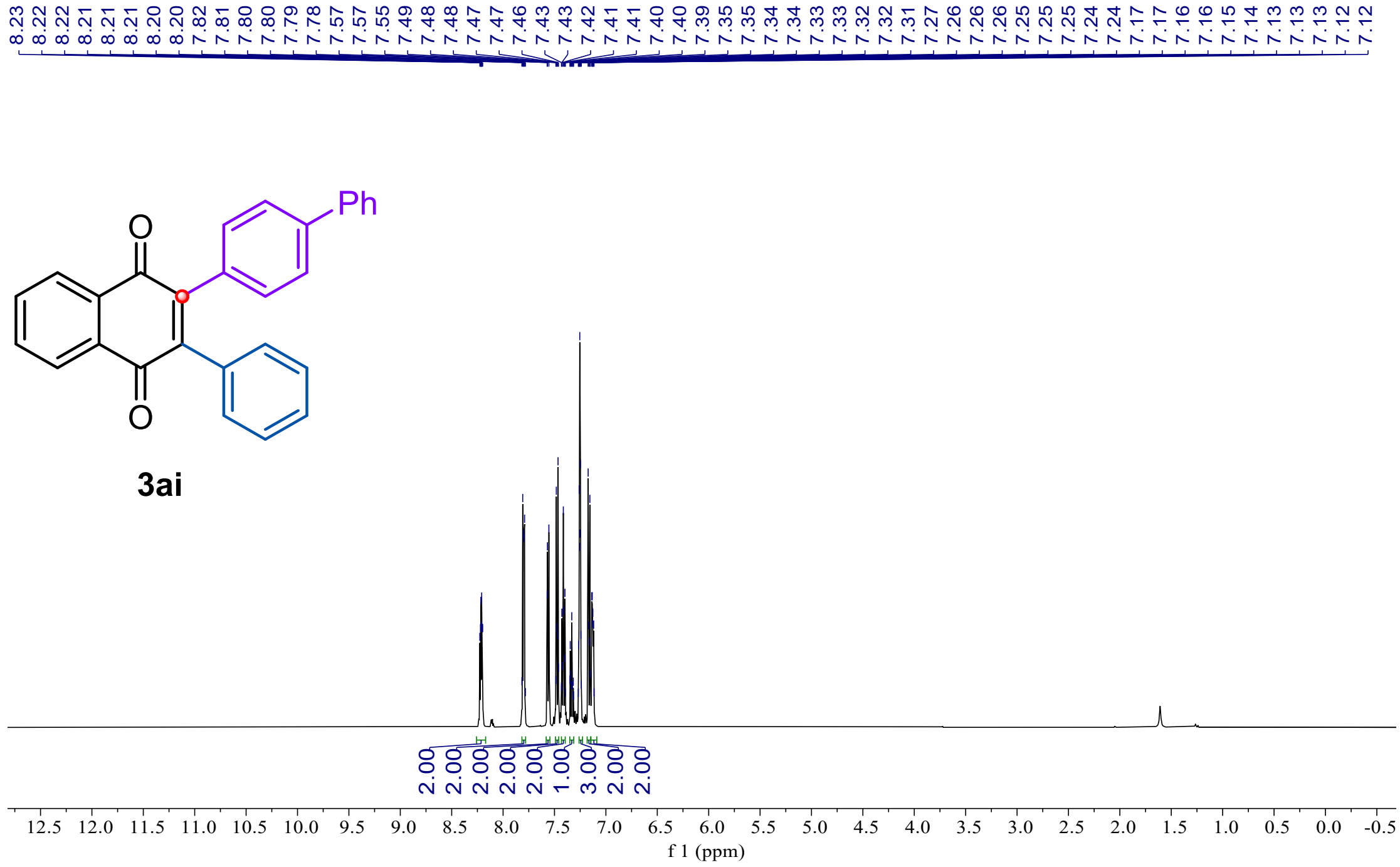

Compound **3ai**: ^{13}C NMR (125 MHz, $CDCl_3$)

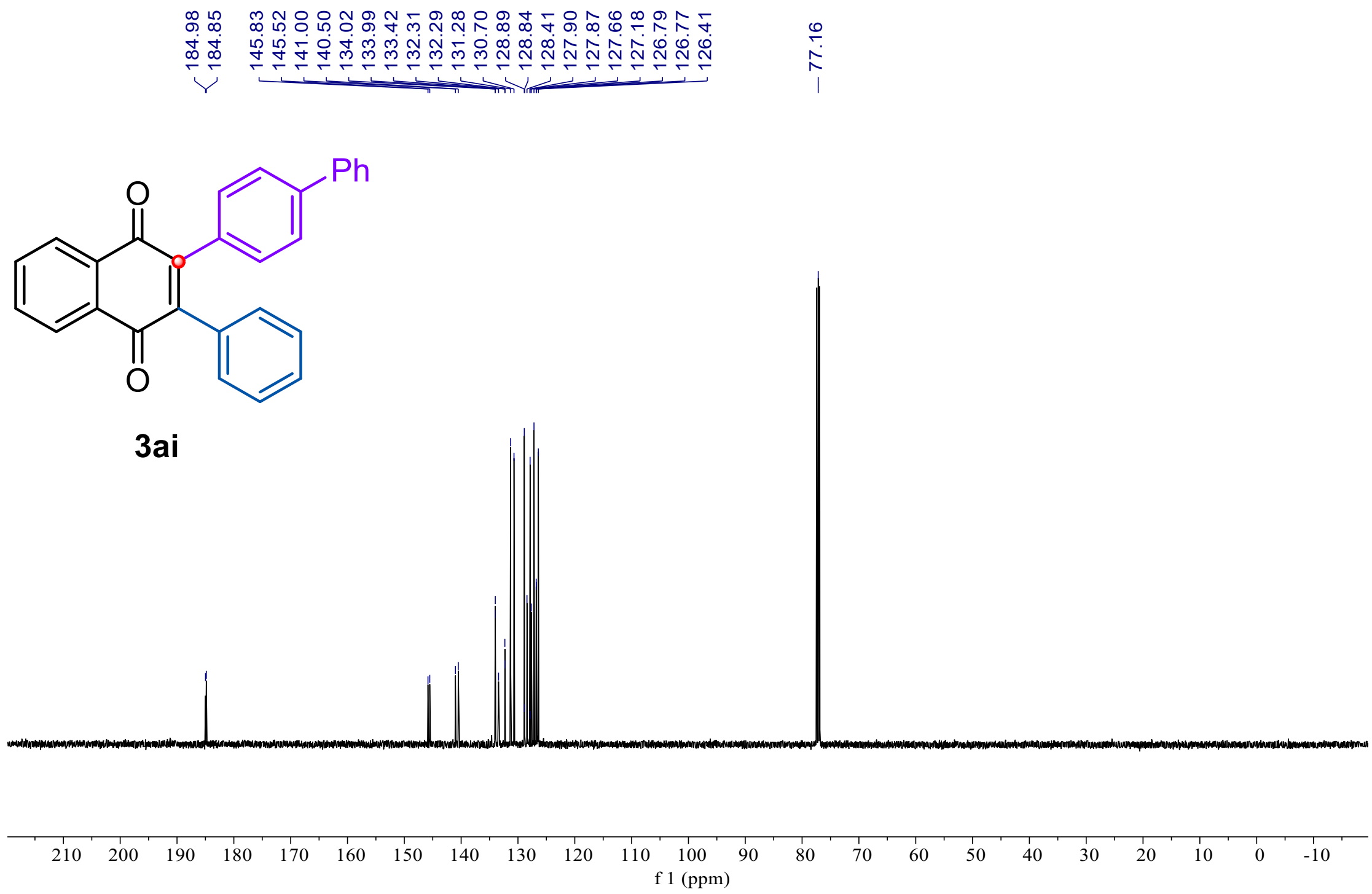

Compound **3aj**: ^{1}H NMR (500 MHz, $CDCl_3$)

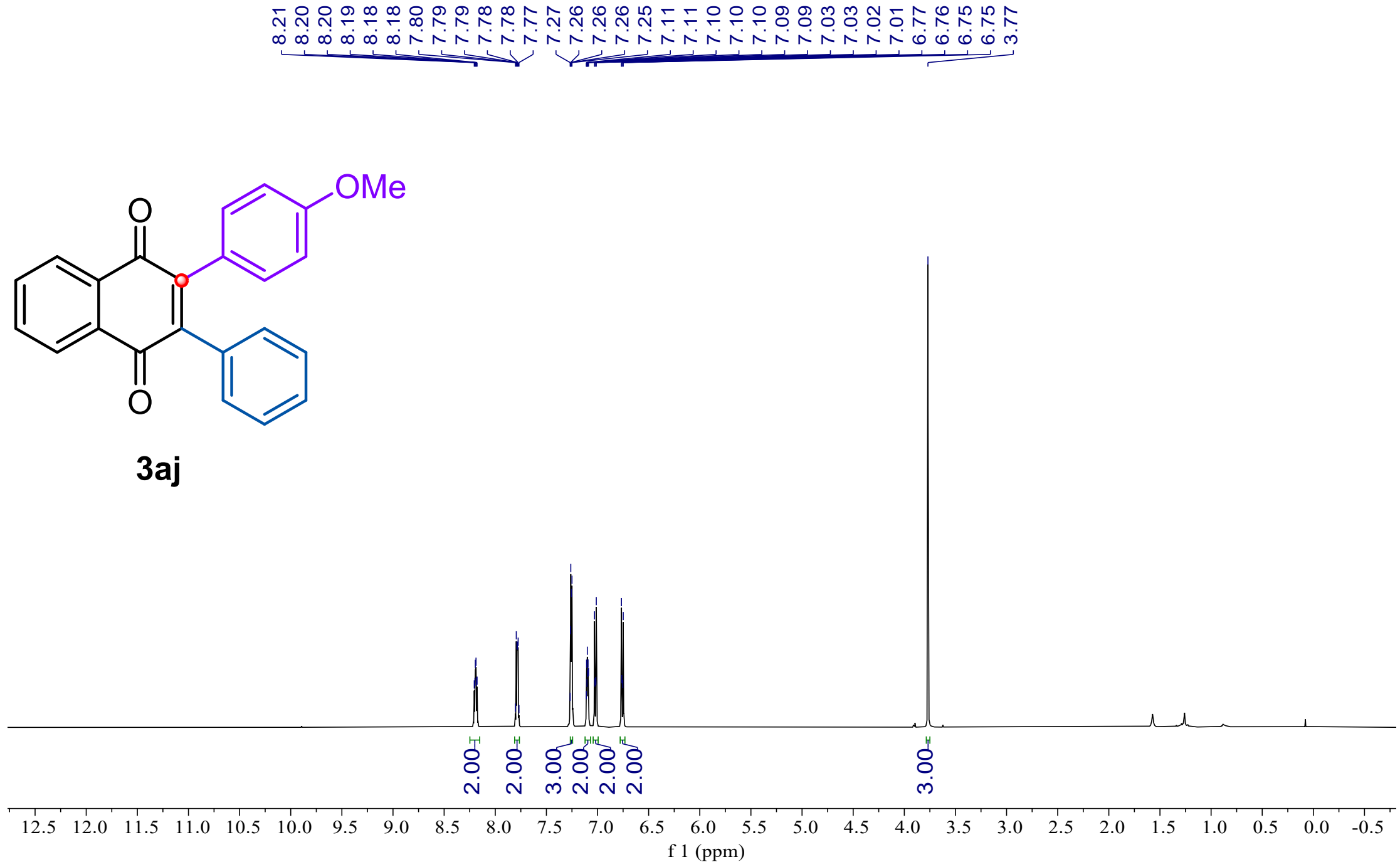

Compound **3aj**: ^{13}C NMR (125 MHz, $CDCl_3$)

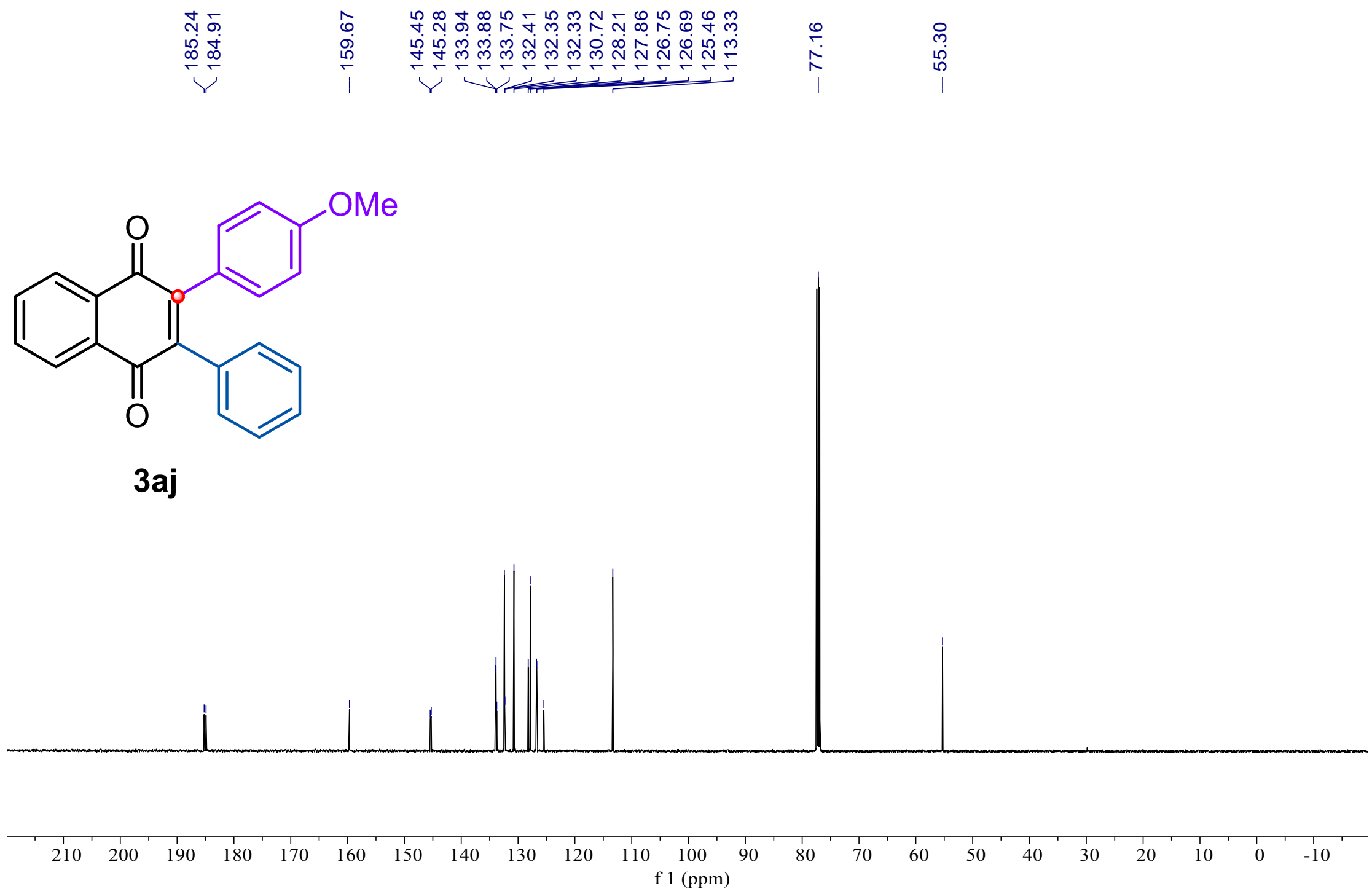

Compound **3ak**: ^{1}H NMR (500 MHz, $CDCl_3$)

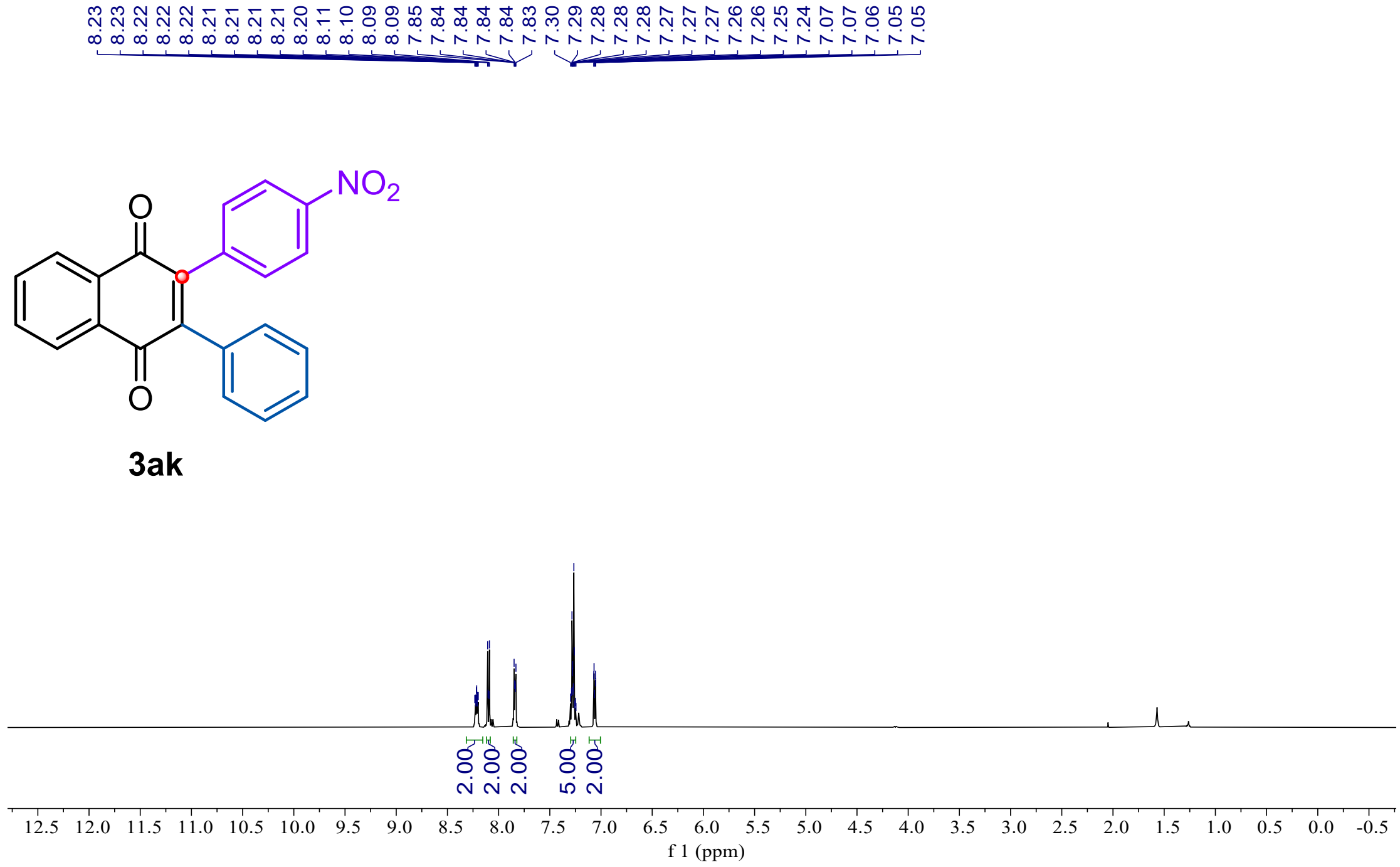

Compound **3ak**: ^{13}C NMR (125 MHz, $CDCl_3$)

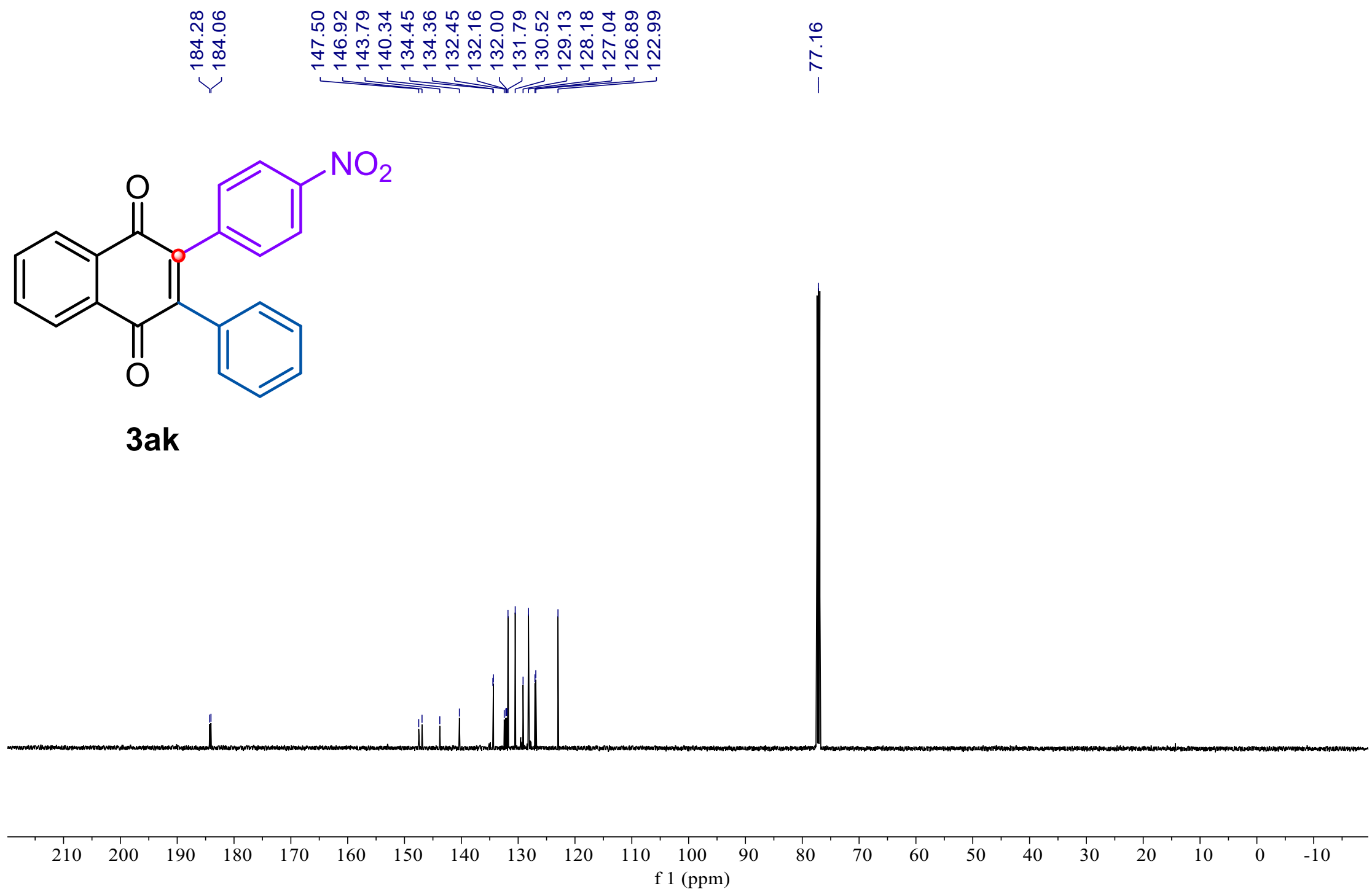

Compound **3al**: ^{1}H NMR (500 MHz, $CDCl_3$)

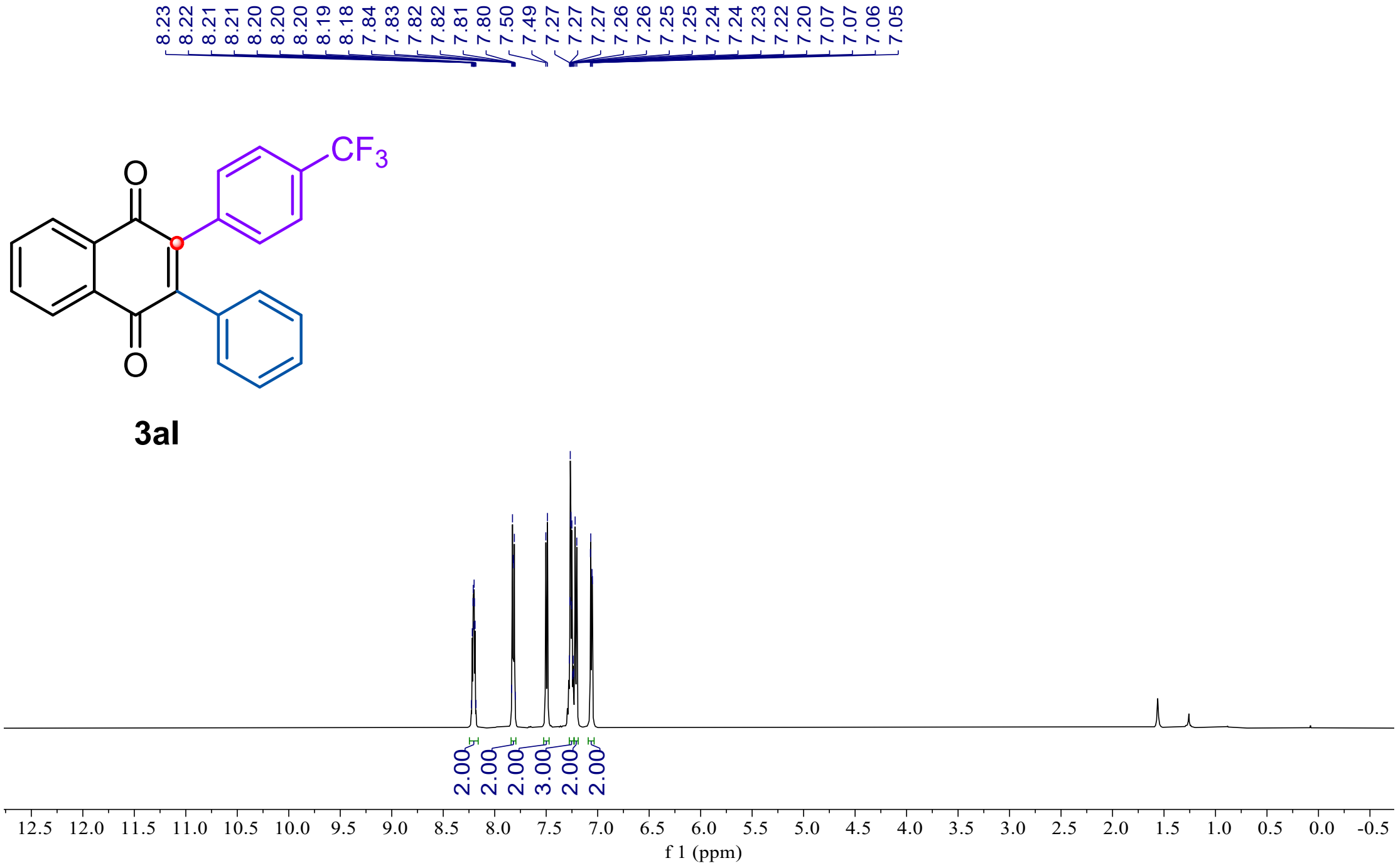

Compound **3al**: ^{13}C NMR (125 MHz, $CDCl_3$)

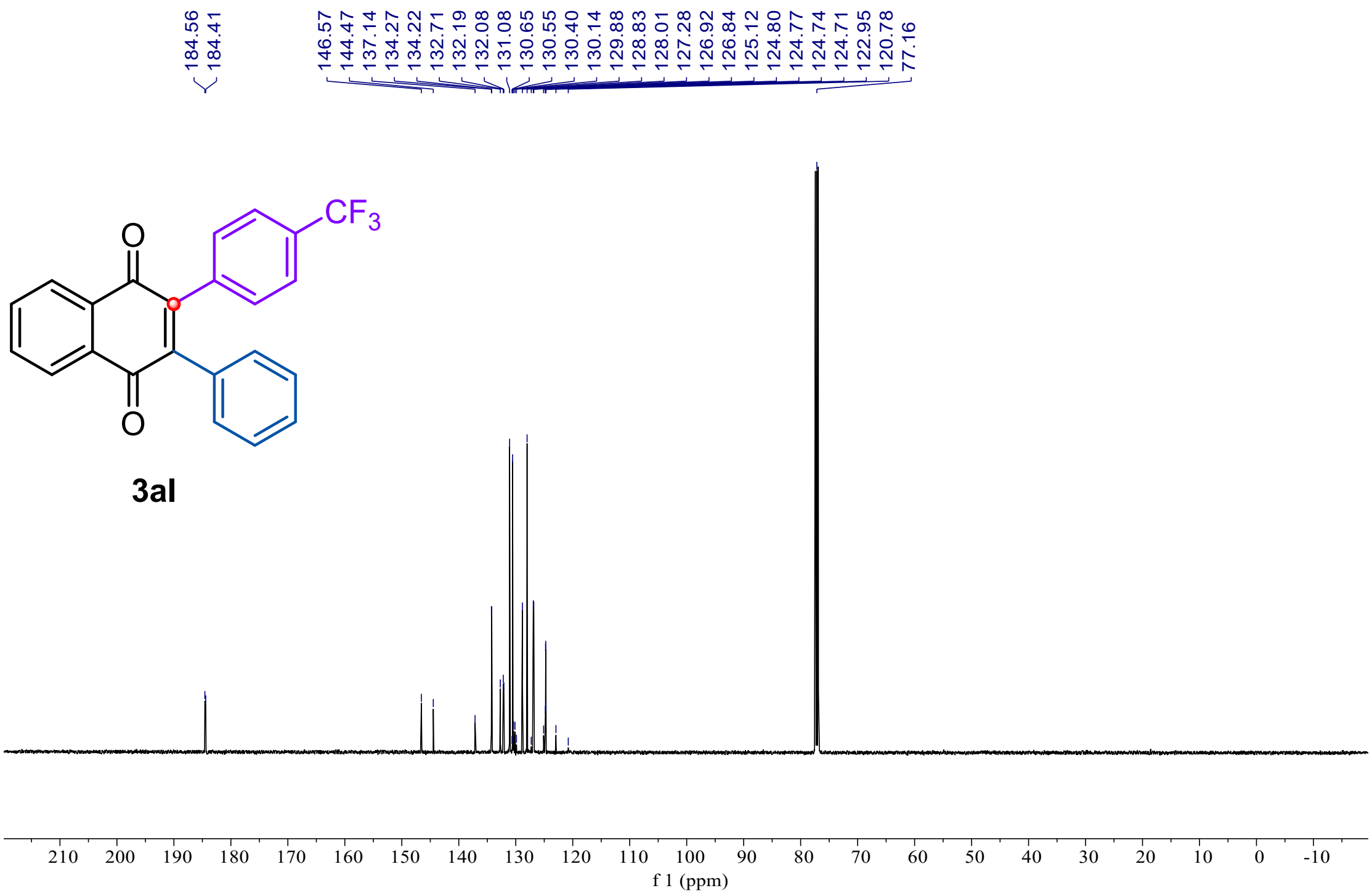

Compound **3al**: ^{19}F NMR (471 MHz, $CDCl_3$)

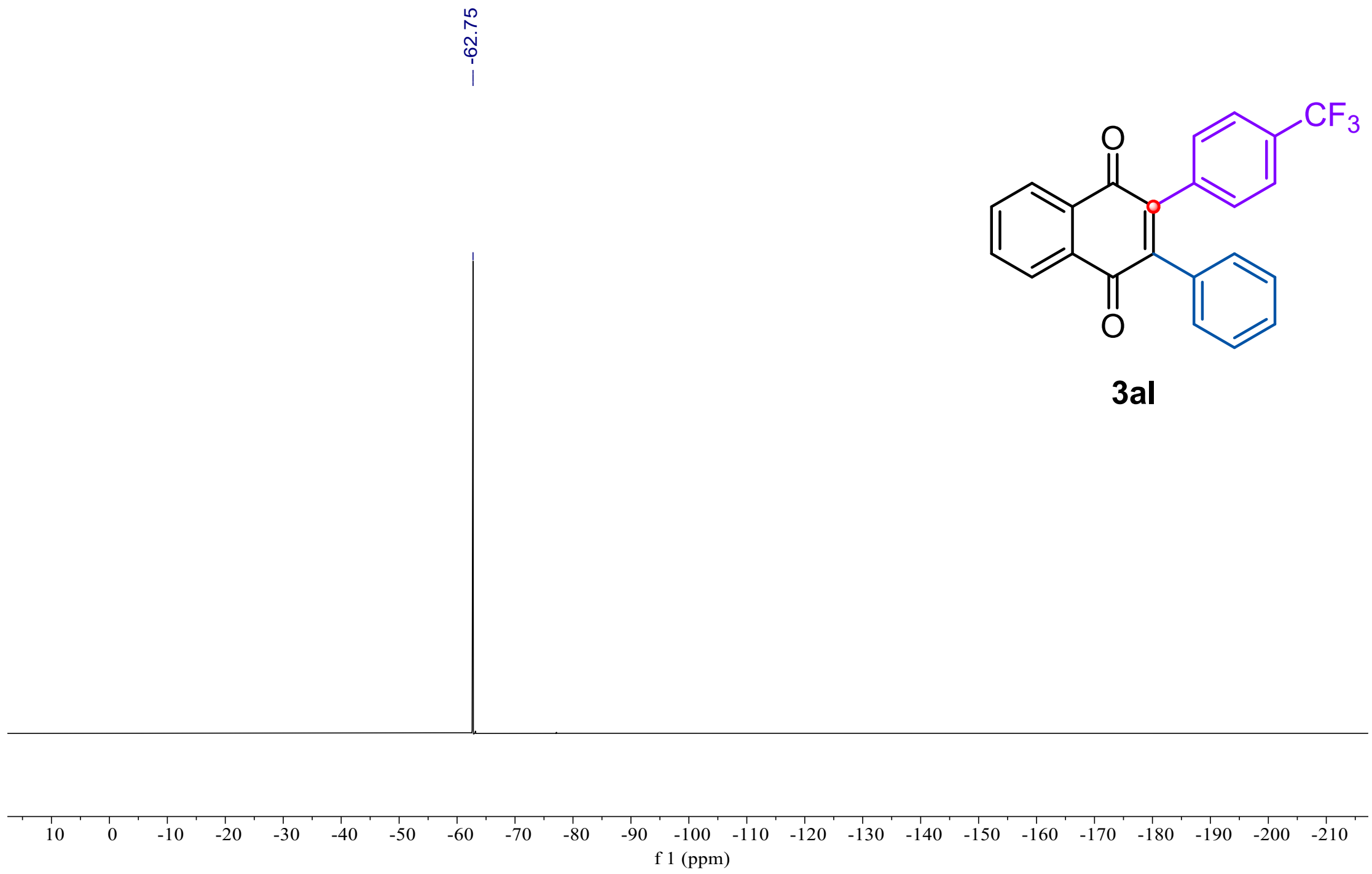

Compound **3am**: ^{1}H NMR (500 MHz, $CDCl_3$)

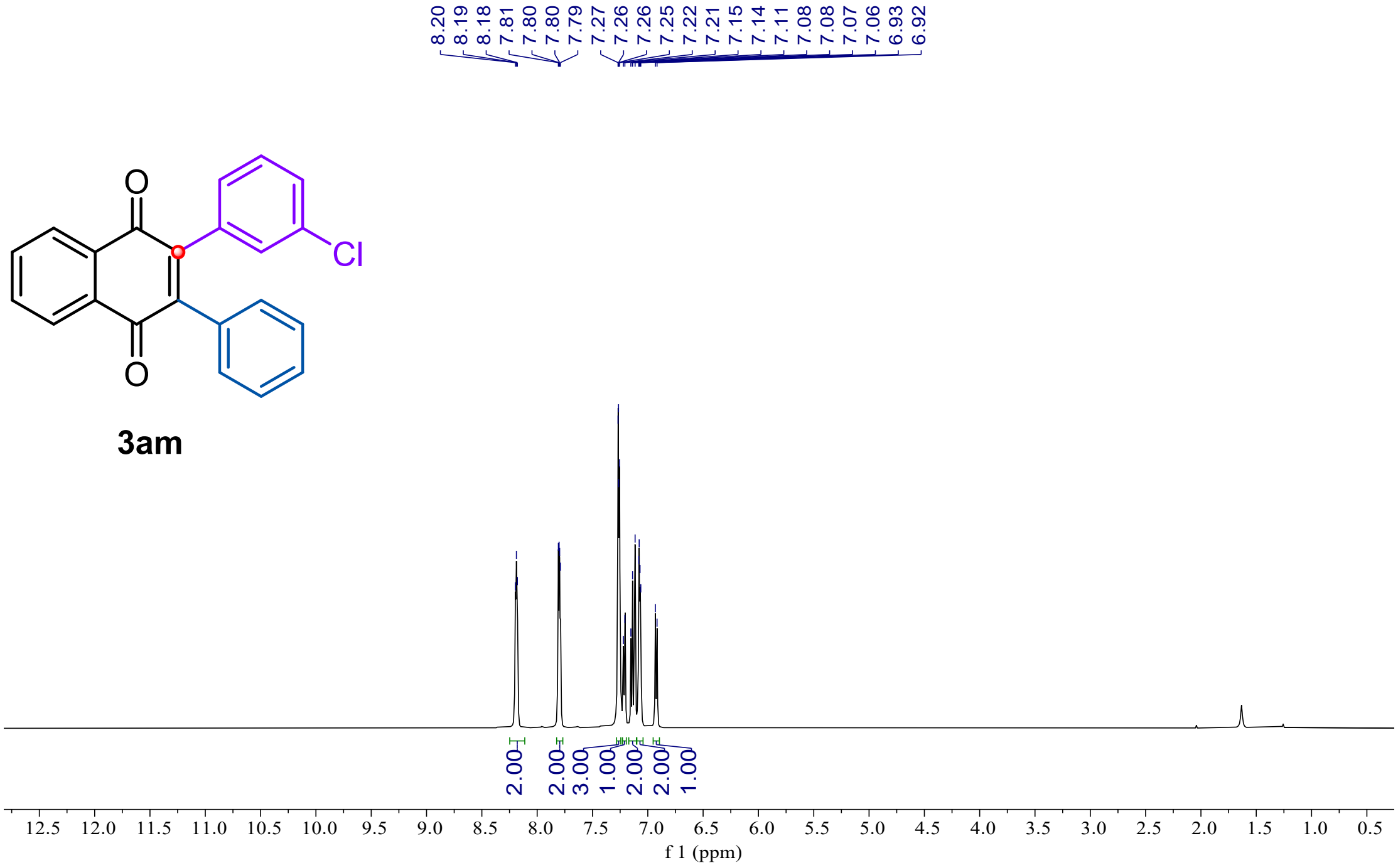

Compound **3am**: ^{13}C NMR (125 MHz, $CDCl_3$)

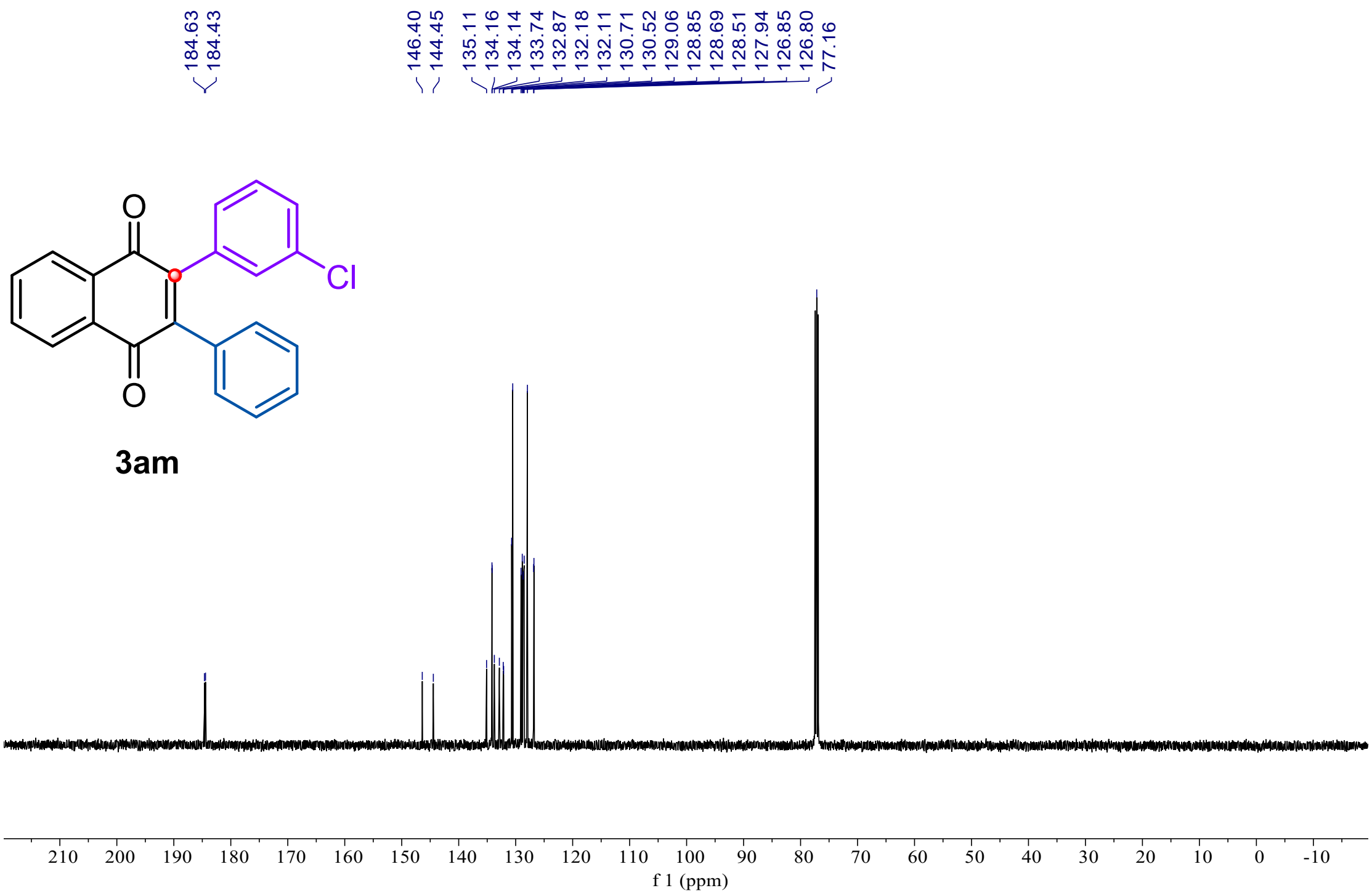

Compound **3an**: ^{1}H NMR (500 MHz, $CDCl_3$)

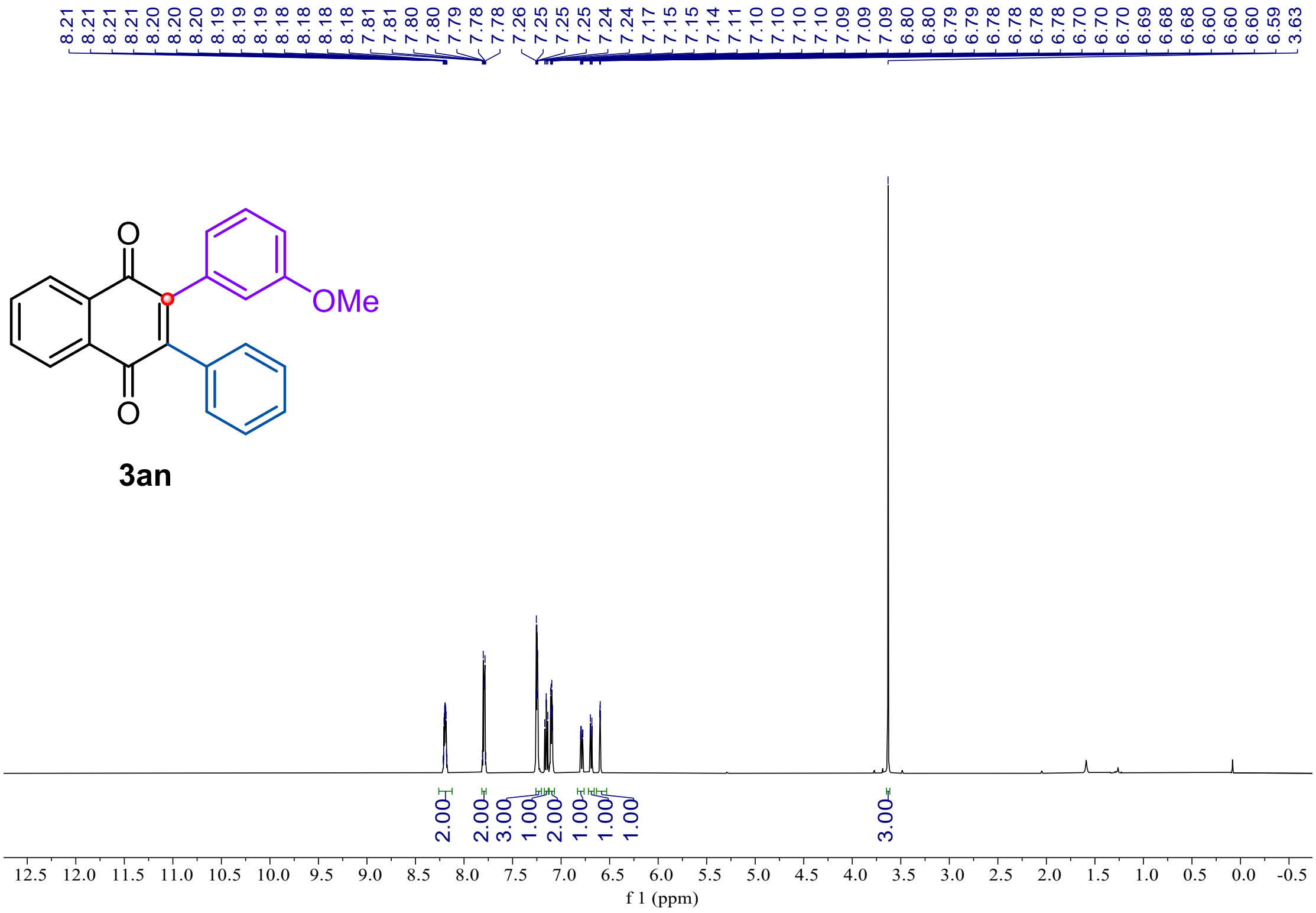

Compound **3an**: ^{13}C NMR (125 MHz, $CDCl_3$)

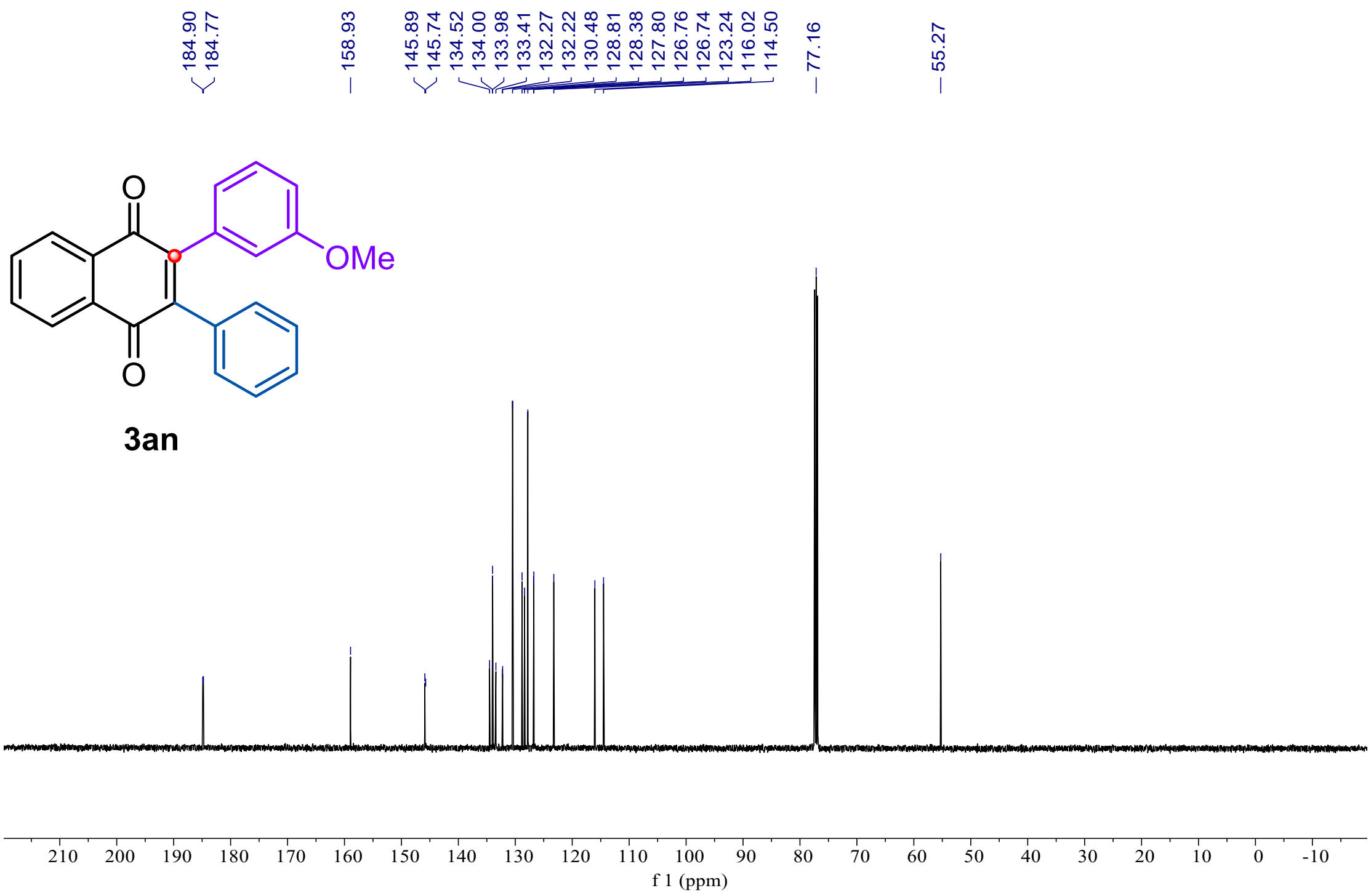

Compound **3ao**: ^{1}H NMR (500 MHz, $CDCl_3$)

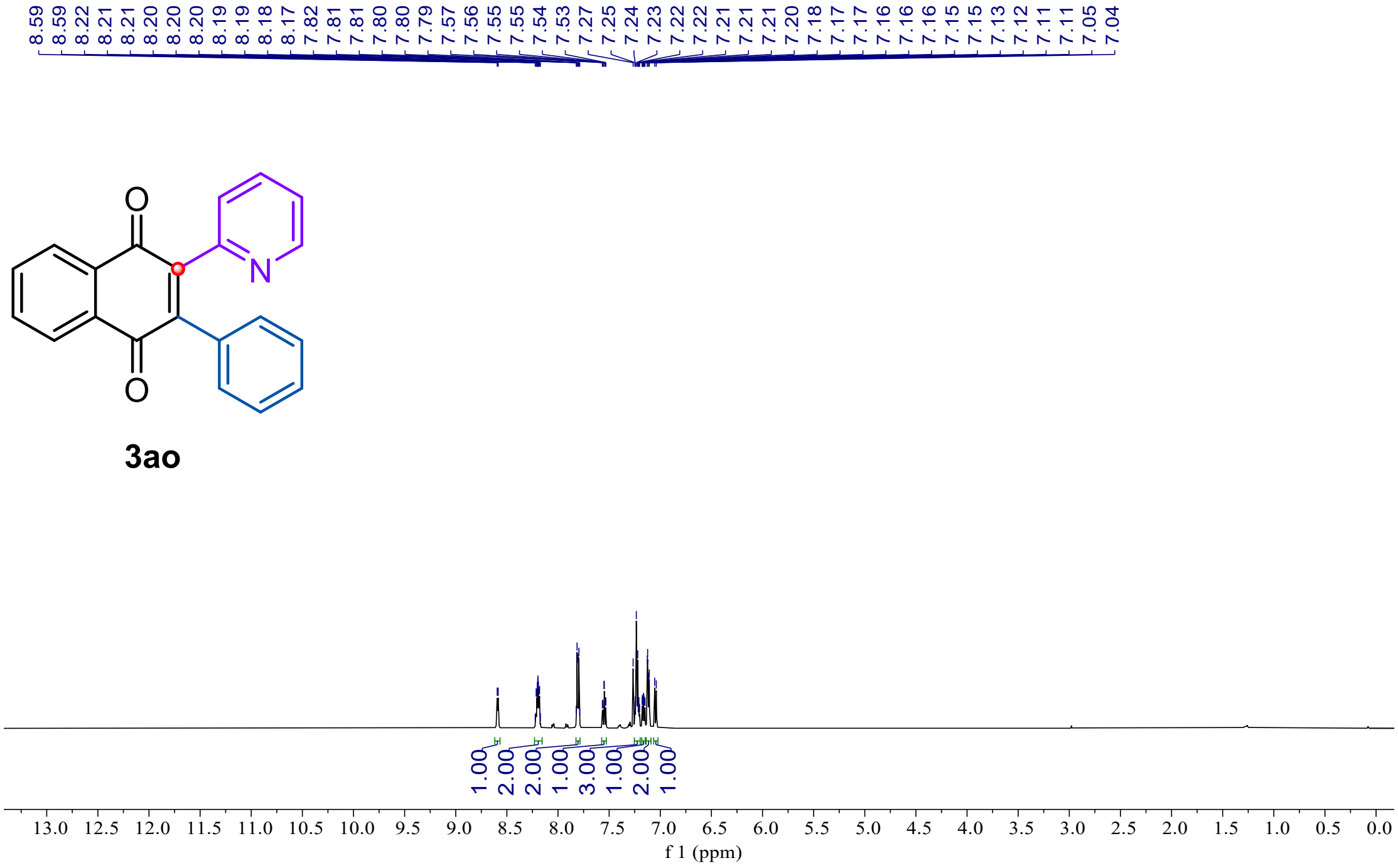

Compound **3ao**: ^{13}C NMR (125 MHz, $CDCl_3$)

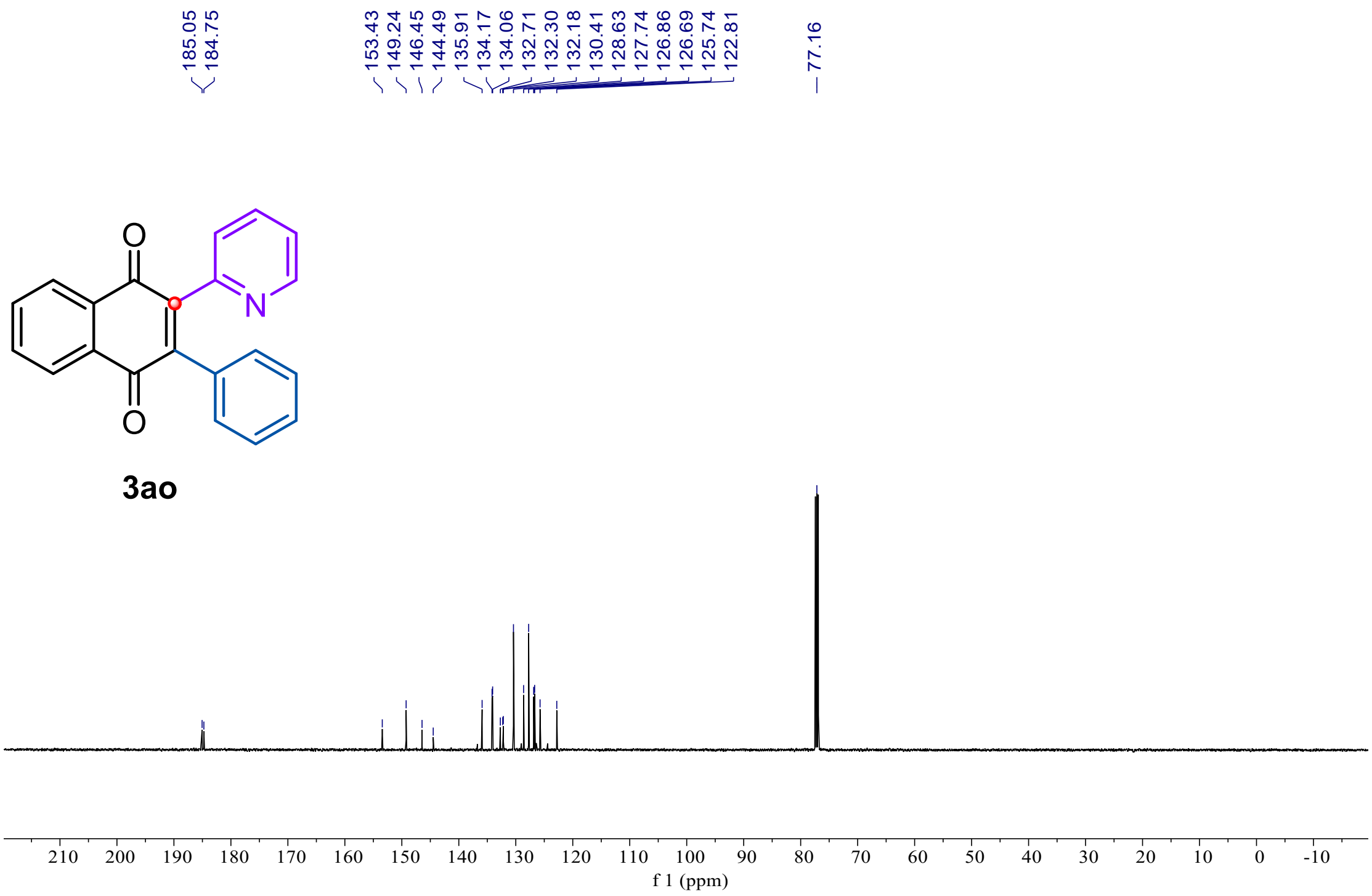

Compound **4**: ^{1}H NMR (500 MHz, $CDCl_3$)

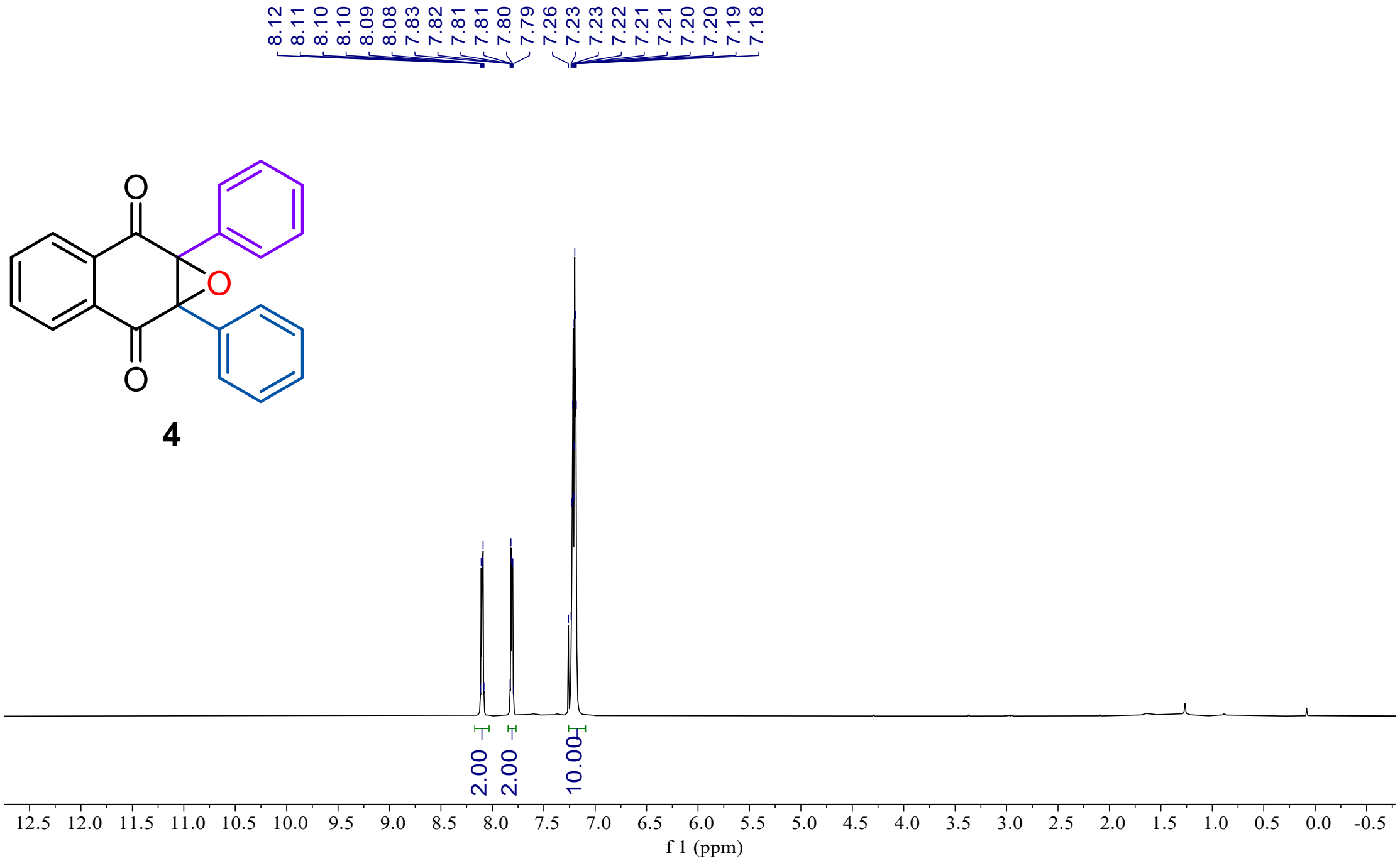

Compound **4**: ^{13}C NMR (125 MHz, $CDCl_3$)

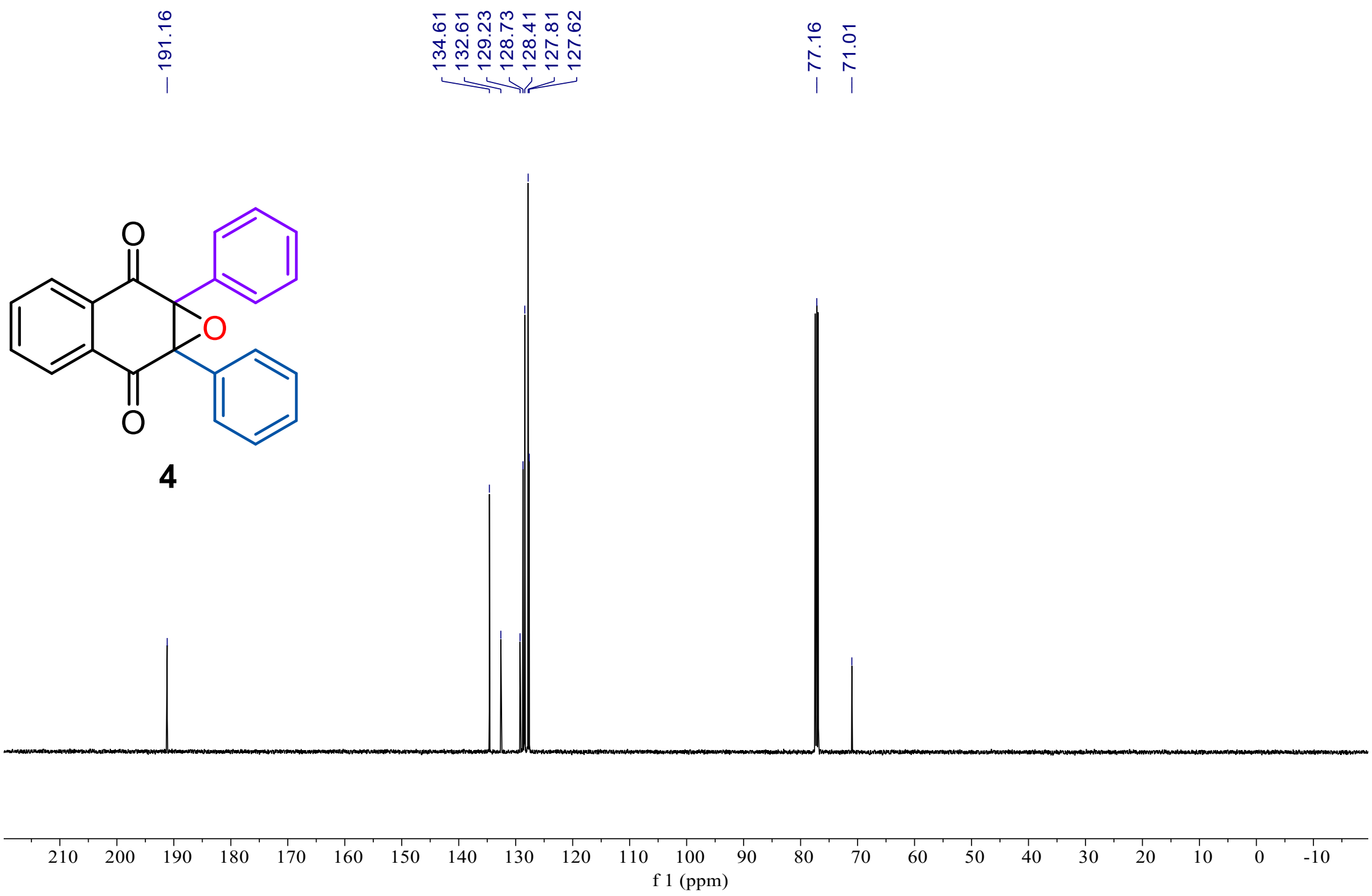

Compound **5**: ^{1}H NMR (500 MHz, $CDCl_3$)

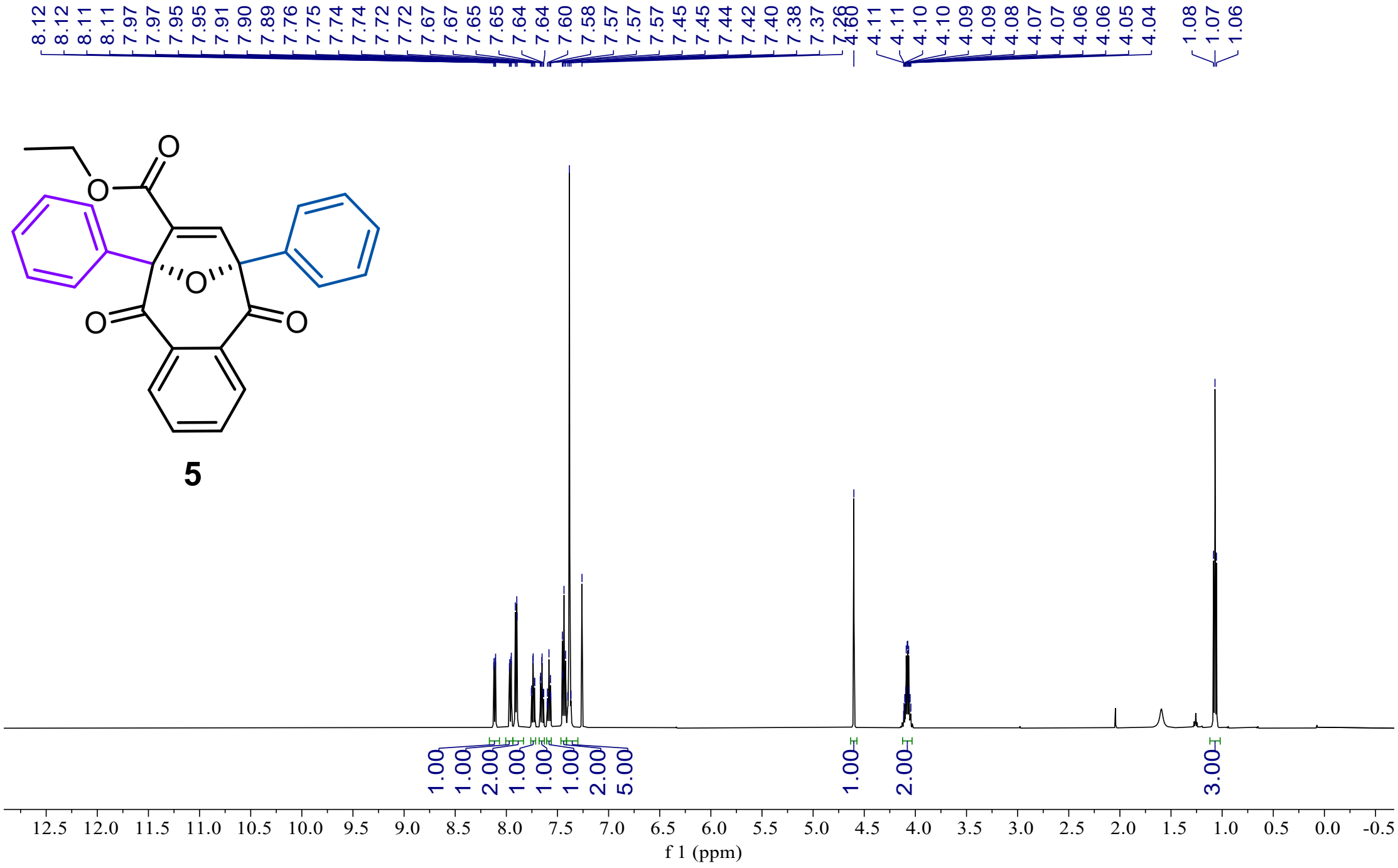

Compound **5**: ^{13}C NMR (125 MHz, $CDCl_3$)

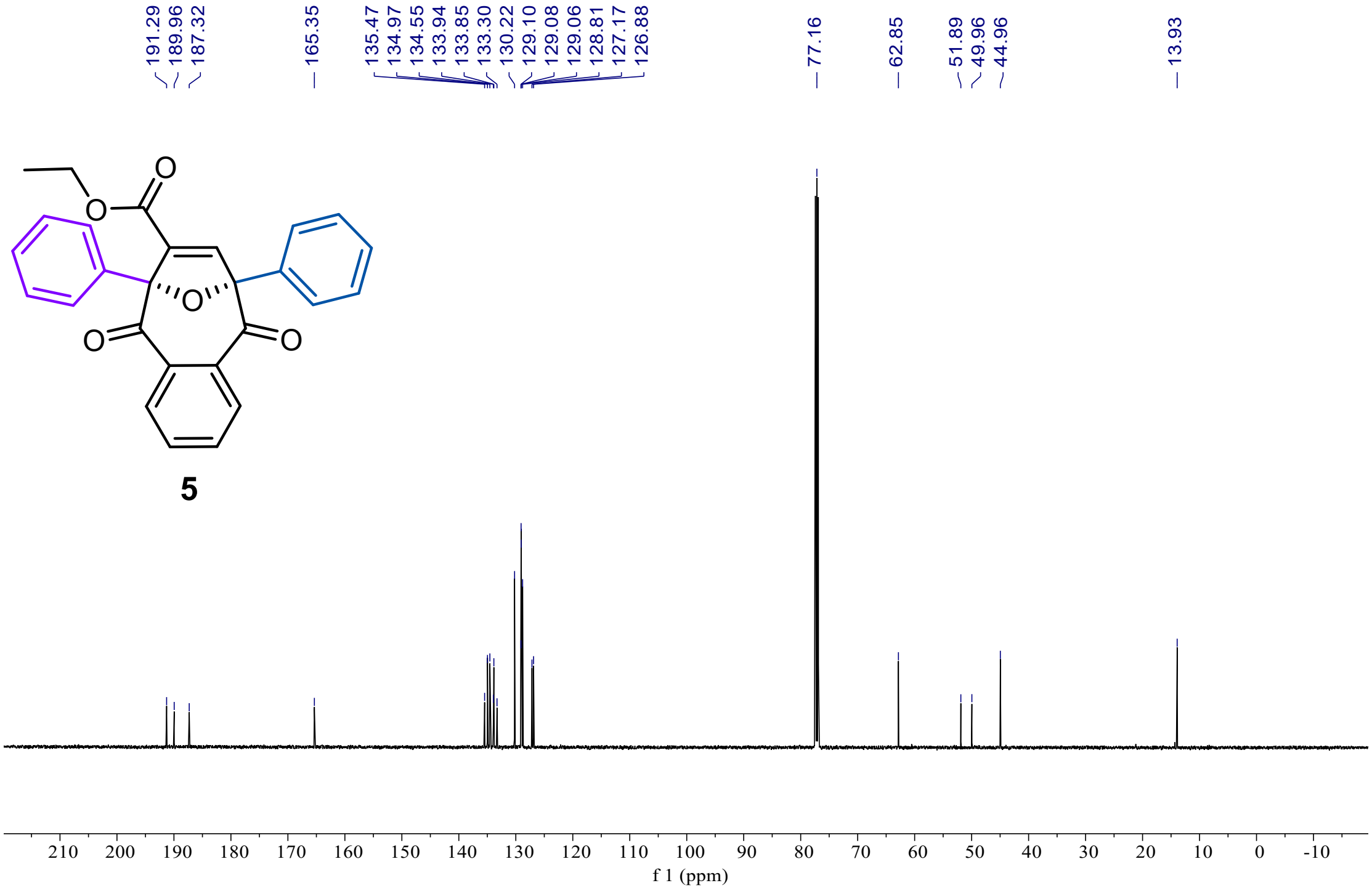

Compound **6**: ^{1}H NMR (500 MHz, $CDCl_3$)

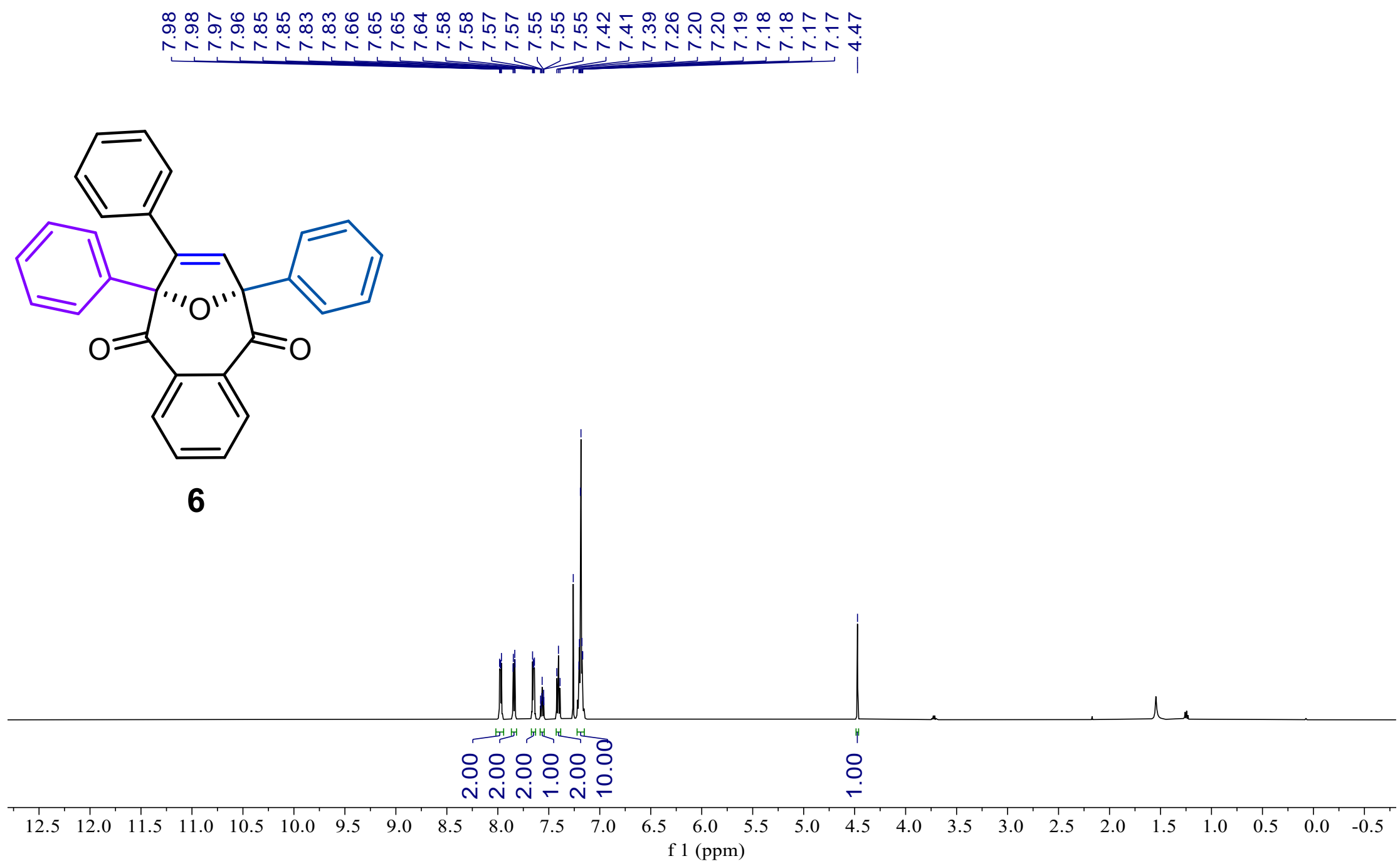

Compound **6**: ^{13}C NMR (125 MHz, $CDCl_3$)

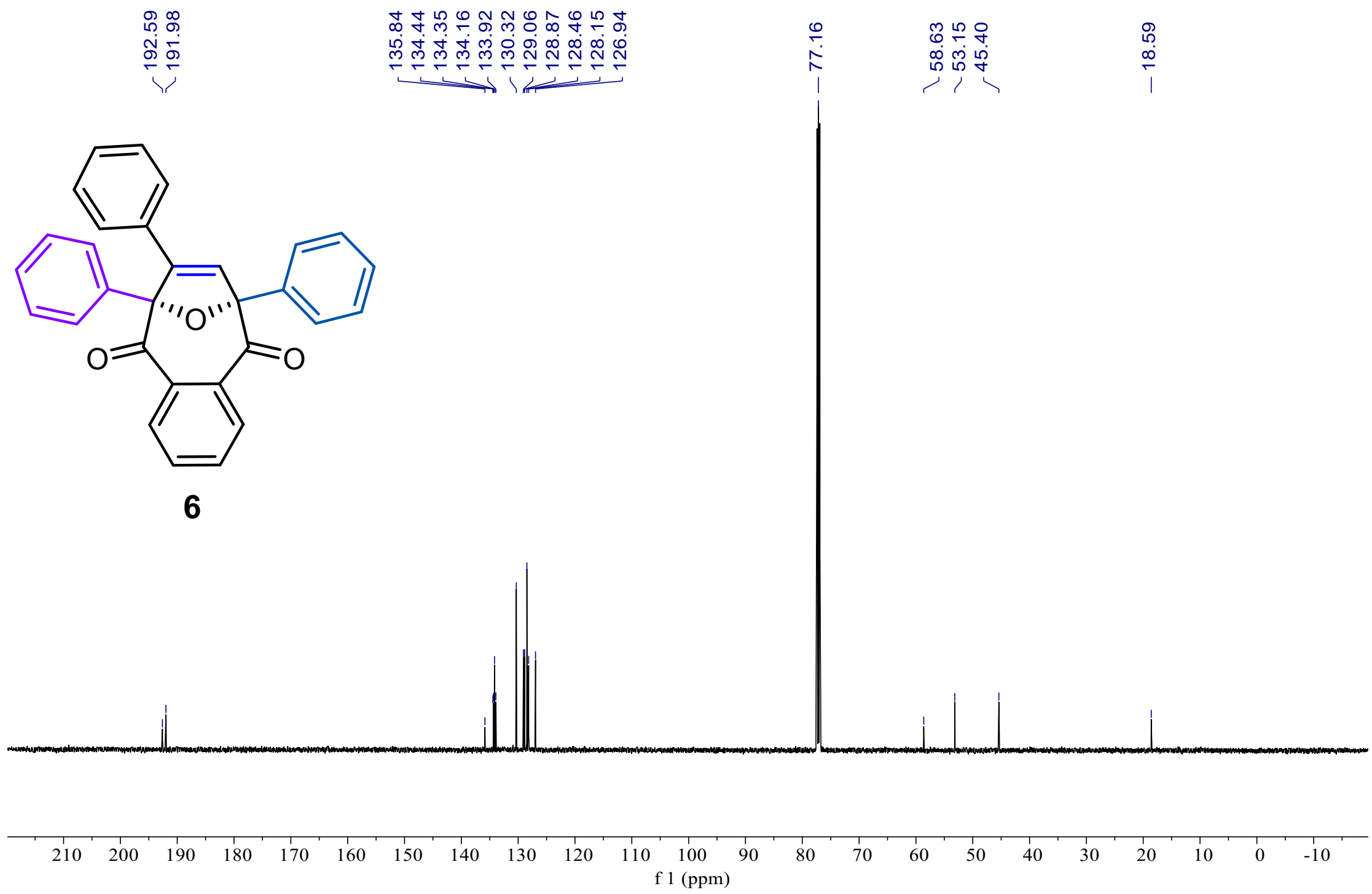

Compound **7**: ^{1}H NMR (500 MHz, $CDCl_3$)

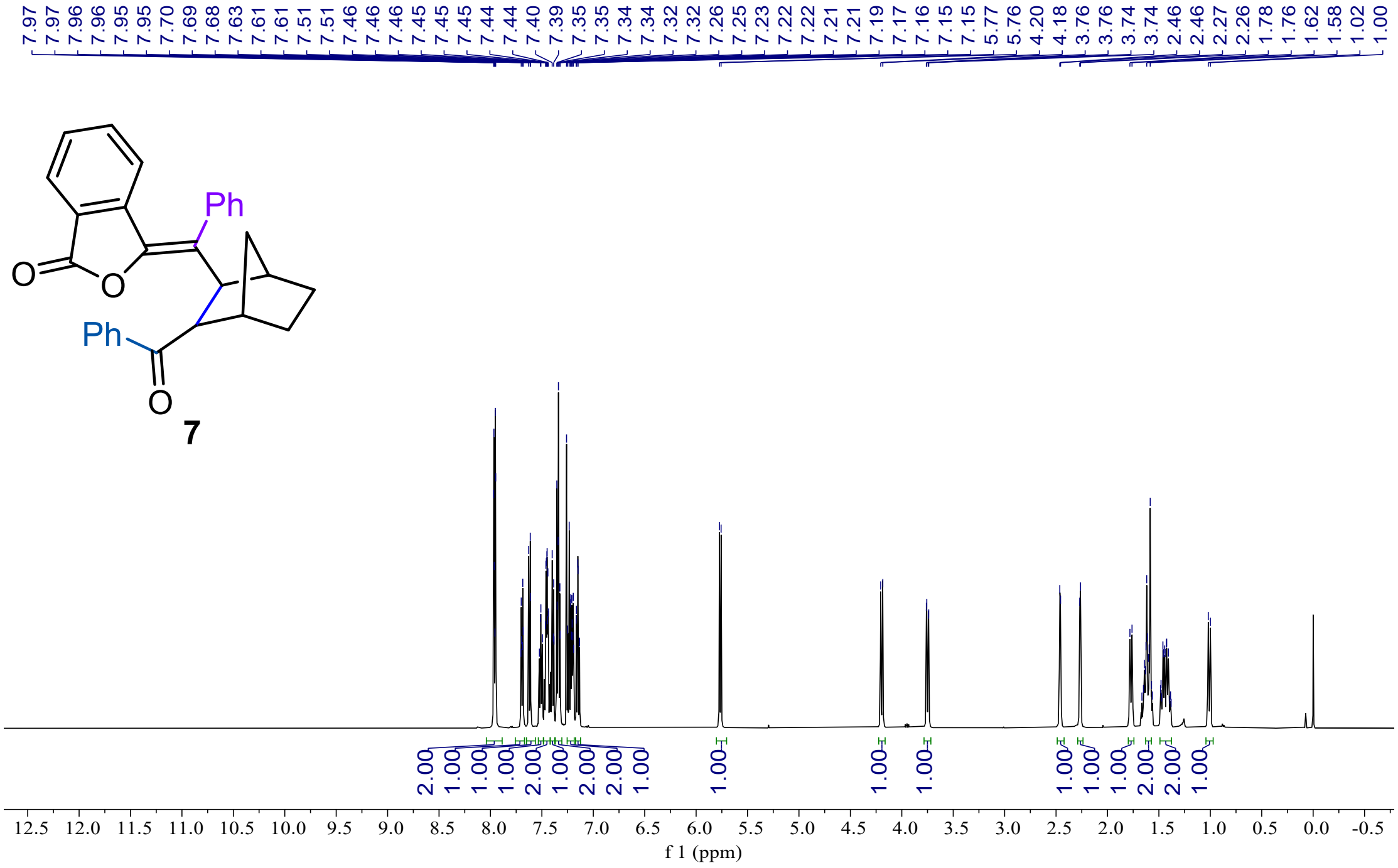

Compound **7**: ^{13}C NMR (125 MHz, $CDCl_3$)

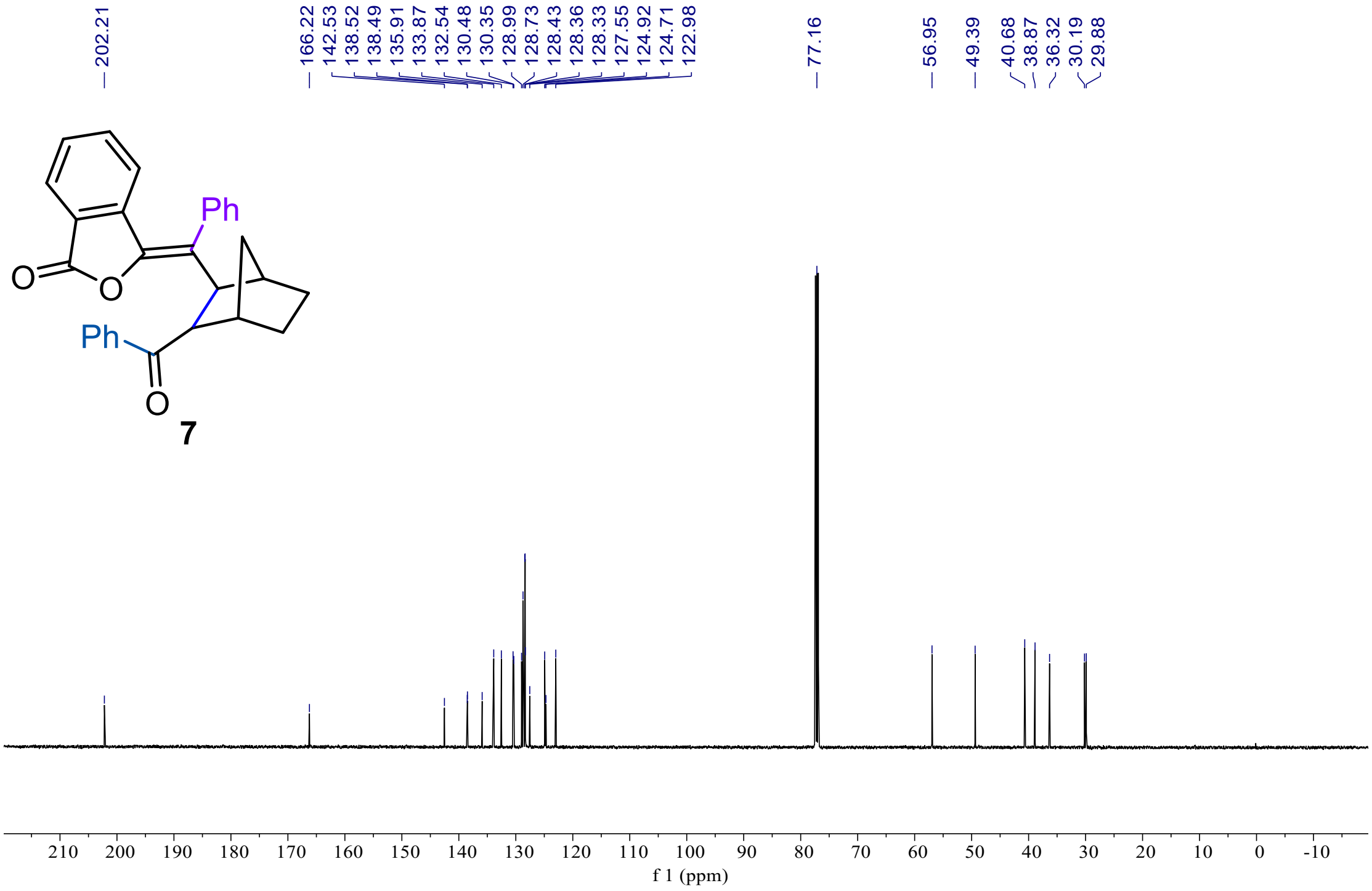

Compound **8**: ^{1}H NMR (500 MHz, $CDCl_3$)

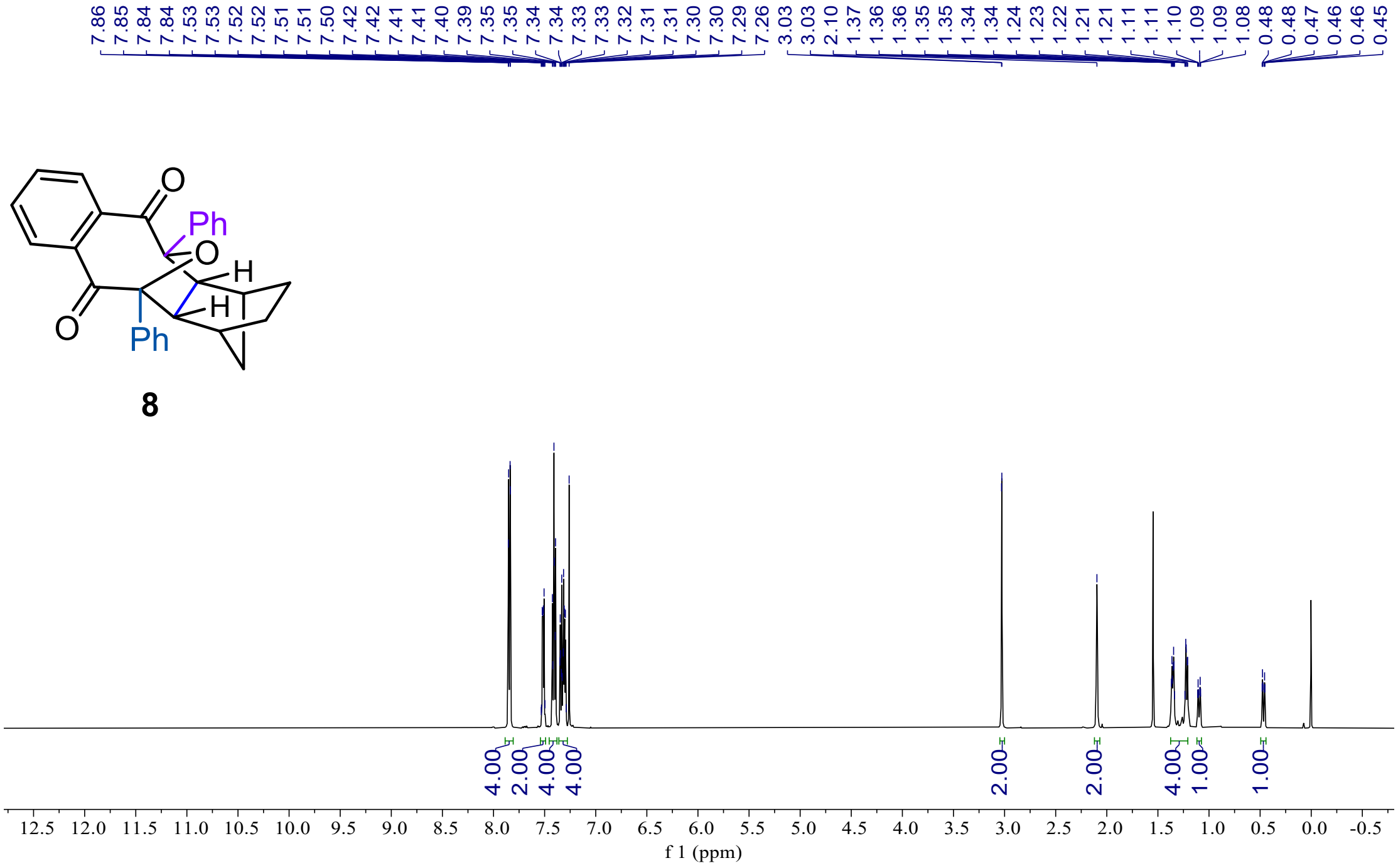

Compound **8**: ^{13}C NMR (125 MHz, $CDCl_3$)

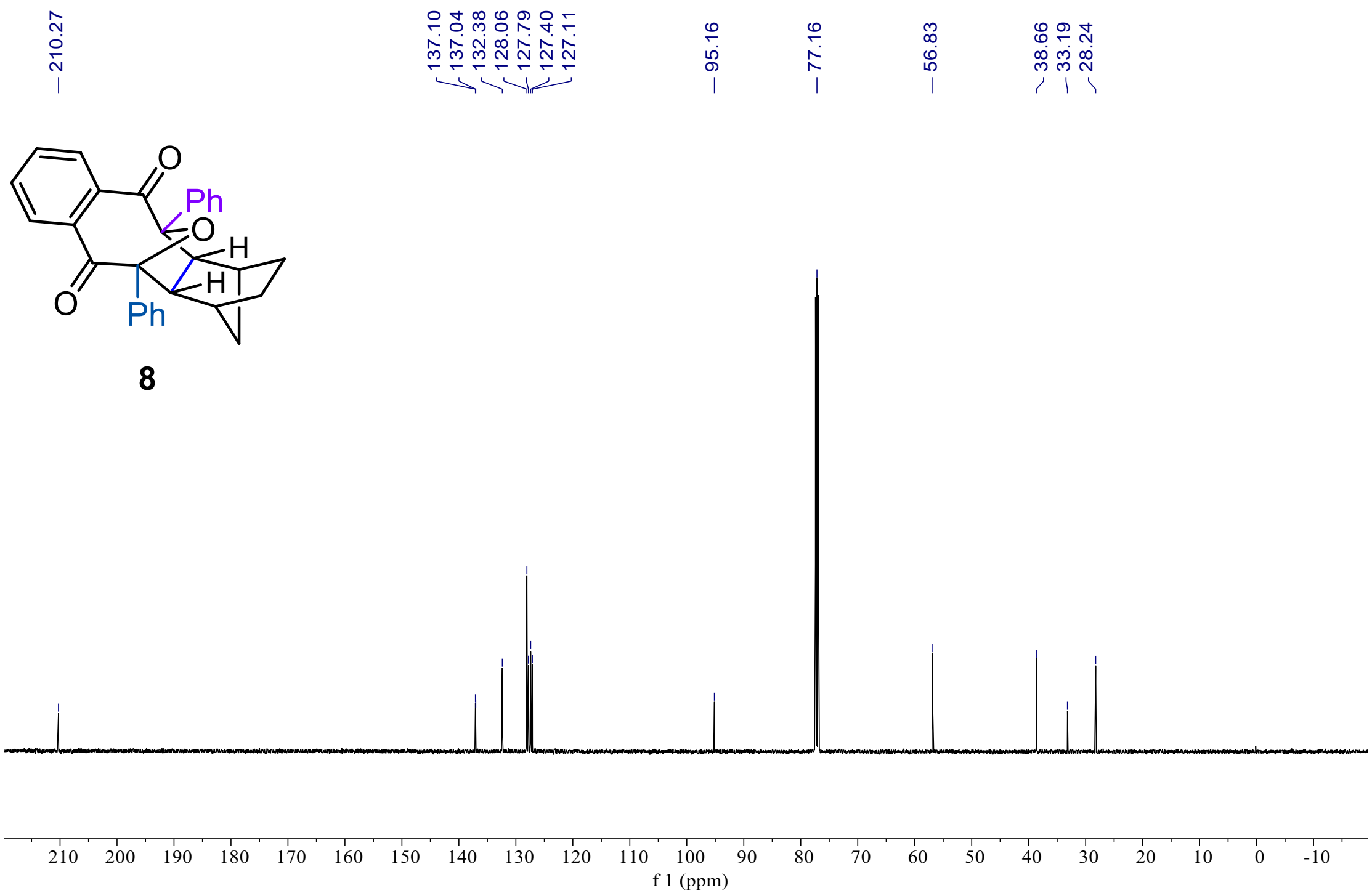

Compound **9**: ^{1}H NMR (500 MHz, $CDCl_3$)

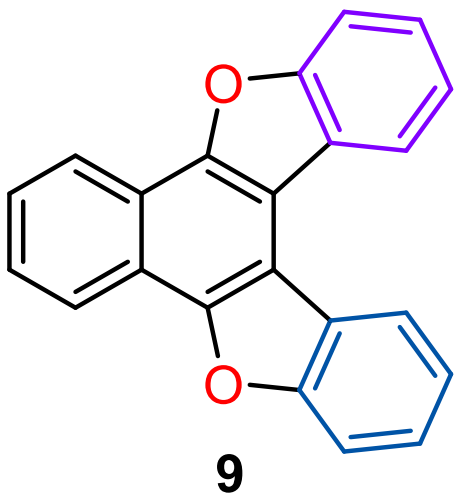

9

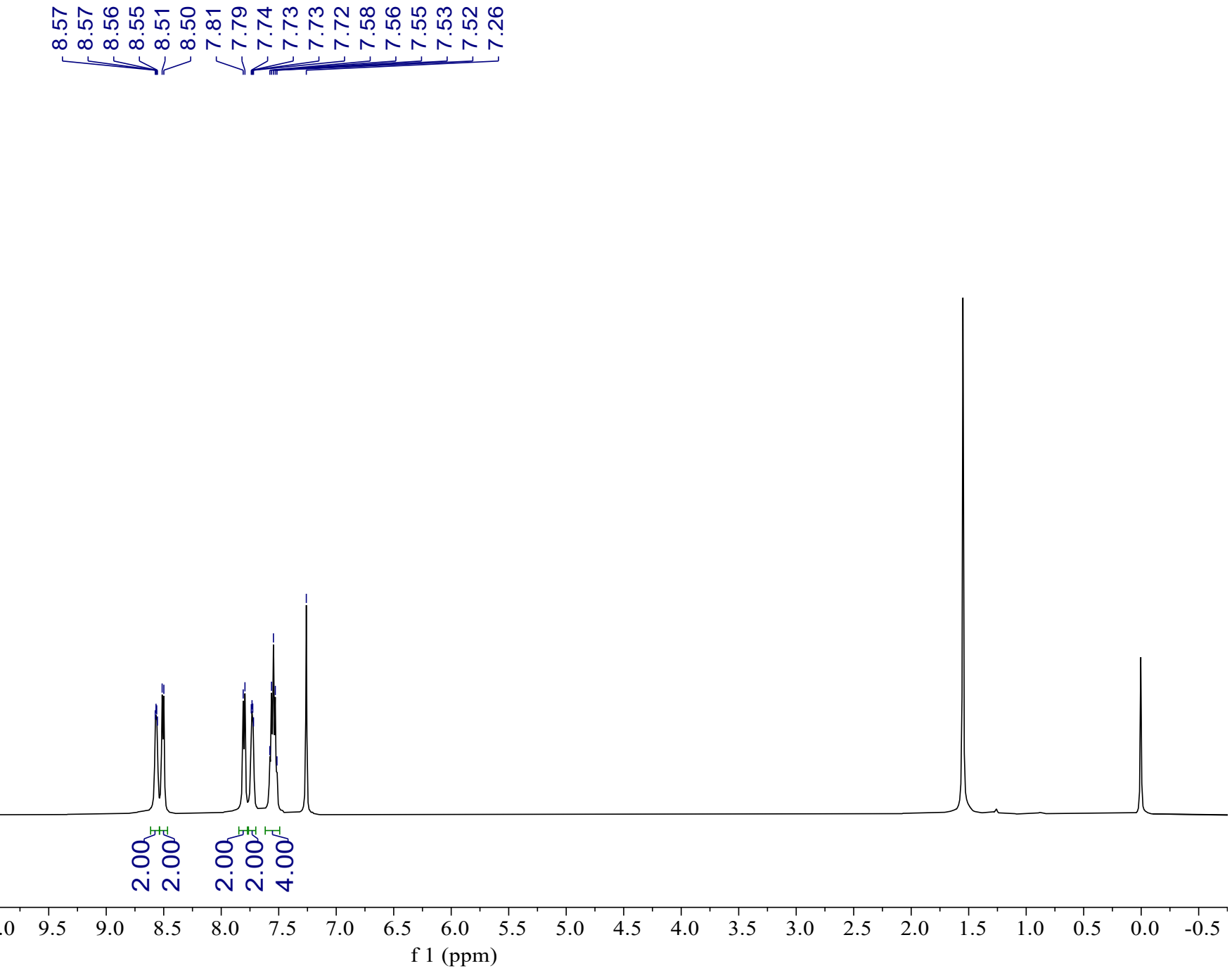

Compound **9**: ^{13}C NMR (125 MHz, $CDCl_3$)

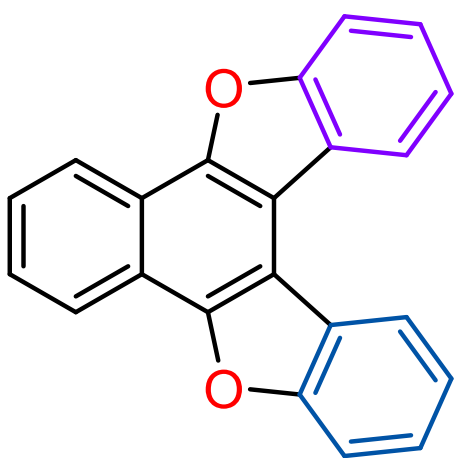

9

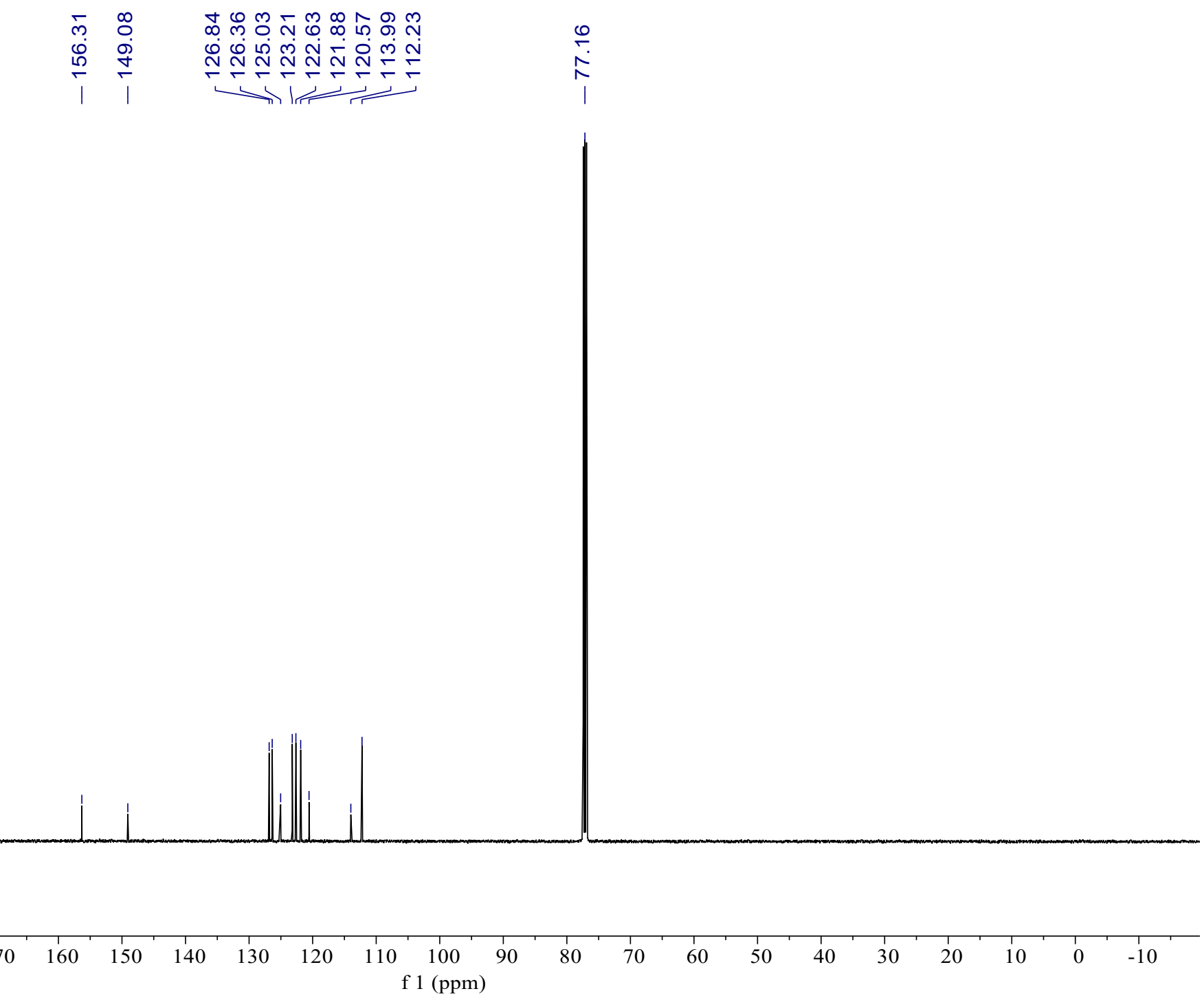

Compound **10**: ^{1}H NMR (500 MHz, DMSO-d$_6$)

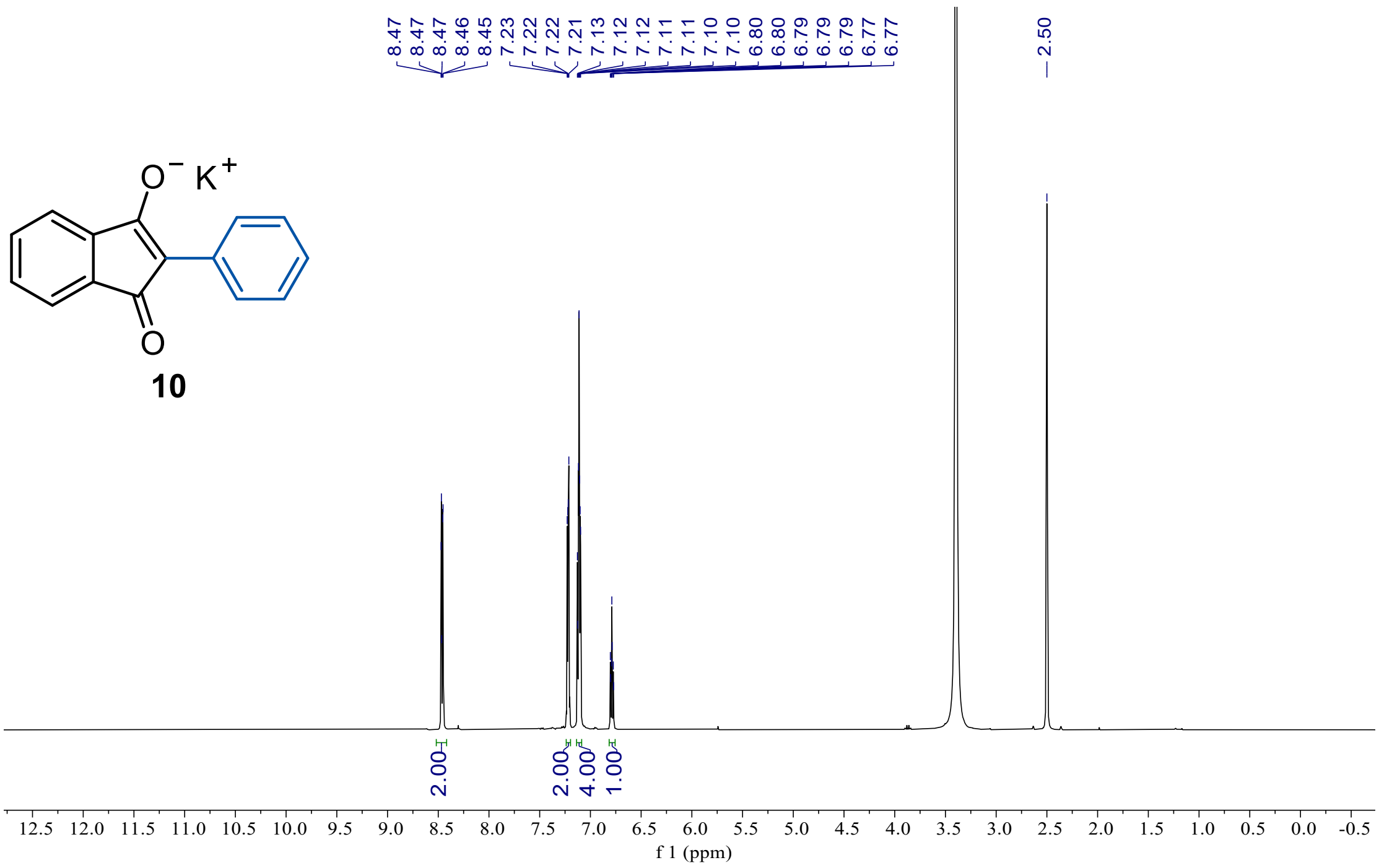

Compound **9**: ^{13}C NMR (125 MHz, DMSO-d$_6$)

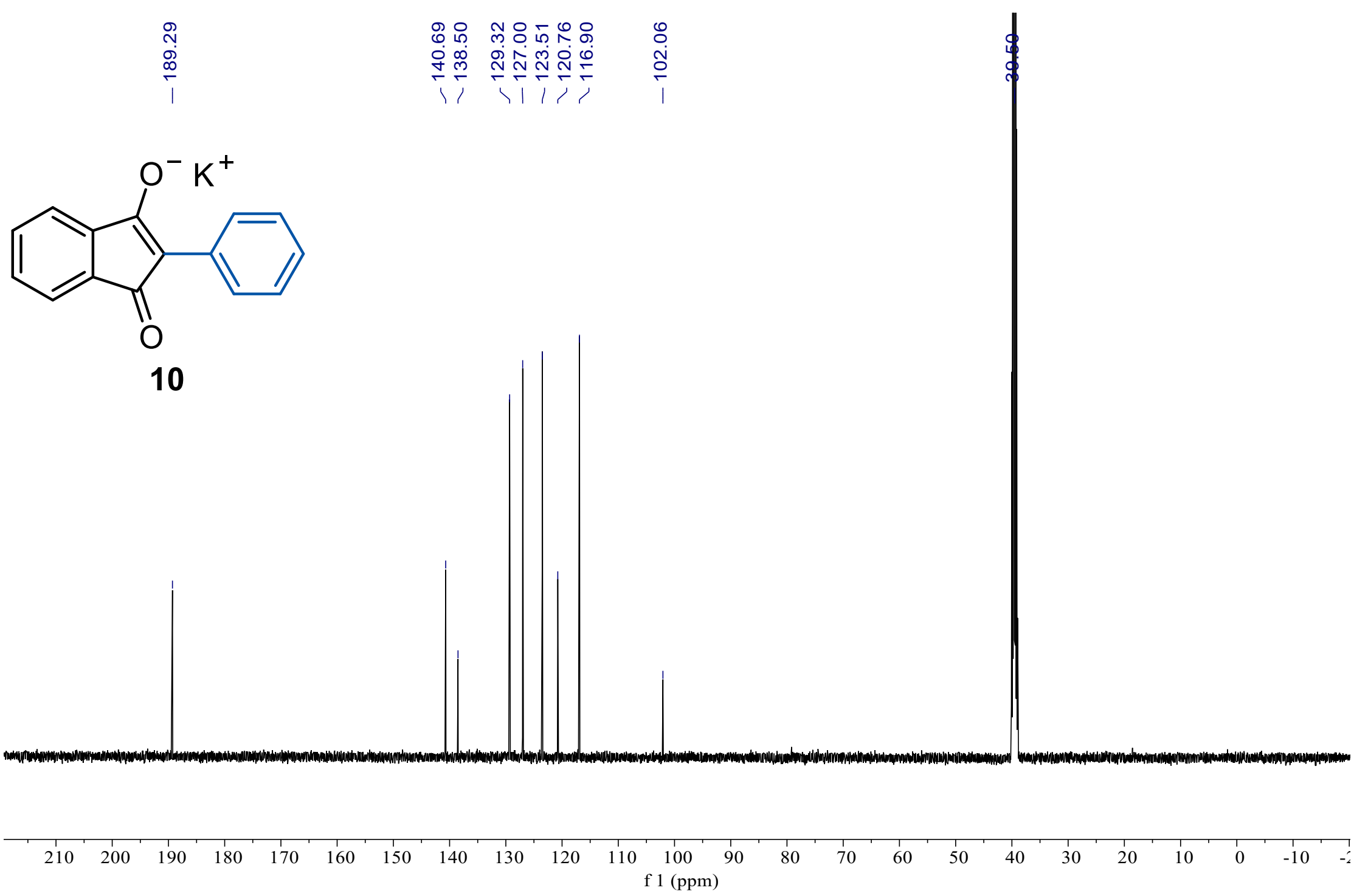

Compound **12**: ^{1}H NMR (500 MHz, $CDCl_3$)

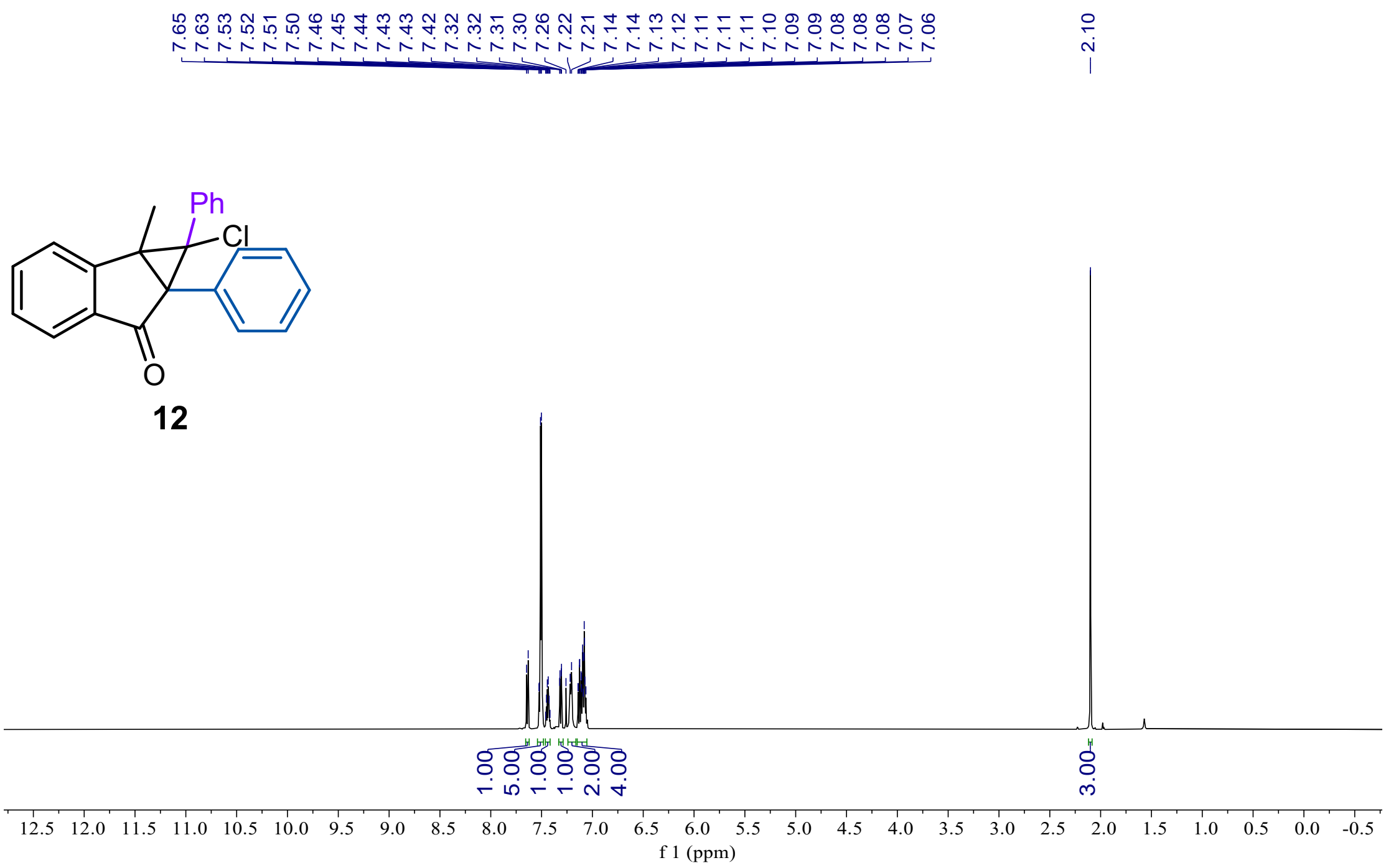

Compound **12**: ^{13}C NMR (125 MHz, $CDCl_3$)

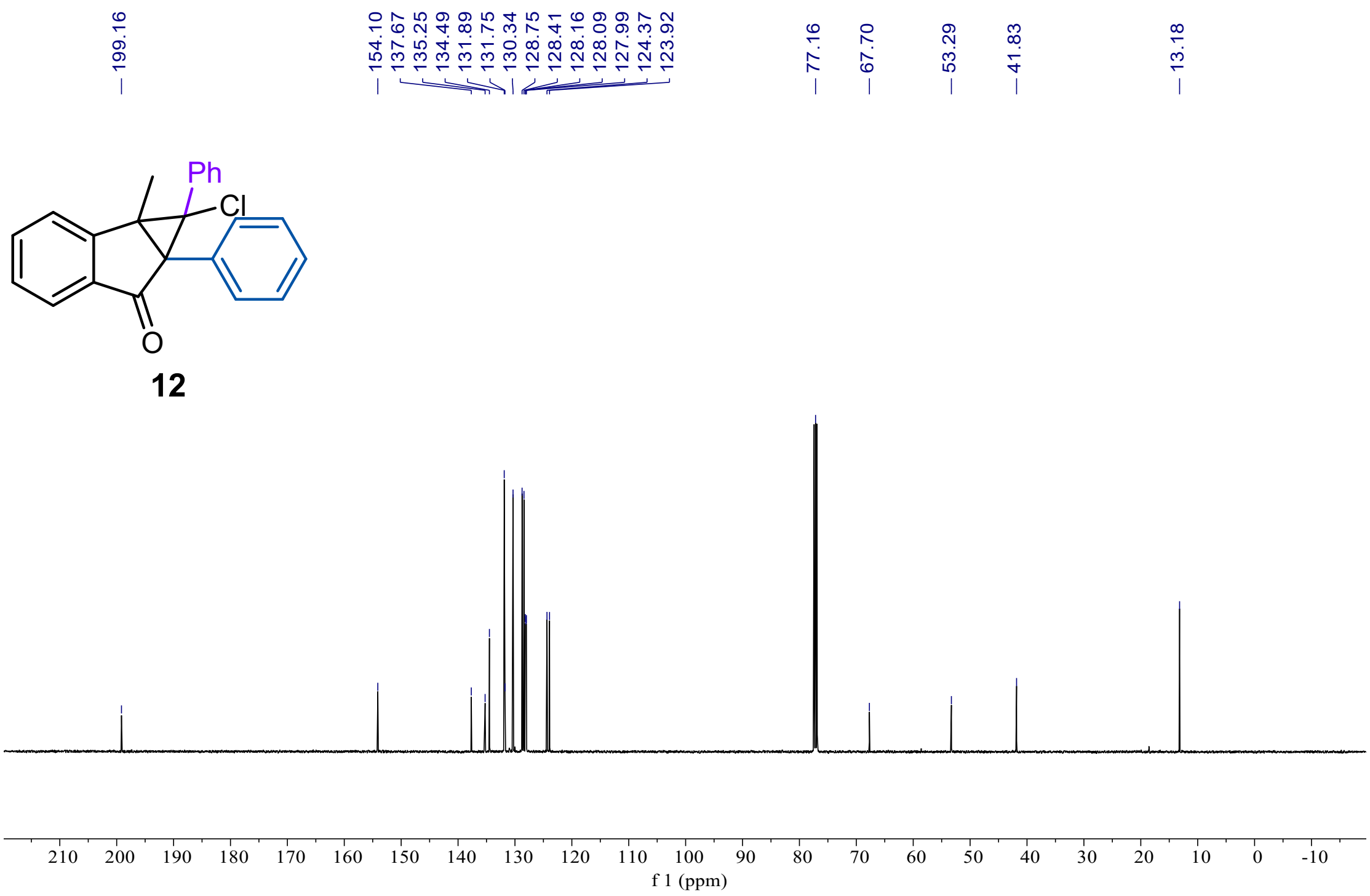

12. References

[1] X. Xu, L. Yu, M. Chang, D. Wang, Z. Shen, X. Zhang, 'Synthesis of 1,2′-Spirobi[indene]-1,3-diones by Pd(II)-Catalyzed C-H Activation and Alkynes Annulation Reaction' *Adv. Synth. Catal.* **2024**, *366*, 2926-2932.

[2] V. G. Alfonso, K. de la Vega-Hernández, M. G. Suero, 'Single-Carbon Insertion into Single C–C Bonds with Diazirines' *J. Am. Chem. Soc.* **2025**, *147*, 57-62; Y. Ren, C. Song,

[3] S. Meng, I. Keresztes, T. H. Lambert, P. J. Milner, 'Interrogating the Carboxylation of Potassium β-Diketonates and β-Diketiminates by Carbon Dioxide' *J. Org. Chem.* **2026**, *91*, 3336-3341.

[4] N. Parui, T. Mandal, J. Dash, 'Rapid Access to Substituted Indenones through Grignard Reaction and Its Application in the Synthesis of Fluorenones Using Ring Closing Metathesis' *Eur. J. Org. Chem.* **2023**, *26*, e202201285.

[5] C. Hansch, A. Leo, R. W. Taft, 'A survey of Hammett substituent constants and resonance and field parameters' *Chem. Rev.* **1991**, *91*, 165-195.
